# Predicting the pathway involved in post-translational modification of Elongation factor P in a subset of bacterial species

**DOI:** 10.1186/1745-6150-5-3

**Published:** 2010-01-13

**Authors:** Marc Bailly, Valérie de Crécy-Lagard

**Affiliations:** 1 Department of Microbiology and Cell Science, University of Florida, Gainesville, FL, USA

## Abstract

**Background:**

The bacterial elongation factor P (EF-P) is strictly conserved in bacteria and essential for protein synthesis. It is homologous to the eukaryotic translation initiation factor 5A (eIF5A). A highly conserved eIF5A lysine is modified into an unusual amino acid derived from spermidine, hypusine. Hypusine is absolutely required for eIF5A's role in translation in *Saccharomyces cerevisiae*. The homologous lysine of EF-P is also modified to a spermidine derivative in *Escherichia coli*. However, the biosynthesis pathway of this modification in the bacterial EF-P is yet to be elucidated.

**Presentation of the Hypothesis:**

Here we propose a potential mechanism for the post-translational modification of EF-P. By using comparative genomic methods based on physical clustering and phylogenetic pattern analysis, we identified two protein families of unknown function, encoded by *yjeA *and *yjeK *genes in *E. coli*, as candidates for this missing pathway. Based on the analysis of the structural and biochemical properties of both protein families, we propose two potential mechanisms for the modification of EF-P.

**Testing the hypothesis:**

This hypothesis could be tested genetically by constructing a bacterial strain with a tagged *efp *gene. The tag would allow the purification of EF-P by affinity chromatography and the analysis of the purified protein by mass spectrometry. *yjeA *or *yjeK *could then be deleted in the *efp *tagged strain and the EF-P protein purified from each mutant analyzed by mass spectrometry for the presence or the absence of the modification. This hypothesis can also be tested by purifying the different components (YjeK, YjeA and EF-P) and reconstituting the pathway *in vitro*.

**Implication of the hypothesis:**

The requirement for a fully modified EF-P for protein synthesis in certain bacteria implies the presence of specific post-translational modification mechanism in these organisms. All of the 725 bacterial genomes analyzed, possess an *efp *gene but only 200 (28%) possess both *yjeA *and *yjeK *genes. In the other organisms, EF-P may be modified by another pathway or the translation machinery must have adapted to the lack of EF-P modification. Our hypotheses, if confirmed, will lead to the discovery of a new post-translational modification pathway.

**Reviewers:**

This article was reviewed by Céline Brochier-Armanet, Igor B. Zhulin and Mikhail Gelfand. For the full reviews, please go to the Reviewers' reports section.

## Background

Protein translation is dependent on a complete set of translation factors. Elongation factor P (EF-P) is one of these factors. It is strictly conserved in bacteria and has recently been shown to have a role in translation initiation by promoting the formation of the first peptide bond [1,2]. Similarly to other initiation factors, EF-P is present at one copy per ten copies of ribosome [3,4]. The corresponding gene *efp *is essential in *E. coli *[5], but is not a required component of reconstituted *in vitro *protein translation systems [6]. EF-P is the bacterial homologue of the conserved eukaryotic/archaeal translation initiation factor 5A (eIF5A) [2] that has recently been shown to be required for ribosome translocation in a concerted action with eukaryotic translation elongation factor 2 [7]. A strictly conserved lysine of eIF5A (position 51 of the *S. cerevisiae *eIF5A protein) is post-translationally modified to hypusine [*N*^ε^-(4-amino-2-hydroxybutyl)-lysine] [8]. Hypusine is required for eIF5A function and the hypusine biosynthesis genes are essential in *S. cerevisiae *[9-14]. This unusual amino acid is not synthesized as a free intermediate but is exclusively formed by a post-translational modification of eIF5A involving two enzymatic steps. First, a deoxyhypusine synthase transfers the 4-amino butyl moiety of spermidine to the ε-amino group of the conserved Lys 51 of the *S. cerevisiae *eIF5A precursor, to form a deoxyhypusine intermediate [15,16]. This residue is then hydroxylated to hypusine by deoxyhypusine hydrolase [17]. No hypusine has ever been identified in bacteria but it has recently been shown that the *E. coli *EF-P protein contains a spermidine modification at the homologous lysine 34 [18]. The pathway that introduces this modification in the bacterial EF-P is yet to be elucidated.

Based on a combination of comparative genomics, literature mining and phylogenetic analyses, we predict that the YjeA and YjeK families of proteins are involved in EF-P modification in a subset of bacterial species. We also propose two potential mechanisms for EF-P modification, based on structural and functional characteristics of the YjeA and YjeK families of enzymes.

## Presentation of the hypothesis

### Genome organization and phyletic distribution of *efp*, *yjeA *and *yjeK*

**Figure 1:**
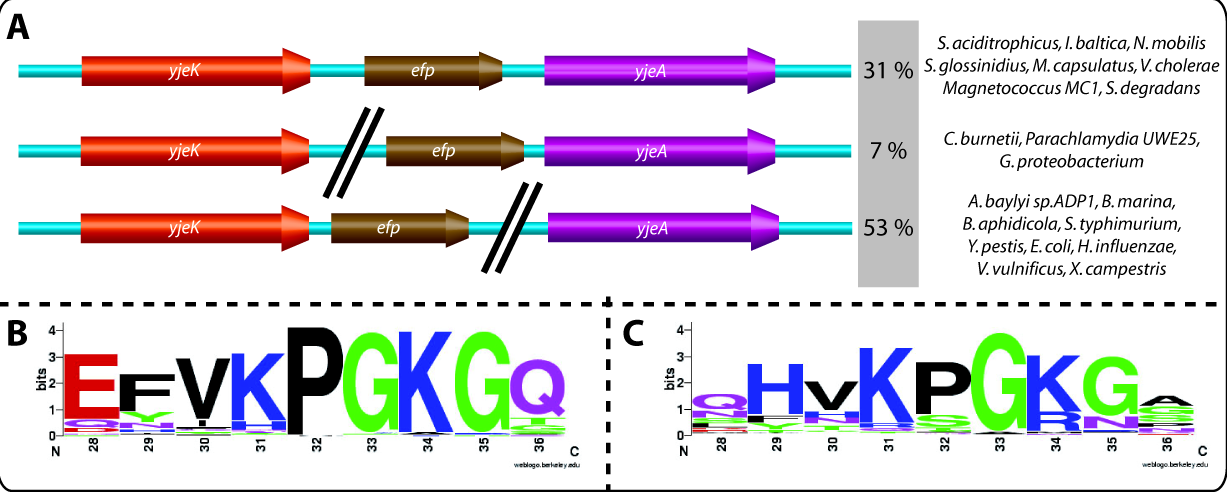
**Genomic organisation of *efp, yjeA *and *yjeK *genes**. A-Physical clustering of *efp *(in brown), *yjeA *(in pink) and *yjeK *(in orange) genes in several organisms. The black lines indicate that the genes are not contiguous in the genome. Examples of organisms and percentages for each genomic organisation among the 200 genomes that possess both *yjeA *and *yjeK *genes are indicated. The full list is given in Additional file 1. B and C- The sequence logos were created using an alignments of 9 amino acids from the EF-P protein sequences surrounding the position 34 generated in Clustal W2. The Logos were then generated by pasting the alignment in the WebLogo interface version 2.8.2 [39,40]. B- Logo for EF-P proteins from organisms that possess YjeA and YjeK (the list of sequences used is given in Additional file 3). C- Logo for EF-P proteins from organisms deprived of YjeA and YjeK.

The SEED database [19] was used to investigate the EF-P modification pathway. The results are given in the "Elongation factor P modification" subsystem in the public SEED http://theseed.uchicago.edu/FIG/index.cgi. Among the 725 bacterial genomes analyzed all contain an *efp *gene. By using the neighbourhood analysis tool of SEED database [19], we were able to establish a physical clustering association between the *yjeA*, *yjeK *and *efp *genes in 183 genomes. As shown on Fig. 1A and Additional file 1, *efp*, *yjeA *and *yjeK *genes are physically clustered in phylogenetically distant organisms. 200 genomes out of 725 analyzed (28%) possess both *yjeA *and *yjeK *genes. In 31% of these 200 genomes, the three genes are organized in an operon as in *Vibrio cholerae*. Only two of the three genes are clustered in 60% of the 200 genomes such as *efp*/*yjeA *in *Coxiella burnetii *or *efp*/*yjeK *in *E. coli*. Finally, 9% of the 200 genomes do not show any clustering between any two of these three genes. Only two organisms *Planctomyces limnophilus *and *Buchnera aphidicola *str. APS (Additional file 1) only contained a *yjeA *and not a *yjeK *homolog. These are two symbiotic organisms known to shed genes and pathways [20].

Not all EF-P proteins are predicted to be modified because some lack the conserved lysine 34 residue Fig. 1C[21]. However, 97% of the EF-P from organisms that contain homologs of YjeA and YjeK have a lysine residue at position 34 as part of the conserved "PGKG" motif (Fig. 1B). The only exceptions are organisms that contain paralogs copies of *efp *genes such as *Geobacter uraniireducens Rf4, Pelobacter carbinolicus DSM 2380 *and *Alcanivorax borkumensis SK2 *(Additional file 1). In these cases, one EF-P protein has the conserved Lys, while in the other it has been replaced by an Ala or His residue. In the organisms that lack the *yjeA*/*yjeK *pair (Fig. 1C) only 70% have kept the Lys 34 in EF-P. 24.1% have an Arg, 3.4% a Met, 2.2% a Asn and 0.3% a Gln at that position. The strong physical clustering between *efp *and *yjeA*/*yjeK *and the strict correlation between the presence of *yjeA *and *yjeK *and the presence of Lys34 in EF-P led us to propose that the corresponding gene products might be involved in post-translational modification of EF-P. This hypothesis was further explored by detailed sequence and structural analyses of the YjeK and YjeA families.

### yjeK encodes a truncated 2,3 Lysine aminomutase (LAM)

**Figure 2:**
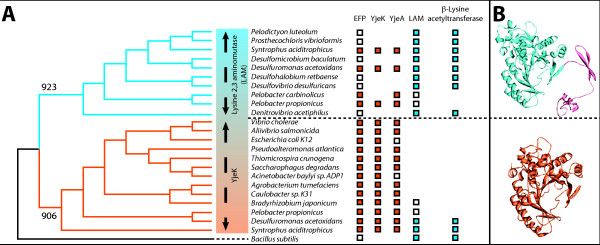
**Phylogenetic and structural analysis of the LAM family of proteins**. A- Phylogenic tree generated with a subset YjeK and LAM proteins. Methods for alignment and tree construction are described in the text. This analysis shows that YjeK (in orange) and LAM (in blue) proteins forms distinct clades with relevant bootstrap values (923 for the LAM clade and 906 for the YjeK clade). The boxes correspond to the presence of the genes encoding for the protein indicated on top of the figure in the corresponding organism, white for genes present but not involved in a clustering, orange for genes that cluster with *efp*, and blue for genes that cluster with *β-lysine acetyltransferase *(Lysine degradation pathway). Accession numbers for the protein used can be found in Additional file 1. B- Three dimensional structure of LAM from *Clostridium subterminale *SB4 [24] (PDB: 2A5H) in blue with the C-terminal multimerization domain in pink, and 3D-model of YjeK from *Acinetobacter baylyi *based on *C. subterminale *SB4. The 3D model was build by using the homology method on the SWISS-MODEL web server [41-43].

*yjeK *encodes a homologue of lysine 2,3 aminomutase (LAM) involved in lysine catabolism [22]. However, it was shown *in vitro *that *E. coli *YjeK catalyzes the conversion of (S)-α-lysine to (R)-β-lysine and not of (S)-α-lysine to (S)-β-lysine like classical LAM enzymes [22]. The *E. coli *YjeK catalytic efficiency is quite low compared to the LAM catalyzed reaction (0.1% of the activity of the *Clostridium subterminale *SB4 LAM [22]). (S)-α-lysine might not therefore be the real *in vivo *substrate of the YjeK enzyme. Primary sequence alignment analysis on 95 LAM/YjeK homologs were performed using Clustal W2 [23] and revealed that the LAM (YjeK) that clusters with the *efp *gene can be separated from the canonical LAM involved in Lys degradation pathway. The major difference between the two families is that the YjeK proteins lack the C-terminal multimerization domain present in the LAM family of proteins [24] (Fig. 2B). Further phylogenetic analysis on 24 LAM/YjeK homologs was performed on 118 amino acid sequences present in the N-terminus active site and conserved between the YjeK and LAM subfamilies (Fig. 2A). The amino acid sequences were aligned using the ClustalW2 algorithm with default parameters [23]. Phylogenetic analyses were carried out by employing the Phylip 3.68 program package [25]. Distance-based matrices were generated between all pairs of sequences using the Jones-Taylor-Thornton matrix as employed in Protdist (Phylip). Phylogenetic trees were generated from these matrices using the neighbour-joining method as implemented in Neighbor (Phylip). Reliability of branches was determined with the bootstrap method of 1000 replicates using Seqboot (Phylip). The final tree was generated with Consense (Phylip). This analysis showed that despite the fact that YjeK from *E. coli *is 33% identical to LAM from *C. subterminale *SB4, the two enzyme subfamilies form two distinct clades on the YjeK/LAM phylogenetic tree separated by bootstrap scores ranging from 923 to 906 (Fig. 2A). Genome neighbourhood analyses were also used to split the two families. We observed that *ablA *(LAM) genes physically cluster mainly with other lysine degradation gene such as *ablB *(β-lysine acetyltransferase) in several organisms (See Fig. 2A and Additional file 1). On the contrary, *yjeK *genes cluster mainly with *efp *homologs but never with *ablB *genes (See Fig. 2A and Additional file 1). In genomes such as *Syntrophus aciditrophicus *and *Desulfuromonas acetoxidans*, where both *yjeK *and *ablA *genes are present, one was found to cluster with *efp *and the other with *ablB *respectively (Fig. 2A). The combination of structural, phylogenetic and physical clustering pattern differences between YjeK and LAM enzymes were used to split the LAM family of proteins into two subfamilies that will be referred to as LAM and YjeK from hereon and suggest that these families have distinct functions, the first in lysine catabolism, the second related to EF-P.

### yjeA encodes a truncated Lysyl-tRNA synthetase of Class II

**Figure 3:**
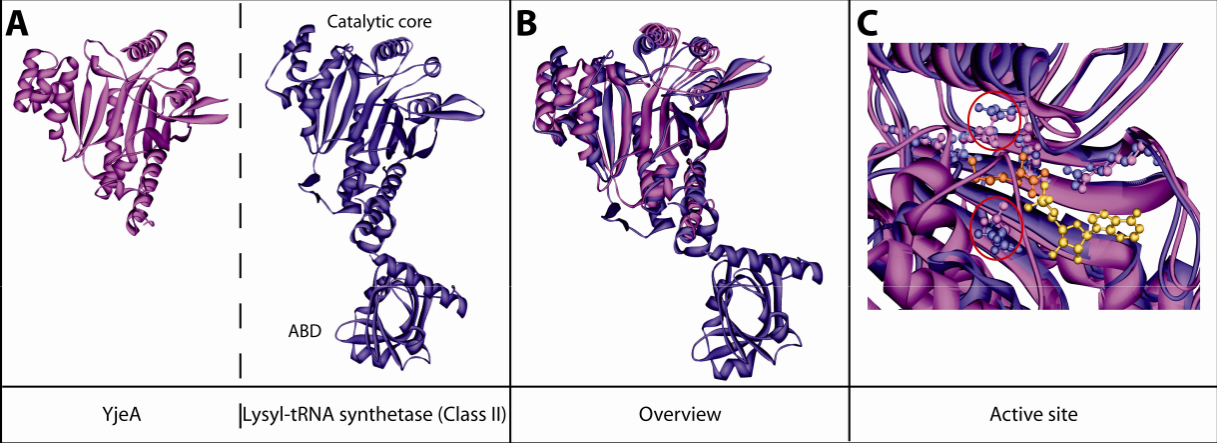
**Structural analysis of YjeA protein family**. A- Three dimensional structure of YjeA from *Salmonella typhimurium *(PDB: 3G1Z) in pink and class II lysyl-tRNA synthetase (LysRS) from *Escherichia coli *[26] (PDB: 1BBU) in purple. The domains that constitute LysRS are indicated: catalytic core and anticodon binding domain (ABD). B- Merging of the two previous global structures and zoom into the active site and catalytic residues. The residues responsible for lysine binding are in pink for YjeA and in purple for the LysRS2. The lysine substrate present in LysRS2 structure is indicated in orange and the AMP present in YjeA structure is indicated in yellow. Red circles highlight the residues for which the 3 dimensional positions are not conserved.

*yjeA *in *E. coli *encodes a homolog of Class II lysyl-tRNA synthetase (LysRS2) sharing 31% identity with the canonical *E. coli *LysRS2 [26] (Additional file 2). Pleiotropic phenotypes have been associated with the absence of this protein [27,28] but its exact function is not known. Analysis of the three dimensional structure of *E. coli *YjeA (Fig. 3A) revealed that this protein contains only the catalytic core of LysRS2 deprived of the anticodon binding domain (ABD), responsible for tRNA^Lys ^recognition (Fig. 3B). The structural alignment of YjeA and LysRS2 from *E. coli *shows that their catalytic cores are very similar: the lysine binding residues are conserved and in the same spatial location [except for two residues surrounding the lysine substrate that are slightly displaced in the catalytic pocket (red circles in Fig. 3C)]. The absence of the anticodon binding domain YjeA points to a function different from tRNA aminoacylation as already observed for other aminoacyl-tRNA synthetase (aaRS) catalytic core homologs. Examples include the catalytic core homolog of *E. coli *glutamyl-tRNA synthetase (Glu-Q-RS) involved queuosine glutamylation of the tRNA^Asp ^anticodon [29-32], and the catalytic core homolog of *Pyrococcus abyssi *AsnRS2 (archaeal Asn synthetase, AS-AR) involved in asparagine biosynthesis [33].

### Potential mechanisms involved in EF-P modification

The analysis presented above led to the hypothesis that YjeK and YjeA are involved in the modification of Lys34 of EF-P to a spermidine-like molecule [18]. We propose two possible mechanisms for the insertion of this modification.

**Figure 4:**
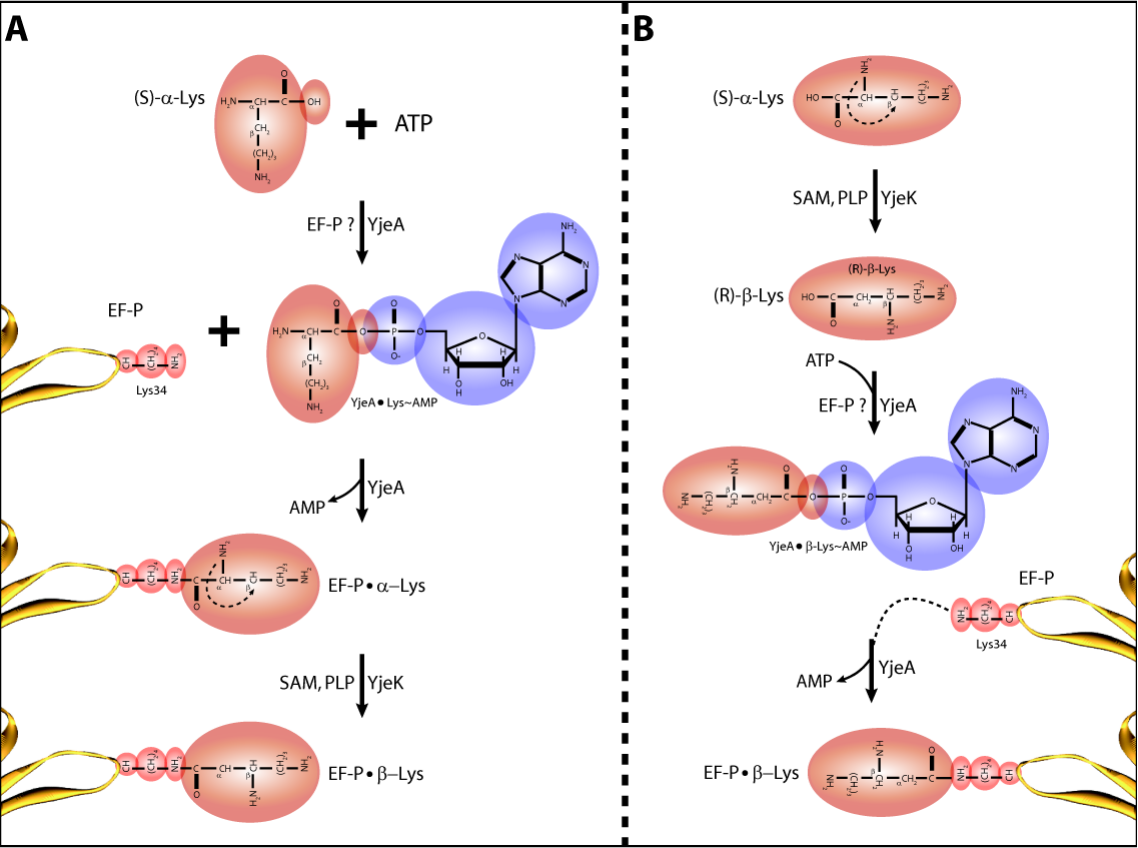
**Potential EF-P modification pathways**. A- Mechanism in which YjeA acts first on free lysine (Lys) and attaches it to EF-P Lys34 which is then modified on EF-P into β-lysine by YjeK. B- Mechanism in which YjeK acts first to modify free lysine into β-lysine which is subsequently activated by YjeA and attached to EF-P lysine 34. EF-P N-terminal loop is indicated in yellow, Lys 34 is indicated in red, the modification appear on light brown and the AMP generated by YjeA during the activation of the Lys residue appear in blue. Potential and known substrates and cofactors of each enzyme are indicated.

In the first model (Fig. 4A), YjeA activates (S)-α-lysine into (S)-α-lysyl•AMP in presence of ATP and magnesium. This is coherent with the presence of AMP in the YjeA three dimensional structure (PDB: 3G1Z) and the conservation of the lysine binding residues in the YjeA active site (Fig. 3C). The activated (S)-α-lysine could then be transferred to the conserved Lys34 of EF-P to form an iso-peptidic bond between the Lys34 εNH_2 _and the α-COOH of the activated Lys. The ability of activated amino acids to make isopeptidic bonds with the εNH_2 _moiety of Lys lateral chains has already been demonstrated for *S. cerevisiae *AspRS [34]. Finally, YjeK could convert the EF-P bound α-lysine into β-lysine in presence of S-adenosyl methionine (SAM) and pyridoxal phosphate (PLP) (Fig 4A). The low activity of the *E. coli *YjeK on free (S)-α-lysine noted above could be due to the fact that the natural substrate is linked to EF-P [22]. The absence of the C-terminal dimerization domain in YjeK might allow it to access the EF-P-bound substrate.

In the second model (Fig. 4B), YjeK would first convert the (S)-α-lysine into (R)-β-lysine in presence of SAM and PLP [22] with a low activity to prevent potential use of (R)-β-lysine by other enzymes and a potential toxicity of this intermediate in cellular metabolism. Then YjeA would recognise (R)-β-lysine as a substrate and activate this non cognate amino acid in presence of ATP and magnesium prior to its transfer on the EF-P Lys34. The use of non cognate amino acid by aaRS or truncated aaRS is well known [35] and the fact that YjeA active site is slightly different than the LysRS2 site may allow for the accommodation (R)-β-lysine.

Both reactions scheme would generate an EF-P modified on Lys34 by a β-lysine (146 Da) compatible with the 144 Da modification that was experimentally observed [18].

## Testing the hypothesis

Our hypotheses could be investigated *in vivo *by using a strain of *Acinetobacter baylyi *sp. ADP1 in which the *efp *gene has been replaced by a tagged version by using genomic replacement as described previously in Metzgar et al [36]. The tag would allow the purification of EF-P from the crude extract of *A. baylyi *and the subsequent analysis of the factor by mass spectrometry. Then the *yjeK *or *yjeA *genes could be deleted in this strain and the EF-P purified to analyze the factor for the presence or the absence of the modification.

This method seems to be an ideal way to test our model, but several issues make it technically challenging. First, EF-P is present in the cell in a lower ratio than the elongation factors (one EF-P per ten ribosomes [4,37]), thus EF-P might always be in complex with the ribosome and inaccessible for our affinity purification method. Moreover, the method used to release the EF-P bound to purified ribosomes requires the use of high salt concentration and low magnesium concentration, but high salt concentration is not compatible with most affinity purification and leads to the loss of 80% of the factor during the purification step. Finally, deleting *yjeK *and *yjeA *in *A. baylyi *gives rise to severe growth phenotypes [36] that decreases the amount of EF-P available for the affinity purification step and makes the *in vivo *approach difficult to realize.

Our hypothesis could also be tested using an *in vitro *approach. EF-P would need to be purified in the apo form from a Δ*yjeK *or Δ*yjeA E. coli *strain. Then, the YjeA and YjeK enzymes could be over-expressed and purified and the putative activities presented in Fig. 4 could be followed by using radiolabeled (S)-α-lysine as a substrate.

## Implication of the hypothesis

Using a comparative genomic approach, we predict that the *yjeK *and *yjeA *genes of unknown function that physically cluster with *efp *gene are potential candidates of EF-P modification genes. We propose two potential mechanisms based on further structural and functional data mining and analysis of YjeK and YjeA protein families. However, among the 725 bacterial genomes used in our analysis, only 200 (28%) possess both *yjeA *and *yjeK *genes. The taxonomic distribution of these organisms spans several phyla but are mainly represented by members of the protebacteria (gamma, alpha and delta) and by scattered representatives of the Aquificales, Chlamydiae, Chlorofexi, Planctomycetes and Sphirochetes phyla (Additional file 1). It is totally absent in Actinomycetes, Firmicutes and Cyanobacteria (Additional file 1). A more extensive phylogenetic analysis beyond the scope of this article could establish if the common ancestor of bacteria had a EF-P modification pathway that was lost in specific phyla or if it appeared later on, as for example in the proteobacteria ancestor, and then was horizontally transferred in other clades.

In organisms that lack the *yjeK*/yjeA genes only 70% of the EF-P proteins have retained a Lys residue at the 34 position (Fig. 1C). This implies that the translation machinery can adapt to the lack of EF-P modification. Indeed, this adaptation happens as shown for *Thermus thermophilus *EF-P [38]. In this case, an arginine (Arg32) replaces the corresponding *E. coli *Lys34. This Arg32 lies close to the peptidyl transferase center (PTC) and interacts with the C75 position of the CCA end of the acceptor stem of the initiator tRNA and with the phosphates of positions C2064 and C2065 of the 23*S *ribosomal RNA (rRNA) [38]. The authors propose that the hypusine moiety in eukaryotes/archaea and the spermidine (or β-lysine) moiety in bacteria could allow closing of the distance between EF-P and the PTC and enable proper positioning and stabilization of the initiator tRNA in the peptidyl site of the ribosome [38]. How *T. thermophilus *and other organisms that lack the modification achieve this is not known but favors an adaptation of the translation machinery, particularly of the 23*S *rRNA and initiator tRNA. Finally, our hypotheses if confirmed will lead to the discovery of a new post-translational modification mechanism, β-lysinylation.

## Competing interests

The authors declare that they have no competing interests.

## Authors' contributions

MB did the detailed alignment analysis and all the figures and proposed the mechanisms. VdCL performed the comparative genomic analysis and made the functional predictions. Both authors wrote the paper and read and approved the final manuscript.

## Reviewers'reports

### Reviewer 1

Céline Brochier Armanet, |Laboratoire de Chimie Bacterienne (CNRS - UPR9043) Marseille, |France

In the paper entitled "Predicting the pathway involved in post-translational modification of Elongation factor P in a subset of bacterial species" Bailly et de Crécy-Largard identify two genes (*yjeA *and *yjeK*) as candidates for the posttranslational modification of lysine 34 of EfP to a spermidine derivative. This prediction is based on various *in silico *analyses (genomic clustering of Ef-P coding genes, the presence or the absence of a lysine at the targeted position in Ef-P protein, functional annotations, structural comparisons etc).

I think that the work presented is very convincing and is a good illustration of the predictive power of *in silico *approaches for functional prediction. Accordingly I strongly recommend its publication in Biology Direct.

However, I have few suggestions that may increase the value of the work (especially from an evolutionary point of view).

A) The authors say that EfP is strictly conserved in Bacteria and accordingly they quote two references. However, these papers are "old" from a genomic point of view (e.g. in 1992, less than 70 bacterial genomes were available and many bacterial phyla were not represented in genomic databases). It would be interesting to verify that this important assumption is still verified by available data.

***Authors' response: ****In the revised version of the manuscript we included a sentence stating that efp was found in all 725 genomes analyzed. And two additional files *3 and 4* provide all the EF-P sequences used in the analysis*.

### Reviewer 1

B) Also important is the presence of the lysine at position 34. The authors say that some EfP sequences lack this residue quoting a paper published in 1992. As previously, I think it would be interesting to precise how many and which EfP sequences harbour this conserved lysine and their taxonomic distribution across bacterial phyla.

***Authors' response: ****In the revised version we provided an additional figure *(Fig. 1B) *that shows that the EF-P Lys-34 residue is nearly strictly conserved in all the organisms that have the yjeA/yjeK pair. The only exceptions are found in organisms that have two EF-P encoding genes (one with the conserved lysine, one without). The corresponding list of sequences is given in S2. Conversely*, Fig. 1C* shows that in organisms that lack yjeA/yjeK the Lys can be replaced by other residues (Arg, Met, Asn or Gln). Here again the corresponding protein sequences are given in S3. A section of the taxonomic distribution of organisms that have or not the yjeA/yjeK pair has been added to the implication section and this information is also included in Additional file *1.

### Reviewer 1

C) More important, based on the SEED database the authors say that 125 on 722 bacterial genomes analysed harbour both *yjeA *and *yjeK *genes. I think it is very important to provide and to comment their taxonomic distribution: are these genomes closely related? Or are they well distributed among bacterial phyla? Even if I think that this will not change the main conclusions of the paper, I think it would be interesting to develop this point. Indeed, these two situations have very different evolutionary implications. The presence of these two genes only in closely related genomes may suggest a recent appearance of this modification during bacterial evolution. On the contrary, if these genes are present in distantly related genomes (e.g. genomes from different bacterial phyla), this may point to either an ancient origin of this modification in Bacteria followed by numerous independent secondary losses, or to a recent origin followed by horizontal gene transfer across bacterial phyla. To my point of view, the addition of this information is important and may interest many readers.

***Authors' response: ****The information on the taxonomy of the organisms that contain the yjeK/yjeA pair is given in Additional file *1*and the analysis of this taxonomic distribution has also been added to the "Implications" section in the revised version of the manuscript*.

### Reviewer 1

D) Finally, it is important to show the genomes that harbour only *yjeA *or *yjeK*.

***Authors' response: ****Only two organisms Planctomyces limnophilus and Buchnera aphidicola str. APS (Additional file *1) *contained a yjeA and not a yjeK homolog. These are two symbiotic organisms known to shed genes and pathways. This information was added to the text*.

### Reviewer 1

To sum up, my main request concerns the inclusion of raw results in the paper (and not only their analysis). This may be easily done by the inclusion of a single supplementary table, showing for each bacterial genome analysed: (1) their taxonomic affiliation, (2) the presence of efP coding genes (with their accession numbers), (3) the conservation of lysine-34 in the corresponding proteins, (4) the presence of *yjeA *and *yjeK *genes (with their accession numbers) and (5) their physical clustering on the chromosome.

***Authors' response: ****The requested information covering points 1, 2, 4 and 5 are now included in the Additional file *1. *For point 3 we included a new *Fig 1B* and *Fig 1C* giving the distribution of Lys residues in organisms that have yjeK/yjeA and in organisms that do not and added the list of sequences used for this analysis as Additional files *3 and 4.

### Reviewer 1

Other remarks:

1) Precise if the two lysines that are modified in the bacterial efP and in the eukaryotic/archaeal eIF5A are homologous or analogous. Please use one of these two terms instead of "equivalent" (p.3 line 1). If they are homologous, it would be nice to show an alignment of a subsets efP and eIF5A as supplementary material.

***Authors' response: ****The two proteins are homologous as stated in the background section EF-P "is the bacterial homologue of the conserved eukaryotic/archaeal translation initiation factor 5A (eIF5A) *[2]". *The referenced paper by Kyrpedes and Woese had discovered this homology in 1998. We replaced the p. 3 equivalent by homologous in the revised version*.

### Reviewer 1

2) P.4 the authors say that clustalW2 were used to construct the phylogenetic tree showed as Fig. 2A. ClustalW is an alignment software that provides guide trees (but these are not phylogenetic trees). Thus, I suggest to the authors to re-compute their phylogenetic tree using typical tree reconstruction software (e.g. PhyML, MrBayes, Phylip, etc). Moreover, the authors should provide the methods and the parameters used for the phylogenetic reconstruction.

***Authors' response: ****We redid the phylogenetic tree using the Phylip software and the methods and parameters were added to the text*.

### Reviewer 1

3) P.4, it would be interesting to provide an alignment of primary sequences of LysRS2 and YjeA as supplementary material.

***Authors' response: ****This alignment was included as Additional file *2.

### Reviewer 1

4) P.6 In the sentence "First Ef-P is present in the cell in a lower ratio than the elongation factors... do the authors mean that Ef-P is present in the cell in a lower ratio than ribosomes?

***Authors' response: ****Yes all initiation factors are used only once per protein synthesis cycle and can be recycled*.

### Reviewer 2

Igor B. Zhulin, University of Tennessee - Oak Ridge National Laboratory, Oak Ridge, TN, USA

Synopsis:

This paper identifies two proteins that may be involved in post-translational modification of the bacterial EF-P translation elongation factor. The two proposed candidate proteins, YjeA and YjeK, were identified by genome neighborhood analysis that showed the genes coding for these proteins were adjacent to the *efp *gene (encoding EF-P) on 125 of 722 bacterial genomes. All genomes with the conserved motif for lysine modification of EF-P encoded homologs of these proteins. The YjeK homologs share common enzymatic domains with a protein involved in lysine catabolism, lysine aminomutase (LAM). The YjeK homologs are shown by phylogenetic analysis to form a distinct clade from the LAM proteins. YjeA is a homolog of lysine t-RNA synthase and the authors demonstrate a high degree of overlap between the 3D structures of YjeA from *S. typhimurium *and class II lysyl-tRNA synthetase (LysRS) from *E. coli*. Based on the chemistries of LAM and LysRS2, the authors propose how YjeA and YjeK could carry out the observed lysine modification of EF-P and discuss the implications of this hypothesis.

In general, this is an interesting paper and it offers a testable hypothesis, which can lead to better understanding the mechanisms of post-translational modification in bacteria.

I am not sure about the terms "bacteria" and "bacterial" that are used throughout this paper. I recall they were used to contrast "archaebacteria" that we now call "archaea". Thus, simply "bacteria" and "bacterial" will do.

***Authors' response: ****Eubacterial and eubacteria was replaced by bacterial and bacteria in the revised version*.

### Reviewer 2

Analysis:

Chromosomal clustering of *efp *and *yjeA*/*K*

The presentation of yjeA and yjeK can be improved. In the current version of the paper these genes first come from nowhere (***Genome organization and phyletic distribution of  *efp, yjeA *and *yjeK**: the second sentence). It would be helpful to mention that in order to find potential members of the pathway, investigators decided to look into the genomic neighborhoods of the *efp *gene. There, they have found *yjeA *and *yjeK *on many occasions, indicating a likely link. The initial chromosomal clustering study seems solid, although "clustering" as used is not a perfect term. "Clustering" can be a part of a very different type of sequence analysis (grouping sequences by similarity). "Chromosomal clustering", "chromosomal proximity", "genome context", "genomic neighborhood" seem to be better terms. Well, after all, there is a reference to "physical clustering" in the Fig. 1 legend. Still, there is a need for consistency in terminology.

***Authors' response: ****As suggested clustering was replaced by physical clustering when adequate throughout the revised manuscript*.

### Reviewer 2

Of the 125 genomes with *yjeK *and *yjeA *genes, 94% are chromosomally clustered with *efp*. Additional evidence comes from the fact that all EF-P proteins with the conserved lysine motif also contained homologs of YjeA and YjeK. Unfortunately, this critical piece of supporting data is not shown in any way. No mention is made of how many genomes contain only one of these two proteins (in which case one or the other enzymes could have been replaced by another function). I strongly recommend producing a supplementary table that would show the results. Column subheadings for such a table should include: 1) Species name; 2) Genome accession number; 3) efp ID (locus, GI or accession number. Locus tags would be the best because they will show proximity of neighboring genes); 4) YjeA (the same information or blank if absent); 5) yjeK (the same information or blank if absent).

***Authors' response: ****This information was included in the revised version as Additional file *1.

### Reviewer 2

YjeK/LAM homology:

YjeK and LAM were previously reported as homologs. The multiple sequence alignment (MSA) and phylogenetic analysis of these homologs seem a bit simplistic, carried out only using Clustal W. I am not familiar with the improvements made in Clustal W2, but the original Clustal W is certainly not the best program for MSA construction. No examples of the alignment are shown, so it is impossible to judge how well the Clustal W2 output looks like.

There is a similar problem with a tree shown in Fig. 2. Which method was used for its construction? I presume it is NJ, but this should be explicitly stated.

The results indicate that LAM and YjeK form two distinct clades (Fig. 2). However, LAM contains an additional domain not found in YjeK. From the figure legend, it appears that the full length proteins were used for the alignment (rather than just the conserved domain). If so, this will obviously strongly favor creation of a separate clade. The genome context analysis does support the idea of separate functional groups for LAM and YjeK proteins. This whole issue must be better clarified/supported.

***Authors' response: ****we thank the reviewer for pointing this to us as we had done the mistake of doing the alignment with the whole protein and not only the common domain. We redid the alignment using 118 amino acid sequence present in the N terminus active site and conserved between the YjeK and LAM subfamilies. Then we ran a phylogenetic analysis using the Phylip package *[25]. *using neighbour-joining method, with 1000 bootstrap and this analysis shown clear separations with high bootsrap values between the two clades. This analysis also helped us better separate YjeK/LAM paralogs in several genomes that we had not been able to really separate in our previous work that is why we have now 200 genomes that have both yjeA/yjeK s instead of 125 in the previous analysis*.

### Reviewer 2

YjeA/LysRS2 similarity:

The structural overlay of YjeA and LysRS2 clearly shows the structural similarity. The very close overlap is suggestive of similar function.

Potential mechanisms:

This section is plausible and can provide a starting point for experiments, but is clearly highly speculative.

Testing the hypothesis:

This section is weak. The authors point out a scheme to make an affinity tagged version of the protein and substitute the normal chromosomal copy in an *Acinetobacter *species. This is a very standard technique that could be summarized in a phrase instead of a paragraph. No reason is listed for carrying out these experiments in *Acinetobacter *instead of *E. coli *or another model organism (although the referenced paper claims that *Acinetobacter *is now a good model). The section does clarify the problems of dealing with a low copy essential gene, but no ideas are presented to overcome these difficulties.

While the paper spends almost one page of the manuscript on the detailed mechanistic hypothesis, no mention is made of experiments that could be used to differentiate these mechanisms from other possible mechanisms. Likewise, no experiments are proposed to more carefully examine the proposed phylogenetic relationships of YjeK and LAM.

To the authors' defence, I do not think this part of the paper should even be there. In this, I disagree with the requirements of Biology Direct. Experimenters, not computational scientists should think about testing a hypothesis, because the latter are not quite familiar with modern experimental approaches and techniques (especially the newer ones).

***Authors' response: ****a section was added on the *in vitro *testing of the hypothesis in the revised version*.

### Reviewer 2

Implications:

This section seems to offer a reasonable interpretation of the possible implications - possibly identifying a new post-translational modification mechanism while acknowledging that this is not a ubiquitous mechanism used by all prokaryotes.

### Reviewer 3

Mikhail Gelfand, Research and Training Center on Bioinformatics, Moscow, Russia

Overall this is a nice paper describing an interesting hypothesis. I feel, however, that the authors have nit exhausted the possibilities of comparative genomic analysis, nor presented their results in the best possible form.

The main problem with the hypothesis is that the suggested candidates are not universal. The abstract says: "In the other organisms, EF-P may be modified by another pathway or the translation machinery must have adapted to the lack of EF-P modification", but this means nothing. At that, how strictly conserved is the modification pathways in eukaryotes? How strictly conserved is the lysine: are there any examples when the corresponding position is occupied by another residue?

***Authors' response: ****The eIF5A modification pathway is strictly conserved in eukaryotes. The predicted modification pathway is clearly not conserved in prokaryotes. We changed the Implication section to reflect this and also included the data on the distribution of residues other than Lys in bacterial EF-P in *Fig. 1C.

### Reviewer 3

**Figure 5:**
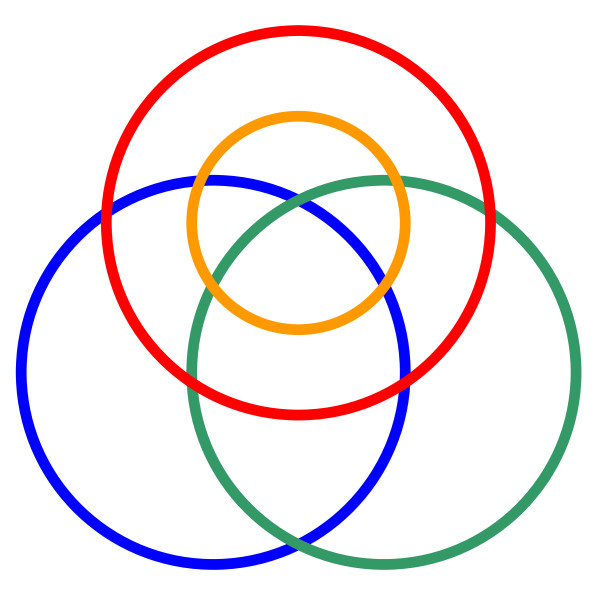
**Wenn diagram**. Red representing bacteria with *efp*; orange, a subset of those with the conserved. lysine; blue, bacteria with *yjeA*; and green, bacteria with *yjeK *(the colors, of course, are arbitrary, whereas the topology is not).

Since only 125 of 722 studied bacterial species have both candidate genes, an obvious question is, whether these genes always co-occur (if they are often found solo, this would be suspicious), whether they occur in species without the conserved lysine (if they do, what are they doing there?), and how many of 597 = 722-125 genomes have this lysine. Such analysis would allow the authors to verify the conclusions obtained by positional clustering using the phylogenetic profiles. The answer is best represented by a Wenn diagram (Fig. 5).

In fact, the authors write: "all organisms that contain EF-P proteins with a lysine residue at position 34 as part of the conserved "PGKG" motif contain homologs of YjeA and YjeK", but this crucial bit of their case is largely ignored: "(data not shown)".

***Authors' response:****as discussed in response to reviewer 1 and 2 this information was added not as Wenn diagram but in the text, as Additional file *1* and as *Fig 1B*and *1C.

### Reviewer 3

Not being a specialist in mass-spec, I cannot judge the validity of the the sentence: "Both reactions scheme would generate an EF-P modified on Lys34 by a β-lysine (146 Da) compatible with the 144 Da modification", - is 144 = 146 within the accepted error margins?

***Authors' response: ****The data describing the presence of the modification in E. coli EF-P in *Aoki H et al publication (*Febs J *1998, **275:**671-681)*is quite crude based only on predicted mass of tryptic peptide, so we feel we are in the accepted error margins*.

### Reviewer 3

Fig. 4 is rather difficult to comprehend. The notation and the distribution of proteins and substrates as summands at the top, or additional summands in intermediate stages, or catalyzing enzymes is unclear. I feel a more traditional form of presentation would be more useful here. Finally, for some reason the right panel is a mirror reflection of the left one: it may create a nice-looking symmetry, but for a less informed reader may create a minor, but annoying problem.

***Authors' response: ****we simplified the figure but kept the mirror image organization as we felt that was not really a problem*.

### Reviewer 3

A technical comment: I cannot deduce the meaning of parentheses "(red circle Fig. 3B zoom merge)" and "(Fig. 3B active site zoom merge)" on page 5.

***Authors' response: ****We clarified references to *Fig. 3* in the text in the revised manuscript*.

## Supplementary Material

Additional file 1 **Table S1**. Distribution and clustering of *efp*, *yjeK*, *yjeA*, *ablB *and *ablA *in the 725 organisms analyzed. Click here for file

Additional file 2 **Fig. S4**. Multiple Alignment of YjeA and LysRS2 sequences. Click here for file

Additional file 3 **Text S2**. List of EF-P sequences from organisms that have *yjeA/yjeK*. Click here for file

Additional file 4 **Text S3**. List of EF-P sequences from organisms that do not have have *yjeA/yjeK*. Click here for file

## References

[B1] Ganoza MC Kiel MC Aoki H Evolutionary conservation of reactions in translation Microbiol Mol Biol Rev 2002 66 460 485 10.1128/MMBR.66.3.460-485.2002 12209000 PMC120792

[B2] Kyrpides NC Woese CR Universally conserved translation initiation factors Proc Natl Acad Sci USA 1998 95 224 228 10.1073/pnas.95.1.224 9419357 PMC18182

[B3] An G Glick BR Friesen JD Ganoza MC Identification and quantitation of elongation factor EF-P in *Escherichia coli *cell-free extracts Can J Biochem 1980 58 1312 1314 7011506 10.1139/o80-177

[B4] Cole JR Olsson CL Hershey JW Grunberg-Manago M Nomura M Feedback regulation of rRNA synthesis in *Escherichia coli*. Requirement for initiation factor IF2 J Mol Biol 1987 198 383 392 10.1016/0022-2836(87)90288-9 2448483

[B5] Aoki H Dekany K Adams SL Ganoza MC The gene encoding the elongation factor P protein is essential for viability and is required for protein synthesis J Biol Chem 1997 272 32254 32259 10.1074/jbc.272.51.32254 9405429

[B6] Shimizu Y Inoue A Tomari Y Suzuki T Yokogawa T Nishikawa K Ueda T Cell-free translation reconstituted with purified components Nat Biotechnol 2001 19 751 755 10.1038/90802 11479568

[B7] Saini P Eyler DE Green R Dever TE Hypusine-containing protein eIF5A promotes translation elongation Nature 2009 459 118 121 10.1038/nature08034 19424157 PMC3140696

[B8] Cooper HL Park MH Folk JE Safer B Braverman R Identification of the hypusine-containing protein hy+ as translation initiation factor eIF-4D Proc Natl Acad Sci USA 1983 80 1854 1857 10.1073/pnas.80.7.1854 6403941 PMC393708

[B9] Byers TL Ganem B Pegg AE Cytostasis induced in L1210 murine leukaemia cells by the S-adenosyl-L-methionine decarboxylase inhibitor 5'-([(Z)-4-amino-2-butenyl]methylamino)-5'-deoxyadenosine may be due to hypusine depletion Biochem J 1992 287 Pt 3 717 724 1445235 10.1042/bj2870717 PMC1133067

[B10] Chattopadhyay MK Tabor CW Tabor H Spermidine but not spermine is essential for hypusine biosynthesis and growth in *Saccharomyces cerevisiae *: spermine is converted to spermidine in vivo by the FMS1-amine oxidase Proc Natl Acad Sci USA 2003 100 13869 13874 10.1073/pnas.1835918100 14617780 PMC283513

[B11] Chen KY Liu AY Biochemistry and function of hypusine formation on eukaryotic initiation factor 5A Biol Signals 1997 6 105 109 10.1159/000109115 9285092

[B12] Gerner EW Mamont PS Bernhardt A Siat M Post-translational modification of the protein-synthesis initiation factor eIF-4D by spermidine in rat hepatoma cells Biochem J 1986 239 379 386 3101665 10.1042/bj2390379 PMC1147291

[B13] Park MH The post-translational synthesis of a polyamine-derived amino acid, hypusine, in the eukaryotic translation initiation factor 5A (eIF5A) J Biochem 2006 139 161 169 10.1093/jb/mvj034 16452303 PMC2494880

[B14] Park MH Joe YA Kang KR Deoxyhypusine synthase activity is essential for cell viability in the yeast *Saccharomyces cerevisiae* J Biol Chem 1998 273 1677 1683 10.1074/jbc.273.3.1677 9430712

[B15] Murphey RJ Gerner EW Hypusine formation in protein by a two-step process in cell lysates J Biol Chem 1987 262 15033 15036 3117792

[B16] Wolff EC Lee YB Chung SI Folk JE Park MH Deoxyhypusine synthase from rat testis: purification and characterization J Biol Chem 1995 270 8660 8666 10.1074/jbc.270.15.8660 7721768

[B17] Abbruzzese A Park MH Folk JE Deoxyhypusine hydroxylase from rat testis. Partial purification and characterization J Biol Chem 1986 261 3085 3089 3949761

[B18] Aoki H Xu J Emili A Chosay JG Golshani A Ganoza MC Interactions of elongation factor EF-P with the *Escherichia coli *ribosome Febs J 2008 275 671 681 10.1111/j.1742-4658.2007.06228.x 18201202

[B19] Overbeek R Begley T Butler RM Choudhuri JV Chuang HY Cohoon M de Crécy-Lagard V Diaz N Disz T Edwards R Fonstein M Frank ED Gerdes S Glass EM Goesmann A Hanson A Iwata-Reuyl D Jensen R Jamshidi N Krause L Kubal M Larsen N Linke B McHardy AC Meyer F Neuweger H Olsen G Olson R Osterman A Portnoy V Pusch GD Rodionov DA Rückert C Steiner J Stevens R Thiele I Vassieva O Ye Y Zagnitko O Vonstein V The subsystems approach to genome annotation and its use in the project to annotate 1000 genomes Nucleic Acids Res 2005 33 5691 5702 10.1093/nar/gki866 16214803 PMC1251668

[B20] Moran NA McLaughlin HJ Sorek R The dynamics and time scale of ongoing genomic erosion in symbiotic bacteria Science 2009 323 379 382 10.1126/science.1167140 19150844

[B21] Brochier C Lopez-Garcia P Moreira D Horizontal gene transfer and archaeal origin of deoxyhypusine synthase homologous genes in bacteria Gene 2004 330 169 176 10.1016/j.gene.2004.01.018 15087136

[B22] Behshad E Ruzicka FJ Mansoorabadi SO Chen D Reed GH Frey PA Enantiomeric free radicals and enzymatic control of stereochemistry in a radical mechanism: the case of lysine 2,3-aminomutases Biochemistry 2006 45 12639 12646 10.1021/bi061328t 17042480 PMC2553251

[B23] Larkin MA Blackshields G Brown NP Chenna R McGettigan PA McWilliam H Valentin F Wallace IM Wilm A Lopez R Thompson JD Gibson TJ Higgins DG Clustal W and Clustal X version 2.0 Bioinformatics 2007 23 2947 2948 10.1093/bioinformatics/btm404 17846036

[B24] Lepore BW Ruzicka FJ Frey PA Ringe D The x-ray crystal structure of lysine-2,3-aminomutase from *Clostridium subterminale* Proc Natl Acad Sci USA 2005 102 13819 13824 10.1073/pnas.0505726102 16166264 PMC1236562

[B25] PHYLIP (Phylogeny Inference Package) version 3.68 http://evolution.genetics.washington.edu/phylip.html

[B26] Onesti S Desogus G Brevet A Chen J Plateau P Blanquet S Brick P Structural studies of lysyl-tRNA synthetase: conformational changes induced by substrate binding Biochemistry 2000 39 12853 12861 10.1021/bi001487r 11041850

[B27] Kaniga K Compton MS Curtiss R Sundaram P Molecular and functional characterization of *Salmonella enterica serovar typhimurium poxA *gene: effect on attenuation of virulence and protection Infect Immun 1998 66 5599 5606 9826331 10.1128/iai.66.12.5599-5606.1998 PMC108707

[B28] Van Dyk TK Smulski DR Chang YY Pleiotropic effects of *poxA *regulatory mutations of *Escherichia coli *and *Salmonella typhimurium*, mutations conferring sulfometuron methyl and alpha-ketobutyrate hypersensitivity J Bacteriol 1987 169 4540 4546 2820932 10.1128/jb.169.10.4540-4546.1987 PMC213819

[B29] Blaise M Becker HD Keith G Cambillau C Lapointe J Giegé R Kern D A minimalist glutamyl-tRNA synthetase dedicated to aminoacylation of the tRNAAsp QUC anticodon Nucleic Acids Res 2004 32 2768 2775 10.1093/nar/gkh608 15150343 PMC419609

[B30] Blaise M Becker HD Lapointe J Cambillau C Giegé R Kern D Glu-Q-tRNA(Asp) synthetase coded by the *yadB *gene, a new paralog of aminoacyl-tRNA synthetase that glutamylates tRNA(Asp) anticodon Biochimie 2005 87 847 861 10.1016/j.biochi.2005.03.007 16164993

[B31] Blaise M Olieric V Sauter C Lorber B Roy B Karmakar S Banerjee R Becker HD Kern D Crystal structure of glutamyl-queuosine tRNAAsp synthetase complexed with L-glutamate: structural elements mediating tRNA-independent activation of glutamate and glutamylation of tRNAAsp anticodon J Mol Biol 2008 381 1224 1237 10.1016/j.jmb.2008.06.053 18602926

[B32] Dubois DY Blaise M Becker HD Campanacci V Keith G Giegé R Cambillau C Lapointe J Kern D An aminoacyl-tRNA synthetase-like protein encoded by the *Escherichia coli yadB *gene glutamylates specifically tRNAAsp Proc Natl Acad Sci USA 2004 101 7530 7535 10.1073/pnas.0401634101 15096594 PMC419640

[B33] Roy H Becker HD Reinbolt J Kern D When contemporary aminoacyl-tRNA synthetases invent their cognate amino acid metabolism Proc Natl Acad Sci USA 2003 100 9837 9842 10.1073/pnas.1632156100 12874385 PMC187858

[B34] Lorber B Kern D Giegé R Ebel JP Covalent attachment of aspartic acid to *yeast *aspartyl-tRNA synthetase induced by the enzyme FEBS Lett 1982 146 59 64 10.1016/0014-5793(82)80705-9 6754443

[B35] Ling J Reynolds N Ibba M Aminoacyl-tRNA synthesis and translational quality control Annu Rev Microbiol 2009 63 61 78 10.1146/annurev.micro.091208.073210 19379069

[B36] Metzgar D Bacher JM Pezo V Reader J Döring V Schimmel P Marlière P de Crécy-Lagard V *Acinetobacter sp. ADP1 *: an ideal model organism for genetic analysis and genome engineering Nucleic Acids Res 2004 32 5780 5790 10.1093/nar/gkh881 15514111 PMC528786

[B37] Glick BR Green RM Ganoza MC Purification of *Escherichia coli *Elongation Factor P Can J Biochem 1979 57 749 757 383236

[B38] Blaha G Stanley RE Steitz TA Formation of the first peptide bond: the structure of EF-P bound to the 70S ribosome Science 2009 325 966 970 10.1126/science.1175800 19696344 PMC3296453

[B39] Crooks GE Hon G Chandonia JM Brenner SE WebLogo: a sequence logo generator Genome Res 2004 14 1188 1190 10.1101/gr.849004 15173120 PMC419797

[B40] WebLogo version 2.8.2 http://weblogo.berkeley.edu/logo.cgi

[B41] Arnold K Bordoli L Kopp J Schwede T The SWISS-MODEL workspace: a web-based environment for protein structure homology modelling Bioinformatics 2006 22 195 201 10.1093/bioinformatics/bti770 16301204

[B42] Kiefer F Arnold K Kunzli M Bordoli L Schwede T The SWISS-MODEL Repository and associated resources Nucleic Acids Res 2009 37 D387 392 10.1093/nar/gkn750 18931379 PMC2686475

[B43] Peitsch MC Wells TN Stampf DR Sussman JL The Swiss-3DImage collection and PDB-Browser on the World-Wide Web Trends Biochem Sci 1995 20 82 84 10.1016/S0968-0004(00)88963-X 7701568

