# Supplementary material for: Predicting the pathway involved in post-translational modification of Elongation factor P in a subset of bacterial species
Source: Biol Direct. 2010 Jan 13;5:3. doi: 10.1186/1745-6150-5-3 (PMC2821294; doi:10.1186/1745-6150-5-3)
Supplement: Additional file 2 — Fig. S4. Multiple Alignment of YjeA and LysRS2 sequences. [file 1745-6150-5-3-S2.PDF]

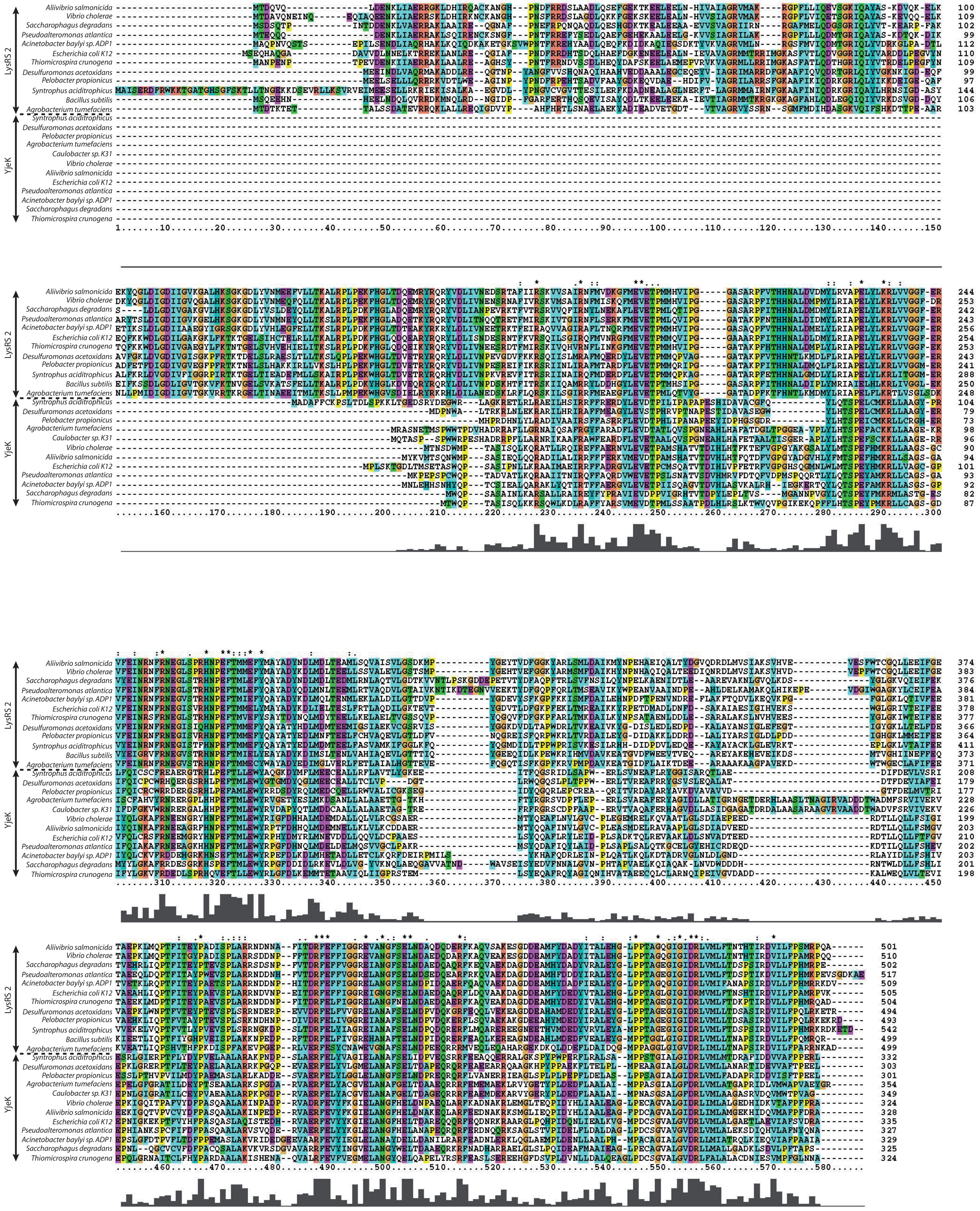

Bailly et *al.*, supp Figure 1

Fig. S4: Alignment of YjeA and LysRS 2.

The alignment was generated by comparing 12 sequences of LysRS 2 to 12 YjeA proteins from a subset of phylogenetically distant organisms. The alignment was generated in Clustal W2 [23], by using the default parameters. The alignment clearly shows the lack of the N-terminal anticodon binding domain (ABD) in the YjeA enzymes. YjeA and LysRS2 proteins are annotated.
