# Supplementary material for: Predicting the pathway involved in post-translational modification of Elongation factor P in a subset of bacterial species
Source: Biol Direct. 2010 Jan 13;5:3. doi: 10.1186/1745-6150-5-3 (PMC2821294; doi:10.1186/1745-6150-5-3)
Supplement: Additional file 3 — Text S2. List of EF-P sequences from organisms that have yjeA/yjeK. [file 1745-6150-5-3-S3.DOC]

**Supplemental text S2 List of EF-P sequences from organisms that have *yjeA/yjeK***

>fig|224324.1.peg.955 [Aquifex aeolicus VF5] [Translation elongation factor P @ Translation initiation factor 5A]

MATEIDINRIQKDIFIEHKGEPYRVLDYEHVKPGKGQAFVRVKAKNMLTG

NVTELTFKASDRIPLADFEQVYATYSYNDGENYYFMNTQTYDMIAVPKEK

IEEEAKFLKEGMEVIVFLYKGQPIGIELPKHVELQVVETEPAFKGDTQAG

GTKPAKLETGAVIQVPFFVKEGDIVKVDTRTGSYVERVKEAK

>fig|264201.1.peg.1529 [Parachlamydia sp. UWE25] [Translation elongation factor P @ Translation initiation factor 5A]

MAQMSTSEIRGGVKVEVEAQPYTIISNEFVKPGKGQAFNRIKMKHLLTGR

VIERTFKSGEKLNLADVAEEEMRMLYKESDGVIFMDEKSFEQIKISLESI

GETVQWLLDDHVYEVIFYNGNAVNVEPPTFMEMVITDTSPGVRGDTASGR

VLKPAILESGAKVQVPIFVEQGEKIKVDTRTGEYVSRVSS

>fig|243164.3.peg.821 [Dehalococcoides ethenogenes 195] [Translation elongation factor P @ Translation initiation factor 5A]

MATGNELKKSCIIQQENGLFQVMDIQHVKMKHTALLRLKLKDIRDGHTME

QTFQSDEKFNLVNLEYRHMQFLYNDENVYHFMDDKTFEQIALNKSVLGDA

VNYLLENGSVRVMTFQEEAIGVELPASVNLKIAHTEPGFKGDTAATTTKP

ATLETGLVVQVPLFINNEDLIRVDTRSGQYLGKATN

>fig|243164.3.peg.1144 [Dehalococcoides ethenogenes 195] [Translation elongation factor P @ Translation initiation factor 5A]

MNIEEVSKNKKILLDEIPYNVDDVDFMKPGKGSAVYRIKFRNLMDGGILE

RTYHSGDKLKEADVATYDMQYLYHENGNYIFMNTDTFEQHYMTEDRMGSR

GVFLKDGMVIPVQLLGDAPIEITLPNFVELEIVETSMSTGKETVTAQNKP

AKLSNGMEIGVPTFIKAGDIIKVDTRTGAYVERVGNKK

>fig|216389.5.peg.779 [Dehalococcoides sp. BAV1] [Translation elongation factor P @ Translation initiation factor 5A]

MNIEEISKNKKIMLDDIPYNVDDVDFMKPGKGSAVYRIKFRNLMDGGILE

RTFHSGDKLKEADVSTYDMQYLYHENGNYIFMNTDTFEQHFMAEDRMGSR

GVFLKDGMVVPVQLLGDNPIEITLPNFIELEVMETSMSTGKETVTAQNKP

AKLSNGMEIGVPTFMKAGDVIRVDTRTGAYVERVGNKK

>fig|216389.5.peg.642 [Dehalococcoides sp. BAV1] [Translation elongation factor P @ Translation initiation factor 5A]

MATGNELKKSCIIQQENGLFQVMDIQHVKMKHTALLRLKLKDIRDGHTME

QTFQSDEKFNLVNLEYRHMQFLYNDENVYHFMDEKTFEQIALNKAILGDA

VNYLLENGSVRVMTFQEDAIGVELPASVNLRIAHTEPGFKGDTAATTTKP

ATLETGLVVQVPLFINTEDLIRVDTRSGQYLGKATT

>fig|255470.3.peg.1357 [Dehalococcoides sp. CBDB1] [Translation elongation factor P @ Translation initiation factor 5A]

MATGNELKKSCIIKQENGLFQVIDIQHVKMKHTALLRLKLKDIRDGHTME

QTFQSDEKFNLVNLEYRHMQFLYNDENVYHFMDEKTFEQIALNKAVLGDA

VNYLLENGSVRVMTFQEDAIGVELPASVNLRIAHTEPGFKGDTAATTTKP

ATLETGLVVQVPLFINTEDLIRVDTRSGQYLGKATT

>fig|255470.3.peg.331 [Dehalococcoides sp. CBDB1] [Translation elongation factor P @ Translation initiation factor 5A]

MNIEEISKNKKIMLDDIPYNVDDVDFMKPGKGSAVYRIKFRNLMDGGILE

RTFHSGDKLKEADVSTYDMQYLYHENGNYIFMNTDTFEQHFMAEDRMGSR

GVFLKDGMVVPVQLLGDNPIEITLPNFIELEVMETSMSTGKETVTAQNKP

AKLSNGMEIGVPTFMKAGDVIRVDTRTGAYVERVGNKK

>fig|314230.3.peg.5286 [Blastopirellula marina DSM 3645] [Translation elongation factor P @ Translation initiation factor 5A]

MARRSFLHVGRPPGFIAPGATLSLQRELKVQYGTSDFRKGLKVQIDGEPY

LMTECNFVKPGKGNALYKCRLRNLIRGSSLDRTYRGGETLESADVSETDC

QFLYAQGDKFVFMENTTFEQYEMTKDQIDDAWKYLKDGMQCMATLYNDFP

ITINPPIHVELEITYCEPGVRGDTATNVSKPATVETGAEILVPAFVNLGD

VIRIDTRTGEYVERVKK

>fig|243090.1.peg.631 [Pirellula sp. 1] [Translation elongation factor P @ Translation initiation factor 5A]

MIVEPTVATYNTSDFRKGLKVQIDGEPYLITEMNFVKPGKGNAMYKCKMK

NLIRGTTLDRTYKGGDSLEAADVETTTVQFLYRQGQDYVFMDGTTFEQYE

VPNEVAGDIWKYLKDGTECSMTLYNGAAIIVEPPQHVQLEVTECGPGTKG

DTATNVTKPAMVETGAEFNVPGFIKEGNIIKINTLNNEYVERVNN

>fig|366602.3.peg.1131 [Caulobacter sp. K31] [Translation elongation factor P @ Translation initiation factor 5A]

MKVAASSLRKGFVVDMDGKLYVVLNVENIHPGKGTPVTQLNMRRISDGVK

VSERYRTTETVERAFVDQRDHTFLYQDGEGYHFMNPESFDQLVASPEVIG

DLGAYLAEGMVVQLSTHNDLPIALELPRTVTLEIVETEPSVKGQTASSSY

KPAILSNGVRTMVPPYIAAGTRVIILTEDGSYQERAKD

>fig|190650.1.peg.718 [Caulobacter crescentus CB15] [Translation elongation factor P @ Translation initiation factor 5A]

MKVAASSLRKGSVVDMDGKLYVVLSAENIHPGKGTPVTQLNMRRISDGVK

VSERYRTTEQVERAFVDDRNHTFLYSDGDGYHFMNPESYDQLVATEDVIG

DAAPYLQEGMTVILSTHNDVPIAIDLPRTVVLEIVDTEPSVKGQTASSSY

KPAVLSNGVKTTVPPYITAGTKVVILTEDGSYVERAKD

>fig|224911.1.peg.4386 [Bradyrhizobium japonicum USDA 110] [Translation elongation factor P @ Translation initiation factor 5A]

MIEQDGKLYVVVSAENIHPGKGTPVSQIEMRRISDGVKISERYKTTDQVE

KATIEERNFTFLYEDGDGYHFMNPETYDQVQVSKDVVGDAAAYLQPDMTV

KLSTHDVNVVSLALPQRVTLEVVETEPVTKGQTASSSYKPAVLSNGIRTT

VPPHIAVGTRIVVMTEDGSYSERAKD

>fig|288000.5.peg.4002 [Bradyrhizobium sp. BTAi1] [Translation elongation factor P @ Translation initiation factor 5A]

MKVIASSIRKGNVIEQDGKLYVVVTAENIHPGKGTPVSQIEMRRISDGVK

ISERYKTTDQVEKVTIEERNYTYLYEDPDGFHFMNPETYDQVLVPKDVVG

SQAAYLQENMTVKLSMHDVVPVSIALPQRVTLEVVDTEPVTKGQTASSSY

KPAVLSNGVRTGVPPHITVGTRIVVMTEDGSYCERAKD

>fig|314253.3.peg.3247 [Nitrobacter sp. Nb-311A] [Translation elongation factor P @ Translation initiation factor 5A]

MRVIASSIRKGNVIEQDGKLYVVLTAENIHPGKGTPVSQIEMRRISDGVK

ISERYKTTDQVERATIEDHNFTFLYEDADGFHFMNAETYDQVQVPKDIVG

NAAPYLQENMVVKLSLHEMVPVAITLPQRVTLEVVETEPVTKGQTASSSY

KPAVLSNGVRTGVPPHITVGTRVVVMTEDGSYVERAKD

>fig|323097.3.peg.2423 [Nitrobacter hamburgensis X14] [Translation elongation factor P @ Translation initiation factor 5A]

MRVIASSIRKGNVIEQDGKLYVVLTAENIHPGKGTPVSQIEMRRISDGVK

ISERYKTTDQVERATIEDHNFTFLYEDADGFHFMNAENYDQVQVPKDVVG

NVAPYLQENMVVKLSLHEMVPVAITLPQRVTLEVVETEPVTKGQTASSSY

KPAMLSNGVRTGVPPHVAVGTRIVVMTEDGSYVERAKD

>fig|323098.3.peg.1203 [Nitrobacter winogradskyi Nb-255] [Translation elongation factor P @ Translation initiation factor 5A]

MRVIASSIRKGNVIEQDGKLYVVLTAENIHPGKGTPVSQIEMRRISDGVK

ISERYKTTDQVERATIEDHNFTFLYEDGDGFHFMNAETYDQVQVPKDIVG

SAAPYLQENMVVKLSLHDMVPVAITLPQRVTLEVVETEPVTKGQTASSSY

KPAVLSNGVRTGVPPHVAVGTRVVVMTEDGSYVERAKD

>fig|316055.14.peg.2906 [Rhodopseudomonas palustris BisA53] [Translation elongation factor P @ Translation initiation factor 5A]

MRVIASSIRKGNVIEQDGKLYVVLTAENIHPGKGTPVSQIEMRRISDGVK

ISERYKTTDQVEKATIEDSNFTFLYEDADGFHFMNPESFDQVQVPKEVVG

NAAPYLAENMSVKLSMHDTTPVAIQLPQRATLEVVDTEPVTKGQTASSSY

KPAMLSNGVRTAVPPHIGVGTRIVVMTEDGSYVERAKD

>fig|316058.9.peg.2946 [Rhodopseudomonas palustris HaA2] [Translation elongation factor P @ Translation initiation factor 5A]

MRVIASSIRKGNVLEQDGKLYVVLSAENIHPGKGTPVSQIEMRRISDGVK

ISERYKTTDQVEKATIEERNYSFLYEDGEGFHFMEPESFDQVQVTKDVVG

NSAPYLQEGMVVKLSMHDTVAVAITLPQRATLEVVETEPVTKGQTASSSY

KPAVLSNGVRTAVPPHVGVGTRIVVLTEDGSYVERAKD

>fig|316056.14.peg.2778 [Rhodopseudomonas palustris BisB18] [Translation elongation factor P @ Translation initiation factor 5A]

MLQAETASLATGHLCPDRSRARNLGNSPLKVIASSIRKGNVIEQDGKLYV

VLTAENIHPGKGTPVSQIEMRRISDGVKISERYKTTDQVEKATIEDHNFN

YLYEDPDGFHFMNTENFDQVQVPKDVVGNAAPYLQENMTVKLSLHGVVPV

AIQMPQRATLEVVDTEPVTKGQTASSSYKPAILSNGVRTLVPPHIGTGTR

IVIMTEDGSYVERAKD

>fig|316057.3.peg.3944 [Rhodopseudomonas palustris BisB5] [Translation elongation factor P @ Translation initiation factor 5A]

MRVIASSIRKGNVLEQDGKLYVVLSAENIHPGKGTPVSQIEMRRISDGVK

VSERYKTTDQVEKATIEERNYTFLYEDGEGFHFMEPESFDQVQVTKDVVG

NSAPYLAENMVVKLSMHDTTAVAITLPQRATLEVVETEPVTKGQTASSSY

KPAILSNGVRTAVPPHVGVGTRVVVLTEDGSYVERAKD

>fig|258594.1.peg.2498 [Rhodopseudomonas palustris CGA009] [Translation elongation factor P @ Translation initiation factor 5A]

MRVIASSIRKGNVLEQDGKLYVVLSAENIHPGKGTPVSQIEMRRISDGVK

ISERYKTTDQVEKVTIEERNYSFLYEDGEGFHFMEPESYDQVQVTKDVVG

SAAPYLQEGMVVKLSMHDTTAVAITLPQRATLEVVDTEPVTKGQTASSSY

KPAVLSNGVRTQVPPHIGTGTRIVVLTEDGSYVERAKD

>fig|419610.8.peg.2762 [Methylobacterium extorquens PA1] [Translation elongation factor P @ Translation initiation factor 5A]

MIASTLRKGNVVDKDGKLYVILTAENIHPGKGTPVTQLDMRRITDGVKIS

ERYRTTEQVERAFVEDRDHTFLYQDGEGYHFMNPESYEQIAVPADVVGDA

APYLQEGMTVTLSTHNGVPLTIELPQRMTFEIVETEPVTKGQTASSSYKP

ALLSNGVKTSVPPHVSTGTRVVIMTADGSYVERAKD

>fig|426117.3.peg.398 [Methylobacterium sp. 4-46] [Translation elongation factor P @ Translation initiation factor 5A]

MKVIASSLRKGNVVEKDGRLYVILSAENIHPGKGTPVTQLDMRRITDGVK

VSERYRTTEQVERAFVEDREHTFLYKDGEGSHFMNPESYEQVAVPDDVIG

DQAAYLQEGMAVMLSLHNGVPLAIELPQRVTLEIVETEPVTKGQTASSSY

KPAVLSNGVRTLVPPHITTGTRVVIMTADGSYVERAKD

>fig|266835.1.peg.6493 [Mesorhizobium loti MAFF303099] [Translation elongation factor P @ Translation initiation factor 5A]

MVKVIASSLRKGNVVDKDGKLYVILFAENIHPGKGTPVTQLDMRRIGDGV

KVSERYRTTEQVERAYVEEREHTFLYADGEGFHFMNPETYDQVAVSEAVV

GDAAPYLQEGMPVQVSQFNGIAISLVLPQRATFEVVETEPTTKGQTASSS

YKPAVLSNGVRTAVPPHIAPGTRVVVMTADGSYVERAKD

>fig|176299.3.peg.1622 [Agrobacterium tumefaciens str. C58] [Translation elongation factor P @ Translation initiation factor 5A]

MVKVIASSVRKGNVLDVDGKLYVVLTAQNFHPGKGTPVTQVDMRRIVDGT

KVSERWRTTEQVERAFVEDLNFQFLYEDGEGFHFMNPENYDQVVVDVETM

GDQKAYLQEGMTCVLSIHEGNPLAVELPRHVTLEIVETEPVVKGQTASSS

YKPAILSNGIRTMVPPHIDAGIRVVIATEDNSYVERAKN

>fig|216596.1.peg.4735 [Rhizobium leguminosarum bv. viciae 3841] [Translation elongation factor P @ Translation initiation factor 5A]

MVKVIASSVRKGNVLDVDGKLYVVLTAQNFHPGKGTPVTQVDMRRIVDGV

KVSERWRTTEQVERAFVEDVSFQFLYEDGEGFHFMNPSSYDQVVVDVDTM

GDDKAYLQEGMSCILSMHEGIALALQLPRHVTLEIMETEPVVKGQTASSS

YKPAILSNGVRAMVPPHINAGTRVVIATEDNSYVERAKD

>fig|266834.1.peg.1571 [Sinorhizobium meliloti 1021] [Translation elongation factor P @ Translation initiation factor 5A]

MVKVIASSVRKGNVLDVDGKLYVVLTAQNFHPGKGTPVTQVDMRRISDGV

KVSERYRTTEQVERAFVEDREHTFLYEDGEGFHFMNPETYDQLVMSSEDI

GDLKAYLQEGMAVMLSIHEGIAIAIDLPRHVTLEITETEPVVKGQTASSS

YKPAVLSNGVRTLVPPHIQAGTRVVIATEDGSYVERAKD

>fig|318586.4.peg.2577 [Paracoccus denitrificans PD1222] [Translation elongation factor P @ Translation initiation factor 5A]

MARINGNEIKPGFVLDHDGGLWAAVKVNHVKPGKGGAFAQVELKNLRDGR

KLNERFRSEDKVEQVHLETKDQQFLYESDGKLVFMDSETFEQTELDADLL

GERRPFLQDGMVAAVEYYGDEALSVRIPQKVICRVAETEPVLNGQTAAKS

FKPAILDNGLRIMIPPFVGADEDIVVNTELFEYSERA

>fig|349102.4.peg.1936 [Rhodobacter sphaeroides ATCC 17025] [Translation elongation factor P @ Translation initiation factor 5A]

MPKINGNEIKPGFILEHDGGLWAAVKVNHVKPGKGGAFAQVELKNLRDGR

KLNERFRSEDKVERTELENKDQQFLYESDGRLIFMDAETYEQVEIDAELL

GERRPFLQDGMVATVNYYGDEALNVTLPPKVRCRVVETEPVVKGQTAANS

YKPAILDNGMRIMVPPFIGADEEILVHTELMEYSERV

>fig|349102.4.peg.2306 [Rhodobacter sphaeroides ATCC 17025] [Translation elongation factor P; Translation initiation factor 5A]

MKVIASSLRKGNVVDIDGRLYVVLTAQNFHPGKGTPVTQVDMRRISDGTK

VSERWKTTEQVERATVDEREYDFLYEDGEGYHFMEPESYDQVVVSPDVVG

DGKVFLTEGMRVYLQTHNDVAISIEFPQKVVVEITETEPVVKGQTASSSY

KPAMCTNGLRVMVPPHISAGTRIVINTEDLSYVERAKD

>fig|272943.3.peg.2432 [Rhodobacter sphaeroides 2.4.1] [Translation elongation factor P @ Translation initiation factor 5A]

MKVIASSLRKGNVVDIDGRLYVVLTAQNFHPGKGTPVTQVDMRRISDGTK

VSERWKTTEQVERATVDEREYDFLYEDGEGYHFMEPVSYDQVVVSPDVVG

DGKVFLTEGMRVYLQTHNDVAISIEFPQKVTVEITETEPVVKGQTASSSY

KPAMCTNGLRVMVPPHISAGTRIVINTEDLSYVERAKD

>fig|272943.3.peg.1874 [Rhodobacter sphaeroides 2.4.1] [Translation elongation factor P @ Translation initiation factor 5A]

MPKINGNEIKPGFILEHDGGLWAAVKVNHVKPGKGGAFAQVELKNLRDGR

KLNERFRSEDKVERTELENKDQQFLYESDGRLIFMDAESFEQVEIDAELL

GERRPFLQDGMVATVNYFGDEPLNVTLPAKVRCRVVETEPVVKGQTAANS

YKPAILDNGMRIMVPPFIGPDEEILVHTEFMEYSERV

>fig|349101.4.peg.2139 [Rhodobacter sphaeroides ATCC 17029] [Translation elongation factor P @ Translation initiation factor 5A]

MKVIASSLRKGNVVDIDGRLYVVLTAQNFHPGKGTPVTQVDMRRISDGTK

VSERWKTTEQVERATVDEREYDFLYEDGEGYHFMEPVSYDQVVVSPDVVG

DGKVFLTEGMRVYLQTHNDVAISIEFPQKVTVEITETEPVVKGQTASSSY

KPAMCTNGLRVMVPPHISAGTRIVINTEDLSYVERAKD

>fig|349163.4.peg.2177 [Acidiphilium cryptum JF-5] [Translation elongation factor P @ Translation initiation factor 5A]

MKQQANLIRAGQVIEHDGRRWTVLKQQIITPGKGGAFIQVEMRDLKTGNK

TNERWRTADTVERLMTDNRDYTYSYTDGDNLVLMDGETFEQFLVPAELLG

DQAPFLQDNMAVIVDLVEGDPVGIHLPATVTLEIVEADPVVKGQTASSSY

KPAKLSNGVKVMVPPFIEAGERIVVRTEDSTYVERAKG

>fig|290633.1.peg.220 [Gluconobacter oxydans 621H] [Translation elongation factor P @ Translation initiation factor 5A]

MKQQANLIRAGQVIEHDGRRWTVLKQQIITPGKGGAFIQVEMRDLKTGNK

TNERWRTADSVERLVTEDKDYTYSYMDGDNVVLMDPETFEQLILAKDIFG

DQFAFLQDNMPLNVKLVEGDPVGVELPPHVTLEVTEADPVVKGQTASSSY

KPAMLSNGVKTLVPPFIEAGERIVVRTEDGSYVERAKD

>fig|391165.8.peg.1084 [Granulibacter bethesdensis CGDNIH1] [Translation elongation factor P @ Translation initiation factor 5A]

MKQQANLIRAGQVIEHDGRRWTVLKQQIITPGKGGAFIQVEMRDLKTGNK

TNERWRTADTVERLMTEEKDYTYSYTDGDNLVLMDPETFEQALIPAEILG

DAVAFLQDNMQVTVDLVEGDPVAIHLPAQVTLEIVEADPVVKGQTASSSY

KPAKLSNGVRVMVPPFIEAGERIVVRTEDSTYVERAK

>fig|431944.4.peg.1621 [Magnetospirillum gryphiswaldense MSR-1] [Translation elongation factor P @ Translation initiation factor 5A]

MKINANLIRVGNIIENNGKQWAVLKTQIVQPGKGGAFITVEMRDIRTGTK

TNERWRTADTVEKCNVEEKDCTFLFVDGDALTFMDQENFEQFNLTREVLG

DAAAFLQDGMQVAVDFIEGSPVSVSLPEKVTMTVVEADPVVKGQTASSSY

KPAKLENGLKILVPPFIAEGEKIIVNTGDCSYVERAK

>fig|342108.5.peg.1145 [Magnetospirillum magneticum AMB-1] [Translation elongation factor P @ Translation initiation factor 5A]

MKINANLIRPGNILEHNGRQYAVLKTQIVQPGKGGAFITVEMRDIRTGNK

TNERWRTADTIEKCNVEAKECTFLFKDDSNMTFMDSESFEQFTMPNDTLG

DTIGFLQDGMVVEVDFVEGSPVSITLPEKVVMKVVEADPVVKGQTASSSY

KPAKLENGMKILVPPFLEEGEVIIVNTTDCSYVERFKG

>fig|1085.1.peg.3294 [Rhodospirillum rubrum] [Translation elongation factor P @ Translation initiation factor 5A]

MKMQANQIRPGQVLEHQGKRWTVLKIQLIQPGKGGAFIAVEMRDVDSGNK

TNERWRTADTVERLEVREIDCQYLFKDDTGYTFMDKETFEQFSMAADALG

EQAGFLQESMEVTVDCIEGSPVSVNLPSQVILQVVEADAVVKGQTASSSY

KPGLLENGMKVMIPPFIEAGTRIVVSTVDCSYIERAK

>fig|314275.3.peg.177 [Alteromonas macleodii 'Deep ecotype'] [Translation elongation factor P @ Translation initiation factor 5A]

MANYSTNEFKGGLKIMLDGEPCNILENEYVKPGKGQAFNRVKIRKLISGK

VLEKTFRSGESVEGADVMDTELAYLYTDGEFYHFMNNDTFEQIAADEKAV

GENQKWLVENDVCTITLWNGSPITVTPPNFVELEITETDPGLKGDTAGTG

GKPATLSTGAVVRVPLFVQTGEVIRVDTRSGEYVSRVQK

>fig|351348.5.peg.3074 [Marinobacter hydrocarbonoclasticus aquaeolei VT8] [Translation elongation factor P @ Translation initiation factor 5A]

MASYSTNEFRSGLKVLLDGDPCIMLENEFVKPGKGQAFNRVKLRNLMTNR

VWERTFKSGESLEAADVVEQDMEYLYTDGEFWHFMLTDGSFEQYAADAKA

VGDTVKWLKEQDVYTVTLYNGAPLTVTPPNFVELEVVDTDPGMKGDTAQG

GSKPATLSTGAVVNVPLFITIGEVLKVDTRSGEYVNRVKSS

>fig|203122.12.peg.1080 [Saccharophagus degradans 2-40] [Translation elongation factor P @ Translation initiation factor 5A]

MATYSASDFRSGLKVMLDGDPCAIVENELVKPGKGQAFARVRLRNLKTGR

VWERTFKSGETLEGADVMDRDMEYLYTDGEFWHFMEPDSFEQYQADANAV

GDSAKWLREQDKVIVTLFNGSPLAITPPNHVELEIVETDPGLKGDTAQGG

TKPATLTTGAVVKVPLFISTGEVVRVDTRTGEYLGRASK

>fig|167879.3.peg.939 [Colwellia psychrerythraea 34H] [Translation elongation factor P @ Translation initiation factor 5A]

MANFSTNQFKAGLKIMLDGEPCNILENELVKPGKGQAFSRVKIRKLVSGK

VLEKTFKSGETVEGADVMEVELAYLYADGEFWHFMNNETFEQIGAEEKAL

GETVKWLVEGDICTITLWNGTPITVTAANFVEIDITETDPGLKGDTAGTG

GKPATLATGAVVRVPLFVQIGEKVRIDTRSGEYVSRATKAQ

>fig|314276.3.peg.1008 [Idiomarina baltica OS145] [Translation elongation factor P @ Translation initiation factor 5A]

MANYSTNEFKGGLKIMQDGEPCSIVENEMVKPGKGQAFNRVKIRKLISGK

VVEKTFKSGESVEGADVIELELAYLYNDGEFWHFMNNETFEQVAADAKAV

GEAEKWLVEQDVCTLTLWEGNPINVQPPNFVELEITETDPGLKGDTAGTG

GKPATLSTGAVVRVPLFVQIGEVIKVDTRSGEYVSRVQK

>fig|283942.3.peg.2263 [Idiomarina loihiensis L2TR] [Translation elongation factor P @ Translation initiation factor 5A]

MANYSTSEFKGGLKIMQDGEPCSIVENEMVKPGKGQAFNRVKIRKLISGK

VVEKTFKSGESVEGADVIELELSYLYNDGEFWHFMNNETFEQVPADEKAV

GEAEKWLVEQDVCTLTLWEGKPINVQPPNFVELEITETDPGLKGDTAGTG

GKPATLSTGAVVRVPLFVQIGEVIKVDTRSGEYVSRVQK

>fig|342610.3.peg.1800 [Pseudoalteromonas atlantica T6c] [Translation elongation factor P @ Translation initiation factor 5A]

MANISTNEFKGGVKIMLDGEPCNILENEYVKPGKGQAFNRVKLKKLISGK

TLEKTFKSGESFEAADVMDMELSYLYTDGEFWHFMNNESFEQIAADDKAV

GDSVKWLVENDSCTITLWNGTPIVVTPPNFVELEITETDPGLKGDTAGTG

GKPATLTTGAVVRVPLFVQTGEVVKVDTRSGEYASRAQK

>fig|326442.4.peg.457 [Pseudoalteromonas haloplanktis TAC125] [Translation elongation factor P @ Translation initiation factor 5A]

MANYSTNEFKGGLKIMMDGEPSSIIENEMVKPGKGQAFNRVRIRKLISGK

VLEKTFKSGESVEGADVMDTDLAYLYTDGEFWHFMNNETFEQIAADEKAL

GDAGKWLVENDVCTITLWNGNPIVVTPPNFVELEITETDPGLKGDTAGTG

GKPATLSTGAVVRVPLFVQIGEVIKVDTRNGEYVSRVK

>fig|314282.3.peg.1151 [Psychromonas sp. CNPT3] [Translation elongation factor P @ Translation initiation factor 5A]

MATYSTSEFKGGLKFMLDKEPCAIIDNELVKPGKGQAFNRVKFRKLLSGK

VVEKTFKSGETVEAADVMDIELVYLYNDGEFYHFMDNNTFEQVAADAKAV

GDMEKWLVEQNICTITLWNDNPIAVTPANFVEIEVTDTDPGLKGDTAGTG

GKPATLATGATIRVPLFIQIGEVVRVDTRSAEYVGRVKK

>fig|357804.5.peg.3098 [Psychromonas ingrahami ingrahamii 37] [Translation elongation factor P @ Translation initiation factor 5A]

MGTYNTSEFKGGLKFMLDNEPCAIIENEFVKPGKGQAFSRVKLRKLLSGK

VIEKTFKSGETVESADVMDIELSYLYSDDQFYHFMNNETFEQIAADVKAV

GDMAKWLVEQDICTITTWNDNPIVVTPPNFVELEVTDTDPGLKGDTAGTG

GKPATLATGALIRVPLFIQIGDMVKVDTRSGEYVSRVKK

>fig|323261.3.peg.2432 [Nitrosococcus oceani ATCC 19707] [Translation elongation factor P @ Translation initiation factor 5A]

MAGYSTSEFKSGLKVMMDGDPYTIMENEFVKPGKGQAFNRVKLRNLKTGR

VIDRTFKSGDSLEAADVIETQLQYLYSDGEFWHLMSPETYEQYSADAAAV

GDSTKWLKEQDTCTVTLWNGAPLLVSPPNFVTLKVTETDPGIRGDTSGGG

SKPATLETGAVVRVPLFIDVGEMLKIDTRTGEYVERAKE

>fig|187272.6.peg.656 [Alkalilimnicola ehrlichei MLHE-1] [Translation elongation factor P @ Translation initiation factor 5A]

MAQYSTNEFKGGLKIMLDGDPYTIVENEFVKPGKGQAFNRVKVRNLKTGK

VIDRTFKSGESVEAADVLETEMQFLYSDGEFFHMMDPETFEQKAAPASAV

GDAAKWLKEQAMCTVILWNDEPLTVEPPNFVELRVVETDPGVRGDTSGGG

GKPATLETGAVVRVPLFIEEGELLKVDTRSGEYVGRVKG

>fig|349124.5.peg.1948 [Halorhodospira halophila SL1] [Translation elongation factor P @ Translation initiation factor 5A]

MATYSTNEFKGGLKIMLDGDPFTIVENEFVKPGKGQAFNRVKLRNLKTNR

VVEKTFKSGESVEAADVMETELQYLYNDGEFWYFMDPTSFEQKAAPSSAV

GDAANWIKEQDTCTVILWNDTPLQVEAPNFVDLKVIETDPGVRGDTSSGG

TKPAKLETGATVRVPLFIEEGEVLRVDTRKAEYVSRAKD

>fig|314278.3.peg.3318 [Nitrococcus mobilis Nb-231] [Translation elongation factor P @ Translation initiation factor 5A]

MASYTTSQFKAGLKILLDGDPYAIVENEFVKPGKGQAFNRVKIRNLRSGR

VVERTFKSGDSVEAADVIETEMQYLYTDGEFWHFMKPDSFEQYAAGKTVI

GDGAKWLKEQDLCKVTLWNNEPLSIEPPAHVNLAVVETDPGLRGDTSGGG

GKPATLETGAVVQVPLFIEEGEVVRVDTRSGAYISRAKE

>fig|198804.1.peg.21 [Buchnera aphidicola str. Sg (Schizaphis graminum)] [Translation elongation factor P @ Translation initiation factor 5A]

MRVYHSNNFRSGRKIIFEKEPCLIESSEFVKPGKGQSFVRVKLRKLLTKQ

LIEKTFKSTDSLEIADVSEYTLSYLYNDGHFWYFINNNFEELSVDKKIVG

VNKKWLSEQDTCVITFWNNQAISITPNIFVELKVIDTEIALKSDTINTTT

KLATLSTGAILKVPLFIQIGSLIKVDTRSGEYVSRIK

>fig|224915.1.peg.22 [Buchnera aphidicola str. Bp (Baizongia pistaciae)] [Translation elongation factor P @ Translation initiation factor 5A]

MELCHSNQLKIGLKIIYNNEPCIVVSNEFIKPGKGQAFFRVRLKNLLNKK

LIDKTCKYSDCFKIANVIEITATYMFTDKKVWTFMDKKSFEQIFVEKNII

KNVLPWLLEQHDYIISLWNDQPISIVYSSNFIELIVVNTIPNARSGAINT

YSKLALLNTGVTIKVPIFIQIGQIIKVDTRTSEYISKIS

>fig|399742.4.peg.405 [Enterobacter sp. 638] [Translation elongation factor P @ Translation initiation factor 5A]

MATYYSNDFRAGLKIMMDGEPYAVEASEFVKPGKGQAFARVKLRRLLTGT

RVEKTFKSTDSAEGADVVDMNLTYLYNDGEFYHFMNNTTFEQLSADEKAV

GENAKWLLDQAECIVTLWNGQPISVTPPNFVELEIIETDPGLKGDTAGTG

GKPAKLSTGAVVKVPLFVQTGEVIKVDTRSGEYVSRVK

>fig|331112.3.peg.4104 [Escherichia coli HS] [Translation elongation factor P @ Translation initiation factor 5A]

MATYYSNDFRAGLKIMLDGEPYAVEASEFVKPGKGQAFARVKLRRLLTGT

RVEKTFKSTDSAEGADVVDMNLTYLYNDGEFWHFMNNETFEQLSADAKAI

GDNAKWLLDQAECIVTLWNGQPISVTPPNFVELEIVDTDPGLKGDTAGTG

GKPATLSTGAVVKVPLFVQIGEVIKVDTRSGEYVSRVK

>fig|340197.3.peg.3499 [Escherichia coli F11] [Translation elongation factor P @ Translation initiation factor 5A]

MATYYSNDFRAGLKIMLDGEPYAVEASEFVKPGKGQAFARVKLRRLLTGT

RVEKTFKSTDSAEGADVVDMNLTYLYNDGEFWHFMNNETFEQLSADAKAI

GDNAKWLLDQAECIVTLWNGQPISVTPPNFVELEIVDTDPGLKGDTAGTG

GKPATLSTGAVVKVPLFVQIGEVIKVDTRSGEYVSRVK

>fig|340184.3.peg.681 [Escherichia coli B7A] [Translation elongation factor P @ Translation initiation factor 5A]

MATYYSNDFRAGLKIMLDGEPYAVEASEFVKPGKGQAFARVKLRRLLTGT

RVEKTFKSTDSAEGADVVDMNLTYLYNDGEFWHFMNNETFEQLSADAKAI

GDNAKWLLDQAECIVTLWNGQPISVTPPNFVELEIVDTDPGLKGDTAGTG

GKPATLSTGAVVKVPLFVQIGEVIKVDTRSGEYVSRVK

>fig|344601.3.peg.3290 [Escherichia coli B171] [Translation elongation factor P @ Translation initiation factor 5A]

MATYYSNDFRAGLKIMLDGEPYAVEASEFVKPGKGQAFARVKLRRLLTGT

RVEKTFKSTDSAEGADVVDMNLTYLYNDGEFWHFMNNETFEQLSADAKAI

GDNAKWLLDQAECIVTLWNGQPISVTPPNFVELEIVDTDPGLKGDTAGTG

GKPATLSTGAVVKVPLFVQIGEVIKVDTRSGEYVSRVK

>fig|405955.9.peg.3986 [Escherichia coli APEC O1] [Translation elongation factor P @ Translation initiation factor 5A]

MATYYSNDFRAGLKIMLDGEPYAVEASEFVKPGKGQAFARVKLRRLLTGT

RVEKTFKSTDSAEGADVVDMNLTYLYNDGEFWHFMNNETFEQLSADAKAI

GDNAKWLLDQAECIVTLWNGQPISVTPPNFVELEIVDTDPGLKGDTAGTG

GKPATLSTGAVVKVPLFVQIGEVIKVDTRSGEYVSRVK

>fig|155864.1.peg.5088 [Escherichia coli O157:H7 EDL933] [Translation elongation factor P @ Translation initiation factor 5A]

MATYYSNDFRAGLKIMLDGEPYAVEASEFVKPGKGQAFARVKLRRLLTGT

RVEKTFKSTDSAEGADVVDMNLTYLYNDGEFWHFMNNETFZQLSADAKAI

GDNAKWLLDQAECIVTLWNGQPISVTPPNFVELEIVDTDPGLKGDTAGTG

GKPATLSTGAVVKVPLFVQIGEVIKVDTRSGEYVSRVK

>fig|340185.3.peg.4127 [Escherichia coli E22] [Translation elongation factor P @ Translation initiation factor 5A]

MATYYSNDFRAGLKIMLDGEPYAVEASEFVKPGKGQAFARVKLRRLLTGT

RVEKTFKSTDSAEGADVVDMNLTYLYNDGEFWHFMNNETFEQLSADAKAI

GDNAKWLLDQAECIVTLWNGQPISVTPPNFVELEIVDTDPGLKGDTAGTG

GKPATLSTGAVVKVPLFVQIGEVIKVDTRSGEYVSRVK

>fig|340186.3.peg.1075 [Escherichia coli E110019] [Translation elongation factor P @ Translation initiation factor 5A]

MATYYSNDFRAGLKIMLDGEPYAVEASEFVKPGKGQAFARVKLRRLLTGT

RVEKTFKSTDSAEGADVVDMNLTYLYNDGEFWHFMNNETFEQLSADAKAI

GDNAKWLLDQAECIVTLWNGQPISVTPPNFVELEIVDTDPGLKGDTAGTG

GKPATLSTGAVVKVPLFVQIGEVIKVDTRSGEYVSRVK

>fig|344610.3.peg.1428 [Escherichia coli 53638] [Translation elongation factor P @ Translation initiation factor 5A]

MATYYSNDFRAGLKIMLDGEPYAVEASEFVKPGKGQAFARVKLRRLLTGT

RVEKTFKSTDSAEGADVVDMNLTYLYNDGEFWHFMNNETFEQLSADAKAI

GDNAKWLLDQAECIVTLWNGQPISVTPPNFVELEIVDTDPGLKGDTAGTG

GKPATLSTGAVVKVPLFVQIGEVIKVDTRSGEYVSRVK

>fig|199310.1.peg.5123 [Escherichia coli CFT073] [Translation elongation factor P @ Translation initiation factor 5A]

MATYYSNDFRAGLKIMLDGEPYAVEASEFVKPGKGQAFARVKLRRLLTGT

RVEKTFKSTDSAEGADVVDMNLTYLYNDGEFWHFMNNETFEQLSADAKAI

GDNAKWLLDQAECIVTLWNGQPISVTPPNFVELEIVDTDPGLKGDTAGTG

GKPATLSTGAVVKVPLFVQIGEVIKVDTRSGEYVSRVK

>fig|331111.3.peg.2302 [Escherichia coli E24377A] [Translation elongation factor P @ Translation initiation factor 5A]

MATYYSNDFRAGLKIMLDGEPYAVEASEFVKPGKGQAFARVKLRRLLTGT

RVEKTFKSTDSAEGADVVDMNLTYLYNDGEFWHFMNNETFEQLSADAKAI

GDNAKWLLDQAECIVTLWNGQPISVTPPNFVELEIVDTDPGLKGDTAGTG

GKPATLSTGAVVKVPLFVQIGEVIKVDTRSGEYVSRVK

>fig|83334.1.peg.5106 [Escherichia coli O157:H7] [Translation elongation factor P @ Translation initiation factor 5A]

MATYYSNDFRAGLKIMLDGEPYAVEASEFVKPGKGQAFARVKLRRLLTGT

RVEKTFKSTDSAEGADVVDMNLTYLYNDGEFWHFMNNETFEQLSADAKAI

GDNAKWLLDQAECIVTLWNGQPISVTPPNFVELEIVDTDPGLKGDTAGTG

GKPATLSTGAVVKVPLFVQIGEVIKVDTRSGEYVSRVK

>fig|316407.3.peg.3984 [Escherichia coli W3110] [Translation elongation factor P @ Translation initiation factor 5A]

MATYYSNDFRAGLKIMLDGEPYAVEASEFVKPGKGQAFARVKLRRLLTGT

RVEKTFKSTDSAEGADVVDMNLTYLYNDGEFWHFMNNETFEQLSADAKAI

GDNAKWLLDQAECIVTLWNGQPISVTPPNFVELEIVDTDPGLKGDTAGTG

GKPATLSTGAVVKVPLFVQIGEVIKVDTRSGEYVSRVK

>fig|83333.1.peg.4059 [Escherichia coli K12] [Translation elongation factor P @ Translation initiation factor 5A]

MATYYSNDFRAGLKIMLDGEPYAVEASEFVKPGKGQAFARVKLRRLLTGT

RVEKTFKSTDSAEGADVVDMNLTYLYNDGEFWHFMNNETFEQLSADAKAI

GDNAKWLLDQAECIVTLWNGQPISVTPPNFVELEIVDTDPGLKGDTAGTG

GKPATLSTGAVVKVPLFVQIGEVIKVDTRSGEYVSRVK

>fig|216593.1.peg.3853 [Escherichia coli E2348/69] [Translation elongation factor P @ Translation initiation factor 5A]

MATYYSNDFRAGLKIMLDGEPYAVEASEFVKPGKGQAFARVKLRRLLTGT

RVEKTFKSTDSAEGADVVDMNLTYLYNDGEFWHFMNNETFEQLSADAKAI

GDNAKWLLDQAECIVTLWNGQPISVTPPNFVELEIVDTDPGLKGDTAGTG

GKPATLSTGAVVKVPLFVQIGEVIKVDTRSGEYVSRVK

>fig|216592.1.peg.2577 [Escherichia coli 042] [Translation elongation factor P @ Translation initiation factor 5A]

MATYYSNDFRAGLKIMLDGEPYAVEASEFVKPGKGQAFARVKLRRLLTGT

RVEKTFKSTDSAEGADVVDMNLTYLYNDGEFWHFMNNETFEQLSADAKAI

GDNAKWLLDQAECIVTLWNGQPISVTPPNFVELEIVDTDPGLKGDTAGTG

GKPATLSTGAVVKVPLFVQIGEVIKVDTRSGEYVSRVK

>fig|481805.3.peg.4148 [Escherichia coli ATCC 8739] [Translation elongation factor P @ Translation initiation factor 5A]

MATYYSNDFRAGLKIMLDGEPYAVEASEFVKPGKGQAFARVKLRRLLTGT

RVEKTFKSTDSAEGADVVDMNLTYLYNDGEFWHFMNNETFEQLSADAKAI

GDNAKWLLDQAECIVTLWNGQPISVTPPNFVELEIVDTDPGLKGDTAGTG

GKPATLSTGAVVKVPLFVQIGEVIKVDTRSGEYVSRVK

>fig|272620.3.peg.4562 [Klebsiella pneumoniae MGH 78578] [Translation elongation factor P @ Translation initiation factor 5A]

MATYYSNDFRAGLKIMLDGEPYAVEASEFVKPGKGQAFARVKLRRLLTGT

RVEKTFKSTDSAEGADVVDMNLTYLYNDGEFWHFMNNETFEQLSADAKAI

GDNAKWLLDQAECIVTLWNGQPIAVTPPNFVELEIIETDPGLKGDTAGTG

GKPATLSTGAVVKVPLFVQIGEVIKVDTRSGEYVSRVK

>fig|218491.3.peg.2690 [Erwinia carotovora subsp. atroseptica SCRI1043] [Translation elongation factor P @ Translation initiation factor 5A]

MATYSSNDFRSGLKIIFESEPYAIESSEFVKPGKGQAFARVKMRRLLTGS

RVEKTFKSTDSLEGADVVDTNMNYLYNDGEFYHFMHPETFEQHQVEEKTV

GDSAKWLQDNAECIVTLWDGRPITVLPPNFIEAEITDTDPGLKGDTAGTG

GKPATLSTGAVVKVPLFVQIGEVVRVDTRSGEYVSRVK

>fig|243265.1.peg.3943 [Photorhabdus luminescens subsp. laumondii TTO1] [Translation elongation factor P @ Translation initiation factor 5A]

MATYSTNEFRSGLKIMLDGEPCAILESEFVKPGKGQAFARVRIRKLISGK

LLEKTFKSTDSVESADVMDMNLTYLYNDGEFWHFMNNETFEQLAADEKAV

GDNAKWLVEQAECILTLWNGQPISVTPPNFVELEITDTDPGLKGDTAGTG

GKPATLNTGAVVKVPLFVQIGEVIKVDTRSGEYVSRVK

>fig|171440.1.peg.1152 [Photorhabdus asymbiotica subsp. asymbiotica] [Translation elongation factor P @ Translation initiation factor 5A]

MATYSTNEFRSGLKIMLDGEPCAILESEFVKPGKGQAFARVRIRKLISGK

LLEKTFKSTDSVESADVMDMNLTYLYNDGEFWHFMNNETFEQLAADEKAV

GENAKWLVEQAECILTLWNGQPISVTPPNFVELEITETDPGLKGDTAGTG

GKPATLSTGAVVKVPLFVQIGEVIKVDTRSGEYVSRVK

>fig|584.1.peg.2950 [Proteus mirabilis HI4320] [Translation elongation factor P @ Translation initiation factor 5A]

MASYNTNDFRSGLKIMLDGEPAVITECEFVKPGKGQAFARVRLRKLISNK

LLEKTFKSTDSAEGADVMDINLTYLYNDGEFWHFMNNETFEQLAADEKAV

GENAKWLIDQAECIVTLWDNRPIAVVPPNFVELEIVDTDPGLKGDTAGTG

GKPATLSTGAVVKVPLFVQIGEVIKVDTRSGEYVSRVK

>fig|220341.1.peg.4163 [Salmonella enterica subsp. enterica serovar Typhi str. CT18] [Translation elongation factor P @ Translation initiation factor 5A]

MATYYSNDFRSGLKIMLDGEPYAVESSEFVKPGKGQAFARVKLRRLLTGT

RVEKTFKSTDSAEGADVVDMNLTYLYNDGEFWHFMNNETFEQLSADAKAI

GDNAKWLLDQAECIVTLWNGQPISVTPPNFVELEIVDTDPGLKGDTAGTG

GKPATLSTGAVVKVPLFVQIGEVIKVDTRSGEYVSRVK

>fig|99287.1.peg.4171 [Salmonella typhimurium LT2] [Translation elongation factor P @ Translation initiation factor 5A]

MATYYSNDFRSGLKIMLDGEPYAVESSEFVKPGKGQAFARVKLRRLLTGT

RVEKTFKSTDSAEGADVVDMNLTYLYNDGEFWHFMNNETFEQLSADAKAI

GDNAKWLLDQAECIVTLWNGQPISVTPPNFVELEIVDTDPGLKGDTAGTG

GKPATLSTGAVVKVPLFVQIGEVIKVDTRSGEYVSRVK

>fig|209261.1.peg.4087 [Salmonella enterica subsp. enterica serovar Typhi Ty2] [Translation elongation factor P @ Translation initiation factor 5A]

MATYYSNDFRSGLKIMLDGEPYAVESSEFVKPGKGQAFARVKLRRLLTGT

RVEKTFKSTDSAEGADVVDMNLTYLYNDGEFWHFMNNETFEQLSADAKAI

GDNAKWLLDQAECIVTLWNGQPISVTPPNFVELEIVDTDPGLKGDTAGTG

GKPATLSTGAVVKVPLFVQIGEVIKVDTRSGEYVSRVK

>fig|295319.3.peg.3434 [Salmonella enterica subsp. enterica serovar Paratypi A str. ATCC 9150] [Translation elongation factor P @ Translation initiation factor 5A]

MATYYSNDFRSGLKIMLDGEPYAVESSEFVKPGKGQAFARVKLRRLLTGT

RVEKTFKSTDSAEGADVVDMNLTYLYNDGEFWHFMNNETFEQLSADAKAI

GDNAKWLLDQAECIVTLWNGQPISVTPPNFVELEIVDTDPGLKGDTAGTG

GKPATLSTGAVVKVPLFVQIGEVIKVDTRSGEYVSRVK

>fig|12149.1.peg.4306 [Salmonella bongori 12149] [Translation elongation factor P @ Translation initiation factor 5A]

MWLSYQLTISEGLMATYYSNDFRSGLKIMLDGEPYAVESSEFVKPGKGQA

FARVKLRRLLTGTRVEKTFKSTDSAEGADVVDMNLTYLYNDGEFWHFMNN

ETFEQLSADAKAIGDNAKWLLDQAECIVTLWNGQPISVTPPNFVELEIVD

TDPGLKGDTAGTGGKPATLSTGAVVKVPLFVQIGEVIKVDTRSGEYVSRV

K

>fig|321314.4.peg.4269 [Salmonella enterica subsp. enterica serovar Choleraesuis str. SC-B67] [Translation elongation factor P @ Translation initiation factor 5A]

MPFSMRAIKARGTRRANKRLASCPARRFSSASICKRCSSSSGLVTTSASC

ANQSSRNGVFRVTICAILWLSYQLTISEGLMATYYSNDFRSGLKIMLDGE

PYAVESSEFVKPGKGQAFARVKLRRLLTGTRVEKTFKSTDSAEGADVVDM

NLTYLYNDGEFWHFMNNETFEQLSADAKAIGDNAKWLLDQAECIVTLWNG

QPISVTPPNFVELEIVDTDPGLKGDTAGTGGKPATLSTGAVVKVPLFVQI

GEVIKVDTRSGEYVSRVK

>fig|594.1.peg.800 [Salmonella enterica subsp. enterica serovar Gallinarum] [Translation elongation factor P @ Translation initiation factor 5A]

MWLSYQLTISEGLMATYYSNDFRSGLKIMLDGEPYAVESSEFVKPGKGQA

FARVKLRRLLTGTRVEKTFKSTDSAEGADVVDMNLTYLYNDGEFWHFMNN

ETFEQLSADAKAIGDNAKWLLDQAECIVTLWNGQPISVTPPNFVELEIVD

TDPGLKGDTAGTGGKPATLSTGAVVKVPLFVQIGEVIKVDTRSGEYVSRV

K

>fig|399741.3.peg.418 [Serratia proteamaculans 568] [Translation elongation factor P @ Translation initiation factor 5A]

MATYSSNDFRPGLKIMFEGEPYAIESSEFVKPGKGQAFARVKMRRLLTGS

RVEKTFKSTDSCEGADVVDTNMNYLYNDGEFYHFMHPETFEQHGVEEKTV

SDAAKWLQDNAECIVTLWDGRPIAVQPPNFIEAEITDTDPGLKGDTAGTG

GKPATLSTGAVVKVPLFVQIGEVVRVDTRSGEYVSRVK

>fig|615.1.peg.3374 [Serratia marcescens Db11] [Translation elongation factor P @ Translation initiation factor 5A]

MATYSSNDFRPGLKIMFEGEPYAVEASEFVKPGKGQAFARVKMRRLLTGT

RVEKTFKSTDSCEGADVMDTNMNYLYSDGEFYHFMHPESFEQHQVDGKTV

GDAAKWLQDNAECIITLWDGRPIAVQPPNFIEAEITDTDPGLKGDTAGTG

GKPATLSTGAVVKVPLFVQIGEVIKVDTRSGEYVSRVK

>fig|300269.3.peg.3901 [Shigella sonnei Ss046] [Translation elongation factor P @ Translation initiation factor 5A]

MATYYSNDFRAGLKIMLDGEPYAVEASEFVKPGKGQAFARVKLRRLLTGT

RVEKTFKSTDSAEGADVVDMNLTYLYNDGEFWHFMNNETFEQLSADAKAI

GDNAKWLLDQAECIVTLWNGQPISVTPPNFVELEIVDTDPGLKGDTAGTG

GKPATLSTGAVVKVPLFVQIGEVIKVDTRSGEYVSRVK

>fig|216599.1.peg.3628 [Shigella sonnei 53G] [Translation elongation factor P @ Translation initiation factor 5A]

MATYYSNDFRAGLKIMLDGEPYAVEASEFVKPGKGQAFARVKLRRLLTGT

RVEKTFKSTDSAEGADVVDMNLTYLYNDGEFWHFMNNETFEQLSADAKAI

GDNAKWLLDQAECIVTLWNGQPISVTPPNFVELEIVDTDPGLKGDTAGTG

GKPATLSTGAVVKVPLFVQIGEVIKVDTRSGEYVSRVK

>fig|198214.1.peg.4054 [Shigella flexneri 2a str. 301] [Translation elongation factor P @ Translation initiation factor 5A]

MATYYSNDFRAGLKIMLDGEPYAVEASEFVKPGKGQAFARVKLRRLLTGT

RVEKTFKSTDSAEGADVVDMNLTYLYNDGEFWHFMNNETFEQLSADAKAI

GDNAKWLLDQAECIVTLWNGQPISVTPPNFVELEIVDTDPGLKGDTAGTG

GKPATLSTGAVVKVPLFVQIGEVIKVDTRSGEYVSRVK

>fig|344609.3.peg.650 [Shigella boydii BS512] [Translation elongation factor P @ Translation initiation factor 5A]

MATYYSNDFRAGLKIMLDGEPYAVEASEFVKPGKGQAFARVKLRRLLTGT

RVEKTFKSTDSAEGADVVDMNLTYLYNDGEFWHFMNNETFEQLSADAKAI

GDNAKWLLDQAECIVTLWNGQPISVTPPNFVELEIVDTDPGLKGDTAGTG

GKPATLSTGAVVKVPLFVQIGEVIKVDTRSGEYVSRVK

>fig|216598.1.peg.1921 [Shigella dysenteriae M131649] [Translation elongation factor P @ Translation initiation factor 5A]

MATYYSNDFRAGLKIMLDGEPYAVEASEFVKPGKGQAFARVKLRRLLTGT

RVEKTFKSTDSAEGADVVDMNLTYLYNDGEFWHFMNNETFEQLSADAKAI

GDNAKWLLDQAECIVTLWNGQPISVTPPNFVELEIVDTDPGLKGDTAGTG

GKPATLSTGAVVKVPLFVQIGEVIKVDTRSGEYVSRVK

>fig|198215.1.peg.3953 [Shigella flexneri 2a str. 2457T] [Translation elongation factor P @ Translation initiation factor 5A]

MATYYSNDFRAGLKIMLDGEPYAVEASEFVKPGKGQAFARVKLRRLLTGT

RVEKTFKSTDSAEGADVVDMNLTYLYNDGEFWHFMNNETFEQLSADAKAI

GDNAKWLLDQAECIVTLWNGQPISVTPPNFVELEIVDTDPGLKGDTAGTG

GKPATLSTGAVVKVPLFVQIGEVIKVDTRSGEYVSRVK

>fig|343509.6.peg.721 [Sodalis glossinidius str. 'morsitans'] [Translation elongation factor P @ Translation initiation factor 5A]

MASYSTNEFRSGLKIMLDGEPCAIIENEFVKPGKGQAFNRVRLRKLVSGK

VLEKTFKSGDSVEAADVMDINLTYLYNDGEFWHFMNNENFEQLAADAKVV

GDNAKWLVEQAECVLTLWNGQPIAVTPPNFVELEITETDPGLKGDTAGTG

GKPATLTTGAVVKVPLFVQIGEVIKVDTRSGDYVSRVK

>fig|377628.5.peg.3845 [Yersinia pestis Nepal516] [Translation elongation factor P @ Translation initiation factor 5A]

MASYYSNDFRPGLKIMFEGEPYAVESSEFVKPGKGQAFARVKMRRLLTGG

RVEKTFKSTDSLEGADVNDMNLTYLYNDGEFWHFMNNETYEQLQADAKAV

GDNGKWLIDQAECIVTLWNGQPIAVTPPNFVELEIVDTDPGLKGDTAGTG

GKPATLSTGAVVKVPLFVQVGEIIKVDTRSGEYVSRVK

>fig|349968.3.peg.2119 [Yersinia bercovieri ATCC 43970] [Translation elongation factor P @ Translation initiation factor 5A]

MASYYSNDFRPGLKIMFEGEPYSVESSEFVKPGKGQAFARVKMRRLLTGG

RVEKTFKSTDSLEGADVNDLNLTYLYNDGEFWHFMNNETYEQLQADAKAI

GDNGKWLVDQAECIVTLWNGLPIAVTPPNFVELEIVDTDPGLKGDTAGTG

GKPATLSTGAVVKVPLFVQVGEIIKVDTRSGEYVSRVK

>fig|229193.1.peg.499 [Yersinia pestis biovar Medievalis str. 91001] [Translation elongation factor P @ Translation initiation factor 5A]

MACFGLQCLPFFRLTTSFLRTIMASYYSNDFRPGLKIMFEGEPYAVESSE

FVKPGKGQAFARVKMRRLLTGGRVEKTFKSTDSLEGADVNDMNLTYLYND

GEFWHFMNNETYEQLQADAKAVGDNGKWLIDQAECIVTLWNGQPIAVTPP

NFVELEIVDTDPGLKGDTAGTGGKPATLSTGAVVKVPLFVQVGEIIKVDT

RSGEYVSRVK

>fig|502800.3.peg.3770 [Yersinia pseudotuberculosis YPIII] [Translation elongation factor P @ Translation initiation factor 5A]

MASYYSNDFRPGLKIMFEGEPYAVESSEFVKPGKGQAFARVKMRRLLTGG

RVEKTFKSTDSLEGADVNDMNLTYLYNDGEFWHFMNNETYEQLQADAKAV

GDNGKWLIDQAECIVTLWNGQPIAVTPPNFVELEIVDTDPGLKGDTAGTG

GKPATLSTGAVVKVPLFVQVGEIIKVDTRSGEYVSRVK

>fig|386656.4.peg.3548 [Yersinia pestis Pestoides F] [Translation elongation factor P @ Translation initiation factor 5A]

MASYYSNDFRPGLKIMFEGEPYAVESSEFVKPGKGQAFARVKMRRLLTGG

RVEKTFKSTDSLEGADVNDMNLTYLYNDGEFWHFMNNETYEQLQADAKAV

GDNGKWLIDQAECIVTLWNGQPIAVTPPNFVELEIVDTDPGLKGDTAGTG

GKPATLSTGAVVKVPLFVQVGEIIKVDTRSGEYVSRVK

>fig|349746.3.peg.3014 [Yersinia pestis Angola] [Translation elongation factor P @ Translation initiation factor 5A]

MASYYSNDFRPGLKIMFEGEPYAVESSEFVKPGKGQAFARVKMRRLLTGG

RVEKTFKSTDSLEGADVNDMNLTYLYNDGEFWHFMNNETYEQLQADAKAV

GDNGKWLIDQAECIVTLWNGQPIAVTPPNFVELEIVDTDPGLKGDTAGTG

GKPATLSTGAVVKVPLFVQVGEIIKVDTRSGEYVSRVK

>fig|349967.3.peg.3189 [Yersinia mollaretii ATCC 43969] [Translation elongation factor P @ Translation initiation factor 5A]

MASYYSNDFRPGLKIMFEGEPYSVESSEFVKPGKGQAFARVKMRRLLTGG

RVEKTFKSTDSLEGADVNDLNLTYLYNDGEFWHFMNNETYEQLQADAKAI

GDNGKWLVDQAECIVTLWNGLPIAVTPPNFVELEIVDTDPGLKGDTAGTG

GKPATLSTGAVVKVPLFVQVGEVIKVDTRSGEYVSRVK

>fig|187410.1.peg.601 [Yersinia pestis KIM] [Translation elongation factor P @ Translation initiation factor 5A]

MASYYSNDFRPGLKIMFEGEPYAVESSEFVKPGKGQAFARVKMRRLLTGG

RVEKTFKSTDSLEGADVNDMNLTYLYNDGEFWHFMNNETYEQLQADAKAV

GDNGKWLIDQAECIVTLWNGQPIAVTPPNFVELEIVDTDPGLKGDTAGTG

GKPATLSTGAVVKVPLFVQVGEIIKVDTRSGEYVSRVK

>fig|273123.1.peg.535 [Yersinia pseudotuberculosis IP 32953] [Translation elongation factor P @ Translation initiation factor 5A]

MASYYSNDFRPGLKIMFEGEPYAVESSEFVKPGKGQAFARVKMRRLLTGG

RVEKTFKSTDSLEGADVNDMNLTYLYNDGEFWHFMNNETYEQLQADAKAV

GDNGKWLIDQAECIVTLWNGQPIAVTPPNFVELEIVDTDPGLKGDTAGTG

GKPATLSTGAVVKVPLFVQVGEIIKVDTRSGEYVSRVK

>fig|349747.3.peg.3042 [Yersinia pseudotuberculosis IP 31758] [Translation elongation factor P @ Translation initiation factor 5A]

MASYYSNDFRPGLKIMFEGEPYAVESSEFVKPGKGQAFARVKMRRLLTGG

RVEKTFKSTDSLEGADVNDMNLTYLYNDGEFWHFMNNETYEQLQADAKAV

GDNGKWLIDQAECIVTLWNGQPIAVTPPNFVELEIVDTDPGLKGDTAGTG

GKPATLSTGAVVKVPLFVQVGEIIKVDTRSGEYVSRVK

>fig|349966.3.peg.2988 [Yersinia frederiksenii ATCC 33641] [Translation elongation factor P @ Translation initiation factor 5A]

MASYYSNDFRPGLKIMFEGEPYAVESSEFVKPGKGQAFARVKMRRLLTGS

RVEKTFKSTDSLEGADVNDLNLTYLYNDGEFWHFMNNETYEQLQADAKAI

GDNAKWLIDQAECIVTLWNGQPIAVTPPNFIELEIVDTDPGLKGDTAGTG

GKPATLSTGAVVKVPLFVQVGEVIKVDTRSGEYVSRVK

>fig|360102.4.peg.4591 [Yersinia pestis Antiqua] [Translation elongation factor P @ Translation initiation factor 5A]

MASYYSNDFRPGLKIMFEGEPYAVESSEFVKPGKGQAFARVKMRRLLTGG

RVEKTFKSTDSLEGADVNDMNLTYLYNDGEFWHFMNNETYEQLQADAKAV

GDNGKWLIDQAECIVTLWNGQPIAVTPPNFVELEIVDTDPGLKGDTAGTG

GKPATLSTGAVVKVPLFVQVGEIIKVDTRSGEYVSRVK

>fig|630.2.peg.351 [Yersinia enterocolitica 8081] [Translation elongation factor P @ Translation initiation factor 5A]

MASYYSNDFRPGLKIMFEGEPYAVESSEFVKPGKGQAFARVKMRRLLTGG

RVEKTFKSTDSLEGADVNDLNLTYLYNDGEFWHFMNNETYEQLQADAKAI

GDNAKWLIDQAECIVTLWNGQPISVTPPNFIELEIVDTDPGLKGDTAGTG

GKPATLSTGAVVKVPLFVQVGEVIKVDTRSGEYVSRVK

>fig|349965.3.peg.3188 [Yersinia intermedia ATCC 29909] [Translation elongation factor P @ Translation initiation factor 5A]

MATYYSNDFRPGLKIMFEGEPYAVESSEFVKPGKGQAFARVKMRRLLTGS

RVEKTFKSTDSAEGADVNDLNLTYLYNDGEFWHFMNNETYEQLQADAKAI

GDNDKWLIDQAECIVTLWNGQPIAVSPPNFVELEIVDTDPGLKGDTAGTG

GKPATLSTGAVVKVPLFVQVGEVIKVDTRSGEYVSRVK

>fig|214092.1.peg.518 [Yersinia pestis CO92] [Translation elongation factor P @ Translation initiation factor 5A]

MASYYSNDFRPGLKIMFEGEPYAVESSEFVKPGKGQAFARVKMRRLLTGG

RVEKTFKSTDSLEGADVNDMNLTYLYNDGEFWHFMNNETYEQLQADAKAV

GDNGKWLIDQAECIVTLWNGQPIAVTPPNFVELEIVDTDPGLKGDTAGTG

GKPATLSTGAVVKVPLFVQVGEIIKVDTRSGEYVSRVK

>fig|227377.1.peg.1739 [Coxiella burnetii RSA 493] [Translation elongation factor P @ Translation initiation factor 5A]

MATHSTNEFRGGLKVMVDGDPCSIIDNEFVKPGKGQAFNRVKFRNLKTGR

VLERTFKSGETLPAADVVEVEMQYLYNDGEFWHFMTSENYEQHAASKEAV

AEAKQWLKEEALCMVTMWNGVPLSVEPPNFVELKITETEPGVRGDTATGG

TKRAKLETGAVVRVPLFLNEGEIIKVDTRRGEYVSRAK

>fig|297245.3.peg.365 [Legionella pneumophila str. Lens] [Translation elongation factor P @ Translation initiation factor 5A]

MAVYSTNEFKNGLKVMVDDAPCSILDCEFVKPGKGQAFTRIKIRNLKTGR

VVERTFKSGDTLPSADVADVEMQYLYNDGEHWHFMVPDTFEQYAVTENIL

ADAAQWLKEQDVCVVTLWNNEPIQVTPPNFVILAITETDPGLKGDTSGGG

GKPATLETGAVVRVPLFVQTGELIKVDTRKGEYVSRAKE

>fig|272624.3.peg.1803 [Legionella pneumophila subsp. pneumophila str. Philadelphia 1] [Translation elongation factor P @ Translation initiation factor 5A]

MRTLAKIQQNLNYGAEMAVYSTNEFKNGLKVMVDDAPCSILDCEFVKPGK

GQAFTRIKIRNLKTGRVVERTFKSGDTLPSADVADVEMQYLYNDGEHWHF

MVPDTFEQYAVTENVLADAAQWLKEQDVCVVTLWNNEPIQVTPPNFVILA

ITETDPGLKGDTSGGGGKPATLETGAVVRVPLFVQTGELIKVDTRKGEYV

SRAKE

>fig|297246.3.peg.616 [Legionella pneumophila str. Paris] [Translation elongation factor P @ Translation initiation factor 5A]

MAVYSTNEFKNGLKVMVDDAPCSILDCEFVKPGKGQAFTRIKIRNLKTGR

VVERTFKSGDTLPSADVADVEMQYLYNDGEYWHFMVPDTFEQYAVTENVL

ADAAQWLKEQDVCVLTLWNNEPIQVTPPNFVILAITETDPGLKGDTSGGG

GKPATLETGAVVRVPLFVQTGELIKVDTRKGEYVSRAKE

>fig|243233.4.peg.624 [Methylococcus capsulatus str. Bath] [Translation elongation factor P @ Translation initiation factor 5A]

MAIVSTSEFKNGLKVMLDGDPCTMLESEFVKPGKGQAFNRVKLRNLKTGR

VVERTFKSGETLETADVVDVEMQYLYNDGELWHFMVPESFEQYAADQNAV

ADAKKWLKEQDICILTLFNNVPLAVQPPNFVELTITETDPGVRGDTSGGG

GKPATLETGAVVRVPLFVQTGEVIKVDTRTGEYVSRVK

>fig|393595.12.peg.2447 [Alcanivorax borkumensis SK2] [Translation elongation factor P @ Translation initiation factor 5A]

MHTSTLWKSFMANYSTSEFKSGLKVMLDGDPCSIIENEFVKPGKGQAFSR

VKLRNLRNGKVWERTFKSGDSLEGADVMDVSMQYIYSDGEFWHFMDQTSF

EQKQADETAVRDAKQWLKEEDICEVTLYNGEPLSVSPPNFVELEIIETDP

GLKGDTAGTGGKPATLSTGAVVRVPLFVQTGEIVKVDTRTGDYVGRIKQ

>fig|393595.12.peg.684 [Alcanivorax borkumensis SK2] [Translation elongation factor P @ Translation initiation factor 5A]

MKSKPLGISRLSLSIFAACSLQLAAALCCSIGASTFQEAFSMTRASELKK

SDVIEVNGTLYAIRQIEVQSPSARGAATLYRVKASAVGGGPKFEERFKGD

DDVATVALQRRAVQFSYVDGDDYIFMDNEDFSQYLLKQDDIRDELAFITE

ETQGVLALKVEESVIGLELPASVVLDVTETTPAMKAASSSARTKPATLNT

GLVVQVPEYIVAGEKVRVNTAERKFMSRA

>fig|349521.5.peg.4747 [Hahella chejuensis KCTC 2396] [Translation elongation factor P @ Translation initiation factor 5A]

MANYSTNEFKSGLKIMLDGDPCSIIENEFVKPGKGQAFNRVKFRNLKSGR

VGERTFKSGDSVEGADVVDLDMEYLYTDGEFYHFMLTDGSFEQHAADVSA

VGDTTKWLKEQDVYTVTLYNGAPLSVSPPNFVELEIVETDPGVRGDTAQG

GSKPAKLTTGAVVAVPLFINQGEMIKVDTRSGEYVSRVKS

>fig|290398.4.peg.1781 [Chromohalobacter salexigens DSM 3043] [Translation elongation factor P @ Translation initiation factor 5A]

MATYSTNEFKGGLKVMLDGDPCSILENEFVKPGKGQAFSRVKLRNLITGR

VWERTFKSGESLEGADVLELDMEYLYSDGDMWHFMRTDGSFEQYAVDKKA

MGDVEKWLKEQVVYTVTLWNDNAIAVSAPNFIELEVVETDPGLKGDTAQG

GSKPATLSSGAVVRVPLFINEGEVLKVDTRTGEYVSRA

>fig|400668.6.peg.914 [Marinomonas sp. MWYL1] [Translation elongation factor P @ Translation initiation factor 5A]

MANISTSEMRSGSKVMVDGDPCAIIDNEHVKPGKGQAFNRIKLRNLKTGR

VWERTFKSGDTLETADVMDTDMEYLYTDGEFWHFMAVDGSFEQHAADETA

VGDTIKWLKEQEKYVVTLYNGAPLAVAAPNFIELEVKETDPGVKGDTANG

GSKPAFLVTGAMVRVPLFINIGEVLRVDTRTGEYVSRATGK

>fig|228399.1.peg.309 [Actinobacillus pleuropneumoniae serovar 1 str. 4074] [Translation elongation factor P @ Translation initiation factor 5A]

MASYSTNDFKPGLKFIQDGEPCVIVENEFVKPGKGQAFTRTKIRKLISGK

VLEINFKSGTSVEAADVVDYNYTYSYKDEDFWYFMHPETFEQISVDEKAL

GDNDKWLVDQAECIITLWNGSAISVTPPNFVELEVVETDPGLKGDTAGTG

GKPATLSTGAVVRVPLFVQIGEVIRVDTRSGEYVSRVK

>fig|339671.5.peg.2069 [Actinobacillus succinogenes 130Z] [Translation elongation factor P @ Translation initiation factor 5A]

MASYTTTDFKPGLKFMQDGEPCVIIENEFVKPGKGQAFTRTRIRKLISGK

VLDVNFKSGTSVEAADVMDLNLNYSYKDEAFWYFMHPETFEQYSADAKAV

GEAEKWLLDQAECIVTLWNGSPISVTPPNFVELEVVDTDPGLKGDTAGTG

GKPATLSTGAVVRVPLFIQIGEVIKVDTRSGEYVSRVK

>fig|416269.5.peg.647 [Actinobacillus pleuropneumoniae L20] [Translation elongation factor P @ Translation initiation factor 5A]

MASYSTNDFKPGLKFIQDGEPCVIVENEFVKPGKGQAFTRTKIRKLISGK

VLEINFKSGTSVEAADVVDYNYTYSYKDEDFWYFMHPETFEQISVDEKAL

GDNDKWLVDQAECIITLWNGSAISVTPPNFVELEVVETDPGLKGDTAGTG

GKPATLSTGAVVRVPLFVQIGEVIRVDTRSGEYVSRVK

>fig|262727.1.peg.504 [Haemophilus influenzae R2846] [Translation elongation factor P @ Translation initiation factor 5A]

MATYTTSDFKPGLKFMQDGEPCVIVENEFVKPGKGQAFTRTRIRKLISGK

VLDVNFKSGTSVEAADVMDLNLTYSYKDDAFWYFMHPETFEQYSADAKAV

GDAEKWLLDQADCIVTLWNGAPITVTPPNFVELEIVDTDPGLKGDTAGTG

GKPATLSTGAVVKVPLFVQIGEVIRVDTRSGEYVSRVK

>fig|205914.1.peg.877 [Haemophilus somnus 129PT] [Translation elongation factor P @ Translation initiation factor 5A]

MATYTTSDFKPGLKFMQDGEPCVIIENEFVKPGKGQAFTRTRIRKLISGK

VLDVNFKSGTSVEAADVMDLNLTYSYKDEAFWYFMHPETFEQYSADAKAV

GDAEKWLLDQADCIVTLWNGAPITITPPNFVELEVVETDPGLKGDTAGTG

GKPATLSTGAVVKVPLFVQIGEVIKVDTRSGEYVSRVK

>fig|205914.5.peg.1339 [Haemophilus somnus 129PT] [Translation elongation factor P @ Translation initiation factor 5A]

MATYTTSDFKPGLKFMQDGEPCVIIENEFVKPGKGQAFTRTRIRKLISGK

VLDVNFKSGTSVEAADVMDLNLTYSYKDEAFWYFMHPETFEQYSADAKAV

GDAEKWLLDQADCIVTLWNGAPITITPPNFVELEVVETDPGLKGDTAGTG

GKPATLSTGAVVKVPLFVQIGEVIKVDTRSGEYVSRVK

>fig|71421.1.peg.307 [Haemophilus influenzae Rd KW20] [Translation elongation factor P @ Translation initiation factor 5A]

MQDGEPCVIVENEFVKPGKGQAFTRTRIRKLISGKVLDVNFKSGTSVEAA

DVMDLNLTYSYKDDAFWYFMHPETFEQYSADAKAVGDAEKWLLDQADCIV

TLWNGAPITVTPPNFVELEIVDTDPGLKGDTAGTGGKPATLSTGAVVKVP

LFVQIGEVIRVDTRSGEYVSRVK

>fig|281310.3.peg.214 [Haemophilus influenzae 86-028NP] [Translation elongation factor P @ Translation initiation factor 5A]

MATYTTSDFKPGLKFMQDGEPCVIVENEFVKPGKGQAFTRTRIRKLISGK

VLDVNFKSGTSVEAADVMDLNLTYSYKDDAFWYFMHPETFEQYSADAKAV

GDAEKWLLDQADCIVTLWNGAPITVTPPNFVELEIVDTDPGLKGDTAGTG

GKPATLSTGAVVKVPLFVQIGEVIRVDTRSGEYVSRVK

>fig|228400.4.peg.335 [Haemophilus somnus 2336] [Translation elongation factor P @ Translation initiation factor 5A]

MATYTTSDFKPGLKFMQDGEPCVIIENEFVKPGKGQAFTRTRIRKLISGK

VLDVNFKSGTSVEAADVMDLNLTYSYKDEAFWYFMHPETFEQYSADAKAV

GDAEKWLLDQADCIVTLWNGAPITITPPNFVELEVVETDPGLKGDTAGTG

GKPATLSTGAVVKVPLFVQIGEVIKVDTRSGEYVSRVK

>fig|233412.1.peg.1190 [Haemophilus ducreyi 35000HP] [Translation elongation factor P @ Translation initiation factor 5A]

MASYSTNDFKPGLKFIQDGEPCVIVENEFVKPGKGQAFTRTKIRKLISGK

VLEINFKSGTSVEAADVVDYNYTYSYKDEDFWYFMHPETFEQISVDAKAL

GDNDKWLVDQAECIITLWNGAAISVTPPNFVELAVVETDPGLKGDTAGTG

GKPATLSTGAVVRVPLFVQIGEIIKVDTRSGEYISRVK

>fig|221988.1.peg.482 [Mannheimia succiniciproducens MBEL55E] [Translation elongation factor P @ Translation initiation factor 5A]

MATYTTSDFKPGLKFMQDGEPCVIVENEFVKPGKGQAFTRTRIRKLISGK

VLDVNFKSGTSVEAADVMDLNLNYSYKDEDFWYFMHPETFEQYSADSKAV

GDAEKWLLDQAECIITLWNGSPISVTPPNFVELEVVDTDPGLKGDTAGTG

GKPATLSTGAVVKVPLFIQIGEVIKVDTRSGEYVSRVK

>fig|272843.1.peg.100 [Pasteurella multocida subsp. multocida str. Pm70] [Translation elongation factor P @ Translation initiation factor 5A]

MATYTTSDFKPGLKFMQDGEPCVIVENEFVKPGKGQAFTRTRIRKLISGK

VLDVNFKSGTSVEAADVMDLNLTYSYKDDAFWYFMHPETFEQYSADAKAI

GDAEKWLLDQADCIVTLWNGAPISVTPPNFVELEIIDTDPGLKGDTAGTG

GKPATLSTGAVVKVPLFVQIGEVIKVDTRSGEYVSRVK

>fig|62977.3.peg.1597 [Acinetobacter sp. ADP1] [Translation elongation factor P @ Translation initiation factor 5A]

MPVMASYSTNEFKQGLKVMLDGNPCSIMENEYVKPGKGQAFNRVKLRNLK

TGKVLEKTFKSGDSLEAADIVEVEMDYLYNDGELWNFMDPVTFEQIAADK

VAMGDAAKWLKDDSNEKCSIMLFNGVPLNVSAPNFVVLKIVETDPGVRGD

TSGGGGKPAKLETGAVVRVPLFVQQEDSVRVDTRTGEYLERA

>fig|400667.4.peg.2522 [Acinetobacter baumannii ATCC 17978] [Translation elongation factor P @ Translation initiation factor 5A]

MPVMANYSTNDFKPGLKVMLDSNPCSIMENEYVKPGKGQAFNRVKLRNLK

TGKVLEKTFKSGDTLEAADIVEVEMNYLYNDGEMWHFMDPESFEQIAADK

TAMGDAAKWLKDDSNETCTIMLFNGVPLNVNAPNFVVLKVVETDPGVRGD

TSGGGGKPAKLETGAVVRVPLFVQQEESVRVDTRTGEYLERA

>fig|349106.5.peg.533 [Psychrobacter sp. PRwf-1] [Translation elongation factor P @ Translation initiation factor 5A]

MASFSTNEFKAGLKVMYDGNPCAIIDNEFVKPGKGQAFNRVKLRNLRTGK

VLEQTFKSGESLEGADVVDTEMNYLYNDGEFWHFMHPETFEQLQADANAM

ADAKQWLKENGNDLCTITLFNGVPLSVTAPNFVELEIVETDPGVRGDTSG

GGGKPARLETGAVVRVPLFVQQNEVVRVDTRTGDYQTRVSQ

>fig|259536.4.peg.1955 [Psychrobacter sp. 273-4] [Translation elongation factor P @ Translation initiation factor 5A]

MASFSTNEFKAGLKVMLDGNPCAILENEFVKPGKGQAFNRVKLRNLRSGK

VLEQTFKSGDSLEAADVMDTEMNYLYNDGEFWHFMHPESFEQIQADKTAM

SDSTKWLKENSNALCTITLFNGAPLSVTPPNFVELQITETDPGVRGDTSG

GGGKPATLETGAVVRVPLFVQQGEVVRVDTRTGDYQTRVN

>fig|335284.3.peg.2449 [Psychrobacter cryohalolentis K5] [Translation elongation factor P @ Translation initiation factor 5A]

MASFSTNEFKSGLKVMLDGNPCAILENEFVKPGKGQAFNRVKLRNLRSGK

VLEQTFKSGDSLEAADVMDTEMNYLYNDGEFWHFMHPESFEQIQADKTAM

SDSIKWLKENSNALCTITLFNGAPLSVTPPNFVELQITETDPGVRGDTSG

GGGKPATLETGAVVRVPLFVQQGEVVRVDTRTGDYQTRVS

>fig|401614.5.peg.68 [Francisella tularensis subsp. novicida U112] [Translation elongation factor P @ Translation initiation factor 5A]

MASYSTNEFKGGLKVLIDGNPMVIVENEFVKPGKGQAFNRVKLKNLLNDR

VVEKTFKSGESVEAADVEELTTVYSYFDGDSYVFMHPETFEQYMVSEEAL

GETKKWLKDQDEYQVILFNGQPISIIAPNFVNLEIIETDPGLKGDTAGTG

GKPATLSTGAVVRVPLFVQTGEIIKVDTRTSTYVSRVKD

>fig|418136.4.peg.1792 [Francisella tularensis subsp. tularensis WY96-3418] [Translation elongation factor P @ Translation initiation factor 5A]

MASYSTNEFKGGLKVLIDGNPMVIVENEFVKPGKGQAFNRVKLKNLLNDR

VVEKTFKSGESVEAADVEELTTVYSYFDGDSYVFMHPETFEQYMVSEEAL

GETKKWLKDQDEYQVILFNGQPISIIAANFVNLEIIETDPGLKGDTAGTG

GKPATLSTGAVVRVPLFVQTGEIIKVDTRTSTYVSRVKD

>fig|177416.3.peg.668 [Francisella tularensis subsp. tularensis Schu 4] [Translation elongation factor P @ Translation initiation factor 5A]

MASYSTNEFKGGLKVLIDGNPMVIVENEFVKPGKGQAFNRVKLKNLLNDR

VVEKTFKSGESVEAADVEELTTVYSYFDGDSYVFMHPETFEQYMVSEEAL

GETKKWLKDQDEYQVILFNGQPISIIAANFVNLEIIETDPGLKGDTAGTG

GKPATLSTGAVVRVPLFVQTGEIIKVDTRTSTYVSRVKD

>fig|393011.11.peg.194 [Francisella tularensis subsp. holarctica OSU18] [Translation elongation factor P @ Translation initiation factor 5A]

MASYSTNEFKGGLKVLIDGNPMVIVENEFVKPGKGQAFNRVKLKNLLNDR

VVEKTFKSGESVEAADVEELTTVYSYFDGDSYVFMHPETFEQYMVSEEAL

GETKKWLKDQDEYQVILFNGQPISIIAANFVNLEIIETDPGLKGDTAGTG

GKPATLSTGAVVRVPLFVQTGEIIKVDTRTSTYVSRVKD

>fig|393115.8.peg.243 [Francisella tularensis subsp. tularensis FSC198] [Translation elongation factor P @ Translation initiation factor 5A]

MASYSTNEFKGGLKVLIDGNPMVIVENEFVKPGKGQAFNRVKLKNLLNDR

VVEKTFKSGESVEAADVEELTTVYSYFDGDSYVFMHPETFEQYMVSEEAL

GETKKWLKDQDEYQVILFNGQPISIIAANFVNLEIIETDPGLKGDTAGTG

GKPATLSTGAVVRVPLFVQTGEIIKVDTRTSTYVSRVKD

>fig|458234.10.peg.192 [Francisella tularensis subsp. holarctica FTA] [Translation elongation factor P @ Translation initiation factor 5A]

MASYSTNEFKGGLKVLIDGNPMVIVENEFVKPGKGQAFNRVKLKNLLNDR

VVEKTFKSGESVEAADVEELTTVYSYFDGDSYVFMHPETFEQYMVSEEAL

GETKKWLKDQDEYQVILFNGQPISIIAANFVNLEIIETDPGLKGDTAGTG

GKPATLSTGAVVRVPLFVQTGEIIKVDTRTSTYVSRVKD

>fig|484022.4.peg.772 [Francisella philomiragia subsp. philomiragia ATCC 25017] [Translation elongation factor P @ Translation initiation factor 5A]

MASYSTNEFKGGLKILIDGNPMVIVENEFVKPGKGQAFNRVKLKNLLNDR

VVEKTFKSGESVEAADVEELNAVYSYFDGDSYVFMHPETFEQYMVSQEAL

GETKKWLKDQDEYQIILFNGQPISIIAPNFVNLEIVETDPGLKGDTAGTG

GKPATLSTGAVVRVPLFVQTGEIIKVDTRTSTYVSRVKD

>fig|317025.3.peg.1823 [Thiomicrospira crunogena XCL-2] [Translation elongation factor P @ Translation initiation factor 5A]

MATVSTSEFKNGLKFLMDGQPCTIVDNQIVQPGKGQAFNRVKFRNLITGR

VLENTFKSGEKVEAADVMDTDLQYLYNDGEFWHFMDPNTFEQYQAGEAAV

ADVQKWLIEQDMCTVTLWNGDPISVLPPKQVVLEVTETDPGLKGDTAGTG

GKPATLSTGAVVQVPLFIQIGEKVICNTQTGEYISRAK

>fig|312309.3.peg.2338 [Vibrio fischeri ES114] [Translation elongation factor P @ Translation initiation factor 5A]

MASVSTNEFKGGLKFMLDNEPCSIIENEYVKPGKGQAFNRVKLRRLLSGK

TLEKTFKSGESFELADVVDVELDYLYNDGEFYHFMNSVSFEQIAADVKAV

GDTAKWLVENDTCTLTLWNDNPITVTPPNFVELEVTETDPGLKGDTQGTG

GKPATLSTGAVVRVPLFIAIGEVVKVDTRTGEYVGRVK

>fig|388396.7.peg.901 [Vibrio fischeri MJ11] [Translation elongation factor P @ Translation initiation factor 5A]

MASVSTNEFKGGLKFMLDNEPCSIIENEYVKPGKGQAFNRVKLRRLLSGK

TLEKTFKSGESFELADVVDVDLDYLYSDGEFYHFMNSVSFEQIAADVKAV

GDAAKWLVENDTCTLTLWNDNPITVTPPNFVELEVTETDPGLKGDTQGTG

GKPATLSTGAVVRVPLFIAIGEVVKVDTRTGEYVGRVK

>fig|338187.4.peg.147 [Vibrio harveyi ATCC BAA-1116] [Translation elongation factor P @ Translation initiation factor 5A]

MATVSTNEFKGGLKLMLDNEPCVILENEYVKPGKGQAFNRVKIRKLLSGK

VLEKTFKSGDTCEVADVMDIDLDYLYSDGEFYHFMNNETFEQIAADAKAV

GDNAKWLVENNTCMITLWNGNPITVTPPNFVELEVTDTDPGLKGDTQGTG

GKPATLSTGAVVRVPLFIAIGEVIKVDTRTAEYVGRVK

>fig|412966.3.peg.1376 [Vibrio cholerae 1587] [Translation elongation factor P @ Translation initiation factor 5A]

MATVSTNEFKGGLKIMLDNEPCVILENEYVKPGKGQAFNRVRIRKLLTGK

VLEKTFKSGDTAEVADVVDIDLDYLYNDGEFYHFMNNSTFEQLAADAKAV

GENAKWLVENNTCMLTLWNGNPIAVTPPNFVELEVTETDPGVKGDTQGTG

GKPATLSTGAVVRVPLFVQIGEVIKVDTRSAEYVGRVK

>fig|345072.3.peg.2470 [Vibrio cholerae MO10] [Translation elongation factor P @ Translation initiation factor 5A]

MATVSTNEFKGGLKIMLDNEPCVILENEYVKPGKGQAFNRVRIRKLLTGK

VLEKTFKSGDTAEVADVVDIDLDYLYNDGEFYHFMNNSTFEQLAADAKAV

GENAKWLVENNTCMLTLWNGNPIAVTPPNFVELEVTETDPGVKGDTQGTG

GKPATLSTGAVVRVPLFVQIGEVIKVDTRSAEYVGRVK

>fig|404974.3.peg.745 [Vibrio cholerae AM-19226] [Translation elongation factor P @ Translation initiation factor 5A]

MATVSTNEFKGGLKIMLDNEPCVILENEYVKPGKGQAFNRVRIRKLLTGK

VLEKTFKSGDTAEVADVVDIDLDYLYNDGEFYHFMNNSTFEQLAADAKAV

GENAKWLVENNTCMLTLWNGNPIAVTPPNFVELEVTETDPGVKGDTQGTG

GKPATLSTGAVVRVPLFVQIGEVIKVDTRSAEYVGRVK

>fig|150340.3.peg.1230 [Vibrio sp. Ex25] [Translation elongation factor P @ Translation initiation factor 5A]

MATVSTNEFKGGLKLMLDNEPCVILENEYVKPGKGQAFNRVKIRKLLSGK

VLEKTFKSGDTCEVADVMDIDLDYLYSDGEFYHFMNSETFEQIAADAKAV

GDNAKWLVENNTCMITLWNGNPITVTPPNFVELEVVDTDPGLKGDTQGTG

GKPATLSTGAVVRVPLFISIGEVIRVDTRTAEYVGRVK

>fig|314291.3.peg.274 [Vibrio splendidus 12B01] [Translation elongation factor P @ Translation initiation factor 5A]

MASVSTNEFKGGLKFMLDNEPCAIIDNEYVKPGKGQAFNRVKLRKLLSGK

VLEKTFKSGESFELADVVDVELGYLYNDGEFYHFMNNETFEQIAADVKAV

ADSAKWLVENDVCTLTLWNDNPITVTPPNFVEIPVTETDPGLKGDTQGTG

GKPATLATGAVVRVPLFIAIGEVVKVDTRTGEYVGRVK

>fig|314292.13.peg.429 [Vibrio angustum S14] [Translation elongation factor P @ Translation initiation factor 5A]

MASFSTNEFRGGMKIMLDNEPCVIIENEFVKPGKGQAFNRVRIRKLISGK

VLEKTFKSGESVEAADVIDTDLDYLYNDGEFYHFMNNETFEQIAADVKAV

GDNAKWLVENNTCTLTLWNGNPIAVTPPNFVELEVTETDPGLKGDTQGTG

GKPATLSTGAVVRVPLFIAIGEVIKVDTRSGEYVSRVK

>fig|412614.3.peg.2467 [Vibrio cholerae 2740-80] [Translation elongation factor P @ Translation initiation factor 5A]

MATVSTNEFKGGLKIMLDNEPCVILENEYVKPGKGQAFNRVRIRKLLTGK

VLEKTFKSGDTAEVADVVDIDLDYLYNDGEFYHFMNNSTFEQLAADAKAV

GENAKWLVENNTCMLTLWNGNPIAVTPPNFVELEVTETDPGVKGDTQGTG

GKPATLSTGAVVRVPLFVQIGEVIKVDTRSAEYVGRVK

>fig|243277.1.peg.2629 [Vibrio cholerae O1 biovar eltor str. N16961] [Translation elongation factor P @ Translation initiation factor 5A]

MATVSTNEFKGGLKIMLDNEPCVILENEYVKPGKGQAFNRVRIRKLLTGK

VLEKTFKSGDTAEVADVVDIDLDYLYNDGEFYHFMNNSTFEQLAADAKAV

GENAKWLVENNTCMLTLWNGNPIAVTPPNFVELEVTETDPGVKGDTQGTG

GKPATLSTGAVVRVPLFVQIGEVIKVDTRSAEYVGRVK

>fig|223926.1.peg.2845 [Vibrio parahaemolyticus RIMD 2210633] [Translation elongation factor P @ Translation initiation factor 5A]

MATVSTNEFKGGLKLMLDNEPCVILENEYVKPGKGQAFNRVKIRKLLSGK

VLEKTFKSGDTCEVADVMDIDLDYLYSDGEFYHFMNNETFEQIAADAKAV

GENAKWLVENNTCMITLWNGNPITVTPPNFVELEVTDTDPGLKGDTQGTG

GKPATLATGAVVRVPLFIAIGEVIKVDTRTGEYVGRVK

>fig|412967.3.peg.545 [Vibrio cholerae MAK 757] [Translation elongation factor P @ Translation initiation factor 5A]

MATVSTNEFKGGLKIMLDNEPCVILENEYVKPGKGQAFNRVRIRKLLTGK

VLEKTFKSGDTAEVADVVDIDLDYLYNDGEFYHFMNNSTFEQLAADAKAV

GENAKWLVENNTCMLTLWNGNPIAVTPPNFVELEVTETDPGVKGDTQGTG

GKPATLSTGAVVRVPLFVQIGEVIKVDTRSAEYVGRVK

>fig|216895.1.peg.1160 [Vibrio vulnificus CMCP6] [Translation elongation factor P @ Translation initiation factor 5A]

MATVSTNEFKGGLKLMIDSEPCVILENEYVKPGKGQAFNRVKIRKLLSGK

VLEKTFKSGDTCEVADVMDIDLDYLYSDGEFYHFMNNETFEQIAADAKAV

GENVKWLVENNTCMLTLWNGNPIAVTPPNFVELEVIETDPGLKGDTQGTG

GKPATLSTGAVVRVPLFIQIGEVIKVDTRSSEYVGRVK

>fig|196600.1.peg.3168 [Vibrio vulnificus YJ016] [Translation elongation factor P @ Translation initiation factor 5A]

MATVSTNEFKGGLKLMIDSEPCVILENEYVKPGKGQAFNRVKIRKLLSGK

VLEKTFKSGDTCEVADVMDIDLDYLYSDGEFYHFMNNETFEQIAADAKAV

GENVKWLVENNTCMLTLWNGNPIAVTPPNFVELEVIETDPGLKGDTQGTG

GKPATLSTGAVVRVPLFIQIGEVIKVDTRSSEYVGRVK

>fig|88888881.3.peg.1462 [Vibrio cholerae NRT36s] [Translation elongation factor P @ Translation initiation factor 5A]

MATVSTNEFKGGLKIMLDNEPCVILENEYVKPGKGQAFNRVRIRKLLTGK

VLEKTFKSGDTAEVADVVDIDLDYLYNDGEFYHFMNNSTFEQLAADAKAV

GENAKWLVENNTCMLTLWNGNPIAVTPPNFVELEVTETDPGVKGDTQGTG

GKPATLSTGAVVRVPLFVQIGEVIKVDTRSAEYVGRVK

>fig|412883.3.peg.2500 [Vibrio cholerae MZO-3] [Translation elongation factor P @ Translation initiation factor 5A]

MATVSTNEFKGGLKIMLDNEPCVILENEYVKPGKGQAFNRVRIRKLLTGK

VLEKTFKSGDTAEVADVVDIDLDYLYNDGEFYHFMNNSTFEQLAADAKAV

GENAKWLVENNTCMLTLWNGNPIAVTPPNFVELEVTETDPGVKGDTQGTG

GKPATLSTGAVVRVPLFVQIGEVIKVDTRSAEYVGRVK

>fig|314290.3.peg.3099 [Vibrio sp. MED222] [Translation elongation factor P @ Translation initiation factor 5A]

MASVSTNEFKGGLKFMLDNEPCAIIDNEYVKPGKGQAFNRVKLRKLLSGK

VLEKTFKSGESFELADVVDVELGYLYNDGEFYHFMNNETFEQIAADVKAV

ADSAKWLVENDVCTLTLWNDNPITVTPPNFVEIEVTETDPGLKGDTQGTG

GKPATLATGAVVRVPLFIAIGEVVKVDTRTGEYVGRVK

>fig|345073.6.peg.2813 [Vibrio cholerae O395] [Translation elongation factor P @ Translation initiation factor 5A]

MATVSTNEFKGGLKIMLDNEPCVILENEYVKPGKGQAFNRVRIRKLLTGK

VLEKTFKSGDTAEVADVVDIDLDYLYNDGEFYHFMNNSTFEQLAADAKAV

GENAKWLVENNTCMLTLWNGNPIAVTPPNFVELEVTETDPGVKGDTQGTG

GKPATLSTGAVVRVPLFVQIGEVIKVDTRSAEYVGRVK

>fig|314288.3.peg.3928 [Vibrio alginolyticus 12G01] [Translation elongation factor P @ Translation initiation factor 5A]

MATVSTNEFKGGLKLMLDNEPCVILENEYVKPGKGQAFNRVKIRKLLSGK

VLEKTFKSGDTCEVADVMDIDLDYLYSDGEFYHFMNSETFEQIAADAKAV

GENAKWLVENNTCMITLWNGNPITVTPPNFVELEVVDTDPGLKGDTQGTG

GKPATLSTGAVVRVPLFISIGEVIRVDTRTAEYVGRVK

>fig|316275.9.peg.2918 [Aliivibrio salmonicida LFI1238] [Translation elongation factor P @ Translation initiation factor 5A]

MASVSTNEFKGGLKFMFDNEPCSIIDNEYVKPGKGQAFNRVKLRKLLSGK

TLEKTFKSGESFELADVVDVELDYLYSDGEFFHFMNSVSFEQIAADVKSV

GDTAKWLVENNTCTVTLWNDNPITVTPPNFVEIEVTETDPGLKGDTQGTG

GKPATLATGAVVRVPLFIAIGEVVKVDTRTGEYVGRVK

>fig|314280.3.peg.5181 [Photobacterium profundum 3TCK] [Translation elongation factor P @ Translation initiation factor 5A]

MASFSTNEFRSGMKIMLDNEPCVIIENEFVKPGKGQAFSRVKIRKLLSGK

VLEKTFKSGESVEAADVVDVELDYLYNDGEFYHFMDNVSFEQIGADVKAV

GDNAKWLVENNTCTLTLWNGNPIVVTPPNFVELEVTETDPGLKGDTQGTG

GKPATLITGAVVRVPLFIQIGEVVKVDTRSGEYVGRVK

>fig|298386.1.peg.5576 [Photobacterium profundum SS9] [Translation elongation factor P @ Translation initiation factor 5A]

MASFSTNEFRSGMKIMLDNEPCVIIENEFVKPGKGQAFSRVKIRKLLSGK

VLEKTFKSGESVEAADVVEVELDYLYNDGEFYHFMDNVSFEQIAADVKAV

GDNAKWLVENNTCTLTLWNGNPIIVTPPNFVELEVTETDPGLKGDTQGTG

GKPATLITGAVVRVPLFIQIGEVIKADTRSGEYVSRVK

>fig|40324.1.peg.279 [Stenotrophomonas maltophilia K279a] [Translation elongation factor P @ Translation initiation factor 5A]

MASYGMNDVKNGMKILVNNQPAVIIDTEYVKPGKGQAFTRVKYRLIKDGR

TQEVTMKSTDSLDAADVVDTDMNFMYSDGEYWHFMDPESFEQVQATKAGM

GGAEKWLKGEESCVVTLWNGEPIFVQPPNFVELKITETDPGVRGDTSGGG

GKPATLETGAVVRVPLFVNQDEVIRVDTRSGEYSARVK

>fig|291331.3.peg.2648 [Xanthomonas oryzae pv. oryzae KACC10331] [Translation elongation factor P @ Translation initiation factor 5A]

MAAGQGAGMAVRRGGLRLSCEVTKPMPAGCGGRPILIRPATAWRVVVLSR

SCTMATVGMNDVKNGMKILVNNEPAVITETEYVKPGKGQAFTRMKYRFIK

SGRVVEMTMKATDDVEVADVVDTDMRYLYSDGEYWHFMDPETFEQVQTDK

AGMGGADKWLKGEEDCIVTLWNGAPIWVQPPNFVELKITETDPGVRGDTS

GGGGKPATLETGAVVRVPLFVNQDEIIKVDTRSGEYSARVK

>fig|314565.3.peg.1595 [Xanthomonas campestris pv. campestris str. 8004] [Translation elongation factor P @ Translation initiation factor 5A]

MATVGMNDVKNGMKILVNNEPAVITETEYVKPGKGQAFTRMKYRFIKSGR

VVEMTMKATDDVEVADVVDTDMRYLYTDGEYWHFMDPESFEQVQADKAGM

GGAEKWLKGEEDCIVTLWNGAPIWVQPPNFVELKITETDPGVRGDTSGGG

GKPATLETGAVVRVPLFVNQDEVIKVDTRSGEYSARVK

>fig|190485.1.peg.2232 [Xanthomonas campestris pv. campestris ATCC 33913] [Translation elongation factor P @ Translation initiation factor 5A]

MATVGMNDVKNGMKILVNNEPAVITETEYVKPGKGQAFTRMKYRFIKSGR

VVEMTMKATDDVEVADVVDTDMRYLYTDGEYWHFMDPESFEQVQADKAGM

GGAEKWLKGEEDCIVTLWNGAPIWVQPPNFVELKITETDPGVRGDTSGGG

GKPATLETGAVVRVPLFVNQDEVIKVDTRSGEYSARVK

>fig|190486.1.peg.2340 [Xanthomonas axonopodis pv. citri str. 306] [Translation elongation factor P @ Translation initiation factor 5A]

MATVGMNDVKNGMKILVNNEPAVITETEYVKPGKGQAFTRMKYRFIKSGR

VVEMTMKATDDVEVADVVDTDMRYLYSDGEYWHFMDPDTFEQVQTDKAGM

GGADKWLKGEEDCIVTLWNGTPIWVQPPNFVELKITETDPGVRGDTSGGG

GKPATLETGAVVRVPLFVNQDEIIKVDTRSGEYSARVK

>fig|316273.3.peg.1843 [Xanthomonas campestris pv. vesicatoria str. 85-10] [Translation elongation factor P @ Translation initiation factor 5A]

MATVGMNDVKNGMKILVNNEPAVITETEYVKPGKGQAFTRMKYRFIKSGR

VVEMTMKATDDVEVADVVDTDMRYLYSDGEYWHFMDPETFEQVQTDKAGM

GGADKWLKGEEDCIVTLWNGTPIWVQPPNFVELKITETDPGVRGDTSGGG

GKPATLETGAVVRVPLFVNQDEIIKVDTRSGEYSARVK

>fig|155920.1.peg.1299 [Xylella fastidiosa Ann-1] [Translation elongation factor P @ Translation initiation factor 5A]

MASYGMNDVKNGMKILVNAEPAVITDTEYVKPGKGQAFTRVKYRLIKSGR

VQEVTMKSTDTLEAADVVDTDMQYLYSDGEYWHFMQQETFEQVQADKNGM

GGAEKWLKGEEQCVVTLWNGVPIGVQPPNFVELKITETDPGLRGDTSDGG

GKPATLETGAVVRVPLFVNQDEVIKVDTRSGEYVSRVK

>fig|160492.1.peg.2458 [Xylella fastidiosa 9a5c] [Translation elongation factor P @ Translation initiation factor 5A]

MRNFSMASYGMNDVKNGMKILVNAEPAVITDTEYVKPGKGQAFTRVKYRL

IKSGRVQEVTMKSTDTLEAADVVDTDMQYLYSDGEYWHFMQQETFEQVQA

DKNGMGGAEKWLKGEEQCVVTLWNGVPIGVQPPNFVELKITETDPGLRGD

TSGGGGKPATLETGAVVRVPLFVNQDEVIKVDTRSGEYVSRVK

>fig|405440.3.peg.1488 [Xylella fastidiosa M12] [Translation elongation factor P @ Translation initiation factor 5A]

MASYGMNDVKNGMKILVNAEPAVITDTEYVKPGKGQAFTRVKYRLIKSGR

VQEVTMKSTDTLEAADVVDTDMQYLYSDGEYWHFMQQETFEQVQADKNGM

GGAEKWLKGEEQCVVTLWNGVPIGVQPPNFVELKITETDPGLRGDTSGGG

KPATLETGAVVRVPLFVNQDEVIKVDTRSGEYVSRVK

>fig|183190.1.peg.1428 [Xylella fastidiosa Temecula1] [Translation elongation factor P @ Translation initiation factor 5A]

MASYGMNDVKNGMKILVNAEPAVITDTEYVKPGKGQAFTRVKYRLIKSGR

VQEVTMKSTDTLEAADVVDTDMQYLYSDGEYWHFMQQETFEQVQADKNGM

GGAEKWLKGEEQCVVTLWNGVPIGVQPPNFVELKITETDPGLRGDTSGGG

GKPATLETGAVVRVPLFVNQDEVIKVDTRSGEYVSRVK

>fig|314285.3.peg.2822 [gamma proteobacterium KT 71] [Translation elongation factor P @ Translation initiation factor 5A]

MANYSTNEFRSGLKVMLDGDPCSILDNEFVKPGKGQAFNRVRMRNLKTGR

VWDRTFKSGESLEGADVMDTTMEYLYTDGEFWFFMEPNTYEQYQADAAAV

GDTHKWLKEQEPCEVTLYNGTPLSVSPPNFVELEITETDPGLKGDTAQGG

SKPATLSTGAVVRVPLFISQGEVIRVDTRSGEYVARASKS

>fig|523791.4.peg.566 [Kangiella koreensis DSM 16069] [Translation elongation factor P @ Translation initiation factor 5A]

MLDGEPCTVVDNQFVRPGKGQAFSKTKVLYLLTGRTVEKTFKSGESIETA

DVMDTEMEYLYNDGEFWYFMDPNTFEQVSADKNAVSDKAVYLIEQDKCSI

TLWNGNPIDVTPPNFVVLEVTDTDPGLRGDTSGGGSKPATMHTGAVVKVP

LFVNIGDKLKVDTRTGDYVSRA

>fig|314283.3.peg.2473 [Reinekea sp. MED297] [Translation elongation factor P @ Translation initiation factor 5A]

MANYSTNEFRSGLKVLLDGEPCAIVENEFVKPGKGQAFNRVKLKNLKTGR

TWERTFKSGESIEGADVLDIDMEYLYTDGEFWHFMKTDGSFEQISADATA

MGDTVEWLKEQEVFQVTLFNGAPISVTAPNHIELEVSETDPGVKGDTATG

GSKPAKLVSGAVVKVPLFINEGEVIRVDTRSKEYLSRAKS

>fig|97084.1.peg.3212 [Bacteriovorax marinus SJ] [Translation elongation factor P @ Translation initiation factor 5A]

MARELQTTELKKGVRLELENKPYQIMKADFTNPGKGSAFTICKLKNLETG

AVFDRTFKSGVATGVFEPDLELLIVEYMYSDPDGFNFMDQTTYETIHVTT

EQVGEAAGYLQEGIKLDLLFYKGNPIAIDLPNFVVLKIAETDPGLKGDTA

QGGTKVAIMETGLQVKVPLFIKEGEIIKIDTRTGDYIERAKE

>fig|264462.1.peg.2273 [Bdellovibrio bacteriovorus HD100] [Translation elongation factor P @ Translation initiation factor 5A]

MYETSDFRKGLKIMLEGKPYVIVDFQHVKPGKGNQFTRTKLRNMLTGQNL

ESTFKSGEKFEVPNVENKEMSFLYKDDTGYNFMSQETFEQIAMSEEDLGE

AKYYLTENLKVVILFYNEKAVACDVPKAVNLTVAQTDPGIKGDRVTGATK

PATMETGLTVGVPLHINEGDVLRIDTSTGEYVERVSQK

>fig|96561.3.peg.2352 [Desulfococcus oleovorans Hxd3] [Translation elongation factor P @ Translation initiation factor 5A]

MYEAGELRKGLKVEIDGDPYVIMEFEFVKPGKGQALYKCKLKNMLTGSQY

DHTYRSGDKVGRANLEERKMEYLYFDGENYCFMDCTTYDQIFVPPSQVAE

VLDLLKENTVCDVLFFDNRAIGVTLPNFVELAITEADPWVKGDTASGSNK

PVTVETGCVLQVPPFIEVGEVIKIDTRTKNYVERVKK

>fig|177439.1.peg.845 [Desulfotalea psychrophila LSv54] [Translation elongation factor P @ Translation initiation factor 5A]

MLSASDLRKGLKLDIEGSPYIIIDFDFSKPGKGQALYRCKMRNMITGNQL

VKTYRSSDKFEKASLEERKMQFLYSQGEEYHFMDNENYDQLFITKDMLGD

NIYFLQDNMDVDVLFFDEKPIDITLPIFVNLEVTRADPWVKGDTSGTDTK

PITVETGYQLQVPPFVEQGDKIQIDTRTGQYVTRVKQ

>fig|891.1.peg.1130 [Desulfuromonas acetoxidans] [Translation elongation factor P @ Translation initiation factor 5A]

MYTCSDLKKGLKLMIDGEPHVIAGFDFTKPGKGQALYKCKLRNMITGALF

DRTYRSGENFEPAPLEERDMQYLYQDETGYVFMDNKTYEQISLREETLGD

DRYFLVDNMEVKILMFGELGIGITLPNFVTLRVTMAEPWVKGDTAAGNNK

PATVETGYNLQVPSFVEEGILIQIDTRTGEYVTRVKE

>fig|891.1.peg.1200 [Desulfuromonas acetoxidans] [Translation elongation factor P @ Translation initiation factor 5A]

MYTCADLKKGLKLMIEGEPHVIVQFDFTKPGKGQALYKCKLRNMITGSLF

DRTYRSGESFEPAALEDRDMQYLYQDEEGYVFMDNKSFEQVTLTEETLGD

DKYFLKDNMEVKILMYNGRGIGITLPNFVNLRVTQADPWVKGDTAAGNNK

PATVESGYNLAVPSFVEEGDLIQIDTRTGDYVTRVKE

>fig|269799.3.peg.1893 [Geobacter metallireducens GS-15] [Translation elongation factor P @ Translation initiation factor 5A]

MYTVADLKKGLKITLDGDPYLVTAFEFSKPGKGQALYRTKMRNMITGTTL

DRTYRSGETFEPARLEERQMQYLYKEDNHYTFMDNQTFEQVQMSEDAVGD

AKNFMIDNIQVDVLMFGEKAIGVSLPNFVNLRVVQTDPWVKGDTSGSDSK

PATVETGYVLRVPPFIEEGEMIVVDTRTGDYSTRVKG

>fig|351605.4.peg.339 [Geobacter uraniireducens Rf4] [Translation elongation factor P @ Translation initiation factor 5A]

MYTTSDFKRGLVIQLDGAPCILVDVTFQSPTARGSNTMVKTKYRNLITAQ

VLEKTFRSGDKVDEADFERHKGQFLYTDGDKGVFMDLETYEQFELESDGF

EPIAPFLLEGTEVQLGLFQGRMVNVDLPMTVELTVTDTAPVLKNATATAQ

TKEAILETGHKLQVPPYLNSGEKIKVDTRDGKFISRA

>fig|351605.4.peg.2116 [Geobacter uraniireducens Rf4] [Translation elongation factor P @ Translation initiation factor 5A]

MYTVADLRKGLKITLDGDPYIVIAFDFAKPGKGQALYRTKMRNMINGTIL

DRTYRSGETFEPASLEDRKMQYLYKEDDHYCFMDNQSYEQIHVDENALGD

AKNYLIDNLPVDVLLFKGKAIGVDVPNFVNLRVVQTDPWAKGDTSGSDSK

PATVETGYVLRVPPFIEEGELITIDTRTGEYSTRVKG

>fig|243231.1.peg.1740 [Geobacter sulfurreducens PCA] [Translation elongation factor P @ Translation initiation factor 5A]

MYTVADLKKGLKLTLDGAPYLVIAFEFSKPGKGQALYRTKMRNMITGVIL

DRTYRSGETFEPARLEERRMQYLYKEDTHYTFMDNQTFEQVQMDEDAVGD

AKNFLIDNLEVDILLFGEKAIGVTLPNFVNLRVVQTDPWVKGDTSGSDSK

PATVETGYILRVPPFIEEGEMIVIDTRSGEYSTRVKG

>fig|351605.3.peg.1032 [Geobacter uraniumreducens Rf4] [Translation elongation factor P @ Translation initiation factor 5A]

MYTTSDFKRGLVIQLDGAPCILVDVTFQSPTARGSNTMVKTKYRNLITAQ

VLEKTFRSGDKVDEADFERHKGQFLYTDGDKGVFMDLETYEQFELESDGF

EPIAPFLLEGTEVQLGLFQGRMVNVDLPMTVELTVTDTAPVLKNATATAQ

TKEAILETGHKLQVPPYLNSGEKIKVDTRDGKFISRA

>fig|351605.3.peg.184 [Geobacter uraniumreducens Rf4] [Translation elongation factor P @ Translation initiation factor 5A]

MYTVADLRKGLKITLDGDPYIVIAFDFAKPGKGQALYRTKMRNMINGTIL

DRTYRSGETFEPASLEDRKMQYLYKEDDHYCFMDNQSYEQIHVDENALGD

AKNYLIDNLPVDVLLFKGKAIGVDVPNFVNLRVVQTDPWAKGDTSGSDSK

PATVETGYVLRVPPFIEEGELITIDTRTGEYSTRVKG

>fig|338963.3.peg.2466 [Pelobacter carbinolicus DSM 2380] [Translation elongation factor P @ Translation initiation factor 5A]

MLTCSDLRKGTKLMIDGEPHVIVQFDFTKPGKGQALYKCKLRNMITGSLF

DRTYRSGESFEPAALEERDMQYLYQDETGYVFMDQKSYEQTTLGEEALGD

QKYFLVDNMEVKILMFGDRGIGITLPNFVNLRVTMAEPWVKGDTAAGNNK

PATVETGYNLQVPSFVEEGTLIQIDTRTGEYVTRVKE

>fig|338963.3.peg.1117 [Pelobacter carbinolicus DSM 2380] [Translation elongation factor P @ Translation initiation factor 5A]

MATTADLKRNLVIQIDNAPCLVLDVNTQTPSARGGSTLIKTKYRNLLTGQ

VLEKAFKGGERIEDADFERRKGQFLYAIDEQGVFMDLESYDQYELGGDMY

EDIRGYLVDGMELQMGVFQGQVVSIDLPQTVELTVVETAPALKNATATAQ

TKEAVLETGLRLQVPPYLESGERIKVDTRECRFISRA

>fig|338966.5.peg.3246 [Pelobacter propionicus DSM 2379] [Translation elongation factor P @ Translation initiation factor 5A]

MYSTSDFKKGLVIQLDGAPCLIVDVTVQSPTARGANTMVKTKYRNLITSQ

VLEKTFRSGDKVDEADFERHKGQFLYADGGRGVFMDLENYEQFEVEQDEF

APLSPYLLEGTEVVLGLFEARLVNVELPMTVELTVAETVPVMKNATATAQ

TKEAMLETGLKLQVPPYLEVGERIKVDTRDGRFISRA

>fig|338966.5.peg.1804 [Pelobacter propionicus DSM 2379] [Translation elongation factor P @ Translation initiation factor 5A]

MYTAADLKKGLKIIIEGDPYIIIAFDFTKPGKGQALYRTKMRNMINGTIL

DRTYRSGETFEPASLEERQMEYLYKEGTHYTFMDQQTYEQVIMEEDTIGD

AKNFLLENIKVEVLLFGEKAIGVTIPNFVNLRVTQTDPWAKGDTSGNDSK

PATVETGYVLRVPPFIEEGELITIDTRTGEYSTRVKG

>fig|404589.4.peg.616 [Anaeromyxobacter sp. Fw109-5] [Translation elongation factor P @ Translation initiation factor 5A]

MAGGATAHELPRPSRAGLPERRRSRRVLAHGHGRDRDRHAPHRRGRRRGR

ALAPRRVLGRPAQRARGGRGARGGRRGRGGDRDRQAARRGPAGLAGVLHG

GRADARGEPREGALPAPRHRAEARGRALGTGGRPSGQAAAAAAARRADHS

HPDLRRSDTAMAETLDTSAFRRGLKIEIDREPWEIIEFQHVKPGKGSAFV

RTRIKNLITGRTIEKTFKSGDVVGKPDIDEKEMQFLYREGDHFNFMDNKT

YDQTFLTEEQMGEARNFIKDNTTTHILFFNGKAIGVTLPNAMDLKVVKCD

PGIRGDTVSGATKPATLETGYVVNVPLFINEGEILRIDTRTGEYLTRVAG

>fig|290397.13.peg.646 [Anaeromyxobacter dehalogenans 2CP-C] [Translation elongation factor P @ Translation initiation factor 5A]

MAETLDTSAFRRGLKIEIEREPWEIIEFQHVKPGKGSAFVRTRIKNLMTG

RTIEKTFKSGDVVGKPDIDEREMQFLYREGEHYNFMDNKNYEQTFLTAEQ

MGDAKNFIKDNTTTHILFFNGKAIGVTLPNSMDLKVVKCDPGIRGDTVSG

ATKPAELETGYVVNVPLFINEGDTLRIDTRSGEYLTRVAG

>fig|246197.19.peg.5153 [Myxococcus xanthus DK 1622] [Translation elongation factor P @ Translation initiation factor 5A]

MAGVIDTSEFRKGLKIEIDGEPFEIADFQHVKPGKGSAFVRTTIRSLLTG

RVLQPTLKSGEKVGKPDIEEKDMQYLYVQGEEFYFMDTRNYEQTFLGEKV

LGEAKNFLKENINVSVLFYNGKAIGVTLPNSVDLKVTQCDPGIRGDTVSG

ALKPAVLETGYSVYVPLFIEEGDVLKIDTRDGKYLTRVATRG

>fig|448385.11.peg.8500 [Sorangium cellulosum So ce 56] [Translation elongation factor P @ Translation initiation factor 5A]

MACAGGRARAKGAANANPRPVVDGGRVRGPPTRGRGRERGPPSRARVPRT

RIPGLADRPPLRYQEALPMDTSDIRKGLKFMVDGQPYSVIDFQFVKPGKG

QAFTRVKIRNMATGAVLERTYKSGEKLEPADVEERSLQYIYPEGTDFVFM

DPATGEQLTVPGDKIGDDSKWLSDGMSIDVTLFNGLPIGVSMPPHVVLQI

VSSEPGVKGDTASGATKPATVSTGATVNVPLFVKEGEWIKIDTTDGKYLE

RVNR

>fig|56780.10.peg.865 [Syntrophus aciditrophicus SB] [Translation elongation factor P @ Translation initiation factor 5A]

MYSASDLRKNLRIKLEGDPYIITEFNFVKPGKGQALYRCKLKNMITGNQF

ERTFRSVDNFEAADLQEKKMQFLYTEEDRYCFMDNTSYEQIFLTADQVGD

AAHFLIDNLEVEILLFEDKPLGISLPNFVDLVVTKADPWAKGDTVSGNTK

PVTLQTGYQIMVPPFIEEGEKIRVDTRTGEYLTRVKG

>fig|335543.6.peg.81 [Syntrophobacter fumaroxidans MPOB] [Translation elongation factor P @ Translation initiation factor 5A]

MGVTLTAGDLRKGLKLEMDGEPYIIVDFEFSKPGKGQALYRCRLKNMITG

SQFDRTYRSGDKFQSADLEEQDMQFLYKQGDSYHFMNTTSYEQIEMSAAQ

VGDATNYLIENLVVSMLMFQGRPIGISLPNFVELKVIRSDPGIKGDTAAG

ATKPATMETGFIIQVPLFIEEGETLKIDTRNGSYVERVKV

>fig|156889.7.peg.1007 [Magnetococcus sp. MC-1] [Translation elongation factor P @ Translation initiation factor 5A]

MLSHTEMKQGKRVLIDDQPWIIVKADFVKPGKGQAFTKIKVKNLMDGRVI

ERTFKSSDSVAKADVVDVEMQYLYNDGELYHFMNPATFEQVALSEKQVEE

CKKWLKENEVYEVTLWENRAINVVPPSFMILEITECEPGVRGDTVTGATK

PAVVESGASIKVPLFVEIGARVKVDTRTGEYMERAKG

>fig|267671.1.peg.3667 [Leptospira interrogans serovar Copenhageni str. Fiocruz L1-130] [Translation elongation factor P @ Translation initiation factor 5A]

MSVLGGMINRLCILNIRQLGILVWRNLGFLKVSLSSSINSVPDSFNSTSR

FLWGARRVLFLDNGFVSKEIEGGKLFVGNSMTLGITEVKKGMVLKVEGDL

YSVVKTEFVNPGKGSAFIRTKLKNLTRNSSIERTFKAAEKLESVELEKRN

MTICYTEGDDIIFMDSNDFEQMPVSKEYVEDILPFLKEETPMEVTFYEGK

PIGVIPPNFSILEVTYAEEGLKGDTSGTAQKRITVETGGEINVPIFVKQG

DVIKIDLRDLTYVERVSK

>fig|189518.1.peg.4654 [Leptospira interrogans serovar Lai str. 56601] [Translation elongation factor P @ Translation initiation factor 5A]

MTPSIQEYHTSTCLTSKNHTNLWGARRVLFLDNGFVSKEIEGGKLFVGNS

MTLGITEVKKGMVLKVEGDLYSVVKTEFVNPGKGSAFIRTKLKNLTRNSS

IERTFKAAEKLESVELEKRNMTICYTEGDDIIFMDSNDFEQMPVSKEYVE

DILPFLKEETPMEVTFYEGKPIGVIPPNFSILEVTYAEEGLKGDTSGTAQ

KRITVETGGEINVPIFVKQGDVIKIDLRDLTYVERVSK

>fig|243275.1.peg.961 [Treponema denticola ATCC 35405] [Translation elongation factor P @ Translation initiation factor 5A]

MIRGGDIAKGTVLLNKGTPYLVVEREFVNPGKGAAFARVKMKNLRDGSVL

MQTIKTADTVEDAVVDTHKCQYQYKDGDQFMFMDTESFESISVPAETIGD

KEHYLREGDEYDILIWENEPIDVRIPTKMIFIVEQSENYIKGDTVSGATK

PIVTETGLVVRVPLFIKQGEKILVNTETNEYQERVNS

>fig|243276.1.peg.524 [Treponema pallidum subsp. pallidum str. Nichols] [Translation elongation factor P @ Translation initiation factor 5A]

MIRGGDIAKGTVLLHKGAPYLVVEREFVNPGKGAAFARVKMKHLRDGSVL

TQTVKTSDTVEDAVVDSHRAQYQYDDGECFVFMDTRSFEQIFVSKGNVPG

RERYLREGDEYDILIWNGESIDIKIPTKMVFRVAHSEPYLKGDTVSGATK

PVTTETGLVVRVPLFIKQGEKILINTETNEYQERVND
