# Supplementary material for: Predicting the pathway involved in post-translational modification of Elongation factor P in a subset of bacterial species
Source: Biol Direct. 2010 Jan 13;5:3. doi: 10.1186/1745-6150-5-3 (PMC2821294; doi:10.1186/1745-6150-5-3)
Supplement: Additional file 4 — Text S3. List of EF-P sequences from organisms that do not have have yjeA/yjeK. [file 1745-6150-5-3-S4.DOC]

**Supplemental text S3 List of EF-P sequences from organisms that do not have have *yjeA/yjeK***

>fig|273035.4.peg.1099 [Spiroplasma kunkelii CR2-3x] [Translation elongation factor P @ Translation initiation factor 5A]

MISVNDFRPGLTFQYEGNIYVVIEAQHSKSGRGQAHVKTKVKNLRSNAIT

NITFTGGDKVKKAIIDKVEMQYLYDDGTNVIFMDTQTYEQLEIPSTNLVW

EKKFLKEGVMASVTKYDGEVLGIILPDKVDLTVTEAEVAVKGDTSSGAMK

KAVLETGWELQVPLFIKKGEVITVSTSDGKYSGRA

>fig|1313.3.peg.1561 [Streptococcus pneumoniae INV104B] [Translation elongation factor P @ Translation initiation factor 5A]

MIEASKLKAGMTFETADGKLIRVLEASHHKPGKGNTIMRMKLRDVRTGST

FDTSYRPEEKFEQAIIETVPAQYLYKMDDTAYFMNTETYDQYEIPVVNVE

NELLYILENSDVKIQFYGTEVIGVTVPTTVELTVAETQPSIKGATVTGSG

KPATMETGLVVNVPDFIEAGQKLVINTAEGTYVSRA

>fig|336982.3.peg.2704 [Mycobacterium tuberculosis F11] [Translation elongation factor P @ Translation initiation factor 5A]

MATTADFKNGLVLVIDGQLWTITEFQHVKPGKGPAFVRTKLKNVLSGKVV

DKTFNAGVKVDTATVDRRDTTYLYRDGSDFVFMDSQDYEQHPLPEALVGD

AARFLLEGMPVQVAFHNGVPLYIELPVTVELEVTHTEPGLQGDRSSAGTK

PATLQTGAQINVPLFINTGDKLKVDSRDGSYLGRVNA

>fig|279010.5.peg.461 [Bacillus licheniformis ATCC 14580] [Translation elongation factor P @ Translation initiation factor 5A]

MISVNDFRTGLTIEVDGGIWRVVDFQHVKPGKGAAFVRSKLRNLRTGAIQ

EKTFRAGEKVARAQIETKTMQYLYANGDQHVFMDTTSYEQLELNEKQIEH

ELKFLLENMSVQIMMYQTETIGIELPNTVELKVVETEPGIKGDTASGGTK

PAKTETGLVVNVPFFVNEGDTLVVNTSDGSYVSRA

>fig|313593.3.peg.2993 [Flavobacterium sp. MED217] [Translation elongation factor P @ Translation initiation factor 5A]

MATTSDIRNGMCIHYNHDIYKIVEFLHVKPGKGPAFVRTKLKSVTNGKVL

DNTFSAGHKIEEVRVETKGYQFLYSDGEWYHFMNTDDYTQIRLLESALDQ

PGLLKEGEVVSIAINTEDGLPLSVDMPAHVILEVTATEPGVKGNTATNAT

KPATVETGAEVNVPLFINEGDKIKVETEKGTYKERIKE

>fig|498211.3.peg.2057 [Cellvibrio japonicus Ueda107] [Translation elongation factor P @ Translation initiation factor 5A]

MGNTMKIAQECRAGNVVMIDGSPWVVQKAEYNKSGRNAAVVKMKLKNLLS

GINTETVYKADDKFEDILLDRKEVTYSYYADPMYVFMDGEYNQYEVTKED

LGDLLPWIEDGMEDVCDAVFYEGKVISVTAPTSIVREIAYTEPAVRGDTS

GKVMKVAKLKNGTELSVAAFVEIGEKIIIDTRTGEYKSRAKD

>fig|306254.1.peg.340 [Campylobacter coli RM2228] [Translation elongation factor P @ Translation initiation factor 5A]

MASYSMGDLKKGLKIEIDGIPFKIVEYQHVKPGKGPAFVRIKIKSFIDGK

VLEKTFHAGDKCEAPNLEEKTMQYLYDDGENCQFMDTESYEQVAISDEDV

GEAKKWMLDGMMVDVLFHNGKAIGVEVPQVVELKIVETAPNFKGDTQGSN

KKPATLETGAVVQIPFHVLEGEVIRVDTVRGEYIERANK

>fig|314225.3.peg.2014 [Erythrobacter litoralis HTCC2594] [Translation elongation factor P @ Translation initiation factor 5A]

MRRFTGALFLTARHSPMKISGVDIRPGNIIEYEGGIWKVAKIQHTQPGKG

GAYMQVEMKNLQDGRKTNVRFRSADTVEKVRLDTQDYQFLYEDGDQLVFM

DQDTYEQINLDSDLLGDARPFLQDGMTVQLELWEEKPISVQLPQQVEADI

VEADAVVKGQTASSSYKPAVLDNGVRIMVPPHIESGTRIVVDVYEQTYVG

KAG

>fig|518766.5.peg.489 [Rhodothermus marinus DSM 4252] [Translation elongation factor P @ Translation initiation factor 5A]

MADTSDFRNGLVLVWKGDLWQIVEFLHVKPGKGGAFVRTKLKNVRTGQVV

DNTFRAGERVETARIERRPHQFLYEDELGLHFMNLETYEQITISPDLVPR

RGFLKEGGEADVLVHAETETPITVEIPKHVELRVVETEPGVRGDTATGGS

KPAKLESGAVIQVPLFINEGDVVRVNTETGEYITRVATAEAG

>fig|272623.1.peg.710 [Lactococcus lactis subsp. lactis Il1403] [Translation elongation factor P @ Translation initiation factor 5A]

MVLAKDLKSGMTFLNGEKLLRVMEASHHKPGKGNTIMRMKLKDVRSGSTF

DDTYRPEDKFEQAVIETVTAQYLYSMDGIANFMNNETYEQYEIPVEQVKD

ELLYVLENTDVKIQFYGTEVIGIQLPTTVVLEVTETQPSIKGATVTGSGK

PATMETGLVVNVPDFVEAGTKLEINTQTGEYLKRA

>fig|479431.5.peg.2071 [Nakamurella multipartita DSM 44233] [Translation elongation factor P @ Translation initiation factor 5A]

MASTSDFKNGLVLNLEGQLWTITEFQHVKPGKGPAFVRTTLKNVLSGKVV

DKTFNAGVKVETATVDKRDMTYLYKDGSEYVFMDGDTYDQIHIPAATVGS

AADYLLENQEALIAQNEGVVLYVELPASVELVVSHTDPGLQGDRSTGGTK

PATLETGAEIAVPLFLNTGDKIKVDTRDGRYLGRVNS

>fig|74546.3.peg.151 [Prochlorococcus marinus str. MIT 9312] [Translation elongation factor P @ Translation initiation factor 5A]

MISSNDFRTGTTIELDGQVWRVVEFLHVKPGKGSAFVRTKLKSVQSGNVV

EKTFRAGESVQQAILEKSNLQHTYVESGDYVFMDMTSFEETRLSSEQIGK

GSKYLKEGMEVNVILHNGKVLEVELPISITLKVTETDPGVKGDTASGGTK

PAILETGAQVMVPLFISVGEMIKVDTRNDSYLGREN

>fig|272626.1.peg.1381 [Listeria innocua Clip11262] [Translation elongation factor P @ Translation initiation factor 5A]

MISVNDFKTGLTIEVDNGIWRVLDFQHVKPGKGAAFVRSKLRNLRTGAIQ

EKTFRGGEKVAKAQIDNRKMAYLYADGTNHVFMDNESYEQIELPEDQIAH

ELKFLKENMEINIIMYQGETIGIDLPNTVELVVTATDPGIKGDTSSGGSK

PATLETGLVVQVPFFVNEGDKLVINTTEAAYVSRA

>fig|216432.3.peg.1634 [Croceibacter atlanticus HTCC2559] [Translation elongation factor P @ Translation initiation factor 5A]

MASTSDIRKGLCIRYNNDIYKIIEFLHVKPGKGPAFVRTKLKSITTGKVL

DNTFSAGHKIEDIRVETHKFQFLYNDGEFYHFMNQEDYTQIRLVESVLDA

PGLMKEGEVVSIQINTEDNMPLSVEMPAYVVLEIAHTEPGVKGNTATNAT

KPATVETGAEVNVPLFINEGDKVKIDTETGNYKERVTD

>fig|257309.1.peg.1284 [Corynebacterium diphtheriae NCTC 13129] [Translation elongation factor P @ Translation initiation factor 5A]

MATTADFKNGLVLKIDNKLQQIIEFQHVKPGKGPAFVRTKLKDVVSGKVT

DKTFNAGVKVETATVDRRDMTYLYNDGSSYVVMDEKTFEQAELAPHIFGD

AARFLLENTTVQVSFHEGEPLFAELPISLDLRIEHTDPGLQGDRSTGGTK

PATLETGAEIQVPLFIETGNVVKVDTRDGSYLSRVNN

>fig|446462.5.peg.3171 [Actinosynnema mirum DSM 43827] [Translation elongation factor P @ Translation initiation factor 5A]

MATTNDLKNGLVLNLDGQLWTVTAFQHVKPGKGGAFVRTTLKHVLTGKVV

DKTFNAGTKVETATVDKRGMTYLYKDGSDFVFMDGDTFEQINVPGETVAD

AANFMLENQEAVVAIHEGVALYVELPTSVELLIQHTDPGLQGDRSTGGTK

PATLETGAEIQVPLFVTTGEKIKVDTRDGRYLGRVSS

>fig|521098.4.peg.808 [Alicyclobacillus acidocaldarius subsp. acidocaldarius DSM 446] [Translation elongation factor P @ Translation initiation factor 5A]

MISSNDFRNGTTIEYDGSVWRVIEFMHVKPGKGSAFVRTKLKNVKTGAIR

ETTFRAGEKVPRARIETREMQYLYNDGENYTFMDTETYEQINIPRAQLEY

ELNFLKENMNCFIVQYQGEIIGIDLPNTVELEVIDTEPGIRGDTATGGSK

SATVETGYTLQVPFFINVGDKVVIDTRSGEYVSRA

>fig|205920.8.peg.638 [Ehrlichia chaffeensis str. Arkansas] [Translation elongation factor P @ Translation initiation factor 5A]

MAERGSDIRPGHVLEHNNALYLVVKVMHTQPGKGGAYIQAEMKNLKTGAK

QYERFRADGDIKRAIVDESDYQYIYGDGSMITIMHLKTYEQLTISKDILG

DKSIYLQDNIIITLVFYNGEIISAKVPDYVTLQVVETEAVIKGQTVSSSA

YKVAMLENNQRISVPTFIKPGDRIVVYTPDDSYYERAKG

>fig|479436.4.peg.541 [Veillonella parvula DSM 2008] [Translation elongation factor P @ Translation initiation factor 5A]

MISSNDFRPGVTVEIDGNVWQVVDFQHVKPGKGAAFVRTKMKNLQTGSVV

ERTFNAGEKVPKAHVDRRRMQYLYESDGSYTFMDNETYDQVELTLEQLGD

AKNFLLENMEVSIMFFQGIVIGIDLPVAVELRVVETDPGIRGDTATGGNK

PAVLETGYTVKVPLFIEVDDVLRIDTRTGQYIERA

>fig|262724.1.peg.758 [Thermus thermophilus HB27] [Translation elongation factor P @ Translation initiation factor 5A]

MISVTDLRPGTKVKMDGGLWECVEYQHQKLGRGGAKVVAKFKNLETGATV

ERTFNSGEKLEDIYVETRELQYLYPEGEEMVFMDLETYEQFAVPRSRVVG

AEFFKEGMTALGDMYEGQPIKVTPPTVVELKVVDTPPGVRGDTVSGGSKP

ATLETGAVVQVPLFVEPGEVIKVDTRTGEYVGRA

>fig|264730.3.peg.3804 [Pseudomonas syringae pv. phaseolicola 1448A] [Translation elongation factor P @ Translation initiation factor 5A]

MKTGKELKPGTVIRLENDPWLVQKAEFTKSGRNSAIMKTKLKNLLTGYKT

EIVYSADDKLDDVILDRKEATLSFISGDTYTFMDTTDYTMYELNAEDIES

VLPFVEEGMTDVCEAVFFEDRLVSVELPTTIVRQVDYTEGSARGDTSGKV

MKPAKLKNGTELSVADFIEIGDMIEIDTREGGSYKGRAK

>fig|290402.34.peg.1672 [Clostridium beijerincki beijerinckii NCIMB 8052] [Translation elongation factor P @ Translation initiation factor 5A]

MSYFINLEGSLMITAGDIRKGTTFELDGQVFTVVEFLHVKPGKGAAFVRT

KLRNVISGGVTDTTFNPTTKLQEAVIERKEMQYLYSDGELYYFMDQETYE

QIPLNYEKVEDAIRFLKENMFATIKFFKGDAFSVEAPNFVELLITQSEPG

VKGNTATNAMKPATLETGAIVNVPMFVNEGDVIRVDTRTGEYMERV

>fig|1596.1.peg.1615 [Lactobacillus gasseri] [Translation elongation factor P @ Translation initiation factor 5A]

MTMISVNEFKNGLTIEYNNDLWRIVEFQHVKPGKGSAFVRSKLKSLRTGA

VQEYTFRSTAKVNTADIQTKAMQYLYNDGTSFVFMDTTTYEQLEIPEAQV

ERESKFLKENMVVNVIMHDGETLGVDLPNTVDLEVAETEPNIKGDTSSGG

GKPATMETGLVVNVPFFINQGDVLTINTADGTYVSRANK

>fig|273119.1.peg.302 [Ureaplasma parvum serovar 3 ATCC 700970] [Translation elongation factor P @ Translation initiation factor 5A]

MATIIQAKDLRAGHTFLYKGNIYQVIENSFNKTAMREGIVKCKVKNLRTG

AITIEVLTGEKLEQAVIEKSKMTFSYDDGSGYVFMDNDTYEQISIPYSQL

SWEKNFIEEGTEVSVMRYDGELMGVSLPDQLVVTIIEAEEAVQGNSVQNA

TKRAWLESKWEFQVPQFIKSGEKVIINPSNGQYVGRAK

>fig|313598.3.peg.447 [Tenacibaculum sp. MED152] [Translation elongation factor P @ Translation initiation factor 5A]

MATTSDIRNGLCIRYNNDIYKIIEFLHVKPGKGPAFVRTKLKSVTNGKVV

DNTFPAGRKIDDVRVETHKFQYLYNDGDTYHFMNEQDYSQIQLQKNALDT

PELMKEGEVVTIIINSEDDMPLSVEMPLSVILEVTHTEPGVKGNTATNAT

KPATVETGATVNVPLFINEGDKIKVETTKGTYQERIKE

>fig|1148.1.peg.2282 [Synechocystis sp. PCC 6803] [Translation elongation factor P @ Translation initiation factor 5A]

MISSNDFRTGTSIVMDGAVWKVVEFLHVKPGKGSAFVRTKLKSVQTGNVV

EKTFRAGETVPQANIEKSVMQHTYKDGDQYVFMDMETFEEVSIAPDTLGD

KAKFIKEEMEVSVVTWDGTILDVELPTSVVLEIVETDPGVKGDTATGGTK

PAILETGAQVMVPLFIAQGERIKVDTRDGSYLGRDNA

>fig|266940.5.peg.3105 [Kineococcus radiotolerans SRS30216] [Translation elongation factor P @ Translation initiation factor 5A]

MGSLHPRPVPVAGDDGQRDGLRDDVASTNDLKNGTVLNLDGNLWSVVEFQ

HVKPGKGGAFVRTKLKNVLSGKVVDRTFNAGSKVETATVDKRDMQYLYKD

GEDFVFMDSATYDQLYVSATTVGDAANYMLESTEAIVATHEGTPLYVELP

ASVVLEITYTEPGLQGDRSTGGTKPATVETGYEIQVPLFLETGTKVKVDT

RTGDYLGRVND

>fig|281090.3.peg.472 [Leifsonia xyli subsp. xyli str. CTCB07] [Translation elongation factor P @ Translation initiation factor 5A]

MASTADIKNGIVINIDGQLWSVIEFQHVKPGKGGAFVRTKLKNVTTGKTV

DRTYNAGAKIDITNVDRRDYQYLYQDGADFVFMDTSDYDQITIPGPIVGD

AANFMLENQNVTVALHEGSPLYVELPASVVLEITYTEPGLQGDRSTGGTK

PATVQTGYQIQVPLFLETGTKVRVDTRTGDYLGRVND

>fig|292414.1.peg.369 [Silicibacter sp. TM1040] [Translation elongation factor P @ Translation initiation factor 5A]

MKNLRNGSKLNERFRSADKVERVRLEQKDQQFLYETDGMLVFMDTETYEQ

IELPADLLGDRRPFLQDGMTIVVEFYESEALNATVPQKVTCKIVETEPVV

KGQTAANSFKPAILDNGVKVMVPPFVGQDEMIIVNTETMEYSERA

>fig|349161.4.peg.1035 [Desulfotomaculum reducens MI-1] [Translation elongation factor P @ Translation initiation factor 5A]

MISTSDFRTGLTVEIDNDVYQIIEFQHVKPGKGAAFVRTKMRNMRTGAVI

ERTFNPNEKLAPARIERQEVQYLYNDGESYNMMNMETYDQFTVSKADMGD

AIKWLKENMTLTIASFNGNIIAVDLPNTVELAVVETAPGIKGDTASGGGK

PATLETGAVINVPFFVNIGDVLQVDTRTGNYVKRV

>fig|208964.1.peg.2851 [Pseudomonas aeruginosa PAO1] [Translation elongation factor P @ Translation initiation factor 5A]

MKTAQEFRAGQVANINGAPWVIQKAEFNKSGRNAAVVKMKLKNLLTGAGT

ETVFKADDKLEPIILDRKEVTYSYFADPLYVFMDSEFNQYEIEKDDLEGV

LTFIEDGMTDICEAVFYNDKVISVELPTTIVRQIAYTEPAVRGDTSGKVM

KTARLNNGAELQVSAFCEIGDSIEIDTRTGEYKSRVKA

>fig|359786.3.peg.172 [Staphylococcus aureus subsp. aureus JH9] [Translation elongation factor P @ Translation initiation factor 5A]

MISVNDFKTGLTISVDNAIWKVIDFQHVKPGKGSAFVRSKLRNLRTGAIQ

EKTFRAGEKVEPAMIENRRMQYLYADGDNHVFMDNESFEQTELSSDYLKE

ELNYLKEGMEVQIQTYEGETIGVELPKTVELTVTETEPGIKGDTATGATK

SATVETGYTLNVPLFVNEGDVLIINTGDGSYISRG

>fig|471857.4.peg.2009 [Saccharomonospora viridis DSM 43017] [Translation elongation factor P @ Translation initiation factor 5A]

MATPPDTGERTLVATTNDLKNGMVLVLDGELWSVVSFQHVKPGKGGAFVR

TTLKHVTSGKVVDRTFNAGTKVDTATVDRRQMTYLYNDGTDYVFMDGQTY

EQLELSADLVGDAAKFLLENTEVEVGMYEETPLYVELLTESTAVVEHTEP

GVQGDRSTGGTKPATLETGAEIQVPLFVNTGDKVKVDTRDGRYLGRA

>fig|292459.1.peg.1798 [Symbiobacterium thermophilum IAM 14863] [Translation elongation factor P @ Translation initiation factor 5A]

MISVNDLRNGMTIEMDGTVYQVIEFLHVKPGKGAAFVRTKLKNILTGATI

ETTFRAGEKVEQANVDRREYQFLYADQGVWVFMNNETFEQIELTEEQVGN

APNFLLENMTVQIASWKGQVIGVDLPNTVELKVVETEPGFKGDTATGTYK

PAKLETGYVVQVPLFVNTGDVIKVDTRTGEYLSRA

>fig|393121.3.peg.1210 [Listeria monocytogenes FSL J2-071] [Translation elongation factor P @ Translation initiation factor 5A]

MISVNDFKTGLTIEVDNGIWRVLDFQHVKPGKGAAFVRSKLRNLRTGAIQ

EKTFRGGEKVAKAQIDNRKMAYLYADGTNHVFMDNESYEQIELPEDQIAH

ELKFLKENMEINIIMYQGETIGIDLPNTVELVVTATDPGIKGDTSSGGSK

PATLETGLVVQVPFFVNEGDKLVINTTEAAYVSRA

>fig|350058.5.peg.2608 [Mycobacterium vanbaaleni vanbaalenii PYR-1] [Translation elongation factor P @ Translation initiation factor 5A]

MASTADFKNGLVLQIDGQLWQIVEFQHVKPGKGPAFVRTKLKNVVSGKVV

DKTYNAGVKVETATVDRRDATYLYRDGSDFVFMDSEDYEQHPLPEALVGR

AADFLLESMPVQIAFHDGSPLYLELPVTVELVVASTEPGLQGDRSSAGTK

PATLETGAEIQVPLFINTGDKLKVDSRDGSYLGRVNA

>fig|469378.4.peg.216 [Cryptobacterium curtum DSM 15641] [Translation elongation factor P @ Translation initiation factor 5A]

MAISTADFKNGICIEYNGKLWTIAEFQHVKPGKGGAFVRTKLRDVRSGRV

VEYTFNSGTKFDSVRLEERSMQYLYTDGADYYFMDPTSYEQMGIPADVVG

ATAQWLKENDEVTLQYAGDELISLTPKMFVELAVAETDPGFKGDTVQGGT

KPATLETGAVVQVPMYVNVGDVLQIDTRDGRFIKRV

>fig|525897.4.peg.1426 [Desulfomicrobium baculatum DSM 4028] [Translation elongation factor P @ Translation initiation factor 5A]

MLATSDFKKGKKIEIDGAPCEILECSHYKPGKGGAFMRTKYRNLLTGSVV

EQNFRSGIKFPKPDLEVREMQYLYNEGESYAFMDMTTFEQISVPHDLIGE

KGGYLKEAQAYKVMLYQGRPLDLEMSGSVVLEVTMTDPGMKGDTVTGASK

PATLETGIVVNVPLFVEIGTKIKVNTETGEYLGRE

>fig|267608.1.peg.1069 [Ralstonia solanacearum GMI1000] [Translation elongation factor P @ Translation initiation factor 5A]

MALKIAQELRAGNVFMIGNDPMVVLKTEYSRSGRNAAVVKMKYKNLLTGA

PSESVFKADDKMDQIILDKKECTYSYFADPMYVFMDTDYNQYEVEADSMG

DAIHYLEDGMAAEVTFYNEKAISVELPTTLVREIEYTEPAVKGDTSSGKV

LKMAKIKGGFEIQVPLFCSTGDKIEIDTRTHEYRSRAN

>fig|340047.10.peg.508 [Mycoplasma capricolum subsp. capricolum ATCC 27343] [Translation elongation factor P @ Translation initiation factor 5A]

MSVNDLRPGTTFIYDGNIYLVLEQAFSKTGRQQGKVTVKAKNMRTGARVE

LTFTGGEKVDKAMIERKEMQYLYNDGNDAYLMNTETYEQIQIPMTRLEWE

RNFLVDGLMINMTEFEGEVLGIDLPVKVELTVVEAEAAVKGDTTSGAQKK

AVLETGLEIMVPLFVNQGTKIIVSSSDGKYVGRA

>fig|59919.1.peg.26 [Prochlorococcus marinus subsp. pastoris str. CCMP1986] [Translation elongation factor P @ Translation initiation factor 5A]

MISSNDFRTGTTIEIDGQVWRVVEFLHVKPGKGSAFVRTKLKSVRNGNVV

EKTFRAGESVQQAVLEKSNLQHTYVESGDYVFMDMISFEETRLSSDQIGR

GSKYLKEGMEVNVIFYKDKVLEVELPISITLKVTETDPGVKGDTASGGTK

PAILETGAQVMVPLFISVGEMIKVDTRNDSYLGRDN

>fig|319225.3.peg.1938 [Pelodictyon luteolum DSM 273] [Translation elongation factor P @ Translation initiation factor 5A]

MLYSLSLIRGFQEEAPVRKYFTRAMTSISNISKGSIIRFKGEPHIMESLV

HRTPGNLRAFYQASMRNLKSGRNVEYRFSATESVDLIVTERKEYQYLYPD

GSDFVMMDNETFDQINVPESAIGPAVRFLKDGVNAMIVFSDDGAILSVEL

PTFVEVEITETSPATKDDRATSGTKTAVIETGAEVNVPMFLQTGSIIRVD

TRSGEYIERVKK

>fig|95656.5.peg.504 [Ureaplasma parvum serovar 1] [Translation elongation factor P @ Translation initiation factor 5A]

MATIIQAKDLRAGHTFLYKGNIYQVIENSFNKTAMREGIVKCKVKNLRTG

AITIEVLTGEKLEQAVIEKSKMTFSYDDGSGYVFMDNDTYEQISIPYSQL

SWEKNFIEEGTEVSVMRYDGELMGVSLPDQLVVTIIEAEEAVQGNSVQNA

TKRAWLESKWEFQVPQFIKSGEKVIINPSNGQYVGRAK

>fig|76114.4.peg.735 [Azoarcus sp. EbN1] [Translation elongation factor P @ Translation initiation factor 5A]

MKTAQELRSGNVIMVGADALVVQKAEYNKSGRNSAVVKMKLKNLLTGAPS

ESVYKADDKFEVVQLDKKEVTYSYFADPMYVFMDADYEQYEVEAENMTDA

LKYLEDGLQCEVVFYNGKAISVDLPNSVVREVIYTEPAVKGDTSGKVMKP

AKIASGFELPVPAFVEIGDKIEIDTRTDEYKNRVK

>fig|196620.1.peg.1481 [Staphylococcus aureus subsp. aureus MW2] [Translation elongation factor P @ Translation initiation factor 5A]

MISVNDFKTGLTISVDNAIWKVIDFQHVKPGKGSAFVRSKLRNLRTGAIQ

EKTFRAGEKVEPAMIENRRMQYLYADGDNHVFMDNESFEQTELSSDYLKE

ELNYLKEGMEVQIQTYEGETIGVELPKTVELTVTETEPGIKGDTATGATK

SATVETGYTLNVPLFVNEGDVLIINTGDGSYISRG

>fig|314271.3.peg.3184 [Rhodobacterales bacterium HTCC2654] [Translation elongation factor P @ Translation initiation factor 5A]

MPKINGNEIRPGNVLEHNDGLWAAVKVDHVKPGKGGAFAQVELRNLRNGS

KLNERFRSADKVERVRLEQKDMQFLYESDGMLTFMDTETFDQVELPAEIL

GDRRPFLQDGMTIVVEFHDTEALNATLPQKVTCTIEETEPVVKGQTAANS

FKPALLDNGVRITVPPFVGQGEAIIVNTETMEYSERA

>fig|290434.1.peg.312 [Borrelia garinii PBi] [Translation elongation factor P @ Translation initiation factor 5A]

MAVVKSSEIEKGSFLLIKGTPHIVLEREFSKTGRGGAIVRLKLKNLKNKF

VIRETLKGADTAEAIEIYEVSAQYLYKDKDVLVFMDLETYDQVSLDLKES

ANFQDKVPFLQESETYALIMFDNVVIDIKLAPKIAFEVVEVEAAVKGDTV

TNAMKNITLNTGLVVKAPLFINVGDKVLVNSETKEYAERIKS

>fig|324602.4.peg.2830 [Chloroflexus aurantiacus J-10-fl] [Translation elongation factor P @ Translation initiation factor 5A]

MAAGTTSDLRNGIVIRYNNDLYQVVEFQHVAPGNWRAFVRMKLKSLTTGK

VIEDRVRAGAEIDIVRIERRPMQYLYREGDSFIFMDNDTFDQIPVSADLV

GDAVRFMKENETVDLVYDAEKDTIIGVELPIFVNLKVVETTVAVRGDTAT

NVTKPATLETGAVIEVPAFINEGDVLKIDTRTGEYITRV

>fig|283165.1.peg.995 [Bartonella quintana str. Toulouse] [Translation elongation factor P @ Translation initiation factor 5A]

MKINGNEIRPGNVIEHQGSLWVAIKCNAVKPGKGGAFNQVEMKNLIDGTK

FNERFRAAETVERVRLEQKDFTFLYQQGDALIFMDSQSYEQLELQKDFVG

ERAAFLQEGMTVTVELYQEKPIGISLPDQVSVTIAEADPALKGQTVTASY

KPAILENGIRILVPPFINAGEHVIVDTNELVYLRRANDKG

>fig|360108.3.peg.1555 [Campylobacter jejuni subsp. jejuni 260.94] [Translation elongation factor P @ Translation initiation factor 5A]

MASYSMGDLKKGLKIEIDGIPFKIVEYQHVKPGKGPAFVRIKIKSFIDGK

VLEKTFHAGDKCEAPNLEDKTMQYLYDDGENCQFMDTQTYEQVAISDDDV

GEAKKWMLDGMMVDVLFHNGKAIGVEVPQVVELKIIETAPNFKGDTQGSN

KKPATLETGAVVQIPFHVLEGEVIRVDTVRGEYIERANK

>fig|264199.3.peg.472 [Streptococcus thermophilus LMG 18311] [Translation elongation factor P @ Translation initiation factor 5A]

MIEASKLRAGMTFVTNDGKLLKVLEASHHKPGKGNTIMRMKLRDVRSGST

FDTSYRPEEKFEQAIIETVPAQYLYQMDDTAYFMNTETYDQYEIPVVNVQ

EELKFILENSDVKIQFYGTEVIGVQVPTTVELTVTETQPSIKGATVTGSG

KPATLETGLVVNVPDFIEAGQKLVINTAEGTYVSRA

>fig|66692.3.peg.2170 [Bacillus clausii KSM-K16] [Translation elongation factor P @ Translation initiation factor 5A]

MISVNDFKTGLTIEVDNGIWQVMEFQHVKPGKGAAFVRSKLRNLRTGAIQ

EKTFRAGEKVAKAHIENRRMQYLYASGDTHTFMDNETYEQLELQTAQIEY

ELKFLKENMEVHVISYESETLGVEVPNTVILEVVETEPGIKGDTASGGTK

PATLETGLTVQVPFFVNQGDKLVIDTRSASYVSRA

>fig|267410.1.peg.2213 [Listeria monocytogenes str. 4b H7858] [Translation elongation factor P @ Translation initiation factor 5A]

MISVNDFKTGLTIEVDNGIWRVLDFQHVKPGKGAAFVRSKLRNLRTGAIQ

EKTFRGGEKVAKAQIDNRKMAYLYADGTNHVFMDNESYEQIELPEDQIAH

ELKFLKENMEINIIMYQGETIGIDLPNTVELVVTATDPGIKGDTSSGGSK

PATLETGLVVQVPFFVNEGDKLVINTTEAAYVSRA

>fig|351627.4.peg.1238 [Caldicellulosiruptor saccharolyticus DSM 8903] [Translation elongation factor P @ Translation initiation factor 5A]

MVEAGDFRRGLTIEYDGQIFQVIEFLHVKPGKGAAFVRTKLKNIKTGAVI

EKTFRPDERMPLAHIERREMQYLYNDGELYYFMDTQTYEQIALNQEMVGD

ALKFVKENMTVTILSHNGSVFGVEPPRFVELEVIDTEPGFKGDTQTGATK

PAKVETGAVIQVPLFINVGDKIKIDTSTEEYLSRV

>fig|158879.1.peg.1404 [Staphylococcus aureus subsp. aureus N315] [Translation elongation factor P @ Translation initiation factor 5A]

MISVNDFKTGLTISVDNAIWKVIDFQHVKPGKGSAFVRSKLRNLRTGAIQ

EKTFRAGEKVEPAMIENRRMQYLYADGDNHVFMDNESFEQTELSSDYLKE

ELNYLKEGMEVQIQTYEGETIGVELPKTVELTVTETEPGIKGDTATGATK

SATVETGYTLNVPLFVNEGDVLIINTGDGSYISRG

>fig|331978.3.peg.3107 [Burkholderia pseudomallei Pasteur] [Translation elongation factor P @ Translation initiation factor 5A]

MKTAQELRVGNVVMIGNDAWVVSKTEYNKSGRNAAVVKMKLKNLLNGGGQ

ESVYKADDKFEVVVLDRKEVTYSYFADPMYVFMDADYNQYEVEAEMMGDA

LNYLEDGMACEVVFYNEKAISVELPTILVREITYTEPAVKGDTSSGKVLK

NAKLATGFELQVPLFCNTGDKIEIDTRTNEYRSRA

>fig|234826.3.peg.497 [Anaplasma marginale str. St. Maries] [Translation elongation factor P @ Translation initiation factor 5A]

MAERGSDIRPGQILDHNGSLYLVVKTMHTQPGKGGAYIQAELKNLKTGAK

YQERFRSDGYVKRAIVEEVEYQYIFGDGASLTLMNTATYEQVSISADMLG

EKGVYLKEGIILTLSFYQGQVVAARVPDYVVLEVVETESVIKGQTASSSY

KSAVLENGERISVPPFIKVGERIVVYTVDDTYYERAKD

>fig|74547.1.peg.31 [Prochlorococcus marinus str. MIT 9313] [Translation elongation factor P @ Translation initiation factor 5A]

MISSNDFRTGTSIELDGSVWRVVEFLHVKPGKGSAFVRTKLKAVQSGNVV

EKTFRAGEMLPQALLEKSTLQHTYMESGDYVFMDMSSYEETRLTAQQIGD

SRKYLKEGMEVNVVSWNGNPLEVELPNSVVLEITETDPGVKGDTATGGTK

PAILETGAQVMVPLFLSVGEKIKVDTRNDSYLGREN

>fig|36870.1.peg.285 [Wigglesworthia glossinidia endosymbiont of Glossina brevipalpis] [Translation elongation factor P @ Translation initiation factor 5A]

MIIYYSANQSKSGLKILLKNEPCLILENQFVKPGKGQPFNRIKIKKLISG

KIFTKIFKSNEKLIYADVLDVKVKSLYKDKKYWNFIKKENFEQFKISKKN

LGEKYKWIIEQLECIVTFWDENPINITLPRFVDIKVCNANFDIKGDTIKS

GNKYIILTTGAIIKAPIFIRSEEIVRVDTNLGEYVSRIK

>fig|283166.1.peg.1328 [Bartonella henselae str. Houston-1] [Translation elongation factor P @ Translation initiation factor 5A]

MKINGNEIRPGNVIEHQGSLWVAVKCNAVKPGKGGAFNQVEMKNLIDGTK

LNERFRAAETVERVRLEQKDFTFLYQQGDALIFMDSQSYEQLELQKDFVG

ERAAFLQDGMTVTVELYQEKPIGISLPDQVSVTIVEADPALKGQTVTASY

KPAILENGIRILVPPFINAGERIIVDTNELIYVRRANEKDK

>fig|406562.4.peg.2210 [Streptococcus pneumoniae SP19-BS75] [Translation elongation factor P @ Translation initiation factor 5A]

MIEASKLKAGMTFETADGKLIRVLEASHHKPGKGNTIMRMKLRDVRTGST

FDTSYRPEEKFEQAIIETVPAQYLYKMDDTAYFMNTETYDQYEIPVVNVE

NELLYILENSDVKIQFYGTEVIGVTVPTTVELTVAETQPSIKGATVTGSG

KPATMETGLVVNVPDFIEAGQKLVINTAEGTYVSRA

>fig|406560.4.peg.1730 [Streptococcus pneumoniae SP14-BS69] [Translation elongation factor P @ Translation initiation factor 5A]

MIEASKLKAGMTFETADGKLIRVLEASHHKPGKGNTIMRMKLRDVRTGST

FDTSYRPEEKFEQAIIETVPAQYLYKMDDTAYFMNTETYDQYEIPVVNVE

NELLYILENSDVKIQFYGTEVIGVTVPTTVELTVAETQPSIKGATVTGSG

KPATMETGLVVNVPDFIEAGQKLVINTAEGTYVSRA

>fig|63737.1.peg.5024 [Nostoc punctiforme PCC 73102] [Translation elongation factor P @ Translation initiation factor 5A]

MISSNDFRPGVSIVLDGSVWRVLEFLHVKPGKGSAFVRTKLKNVQSGSVM

EKTFRAGETVPQATLEKSTMQHTYKEGDEFVFMDMETYEEGRLTRTQIGD

RVKYLKEGMEAEVIKWGDQVLGVELPKSVVLEIVQTDPGLKGDTATGGSK

PATLETGAIVMVPLFISQGERIKVDTQEDKYISRE

>fig|195099.3.peg.381 [Campylobacter jejuni RM1221] [Translation elongation factor P @ Translation initiation factor 5A]

MASYSMGDLKKGLKIEIDGIPFKIVEYQHVKPGKGPAFVRIKIKSFIDGK

VLEKTFHAGDKCEAPNLEDKTMQYLYDDGENCQFMDTQTYEQVAISDDDV

GEAKKWMLDGMMVDVLFHNGKAIGVEVPQVVELKIIETAPNFKGDTQGSN

KKPATLETGAVVQIPFHVLEGEVIRVDTVRGEYIERANK

>fig|320389.3.peg.2809 [Burkholderia mallei NCTC 10247] [Translation elongation factor P @ Translation initiation factor 5A]

MKLKNLLNGGGQESVYKADDKFEVVVLDRKEVTYSYFADPMYVFMDADYN

QYEVEAEMMGDALNYLEDGMACEVVFYNEKAISVELPTILVREITYTEPA

VKGDTSSGKVLKNAKLATGFELQVPLFCNTGDKIEIDTRTNEYRSRA

>fig|95658.4.peg.621 [Ureaplasma urealyticum serovar 4] [Translation elongation factor P @ Translation initiation factor 5A]

MATIIQAKDLRAGHTFLYKGSIYQVIENSFNKTAMREGIVKCKVKNLRTG

AITVEVLTGEKVEQAIIEKSKMTFSYDDGSGYVFMDNETYEQISIPYNQL

SWEKNFIEEGTEVSVMRYDGELMGVSLPDQLVVTIVEAEEAVQGNSVQNA

TKRAWLASKWEFQVPQFIKSGEKVIINPSNGQYVGRAK

>fig|453364.8.peg.173 [Streptococcus pneumoniae CDC0288-04] [Translation elongation factor P @ Translation initiation factor 5A]

MIEASKLKAGMTFETADGKLIRVLEASHHKPGKGNTIMRMKLRDVRTGST

FDTSYRPEEKFEQAIIETVPAQYLYKMDDTAYFMNTETYDQYEIPVVNVE

NELLYILENSDVKIQFYGTEVIGVTVPTTVELTVAETQPSIKGATVTGSG

KPATMETGLVVNVPDFIEAGQKLVINTAEGTYVSRA

>fig|360109.7.peg.1380 [Campylobacter jejuni subsp. doylei 269.97] [Translation elongation factor P @ Translation initiation factor 5A]

MASYSMGDLKKGLKIEIDGIPFKIVEYQHVKPGKGPAFVRIKIKSFIDGK

VLEKTFHAGDKCEAPNLEDKTMQYLYDDGENCQFMDTQTYEQVAISDDDV

GEAKKWMLDGMMVDVLFHNGKAIGVEVPQVVELKIIETAPNFKGDTQGSN

KKPATLETGAVVQIPFHVLEGEVIRVDTVRGEYIERANK

>fig|458817.3.peg.2228 [Shewanella halifaxensis HAW-EB4] [Translation elongation factor P @ Translation initiation factor 5A]

MKTAHELRPGNVIMLDGSPWVVQKTETTRSGRNAAIVKLKLKHVLQDSST

ESTFKGEDKMEDIILERLDCTYSYFADPMYVFMDAEYNQYDVEAENLGDA

AAYIVDGMEENCQVTFYEGKAISVELPTSVVREVTYTEPSARGDTSGKVM

KPATITGGGTLSVADFVKTGDMIEIDTRTNEFKKRV

>fig|267409.1.peg.1251 [Listeria monocytogenes str. 1/2a F6854] [Translation elongation factor P @ Translation initiation factor 5A]

MISVNDFKTGLTIEVDNGIWRVLDFQHVKPGKGAAFVRSKLRNLRTGAIQ

EKTFRGGEKVAKAQIDNRKMAYLYADGTNHVFMDNESYEQIELPEDQIAH

ELKFLKENMEINIIMYQGETIGIDLPNTVELVVTATDPGIKGDTSSGGSK

PATLETGLVVQVPFFVNEGDKLVINTTEAAYVSRA

>fig|272621.3.peg.1254 [Lactobacillus acidophilus NCFM] [Translation elongation factor P @ Translation initiation factor 5A]

MTMISVNEFKNGLTIQYNNDLWRIVEFQHVKPGKGSAFVRSKLKSLRTGA

VQEYTFRSTAKVETADIQTKSMQYLYNDGSSYVFMDTSTYDQLAIPNEQI

GDEANYLLENMVVSVITHEGETLGIQLPNTVDLKVAKTEPNIKGDTSSGG

GKPATMETGLVVNVPFFINEGDVLTINTSDGTYVSRANK

>fig|349741.3.peg.936 [Akkermansia muciniphila ATCC BAA-835] [Translation elongation factor P @ Translation initiation factor 5A]

MAKVPVINLRKGHAVNYNNDVCVVVSMEHKCPPRMASYVQMSIRSISTKK

VYNLRLTSNESLEGVNLAREEYEFSYIDGMGYHFMNPDTYEDITVSPEIV

EPVKDYLMEGNIYILLFTDETVVSVELPAAITMEVAEAPEGVKGDSANNV

YKSATMTTGLVVQVPLFIKPGEKISVKTEDGSYLGRVN

>fig|246201.1.peg.260 [Streptococcus mitis NCTC 12261] [Translation elongation factor P @ Translation initiation factor 5A]

MIEASKLKAGMTFETADGKLIRVLEASHHKPGKGNTIMRMKLRDVRTGXT

X

>fig|246201.1.peg.259 [Streptococcus mitis NCTC 12261] [Translation elongation factor P @ Translation initiation factor 5A]

MYKMDETAYFMNTETYDQYEIPVVNVENELLYILENSDVKIQFYGTEVIG

VTVPTTVELTVAETQPSIKGATVTGSGKPATMETGLVVNVPDFIEAGQKL

IINTAEGTYVSRA

>fig|526226.5.peg.505 [Gordonia bronchialis DSM 43247] [Translation elongation factor P @ Translation initiation factor 5A]

MATTADFKNGLVLRMDDQLWQILEFQHVKPGKGPAFVRTKIKNVLSGKTV

DKTFNAGVKVETATVDRRDMQYLYNDGTDYVFMDQQDYEQFAIPPAIVGD

GARFLLENMTVQVSLNEGNPLFVELPVTVELVVEHTDPGLQGDRSTGGTK

PAKLETGAEIAVPLFINTGDKLKVDSRDGSYLGRVNS

>fig|138677.1.peg.893 [Chlamydophila pneumoniae J138] [Translation elongation factor P @ Translation initiation factor 5A]

MVRVSTSEFRVGLRIEIDGQPYLILQNDFVKPGKGQAFNRIKVKNFLTGR

VIERTYKSGESVETADIVERSMRLLYTDQEGATFMDDETFEQEVVFWEKL

ENIRQWLLEDTIYTLVLYNGDVVAVEPPIFMELSIAETAPGVRGDTASGR

VLKPAVTNTGAKIMVPIFIDEGELVKVDTRTGSYESRVSK

>fig|314260.3.peg.1578 [Parvularcula bermudensis HTCC2503] [Translation elongation factor P @ Translation initiation factor 5A]

MKLNGNEIRPGNVIQHQDTLWVAVKVNAVKPGKGPAYAQVELKNLLDGRK

LNERFRASETVERVRLEQKDHTFLYEEGEQLVFMDTESYEQINIEKEMVG

ERTAFLQDGMAVVVESHEGRPIGVSLPEQVTLEITETEPTVKGQTASSSY

KPAVLENGLRIMVPPHITPNTRVIVNTETLDYVKRAD

>fig|205922.3.peg.3606 [Pseudomonas fluorescens PfO-1] [Translation elongation factor P @ Translation initiation factor 5A]

MKTGKELKPGTVIRIDNDPWLVQKAEFTKSGRNSAIMKTKLKNLLTGYKT

ETVYGADDKLDDVILDRKEATLSFISGDSYTFMDTTDYTMYELNAEDIES

VLPFVEEGMTDVCEAVFFEGRLVSVELPTTIVRQVDYTEGSARGDTSGKV

MKPAKLKNGTELSVADFIEIGDMIEIDTREGGSYKGRAK

>fig|291272.3.peg.416 [Blochmannia pennsylvanicus str. BPEN] [Translation elongation factor P @ Translation initiation factor 5A]

MVLYNINEFRTGLKIIQNREPCVIISHESIKPGKGQAFSRVRFRQIISGK

ILEKTFKSGDFLESANIMEIRLVYVYHDNEFWYFMDEKNFEEIAVAAKII

GTNIKWITTQLHYIVTLWNKVPILVTPPDSIELKIIKITPIKKNSTGSSG

IKLATVSTGAIVKVPFFIQLGELIKINTRSGIYISRAK

>fig|52598.3.peg.2153 [Sulfitobacter sp. EE-36] [Translation elongation factor P @ Translation initiation factor 5A]

MPKINGNEIRPGNVLEHNGGLWAAVKVDHVKPGKGGAFAQVEMRNLRNGS

KLNERFRSADKVEKVRLEQKDQQFLYEDAGMLVLMDTETYEQVQLSAELL

GERRPFLQDGMMVVVEYHEEEALNASLPQKVTCKIVETEPVVKGQTAANS

FKPAVLDNGVKVMVPPFVGQDEEIIVNTETMEYSERA

>fig|273068.3.peg.1119 [Thermoanaerobacter tengcongensis MB4] [Translation elongation factor P @ Translation initiation factor 5A]

MIAAGDFRKGVTIEVDGQIFTVVDFMHVKPGKGAAFVRTKLKNIMTGAVI

ERTFSPTEKFEEAQIERREMQYLYNDGEFYYFMDTETYEQIPLSYDKVEE

AMKYIKENMIVTVKFYKGEAFSVEPPTFVELEVIDTEPGVRGDTVTGGSK

PATVETGAVIQVPLFINVGDKIKIDTRTGEYIERV

>fig|85963.1.peg.162 [Helicobacter pylori J99] [Translation elongation factor P @ Translation initiation factor 5A]

MAIGMSELKKGLKIELGGVPYRIVEYQHVKPGKGAAFVRAKIKSFLDGKV

IEKTFHAGDKCEEPNLVEKTMQYLYHDGDTYQFMDIESYEQIALNDSQVG

EASKWMLDGMQVQVLLHNDKAISVDVPQVVALKIVETAPNFKGDTSSASK

KPATLETGAVVQVPFHVLEGETIKVNTETEEYLEKVK

>fig|485915.4.peg.922 [Desulfohalobium retbaense DSM 5692] [Translation elongation factor P @ Translation initiation factor 5A]

MLSTKDIKTGLKIELEGEPFEILEFLHVKPGKGGAFVRTKLRNILTGAVK

DHTFRSGEKFAKPDLEKKSMQFLYRDGTDCVFMDMSSYEQVNVPEDSLAQ

QVGFLQDGQEVDVLTYQGQIIDIELPSSVILEVTETEPGLKGDTVSGATK

PATLETGLTVQVPLFIEQGEKVRVDTRSSEYIGRT

>fig|486.1.peg.327 [Neisseria lactamica ST-640] [Translation elongation factor P @ Translation initiation factor 5A]

MKTAQELRAGNVFMVGNDPMVVQKTEYIKGGRSSAKVSMKLKNLLTGAAS

ETIYKADDKFDVVILSRKNCTYSYFADPMYVFMDEEFNQYEIEADNIGDA

LKFIVDGMEDQCEVTFYEGNPISVELPTIIVREVEYTEPAVKGDTSGKVM

KTARLVGGTEIQVMSYIENGDKVEIDTRTGEFRKRA

>fig|453365.3.peg.2306 [Streptococcus pneumoniae CDC3059-06] [Translation elongation factor P @ Translation initiation factor 5A]

MIEASKLKAGMTFETADGKLIRVLEASHHKPGKGNTIMRMKLRDVRTGST

FDTSYRPEEKFEQAIIETVPAQYLYKMDDTAYFMNTETYDQYEIPVVNVE

NELLYILENSDVKIQFYGTEVIGVTVPTTVELTVAETQPSIKGATVTGSG

KPATMETGLVVNVPDFIEAGQKLVINTAEGTYVSRA

>fig|1806.1.peg.1223 [Mycobacterium microti OV254] [Translation elongation factor P @ Translation initiation factor 5A]

VATTADFKNGLVLVIDGQLWTITEFQHVKPGKGPAFVRTKLKNVLSGKVV

DKTFNAGVKVDTATVDRRDTTYLYRDGSDFVFMDSQDYEQHPLPEALVGD

AARFLLEGMPVQVAFHNGVPLYIELPVTVELEVTHTEPGLQGDRSSAGTK

PATLQTGAQINVPLFINTGDKLKVDSRDGSYLGRVNA

>fig|262723.3.peg.420 [Mycoplasma synoviae 53] [Translation elongation factor P @ Translation initiation factor 5A]

MIEVNKFKPGITFQDSGNIYVVLEATHSKQGRGQANVKAKAKNLRTGATT

ILSFTGGDKVEPAHIEKRKMNFLYSDDSNIYLMDSSDYSQIEIDLLKVEW

EMNFLKENSEVQVRMFQDEILDIELPANVDLKVTYAPDAVKGNTTTNPQK

KVTLETNFELETPMFIKENDVIIVSTETGKYVGKSK

>fig|334802.3.peg.808 [Burkholderia mallei FMH] [Translation elongation factor P @ Translation initiation factor 5A]

MKTAQELRVGNVVMIGNDAWVVSKTEYNKSGRNAAVVKMKLKNLLNGGGQ

ESVYKADDKFEVVVLDRKEVTYSYFADPMYVFMDADYNQYEVEAEMMGDA

LNYLEDGMACEVVFYNEKAISVELPTILVREITYTEPAVKGDTSSGKVLK

NAKLATGFELQVPLFCNTGDKIEIDTRTNEYRSRA

>fig|158878.1.peg.1528 [Staphylococcus aureus subsp. aureus Mu50] [Translation elongation factor P @ Translation initiation factor 5A]

MISVNDFKTGLTISVDNAIWKVIDFQHVKPGKGSAFVRSKLRNLRTGAIQ

EKTFRAGEKVEPAMIENRRMQYLYADGDNHVFMDNESFEQTELSSDYLKE

ELNYLKEGMEVQIQTYEGETIGVELPKTVELTVTETEPGIKGDTATGATK

SATVETGYTLNVPLFVNEGDVLIINTGDGSYISRG

>fig|418127.4.peg.1411 [Staphylococcus aureus subsp. aureus Mu3] [Translation elongation factor P @ Translation initiation factor 5A]

MISVNDFKTGLTISVDNAIWKVIDFQHVKPGKGSAFVRSKLRNLRTGAIQ

EKTFRAGEKVEPAMIENRRMQYLYADGDNHVFMDNESFEQTELSSDYLKE

ELNYLKEGMEVQIQTYEGETIGVELPKTVELTVTETEPGIKGDTATGATK

SATVETGYTLNVPLFVNEGDVLIINTGDGSYISRG

>fig|446470.4.peg.2326 [Stackebrandtia nassauensis DSM 44728] [Translation elongation factor P @ Translation initiation factor 5A]

MATTNDLKNGMVLSLDGVLWTVVKFQHVKPGKGGAFVRTTLKNVLSGKVV

DKTFNAGTKVETATVDKRTMQYLYAADDDFVFMDLDTYDQINVPSSTVGD

AKNYLLPEAEVTLGIHEGTPLYIELPASVELEVTHTEPGLQGDRSSGGTK

PATVETGANVAVPLFISTGEKIKVDTRDGKYLSRA

>fig|360106.5.peg.1199 [Campylobacter fetus subsp. fetus 82-40] [Translation elongation factor P @ Translation initiation factor 5A]

MASYSMGDLKKGLKIELDGVPYKIVEYQHVKPGKGAAFVRVKIKSFVNGK

VLEKTFHAGDKCESPNLVEKEMQYLYDDGEFCQFMDVESYEQVAISDEDI

GEAKKWMIDGMMVQILFHNGKAIGVEVPQVVELKIVETQPNFKGDTQGSN

KKPATLESGAVVQIPFHVLEGEVIRVDTVRGEYIERANK

>fig|243160.4.peg.1037 [Burkholderia mallei ATCC 23344] [Translation elongation factor P @ Translation initiation factor 5A]

MKTAQELRVGNVVMIGNDAWVVSKTEYNKSGRNAAVVKMKLKNLLNGGGQ

ESVYKADDKFEVVVLDRKEVTYSYFADPMYVFMDADYNQYEVEAEMMGDA

LNYLEDGMACEVVFYNEKAISVELPTILVREITYTEPAVKGDTSSGKVLK

NAKLATGFELQVPLFCNTGDKIEIDTRTNEYRSRA

>fig|272561.1.peg.769 [Chlamydia trachomatis D/UW-3/CX] [Translation elongation factor P @ Translation initiation factor 5A]

MVRVSTSEFRVGLRVKIDGQPYVILQNDFVKPGKGQAFNRIKVKNFLTGR

VIEKTFKSGESIETADVREQQMRLLYTDQEGATFMDDETFEQELIFWDKL

ENVRQWLLEDTIYTLVLYNGDVISVEPPIFMELTIAETAPGVRGDTASGR

VLKPATTNTGAKIMVPIFIEEGEVVKVDTRTGSYESRVSK

>fig|469371.4.peg.3045 [Thermobispora bispora DSM 43833] [Translation elongation factor P @ Translation initiation factor 5A]

MATTNDLKNGLVLKLEGGELWTVVEFQHVKPGKGGAFVRTKLKNVLSGKV

VDRTFNAGVKVDVANVDKREMQFSYLDGDEFVFMDTQTYDMVHVPRSVVG

DAANYMLENMTATVAFHEGTPLYVELPASVELVIAHTEPGVQGDRSTGGT

KPATLETGAEIKVPLFITTGERVKVDTRTGEYLGRA

>fig|272632.1.peg.426 [Mycoplasma mycoides subsp. mycoides SC str. PG1] [Translation elongation factor P @ Translation initiation factor 5A]

MSVNDLRPGTTFLYDGNIYLVLEQAFSKTGRQQGKVTVKAKNMRTGARVE

LTFTGGEKVDKAMIERKEMQYLYNDGNDAYLMNTETYEQVSIPMTRLEWE

KNFLVDGLMINMTEFENEVLGIDLPVKVELTVVEAEAAVKGDTTSGAQKK

AILETGLEIMVPLFVNQGTKIIVSSADGKYVGRA

>fig|171101.1.peg.392 [Streptococcus pneumoniae R6] [Translation elongation factor P @ Translation initiation factor 5A]

MIEASKLKAGMTFETADGKLIRVLEASHHKPGKGNTIMRMKLRDVRTGST

FDTSYRPEEKFEQAIIETVPAQYLYKMDDTAYFMNTETYDQYEIPVVNVE

NELLYILENSDVKIQFYGTEVIGVTVPTTVELTVAETQPSIKGATVTGSG

KPATMETGLVVNVPDFIEAGQKLVINTAEGTYVSRA

>fig|43989.3.peg.1957 [Cyanothece sp. ATCC 51142] [Translation elongation factor P @ Translation initiation factor 5A]

MISSNDFRTGTSIELDGSVWRVVEFLHVKPGKGSAFVRTKLKNAQTGSVV

ERTFRAGETVPQATLEKRTMQHTYKEGEQYVFMDMETYEEVRLSPEQMGT

TVNYIKEEMEADVLFWNDTVLEVQLPTSVILEVTDTDPGVKGDTATGGTK

PAIVETGAQVMVPLFISIGEKIKVDTRDGSYLGRET

>fig|203907.1.peg.68 [Blochmannia floridanus] [Translation elongation factor P @ Translation initiation factor 5A]

MLYNINELKVGLKVIQNKEPYVIIKNECIKPGKGQSFNRVRFKQIKSGKI

LEKTLKPGDLVESANIVETELIYVYRDRDLWFFMNRDSFDQISVHFDILG

KSVKWMVEQLVYVVVFWDNNPILVIPPEFIKLKIIKTNLITKNISTASGN

KLAVVSTGAVVKVPFFIQSGELIKVNTHSGSYISRIK

>fig|257313.1.peg.1756 [Bordetella pertussis Tohama I] [Translation elongation factor P @ Translation initiation factor 5A]

MKTAQELRVGNVIMVGKDPLVVQKTEYNKSGRNAAVVKLKFKNLLTGSGS

ESVYKADEKFDVVVLERKECTYSYFGDPMYVFMDEEYNQYEIEADSMGDA

LNYLEEAMPVEVVFYDGRAISVELPTILVREITYTEPAVRGDTSGKVLKP

AKINTGFELSVPLFCAIGDKIEIDTRTNEYRSRVN

>fig|95666.5.peg.562 [Ureaplasma urealyticum serovar 12] [Translation elongation factor P @ Translation initiation factor 5A]

MATIIQAKDLRAGHTFLYKGSIYQVIENSFNKTAMREGIVKCKVKNLRTG

AITVEVLTGEKVEQAIIEKSKMTFSYDDGSGYVFMDNETYEQISIPYNQL

SWEKNFIEEGTEVSVMRYDGELMGVSLPDQLVVTIVEAEEAVQGNSVQNA

TKRAWLASKWEFQVPQFIKSGEKVIINPSNGQYVGRAK

>fig|100226.1.peg.1458 [Streptomyces coelicolor A3(2)] [Translation elongation factor P @ Translation initiation factor 5A]

MASTNDLKNGLVLKLEGGQLWSVVEFQHVKPGKGPAFVRTKLKNVLSGKV

VDKTFNAGVKVETATVDKRDMQFSYMDGDYFVFMDMETYDQLMIDRKVVG

DAANFLVEGFEATVAQHEGEVLFVELPAAVELTIQETEPGVQGDRSTGGT

KPATLETGHQINVPLFITTGEKIKVDTRTSDYLGRVNS

>fig|314267.3.peg.1439 [Sulfitobacter sp. NAS-14.1] [Translation elongation factor P @ Translation initiation factor 5A]

MPKINGNEIRPGNVLEHNGGLWAAVKVDHVKPGKGGAFAQVEMRNLRNGS

KLNERFRSADKVEKVRLEQKDQQFLYEDAGMLVLMDTETYEQVQLSAELL

GERRPFLQDGMMVVVEYHEEEALNASLPQKVTCKIVETEPVVKGQTAANS

FKPAVLDNGVKVMVPPFVGQDEEIIVNTETMEYSERA

>fig|318161.14.peg.1946 [Shewanella denitrificans OS217] [Translation elongation factor P @ Translation initiation factor 5A]

MKTAHEIRPGNVIMLDGSPWVVQKTETTRSGRNAAIVKLKLKHVLLDSGT

EQTFKGEDKMDVIVLERLDCTYSYFADPMYVFMDADYNQYDVEADNLGDA

AAYIIDGMEETCQVTFYEGKAISVELPTHIVREVIYTEPSARGDTSGKVM

KPATITGGGTVTVADFVKVGDKIEIDTRTGEFKKRA

>fig|395495.3.peg.1861 [Leptothrix cholodni SP-6] [Translation elongation factor P @ Translation initiation factor 5A]

MKIAQEIRAGNVIMHGKDPMVVLKTEYSRGGRNSATVRMKLKSLLSNSGT

EVVFKADDKMDQIILDKKECTYSYFADPMYAFMDADFNQFEVEAENMGDA

ISYLEDGMAVEVVFYDGKAISVELPTSLVREVTWTEPAVKGDTSGKVLKP

AKLATGFDIQVPIFVAQGDKIEIDTRTHEYRKRV

>fig|227941.1.peg.861 [Chlamydophila caviae GPIC] [Translation elongation factor P @ Translation initiation factor 5A]

MVRVSTSEFRVGLRIEIDGQPYLILQNDFVKPGKGQAFNRIKVKNFLTGR

VIERTFKSGESVETADVREQQMRFLYSDQEGATFMDDETFEQEMIFWDKI

ENIRQWLLEDTIYTLVLYNGNVIGVEPPIFMELTIAETAPGVRGDTASGR

VLKPAVTNTGAKIMVPIFIEEGEVVKIDTRTGSYESRVSK

>fig|390236.5.peg.277 [Borrelia afzelii PKo] [Translation elongation factor P @ Translation initiation factor 5A]

MAVVKSSEIEKGSFLLIKGAPHIVLEREFSKTGRGGAIVRLKLKNLKNKF

VIRETLKGADTAEAIEIYEASVQYLYKDKDVLVFMDLETYDQISLDLKEN

ANFQDKVLFLQESEIYSLIMFDNVVIDIKLAPKIAFEVVEVEAAVKGDTV

TNAMKNITLNTGLIIKAPLFINVGDKVLVNSETKEYAERIKN

>fig|210007.1.peg.1676 [Streptococcus mutans UA159] [Translation elongation factor P @ Translation initiation factor 5A]

MIEASKLKAGMTFETTDGKLIRVLEASHHKPGKGNTVMRMKLRDVRTGST

FDTTYRPEEKFEQAIIETRPAQYLYQMDDTAYFMDTENYEQYEIPIVNIE

NELKFILENSEVKIQFYGSEVIGVTIPTTVELVVTDTQPSIKGATVTGSG

KPATLETGLVVNVPDFIEVGQKLVINTAEGTYVSRA

>fig|280477.3.peg.1192 [Bacillus anthracis str. Australia 94] [Translation elongation factor P @ Translation initiation factor 5A]

MISVNDFRTGLTIAVDNGLWQVLDFQHVKPGKGAAFVRSKLRNLRTGSVQ

EKTFRAGEKVEKAHIENRRMQYLYASGEAHVFMDNGTYEQIELGEKQIER

ELKFLKENMEVSIMTYQGEVLGVELPNTVELQVTETEPGIKGDTASNVTK

PATLETGLVVQVPIFINEGEMLIINTGEGKYVSRA

>fig|321955.3.peg.1138 [Brevibacterium linens BL2] [Translation elongation factor P @ Translation initiation factor 5A]

MAKTDTKKDGLVASTNDLKNGLVLNIDKQLWQVLEFQHVKPGKGPAFVRT

KLKNVISGKIIDKTFNAGTKVETANVDRRDMQYLYHDGTDYVFMDAEDYD

QVSVSPELVGDAANYMLENQNLQISFHEGTALSVELPPSVELLITHTEPG

LQGDRSTGGSKPATLETGYDIQVPLFLEEGTKVKVDTRNGDYLGRVK

>fig|401650.3.peg.950 [Listeria monocytogenes Aureli 1997] [Translation elongation factor P @ Translation initiation factor 5A]

MISVNDFKTGLTIEVDNGIWRVLDFQHVKPGKGAAFVRSKLRNLRTGAIQ

EKTFRGGEKVAKAQIDNRKMAYLYADGTNHVFMDNESYEQIELPEDQIAH

ELKFLKENMEINIIMYQGETIGIDLPNTVELVVTATDPGIKGDTSSGGSK

PATLETGLVVQVPFFVNEGDKLVINTTEAAYVSRA

>fig|203120.1.peg.1026 [Leuconostoc mesenteroides subsp. mesenteroides ATCC 8293] [Translation elongation factor P @ Translation initiation factor 5A]

MAIGMNDLKTGLTIEYSNSIWRVLDFQHVKPGKGGAFVRSKLKNLRTGAV

NEVTFRPGDKFEQADITTRPMSYLYAENDGRVFMDVESYDQINIPDEYLK

NALKFLLEGMEVKITMFGNEVLGAELPSTVQMKVTETQPGIKGATATGSG

KPATVETGATITVPDFVNEGETIIINTEDGSYKGRA

>fig|351607.5.peg.1301 [Acidothermus cellulolyticus 11B] [Translation elongation factor P @ Translation initiation factor 5A]

MATTNDLKNGMTLDLDGELWNVVEFQHVKPGKGPAFVRTKLKHVLTGKVV

DKTFNAGVKVDVATVDKRTMQYLYREGDSFVFMDTETYDQYHIPAQTVGD

AANFLLENAEAVVALHEGTPLYVELPAAVELTITYTEPGVQGDRSTGGTK

PATLETGAQIQVPLFITTGEKVKVDTRTGEYLGRANS

>fig|267747.1.peg.1171 [Propionibacterium acnes KPA171202] [Translation elongation factor P @ Translation initiation factor 5A]

MTNISPGETVRCLRQRLPEQRAALGFRYTYRGWARATSCPYPSISPSDDS

ERARPVVISTNDIKNGTVLNLDGQLWTVIWFQHHKPGKGNTVVRTKLKHV

LSGKVVDRTFNSDVKIESAEVDRRDMQYLYQDGDTYIFMDESTYEQLPIP

TEVVGDAKDFMLENQTATVALHEGNPLYIDLPASVELEITYTEPGLQGDR

STGGTKPATLETGREIQVPLFITTGEKVKVDTRSGDYLGRISEK

>fig|251221.1.peg.1285 [Gloeobacter violaceus PCC 7421] [Translation elongation factor P @ Translation initiation factor 5A]

MISSNDFRTGTTIELDGQVWRVIEFLHVKPGKGSAFVRTKLKNVMTGNVN

ERTFRAGETLPQAVVEKRDMQFVYPQGDNEYVFMDMESYEQEALTRETLG

DGAKYLKEGMSVSILKWQERVIGVDLPNTVVLQVVETDPGVKGDTAQGGT

KPAKVETGAEVMVPLFITIGEKIKIDTRDNSYLGREN

>fig|262719.3.peg.536 [Mycoplasma hyopneumoniae J] [Translation elongation factor P @ Translation initiation factor 5A]

MINVNEFRPGITFEFENEIYVVISAQHSKQGRGQANVKTKVKNLRTGAIT

IKTFSGGERVEKAHIEKISMSFLYNDGASIVLMDDSTYEQVAIENTKITW

ELNFLTEGIKVKLRKFNNEILDIELPAKIELKITSTFDAVRGNTTTNPTK

RATLETGYEIDVPLFIKEGESVIVSTEDGKYVSRG

>fig|313589.3.peg.1697 [Janibacter sp. HTCC2649] [Translation elongation factor P @ Translation initiation factor 5A]

MASTNELKNGLVLNLEGQLWTVIEFQHVKPGKGPAFVRTKLKHVLSGKVV

DKTFNAGTKVDTANVDKRNMTYLYHDGTDYVFMDGDTFDQITVTPTVVGD

AAKYMLENQDAIVARHDGVVLFIELPASVVLEIQYTEPGLQGDRSTGGTK

PATLETGAEIGVPLFLNTGDKVKVDTRDGSYLGRVN

>fig|487214.3.peg.531 [Streptococcus pneumoniae Hungary19A-6] [Translation elongation factor P @ Translation initiation factor 5A]

MIEASKLKAGMTFETADGKLIRVLEASHHKPGKGNTIMRMKLRDVRTGST

FDTSYRPEEKFEQAIIETVPAQYLYKMDDTAYFMNTETYDQYEIPVVNVE

NELLYILENSDVKIQFYGTEVIGVTVPTTVELTVAETQPSIKGATVTGSG

KPATMETGLVVNVPDFIEAGQKLVINTAEGTYVSRA

>fig|413999.4.peg.1809 [Clostridium botulinum A str. ATCC 3502] [Translation elongation factor P @ Translation initiation factor 5A]

MISAGDLRKGTTFEQDGQVYVVVEFLHVKPGKGAAFVRTKLKNAITGAVT

ETTFNPTAKLQEAVIERKEMQYLYTDGELYYFMDQETFEQIPLNYDKVEE

AIKFLKENMFATIKFFKGEAFSVEAPNFVELLISHTEPGAKGNTTSNVMK

PATLETGATIQVPLFVNEGETIRVDTRTGEYMERV

>fig|254945.3.peg.179 [Ehrlichia ruminantium str. Welgevonden] [Translation elongation factor P @ Translation initiation factor 5A]

MAERGSDIRPGYVLEHNNALYLVVKIMHTQPGKGGAYIQAEMKNLKTGAK

QYERFRADGDIKRAILDEADYQYIYGDDSMLTVMHLGNYEQITIKKDILG

DKSIYLKDNMVITLLSYNGEIISAKVPDYVTLQVIETEAVIKGQTVSSSS

YKVAMLENNQRINVPTFIKSGDKIVVYTPDDSYYERAKE

>fig|313590.3.peg.1313 [Cellulophaga sp. MED134] [Translation elongation factor P @ Translation initiation factor 5A]

MATTSDIRNGLCLHYNNDIYKIVEFLHVKPGKGPAFVRTKMKSVTTGKVL

DNTFSAGHKIDEVRVETHKFQFLYNDGEGYHFMNTEDYTQIALQETALDR

SDLLKEGEVVTVLINSEDNSPLSVDMPASVVLEVTHTEPGVKGNTATNAT

KPATVETGAEVNVPLFINEGDKIKIETVKGTYKERVKE

>fig|441771.4.peg.1714 [Clostridium botulinum A str. Hall] [Translation elongation factor P @ Translation initiation factor 5A]

MISAGDLRKGTTFEQDGQVYVVVEFLHVKPGKGAAFVRTKLKNAITGAVT

ETTFNPTAKLQEAVIERKEMQYLYTDGELYYFMDQETFEQIPLNYDKVEE

AIKFLKENMFATIKFFKGEAFSVEAPNFVELLISHTEPGAKGNTTSNVMK

PATLETGATIQVPLFVNEGETIRVDTRTGEYMERV

>fig|103690.1.peg.5365 [Nostoc sp. PCC 7120] [Translation elongation factor P @ Translation initiation factor 5A]

MISSNDFRPGVSIVLDGSVWRVIDFLHVKPGKGSAFVRTTLKNVQSGKVL

EKTFRAGETVPQATLEKITMQHTYKEGDEFVFMDMESYEEGRLSAAQIGD

RVKYLKEGMEVNVIRWGEQVLEVELANSVVLEVIQTDPGVKGDTATGGTK

PAIVETGATVMVPLFISQGERIKIDTRDDKYLGRE

>fig|435590.6.peg.506 [Bacteroides vulgatus ATCC 8482] [Translation elongation factor P @ Translation initiation factor 5A]

MINAQDIKIGTAIRMDGKLYFCIDFLHVKPGKGNTFMRTKLKDVVNGYVL

ERRFNIGEKLEDVRVERRPHQYLYMEGADYIFMNQETFDQIPIAHDLING

VDFLLEGMVVDVVSDASTETVLFADVPVKVQMKITYTEPGLKGDTATNTL

KPATVESGATVRVPLFINEGETIEIDTRDGSYVGRVKA

>fig|76869.3.peg.1424 [Pseudomonas putida GB-1] [Translation elongation factor P @ Translation initiation factor 5A]

MKTGKELKPGTVLRIDNDPWLVQKAEFTKSGRNSAIMKTKLKNLLTGYKT

ETVYGADDKLDDVILDRKEATLSFISGDSYTFMDTTDYTMYELNAEDIDA

VLPYIEEGMEDICEAVFFEGRLVSVELPTTISRQVVYTENAARGDTSGKV

MKPAKLKNGTEIQVADFIQIDEWIDIDTRDNSFKGRSKK

>fig|519441.4.peg.857 [Streptobacillus moniliformis DSM 12112] [Translation elongation factor P @ Translation initiation factor 5A]

MKAAMELRQGNVYVKDNTPYLILKADRHQSTSGKKARAAEMKFKIKDLIS

GKVQEITVLSTDMMNDIILDRAQMQYLYQMDGEYYFMNQETFEQMTLTED

DLGDAVDFLVDEMVIQVLLYEERAVGVELPNTVVREITYTEPGLKGDTIG

RATKPATIETGYQLQVPLFCNIGDKIRIDTRTGEYMERAN

>fig|335541.4.peg.521 [Syntrophomonas wolfei subsp. wolfei str. Goettingen] [Translation elongation factor P @ Translation initiation factor 5A]

MISVNDFKTGVTIELEGQAFQVVEFMHVKPGKGSAFVRAKLKNVKTGGTV

EKTFRGGEKVPRAHLDKREMQYLYNDGEGYVCMDTENYEQISISKESIGE

GAKWLMENMILGVLFFQGNIIGVDLPNFVEMLVVDTEPGVKGDTATGAVK

NATLESGAVVQVPLFVNTGDRLRIDIRTGEYMERV

>fig|487.2.peg.2295 [Neisseria meningitidis FAM18] [Translation elongation factor P @ Translation initiation factor 5A]

MKTAQELRAGNVFMVGNDPMVVQKTEYIKGGRSSAKVSMKLKNLLTGAAS

ETIYKADDKFDVVILSRKNCTYSYFADPMYVFMDEEFNQYEIEADNIGDA

LKFIVDGMEDQCEVTFYEGNPISVELPTIIVREVEYTEPAVKGDTSGKVM

KTARLVGGTEIQVMSYIENGDKIEIDTRTGEFRKRA

>fig|393123.3.peg.989 [Listeria monocytogenes FSL N1-017] [Translation elongation factor P @ Translation initiation factor 5A]

MISVNDFKTGLTIEVDNGIWRVLDFQHVKPGKGAAFVRSKLRNLRTGAIQ

EKTFRGGEKVAKAQIDNRKMAYLYADGTNHVFMDNESYEQIELPEDQIAH

ELKFLKENMEINIIMYQGETIGIDLPNTVELVVTATDPGIKGDTSSGGSK

PATLETGLVVQVPFFVNEGDKLVINTTEAAYVSRA

>fig|302409.3.peg.520 [Ehrlichia ruminantium str. Gardel] [Translation elongation factor P @ Translation initiation factor 5A]

MAERGSDIRPGYVLEHNNALYLVVKIMHTQPGKGGAYIQAEMKNLKTGAK

QYERFRADGDIKRAILDEADYQYIYGDDSMLTVMHLGNYEQITIKKDILG

DKSIYLKDNMVITLLSYNGEIISAKVPDYVTLQVIETEAVIKGQTVSSSS

YKVAMLENNQRINVPTFIKSGDKIVVYTPDDSYYERAKE

>fig|222523.1.peg.4235 [Bacillus cereus ATCC 10987] [Translation elongation factor P @ Translation initiation factor 5A]

MISVNDFRTGLTIAVDNGLWQVLDFQHVKPGKGAAFVRSKLRNLRTGSVQ

EKTFRAGEKVEKAHIENRRMQYLYASGESHVFMDNGTYEQIELGEKQIER

ELKFLKENMEVSIMTYQGEVLGVELPNTVELQVTETEPGIKGDTASNVTK

PATLETGLVVQVPIFINEGETLIINTGEGKYVSRA

>fig|360095.3.peg.1142 [Bartonella bacilliformis KC583] [Translation elongation factor P @ Translation initiation factor 5A]

MKINGNEIRPGNVIEHQGSLWVAVKCNAVKPGKGGAFNQVELKNVIDGTK

LNERFRAAETVEKVRLEQKDFTFLYQQGEALVFMDTESYEQLELQRDFVG

DRAAFLQDGMTVTVELHEEKPLGISLPDQVTVTIAEADPAIKGQTVTSSY

KPAILENGIRILVPPFVQAGERIIVDTNELTYIRRVSEKG

>fig|293653.3.peg.1859 [Streptococcus pyogenes MGAS5005] [Translation elongation factor P @ Translation initiation factor 5A]

MIEASKLKAGMTFEAEGKLIRVLEASHHKPGKGNTIMRMKLRDVRTGSTF

DTTYRPDEKFEQAIIETVPAQYLYKMDDTAYFMNTDTYDQYEIPVANVEQ

ELLYILENSDVKIQFYGSEVIGVTVPTTVELTVAETQPSIKGATVTGSGK

PATLETGLVVNVPDFIEAGQKLIINTAEGTYVSRA

>fig|240292.3.peg.194 [Anabaena variabilis ATCC 29413] [Translation elongation factor P @ Translation initiation factor 5A]

MISSNDFRPGVSIVLDGSVWRVIDFLHVKPGKGSAFVRTTLKNVQSGKVL

EKTFRAGETVPQATLEKITMQHTYKEGDEFVFMDMESYEEGRLSASQIGD

RVKYLKEGMEVNVIRWGEQVLEVELANSVVLEVIQTDPGVKGDTATGGTK

PAIVETGATVMVPLFISQGERIKIDTRDDKYLGRE

>fig|349101.4.peg.1917 [Rhodobacter sphaeroides ATCC 17029] [Translation elongation factor P @ Translation initiation factor 5A]

MPKINGNEIKPGFILEHDGGLWAAVKVNHVKPGKGGAFAQVELKNLRDGR

KLNERFRSEDKVERTELENKDQQFLYESDGRLIFMDAESFEQVEIDAELL

GERRPFLQDGMVATVNYFGDEPLNVTLPAKVRCRVVETEPVVKGQTAANS

YKPAILDNGMRIMVPPFIGPDEEILVHTEFMEYSERV

>fig|203120.4.peg.1585 [Leuconostoc mesenteroides subsp. mesenteroides ATCC 8293] [Translation elongation factor P @ Translation initiation factor 5A]

MAIGMNDLKTGLTIEYSNSIWRVLDFQHVKPGKGGAFVRSKLKNLRTGAV

NEVTFRPGDKFEQADITTRPMSYLYAENDGRVFMDVESYDQINIPDEYLK

NALKFLLEGMEVKITMFGNEVLGAELPSTVQMKVTETQPGIKGATATGSG

KPATVETGATITVPDFVNEGETIIINTEDGSYKGRA

>fig|441770.4.peg.1711 [Clostridium botulinum A str. ATCC 19397] [Translation elongation factor P @ Translation initiation factor 5A]

MISAGDLRKGTTFEQDGQVYVVVEFLHVKPGKGAAFVRTKLKNAITGAVT

ETTFNPTAKLQEAVIERKEMQYLYTDGELYYFMDQETFEQIPLNYDKVEE

AIKFLKENMFATIKFFKGEAFSVEAPNFVELLISHTEPGAKGNTTSNVMK

PATLETGATIQVPLFVNEGETIRVDTRTGEYMERV

>fig|262543.1.peg.192 [Exiguobacterium sp. 255-15] [Translation elongation factor P @ Translation initiation factor 5A]

MVSVNDLKTGLTIKTSDGMIWQVLEFQHVKPGKGAAFVRTKMRNIRNGNI

QEMTFRGGERVERAHIERNKMQYLYPMGETYVFMDTESYEQLELTTAQVE

AALPFLLENMEVQIAIYNGEVLGIELPNTIVMTIVEAEPGVKGDTASNVK

KNATVETGHIIHVPLFIEAGEKVTVDTRTGDFTGRYNG

>fig|84588.1.peg.30 [Synechococcus sp. WH 8102] [Translation elongation factor P @ Translation initiation factor 5A]

MISSNDFRTGTTIEIDGAVWRVVEFLHVKPGKGSAFVRSKLKAVKTGNVV

EKTFRAGEMLPQALLEKASLQHTYMEGEDYVFMDMSTYEETRLSADQIGE

SRKYLKEGMEVNVVSWNGSPLEVELPNSVVLEITETDPGVKGDTATGGTK

PAILETGAQVMVPLFLSIGEKIKVDTRSDSYLGRENG

>fig|93059.3.peg.1463 [Prochlorococcus marinus str. MIT 9211] [Translation elongation factor P @ Translation initiation factor 5A]

MISSNDFRTGTTIELDGAVWRVIEFLHVKPGKGSAFVRTKLKAVQSGSVV

EKTFRAGEMVPQALLEKTTLQHTYMDSGDYVFMDMSSYEETRLTAAQIGE

SRKYLTEGMEVNVVSWNERPLEVELPNSVVLTVKETDPGVKGDTATGGTK

PAILETGAQVMVPLFISVGEKIKVDTRNDSYLGREN

>fig|216591.1.peg.4149 [Burkholderia cenocepacia J2315] [Translation elongation factor P @ Translation initiation factor 5A]

MKTAQELRVGNVVQIGSDAWVIAKTEYNKSGRNAAVVKMKMKNLLSNAGQ

ESVYKADDKFDVVVLDRKEVTYSYFADPMYVFMDADYNQYEVEAEMMGEA

LNYLEDGMACEVVFYNEKAISVELPTILVREITYTEPAVKGDTSSGKVLK

NAKLATGFELQVPLFCNTGDKIEIDTRTNEYRSRA

>fig|212042.5.peg.538 [Anaplasma phagocytophilum HZ] [Translation elongation factor P @ Translation initiation factor 5A]

MEVAMAERGNDIRPGQVLDHNGALYLVVKTMHTQPGKGGAYIQAELKNLK

TGAKYQERFRADGYVNRAIIEEVSFQYIFGDSSSLTLMNTENYEQISIPV

ELLGDKSVYLQENMILTLLFHKGQVISAKVPDYVVLEVVEAESVIKGQTA

SSSYKSAVLENGRRVSVPPFIKVGEKIVIYTPDDTYYERAKD

>fig|521095.5.peg.999 [Atopobium parvulum DSM 20469] [Translation elongation factor P @ Translation initiation factor 5A]

MATISTADFKNGLGLKINDKYYTIVEFQHVKPGKGGAFVRYKIRDLRSGR

VIDQTCNAGTKFENVLLLTKEMQYLYNDGESFYFMDNETYDQVAVPADFI

GEKSVWFKENDNAQLLYADTELLGVEPPMFIEAEITETDPGFKGDTVQGG

TKPATIETGATLQVPMYLNQGERIKVDTRDGKFVSRV

>fig|280355.3.peg.939 [Bacillus anthracis str. A1055] [Translation elongation factor P @ Translation initiation factor 5A]

MISVNDFRTGLTIAVDNGLWQVLDFQHVKPGKGAAFVRSKLRNLRTGSVQ

EKTFRAGEKVEKAHIENRRMQYLYASGEAHVFMDNGTYEQIELGEKQIER

ELKFLKENMEVSIMTYQGEVLGVELPNTVELQVTETEPGIKGDTASNVTK

PATLETGLVVQVPIFINEGEMLIINTGEGKYVSRA

>fig|264732.9.peg.1543 [Moorella thermoacetica ATCC 39073] [Translation elongation factor P @ Translation initiation factor 5A]

MISTNDFRTGLTIEVDGDVYTVVEFMHVKPGKGSAFVRTKLKNRRTGAVI

ERTFRAGEKVNRAHVERREMQYLYNDGDNYYFMDTETFEQLSLRKEQLED

AIKYLKDNMNIFVLTYNGETIGIELPNSVELKVVETEPGIKGDTATGGTK

NAVLETGAVIQVPLFIETGDVVRIDTRTGEYIERA

>fig|220664.3.peg.6076 [Pseudomonas fluorescens Pf-5] [Translation elongation factor P @ Translation initiation factor 5A]

MKTGKELKPGTVIRIDNDPWLVQKAEFTKSGRNSAIMKTKLKNLLTGYKT

ETVYGADDKLDDVILDRKEATLSFISGDTYTFMDTTDYTMYELNAEDIEA

VLPFIEEGMTDVCEAVFFEDRLVSVDLPTTIVRQVDYTEGSARGDTSGKV

MKPAKLKNGTELSVADFIEIGDMIEIDTREGGSYKGRAKV

>fig|265669.1.peg.1357 [Listeria monocytogenes str. 4b F2365] [Translation elongation factor P @ Translation initiation factor 5A]

MISVNDFKTGLTIEVDNGIWRVLDFQHVKPGKGAAFVRSKLRNLRTGAIQ

EKTFRGGEKVAKAQIDNRKMAYLYADGTNHVFMDNESYEQIELPEDQIAH

ELKFLKENMEINIIMYQGETIGIDLPNTVELVVTATDPGIKGDTSSGGSK

PATLETGLVVQVPFFVNEGDKLVINTTEAAYVSRA

>fig|226900.1.peg.4023 [Bacillus cereus ATCC 14579] [Translation elongation factor P @ Translation initiation factor 5A]

MISVNDFRTGLTISVDNALWQVLDFQHVKPGKGAAFVRSKLRNLRTGSVQ

EKTFRAGEKVEKAHIENRRMQYLYASGEAHVFMDNGTYEQIELGEKQIER

ELKFLKENMEVAIMTYQGEVLGVELPNTVELQVTETEPGIKGDTASNVTK

PATLETGLVVQVPIFINEGEMLIINTGEGKYVSRA

>fig|156586.3.peg.34 [Flavobacteria sp. BBFL7] [Translation elongation factor P @ Translation initiation factor 5A]

MASTSDIRKGLCIRYNHDIYKIVEFLHVKPGKGPAFVRTKLKSVTNGKVI

DNTFSAGHKIEDIRVETQSFQFLYQDPEGYHFMNTDTYEQIQLQESVLDA

PGLLKEGEVVKIQINTEDDSPLSVDMPQYVVLEVTATEPGLKGNTATNAT

KPATVETGAEVNVPLFINEGDKIRIDTDKGAYHERMK

>fig|1680.3.peg.793 [Bifidobacterium adolescentis] [Translation elongation factor P @ Translation initiation factor 5A]

MAQTTNDIKNGSVLNLDGQLWTVIKFQHVKPGKGPAFVRTTIKNVLSGKI

VDKTFNAGMKMEFETVDNRNLQYSYEDGDNFVFMDMTTYDQIFIPKTLVG

DQAKFLLEGTDCVVSFHDGTPLSVELPASVVLTVTHTEPGLQGNRSNAGT

KPATVETGAEIQVPLFIGEGEKVKVNTTDGSYLGREN

>fig|237727.3.peg.930 [Erythrobacter sp. NAP1] [Translation elongation factor P @ Translation initiation factor 5A]

MISGSACPYALAPPTYKRVIPMKISGVDIRPGNILEYEGGIWKVAKIQHT

QPGKGGAYMQVEMKNLQDGRKTNVRFRSADTVEKVRLDTAEYQFLYEDGD

MLVFMDTNTYEQINLPNDLLGDARPFLQDGMQVQLELWEEKPISVQLPQQ

IEAEIVEADAVVKGQTASSSYKPAVLDNGVRIMVPPHIESGTRIVVDVYE

QTYVGKAG

>fig|169963.1.peg.1347 [Listeria monocytogenes EGD-e] [Translation elongation factor P @ Translation initiation factor 5A]

MISVNDFKTGLTIEVDNGIWRVLDFQHVKPGKGAAFVRSKLRNLRTGAIQ

EKTFRGGEKVAKAQIDNRKMAYLYADGTNHVFMDNESYEQIELPEDQIAH

ELKFLKENMEINIIMYQGETIGIDLPNTVELVVTATDPGIKGDTSSGGSK

PATLETGLVVQVPFFVNEGDKLVINTTEAAYVSRA

>fig|208963.3.peg.419 [Pseudomonas aeruginosa UCBPP-PA14] [Translation elongation factor P @ Translation initiation factor 5A]

MKTAQEFRAGQVANINGAPWVIQKAEFNKSGRNAAVVKMKLKNLLTGAGT

ETVFKADDKLEPIILDRKEVTYSYFADPLYVFMDSEFNQYEIEKDDLEGV

LTFIEDGMTDICEAVFYNDKVISVELPTTIVRQIAYTEPAVRGDTSGKVM

KTARLNNGAELQVSAFCEIGDSIEIDTRTGEYKSRVKA

>fig|246200.3.peg.1658 [Silicibacter pomeroyi DSS-3] [Translation elongation factor P @ Translation initiation factor 5A]

MPKINGNEIRPGNVLEHNGGLWAAVKVDHVKPGKGGAFAQVELRNLRNGS

KLNERFRSADKVERVRLEQKDQQFLYESDGMLVFMDAETYEQIELPADLL

GERRPFLQDGMTILVEFYESEALNATLPQKVTCKIVETEPVVKGQTAANS

FKPAVLDNGVKVMVPPFVGQDEMIVVNTETMEYSERA

>fig|313596.3.peg.3112 [Robiginitalea biformata HTCC2501] [Translation elongation factor P @ Translation initiation factor 5A]

MANTSDIRKGLCIRHNNDIYKIVEFLHVKPGKGPAFVRTKLKSITTGKVI

DNTFSAGHKIDDVRVETRSYQFLYSDGQTYHFMNTDDYNQIALQESALDT

PELLKEGEVVTIMFNTEDSMPLSVDMPASVVLEVTEAEPGVKGNTATNAT

KVAVVETGARINVPLFINEGDKIKVDTSNGSYMERAKDS

>fig|467705.8.peg.1807 [Streptococcus gordonii str. Challis substr. CH1] [Translation elongation factor P @ Translation initiation factor 5A]

MIEASKLKAGMTFETADGKLIRVLEASHHKPGKGNTIMRMKLRDVRTGST

FDTSYRPEEKFEQAIIETVPAQYLYQMDDTAYFMNTETYDQYEIPVVNVE

DELKYILENSDVKIQFYGSEVIGVTVPTTVELVVTETQPSIKGATVTGSG

KPATLETGLVVNVPDFIEVGQKLIINTAEGTYVSRA

>fig|252305.3.peg.198 [Oceanicola batsensis HTCC2597] [Translation elongation factor P @ Translation initiation factor 5A]

MPKINGNEIRPGNVIEHNGGLWAAVKVDHVKPGKGGAFAQVELRNLRNGS

KLNERFRSADKVERVRLEQKDQQFLYESDGMLVFMDTETYEQIELPADLL

GERRPFLQDGMTIVVEFYESEALNATLPQKVTCKVVETEPVVKGQTAANS

FKPAILDNGVKVMVPPFVGTDEDIIVNTETMDYSERA

>fig|393130.3.peg.1094 [Listeria monocytogenes J0161] [Translation elongation factor P @ Translation initiation factor 5A]

MISVNDFKTGLTIEVDNGIWRVLDFQHVKPGKGAAFVRSKLRNLRTGAIQ

EKTFRGGEKVAKAQIDNRKMAYLYADGTNHVFMDNESYEQIELPEDQIAH

ELKFLKENMEINIIMYQGETIGIDLPNTVELVVTATDPGIKGDTSSGGSK

PATLETGLVVQVPFFVNEGDKLVINTTEAAYVSRA

>fig|320390.3.peg.3035 [Burkholderia mallei GB8 horse 4] [Translation elongation factor P @ Translation initiation factor 5A]

MKTAQELRVGNVVMIGNDAWVVSKTEYNKSGRNAAVVKMKLKNLLNGGGQ

ESVYKADDKFEVVVLDRKEVTYSYFADPMYVFMDADYNQYEVEAEMMGDA

LNYLEDGMACEVVFYNEKAISVELPTILVREITYTEPAVKGDTSSGKVLK

NAKLATGFELQVPLFCNTGDKIEIDTRTNEYRSRA

>fig|221109.1.peg.1895 [Oceanobacillus iheyensis HTE831] [Translation elongation factor P @ Translation initiation factor 5A]

MISVNDFKTGLTVEVDNDIWQVIEFQHVKPGKGAAFVRSKLRNLRSGNIQ

EKTFRAGEKVSKAHIENRKMQYLYASGDAHAFMDTNTYDQIEIQGKQIQE

QLKFIKENMEVSIVSYENEILGVDLPNNVELTVTETEPGIKGDTASGGSK

PATLETGLIVQVPFFINEGDVLVINTSDGKYVSRA

>fig|446466.5.peg.1758 [Cellulomonas flavigena DSM 20109] [Translation elongation factor P @ Translation initiation factor 5A]

MATTNDLKNGTVLKIDGQLWTVVEFQHVKPGKGGAFVRTKLKNVLSGKVV

DKTFNAGLKVETANVDKRDMQYLYKDGDDFVFMDTDTYDQINVPAATVGD

AANFMLESQTALVATNEGVPLYVELPPSVVLEVTYTEPGLQGDRSSAGTK

PATLETGYEIQVPLFLEANTKVKVDTRDGSYLGRVND

>fig|204722.1.peg.1652 [Brucella suis 1330] [Translation elongation factor P @ Translation initiation factor 5A]

MKINGNEIRPGNVIEHEGGLWVAVKTNAVKPGKGGAYNQVELKNLINGTK

LNERFRAAETVERVRLEQKDFSFLYEQGEALIFMDTETYEQLELQKDFVG

DRAAFLQDGMMVTVELYEEKPIGIRLPDQVTLAITEADPVVKGQTAASSY

KPAVLENGIRILVPPFIASGERVIVDTNELTYISRA

>fig|391037.3.peg.1791 [Salinispora arenicola CNS-205] [Translation elongation factor P @ Translation initiation factor 5A]

MATTNDLKNGMVLNLDGELWAVVEFQHVKPGKGGAFVRTTLKNVLSGKVV

DKTFNAGTKVDTATVDKRTMQYLYADGEDYVFMDLETFDQITVLGDTVGE

AANYLLPEAEAVVATHESVPLYVELPTSVVLEVTYTEPGLQGDRSTGGSK

PATVETGATVQVPLFITTGEKIKVDTRDGRYLGRA

>fig|339670.3.peg.1907 [Burkholderia ambifaria AMMD] [Translation elongation factor P @ Translation initiation factor 5A]

MKTAQELRVGNVVQIGSDAWVIAKTEYNKSGRNAAVVKMKMKNLLTNAGQ

ESVYKADDKFDVVMLDRKEVTYSYFADPMYVFMDADYNQYEVEAEMMGEA

LNYLEDGMACEVVFYNEKAISVELPTILVREITYTEPAVKGDTSSGKVLK

NAKLATGFELQVPLFCSTGDKIEIDTRTNEYRSRA

>fig|272560.3.peg.4312 [Burkholderia pseudomallei K96243] [Translation elongation factor P @ Translation initiation factor 5A]

MKTAQELRVGNVVMIGNDAWVVSKTEYNKSGRNAAVVKMKLKNLLNGGGQ

ESVYKADDKFEVVVLDRKEVTYSYFADPMYVFMDADYNQYEVEAEMMGDA

LNYLEDGMACEVVFYNEKAISVELPTILVREITYTEPAVKGDTSSGKVLK

NAKLATGFELQVPLFCNTGDKIEIDTRTNEYRSRA

>fig|262768.1.peg.276 [Onion yellows phytoplasma OY-M] [Translation elongation factor P @ Translation initiation factor 5A]

MINTNDFKTGQTIKFNNQIYQILEFLHVKPGKGAAFVRTKLRNLRTGSVI

DYTFNAGIKVQPALITKIKMQFLYALEDNYIFMNTQNYEQLEINKHQLKD

FLKYLYEGLLVDIIFYENDEIVGITLPDKISIKVAYTEPGAKGDTKTNSL

KDAALETGLVIKVPLFINIGEKIIINTETGLYLSRDNNK

>fig|393117.3.peg.1228 [Listeria monocytogenes FSL J1-194] [Translation elongation factor P @ Translation initiation factor 5A]

MISVNDFKTGLTIEVDNGIWRVLDFQHVKPGKGAAFVRSKLRNLRTGAIQ

EKTFRGGEKVAKAQIDNRKMAYLYADGTNHVFMDNESYEQIELPEDQIAH

ELKFLKENMEINIIMYQGETIGIDLPNTVELVVTATDPGIKGDTSSGGSK

PATLETGLVVQVPFFVNEGDKLVINTTEAAYVSRA

>fig|228405.5.peg.3008 [Hyphomonas neptunium ATCC 15444] [Translation elongation factor P @ Translation initiation factor 5A]

MAVEIAADIRRGNIIEHTDGQLYVVLKAESFRPGKGTPTTTIEMRRISDG

IKVVNTFKTSEKLEKAFVEEVEHTYLYPEGDNFVFMNSANYEQVTVSGAM

VGDGAPYLQENMPVSLQMFNGDPVSITLPPRFTAEIVETEPVVKGQTASG

SYKPAILENGVRIMVPAHVGVGTRVIINTEDGTYLERAKD

>fig|203119.1.peg.1881 [Clostridium thermocellum ATCC 27405] [Translation elongation factor P @ Translation initiation factor 5A]

MISAGDFKNGVTFELDGQIFQVIEFQHVKPGKGAAFVRTKLKNIVTGATI

EKTFNPTDKMPKAHIERKDMQYLYNDGDLYYFMDTETFEQLPLGKDKIGD

ALKFVKENEIVKVLSHKGNVFGIEPPNFVELEVTDTEPGFKGDTATGATK

PAIVETGASIKVPLFVNKGDIIRIDTRTGEYMERV

>fig|380703.5.peg.3078 [Aeromonas hydrophila subsp. hydrophila ATCC 7966] [Translation elongation factor P @ Translation initiation factor 5A]

MKTAQEIRAGNVVMIGTEPMVVQKAEFNKSGRNSAVVKMKLKGLLNGSAT

ETVFKADDKLEVVQLERKECTYSYFSDPLYVFMDTEYNQYDVEKDNLGDA

LNYMVDGMEDICEVTFYNEKAISVELPTTIVREVEYTEPAARGDTSGKVT

KPARLKGTTYELAVAAFVEIGDKIEIDTRTGEFKRRVN

>fig|316279.3.peg.830 [Synechococcus sp. CC9902] [Translation elongation factor P @ Translation initiation factor 5A]

MISSNDFRTGTSIEIDGAVWRVVEFLHVKPGKGSAFVRTKLKSVKNGSVV

EKTFRAGEMLPQALLEKSSLQHTYMEGDDYVFMDMSSYEETRLTAAQIGD

SRKYLKEGMEVNVVSWNDTPLEVELPNSVVLEIKETDPGVKGDTATGGTK

PAILETGAQVMVPLFLSIGEKIKVDTRNDSYLGREN

>fig|63737.4.peg.461 [Nostoc punctiforme PCC 73102] [Translation elongation factor P @ Translation initiation factor 5A]

MISSNDFRPGVSIVLDGSVWRVLEFLHVKPGKGSAFVRTKLKNVQSGSVM

EKTFRAGETVPQATLEKSTMQHTYKEGDEFVFMDMETYEEGRLTRTQIGD

RVKYLKEGMEAEVIKWGDQVLGVELPKSVVLEIVQTDPGLKGDTATGGSK

PATLETGAIVMVPLFISQGERIKVDTQEDKYISRE

>fig|221360.3.peg.1207 [Synechococcus sp. RS9917] [Translation elongation factor P @ Translation initiation factor 5A]

MLPQAMLEKSTLQHTYMEGEDYVFMDMSSYEETRLTAQQIGDSRKYLKEG

MEVNVVSWNGKPLEVELPNSVVLEITQTDPGVKGDTATGGTKPAILETGA

QVMVPLFLSIGEKIKVDTRNDTYLGRENG

>fig|479434.4.peg.1353 [Sphaerobacter thermophilus DSM 20745] [Translation elongation factor P @ Translation initiation factor 5A]

MIDTGDLRKGLTLEMNGELVRVVDFQHVKQGRGSAFVRLSLRNLRTGTIT

QQTFQAGSKFTPVRLERQRVQYLYNDEGQYHFMDVDTFEQFALDKETLGD

AVNYLIENETIDLLTHNGQPIDVELPVTVNLRVEQTEPGVRGDTAAGGSK

PARLETGLTINVPLFINEGDVVTVDTRTGEYLGRVN

>fig|218495.3.peg.1423 [Streptococcus uberis 0140J] [Translation elongation factor P @ Translation initiation factor 5A]

MIEASKLRAGMTFESEGKLIRVLEASHHKPGKGNTIMRMKLRDVRTGSTF

DTTYRPDEKFEQAIIETVPAQYLYKMDDTAYFMNTETYDQYEIPVVNVEQ

ELLYILENSDVKIQFYGSEVIGVTVPTTVELTVTETQPSIKGATVTGSGK

PATLETGLVVNVPDFIEAGQKLVINTAEGTYVSRA

>fig|266835.1.peg.3025 [Mesorhizobium loti MAFF303099] [Translation elongation factor P @ Translation initiation factor 5A]

MAKINGNEIRPGYVIEHDGGLWVAVRTNTVKPGKGGAYNQVELKNLINGT

KLNERFRSAETVEQIRLDLKDFSFLYEQDDALVFMDTQSYEQLELNKDFV

GDRAAFLQDGMMVTVQLYEERPIGISLPDQVTLTITEADPVVKGQTAAAS

YKPAVLKNGIRVLVPPFIGAGERIIVDTNEITYVRRAD

>fig|107806.1.peg.29 [Buchnera aphidicola str. APS (Acyrthosiphon pisum)] [Translation elongation factor P @ Translation initiation factor 5A]

MLYNQKSFHINRGCMRIYHSNNFRSGRKIIFENEPCLIESSEFVKPGKGQ

SFVRVKLRKLLTKQLIEKTFKSTDSLEIADIIEYTLSYLYNDGRFWYFIN

NNTFEELSVDEKIIGVHKKWLLEQDTCIVTLWNNQPISITPNNFVNLKVI

HVQATLKGDTINTSSTKLATLSTGAIVRVPLFIQVGSLIKVDTRSGEYVS

RIK

>fig|381764.6.peg.88 [Fervidobacterium nodosum Rt17-B1] [Translation elongation factor P @ Translation initiation factor 5A]

MIEVGDLEKGMYIKYEGDIYRVVDVNKHFRARGSGLIRTKLKSLSTGLIR

DANFASGEKVEEAELSFRKAEYLYNDGENYYFMDLQTYEQYAIPESEVDE

AKYYLIENTQVDLVMHDEKPIGINLPTTVVLEVIETEPNFKGDTVSGGGK

PAVLQTGLKISVPFFINVGDKIKVDTRTGEYIERA

>fig|269800.4.peg.341 [Thermobifida fusca YX] [Translation elongation factor P @ Translation initiation factor 5A]

MPYWLRETTEVATTNDLKNGMTLRLDDGELWNVVEFQHVKPGKGGAFVRT

KLKNVTTGKIVDKTFNAGVKVEVATVDKREMQYLYSDGDSYIFMDTETYD

QIYVPREVVGDNANYLLESAIVTVAVNEGTPLYIELPASVELTIAETEPG

LQGDRSTGGTKPATLETGAVIQVPLFVTTGDKVKVDTRTGEYLGRVN

>fig|882.1.peg.1659 [Desulfovibrio vulgaris subsp. vulgaris str. Hildenborough] [Translation elongation factor P @ Translation initiation factor 5A]

MYSTTDFRKGLKIELDGTPFEIVDFQHFKPGKGGAMVRTKLRNILNGRVV

DNTFRSGEKVGRPDLESRDMQYLYHEGDDLVLMDLTTYEQLYMHEDLTDG

KAGFLKDGQQVRVLLYNGKPLDLELPVSLVLEVVETEPGAKGDTVSNVTK

PAKLETGIVVQVPIFVNQGDRIKVDTRSREYLGRE

>fig|406559.4.peg.387 [Streptococcus pneumoniae SP11-BS70] [Translation elongation factor P @ Translation initiation factor 5A]

MIEASKLKAGMTFETADGKLIRVLEASHHKPGKGNTIMRMKLRDVRTGST

FDTSYRPEEKFEQAIIETVPAQYLYKMDDTAYFMNTETYDQYEIPVVNVE

NELLYILENSDVKIQFYGTEVIGVTVPTTVELTVAETQPSIKGATVTGSG

KPATMETGLVVNVPDFIEAGQKLVINTAEGTYVSRA

>fig|211110.1.peg.1704 [Streptococcus agalactiae NEM316] [Translation elongation factor P @ Translation initiation factor 5A]

MIEASKLKAGMTFETADGKLIRVLEASHHKPGKGNTIMRMKLRDVRTGST

FDTSYRPEEKFEQAIIETVPAQYLYKMDDTAYFMNNETYDQYEIPTVNIE

NELLYILENSEVKIQFYGTEVIGVQIPTTVELTVAETQPSIKGATVTGSG

KPATMETGLVVNVPDFIEAGQKLVINTAEGTYVSRA

>fig|272564.4.peg.875 [Desulfitobacterium hafniense DCB-2] [Translation elongation factor P @ Translation initiation factor 5A]

MISSNEFKTGLTIEVDNDVYTIIEFQHVKPGKGAAFVRTKLKNVKTGGII

ERKFNAGEKVPKAHVERREMQYLYKDGDHFVAMDNETYEQTSLTEAQIGD

GVKYLKENMNLGILFFNGTVIGVDLPNTVILEVAHTEPGVRGDTATGGSK

PATLETGAVVQVPFFVNEGEKLIIDTRTGNYVQRA

>fig|60481.10.peg.1830 [Shewanella sp. MR-7] [Translation elongation factor P @ Translation initiation factor 5A]

MKTAHEVRPGNVIMFEGSPWVVQKTETTRSGRNAAIVKLKLKNLLLNSGT

ETTFKGEDKIDDIILDRLDCTYSYFADPMYVFMDAEYNQYDVEAENLGDA

AAYIVDGMEETCQVTFYEGKAISVEMPTTIVREVIYTEPSARGDTSGKVM

KPATITGGGTISVADFVKVGDKIEIDTRTGEFKKRV

>fig|350703.3.peg.1740 [Pseudomonas aeruginosa 2192] [Translation elongation factor P @ Translation initiation factor 5A]

MKTAQEFRAGQVANINGAPWVIQKAEFNKSGRNAAVVKMKLKNLLTGAGT

ETVFKADDKLEPIILDRKEVTYSYFADPLYVFMDSEFNQYEIEKDDLEGV

LTFIEDGMTDICEAVFYNDKVISVELPTTIVRQIAYTEPAVRGDTSGKVM

KTARLNNGAELQVSAFCEIGDSIEIDTRTGEYKSRVKA

>fig|190304.1.peg.1296 [Fusobacterium nucleatum subsp. nucleatum ATCC 25586] [Translation elongation factor P @ Translation initiation factor 5A]

MKIAQELRAGSTIKIGNDPFVVLKAEYNKSGRNAAVVKFKMKNLISGNIS

DAVYKADDKMDDIKLDKVKAIYSYQNGDSYIFSNPETWEEIELKGEDLGD

ALNYLEEEMPLDVVYYESTAVAVELPTFVEREVTYTEPGLRGDTSGKVMK

PARINTGFEVQVPLFVEQGEWIKIDTRTNEYVERVKK

>fig|218496.1.peg.379 [Tropheryma whipplei TW08/27] [Translation elongation factor P @ Translation initiation factor 5A]

MASTSDIRNGVVLNINGQLNTVIEFQHVKPGKGGAFVRTKLKNILTGKVV

DKTFNAGASVVLENVDRRDCTYLYRDADSFVFMDLADYDQIRLTASQVAS

AANYLSDNQKVVIATHNNAPLYVDLPPSVVLAVTHTEPGVQADRSTGGTK

PATLETGYQIQVPLFITVGTRIRVDTRTGAYIGKA

>fig|319224.13.peg.1721 [Shewanella putrefaciens CN-32] [Translation elongation factor P @ Translation initiation factor 5A]

MKTAHEIRPGNVIMLDGSPWVVQKTETTRSGRNAAIVKLKLKNLLLNSGT

ETTFKGEDKLEDIVLDRLDCTYSYFADPMYVFMDAEYNQYDVEAENLGDA

AAYIVDGMEETCQVTFYDGKAISVEMPTTIVREVIYTEPSARGDTSGKVM

KPATITGGGTVSVADFVKVGDKIEIDTRTGEFKKRV

>fig|195102.1.peg.1898 [Clostridium perfringens str. 13] [Translation elongation factor P @ Translation initiation factor 5A]

MISGSDLRKGTTFELDGQVYTVIDFLHVKPGKGAAFVRTKLRNVIMGGVT

DRTFNPTDKLQEAIIERKEMQYLYSDGELYYFMDQETFEQIPLNHEKVED

AIKFLKENMNAVIKFYKGEAFSVEAPNFVELLITECEPSVKGNTATTAMK

TAVVETGATVMVPMFVEEGNTIRIDTRTGEYMERV

>fig|384676.6.peg.1357 [Pseudomonas entomophila L48] [Translation elongation factor P @ Translation initiation factor 5A]

MKTGKELKPGTVLRIDNDPWLVQKAEFTKSGRNSAIMKTKLKNLLTGYKT

ETVYGADDKLDDVILDRKEATLSFISGDSYTFMDTTDYTMYELNAEDIDA

VLPYIEEGMEDICEAVFFEGRLVSVELPTTISRQVTYTENAARGDTSGKV

MKPAKLKNGTEIQVADFIQIDEWIDIDTRDNSFKGRSKK

>fig|306264.1.peg.271 [Campylobacter upsaliensis RM3195] [Translation elongation factor P @ Translation initiation factor 5A]

MATYAMGDLKKGLKIEIDGVPFKIVEYQHVKPGKGPAFVRVKIKSFIDGK

VLEKTIHAGDKCEAPNLEQKTMQYLYDDGENCQFMDTQSYEQVAISDDDV

GEAKKWMLDGMMVEVLFHNSKAIGVEVPQVVELKIIETAPNFKGDTQGSN

KKPATLETGAVVQIPFHVLEGEVIRVDTTRGEYIEKANK

>fig|393124.3.peg.958 [Listeria monocytogenes FSL N3-165] [Translation elongation factor P @ Translation initiation factor 5A]

MISVNDFKTGLTIEVDNGIWRVLDFQHVKPGKGAAFVRSKLRNLRTGAIQ

EKTFRGGEKVAKAQIDNRKMAYLYADGTNHVFMDNESYEQIELPEDQIAH

ELKFLKENMEINIIMYQGETIGIDLPNTVELVVTATDPGIKGDTSSGGSK

PATLETGLVVQVPFFVNEGDKLVINTTEAAYVSRA

>fig|453363.3.peg.58 [Streptococcus pneumoniae SP195] [Translation elongation factor P @ Translation initiation factor 5A]

MIEASKLKAGMTFETADGKLIRVLEASHHKPGKGNTIMRMKLRDVRTGST

FDTSYRPEEKFEQAIIETVPAQYLYKMDDTAYFMNTETYDQYEIPVVNVE

NELLYILENSDVKIQFYGTEVIGVTVPTTVELTVAETQPSIKGATVTGSG

KPATMETGLVVNVPDFIEAGQKLVINTAEGTYVSRA

>fig|319701.3.peg.46 [Streptococcus pyogenes MGAS6180] [Translation elongation factor P @ Translation initiation factor 5A]

MIEASKLKAGMTFESEGKLIRVLEASHHKPGKGNTIMRMKLRDVRTGSTF

DTTYRPDEKFEQAIIETVPAQYLYKMDDTAYFMNTDTYDQYEIPVANVEQ

ELLYILENSDVKIQFYGSEVIGVTVPTTVELTVAETQPSIKGATVTGSGK

PATLETGLVVNVPDFIEAGQKLIINTAEGTYVSRA

>fig|226186.1.peg.3707 [Bacteroides thetaiotaomicron VPI-5482] [Translation elongation factor P @ Translation initiation factor 5A]

MINAQDIKNGTCIRMDGKLYFCIEFLHVKPGKGNTFMRTKLKDVVSGYVL

ERRFNIGEKLEDVRVERRPYQFLYKEGEDYIFMNQETFDQHPIAHDLING

VDFLLEGAVLDVVSDASTETVLYADMPIKVQMKVTYTEPGMKGDTATNTL

KPATVESGATVRVPLFISEGETIEIDTRDGSYVGRVKA

>fig|182082.1.peg.922 [Chlamydophila pneumoniae TW-183] [Translation elongation factor P @ Translation initiation factor 5A]

MVRVSTSEFRVGLRIEIDGQPYLILQNDFVKPGKGQAFNRIKVKNFLTGR

VIERTYKSGESVETADIVERSMRLLYTDQEGATFMDDETFEQEVVFWEKL

ENIRQWLLEDTIYTLVLYNGDVVAVEPPIFMELSIAETAPGVRGDTASGR

VLKPAVTNTGAKIMVPIFIDEGELVKVDTRTGSYESRVSK

>fig|526225.5.peg.1010 [Geodermatophilus obscurus DSM 43160] [Translation elongation factor P @ Translation initiation factor 5A]

MATTNDLKNGMVLNLDGQLWAVTEFQHVKPGKGGAFVRTTLKNVLSGKVV

DRTFNAGTKVDTATVDKRNMTYLYKDGTDFVFMDGETYDQIHVPAETVGG

GADYLLENTEVVVAVHEGTPLYVELPVTLELVVQHTDPGLQGDRSTGGTK

PATLETGAQIQVPLFVNTGDKLKVDTRDGRYLGRAN

>fig|198094.1.peg.4077 [Bacillus anthracis str. Ames] [Translation elongation factor P @ Translation initiation factor 5A]

MISVNDFRTGLTIAVDNGLWQVLDFQHVKPGKGAAFVRSKLRNLRTGSVQ

EKTFRAGEKVEKAHIENRRMQYLYASGEAHVFMDNGTYEQIELGEKQIER

ELKFLKENMEVSIMTYQGEVLGVELPNTVELQVTETEPGIKGDTASNVTK

PATLETGLVVQVPIFINEGEMLIINTGEGKYVSRA

>fig|94122.5.peg.2205 [Shewanella sp.ANA-3] [Translation elongation factor P @ Translation initiation factor 5A]

MKTAHEVRPGNVIMFEGSPWVVQKTETTRSGRNAAIVKLKLKNLLLNSGT

ETTFKGEDKIDDIILDRLDCTYSYFADPMYVFMDAEYNQYDVEAENLGDA

AAYIVDGMEETCQVTFYEGKAISVEMPTTIVREVIYTEPSARGDTSGKVM

KPATITGGGTISVADFVKVGDKIEIDTRTGEFKKRV

>fig|477974.3.peg.948 [Desulforudis audaxviator MP104C] [Translation elongation factor P @ Translation initiation factor 5A]

MISTNEFRTGLTVEVDGDPCQVIEFMHVKPGKGSPFVRAKLKNLRTGAIA

ERTFNAGEKLPRAILERKEMQYLYNDGANYYLMDNETYDQVGLSAGQLGD

GVKYLKENMIINVVYHRGQVLGVDLPNTVELTVIETTPGIRGDTASGGSK

PAVLETGVVLQVPLFVEEGDVIQVDTRSGAYIKRA

>fig|299768.3.peg.501 [Streptococcus thermophilus CNRZ1066] [Translation elongation factor P @ Translation initiation factor 5A]

MIEASKLRAGMTFVTNDGKLLKVLEASHHKPGKGNTIMRMKLRDVRSGST

FDTSYRPEEKFEQAIIETVPAQYLYQMDDTAYFMNTETYDQYEIPVVNVQ

EELKFILENSDVKIQFYGTEVIGVQVPTTVELTVTETQPSIKGATVTGSG

KPATLETGLVVNVPDFIEAGQKLVINTVEGTYVSRA

>fig|1313.4.peg.424 [Streptococcus pneumoniae INV200] [Translation elongation factor P @ Translation initiation factor 5A]

MIEASKLKAGMTFETADGKLIRVLEASHHKPGKGNTIMRMKLRDVRTGST

FDTSYRPEEKFEQAIIETVPAQYLYKMDDTAYFMNTETYDQYEIPVVNVE

NELLYILENSDVKIQFYGTEVIGVTVPTTVELTVAETQPSIKGATVTGSG

KPATMETGLVVNVPDFIEAGQKLVINTAEGTYVSRA

>fig|387344.13.peg.954 [Lactobacillus brevis ATCC 367] [Translation elongation factor P @ Translation initiation factor 5A]

MAISTADFKNGLTIEVDNAIWRIIEFQHVKPGKGGAFVRSKLKNLRTGAV

QEKTFRAGAKMERADIQTRSMQYLYEDGDNRVFMDTDNFEQIEVPEEQIK

DQLPYLQENMNVDLVQFGSEVLGIEVPNTVVLEVTATEPGIKGNTASGGS

KPATMNTGLVVQVPFFVNEGDKLSINTTDGTYISRA

>fig|269798.12.peg.2400 [Cytophaga hutchinsonii ATCC 33406] [Translation elongation factor P @ Translation initiation factor 5A]

MATTSDISKGCFFKQDGELLTVLSYDHITPGKGNAIYSTKCRNVKTGKQS

EVRFRSGEKVDIVRVTIVEMQYLYEEGDSLVCMNQETFEQISVPKVAFGD

AIKFVKEEMILSIRFDDDEQPLDGVLPKHVELVVTYTENGIKGDSTSKSL

KAAEVEGGINVQVPLFVETGDKLRINTETGEYVERVK

>fig|269798.12.peg.1456 [Cytophaga hutchinsonii ATCC 33406] [Translation elongation factor P @ Translation initiation factor 5A]

MAKPNDLSRGSFIRYNGELLTITEIEHRTPGNLRAFYQAKMKNVKSGKSA

EARFRADEDIDMVRVEVKELQYLYKDGDSLVCMDTETFDQIYIPTILFGD

AIDFVKEEMNMLISFDGETPIQGELPSHVEMEVTYTEPGIKGDTATKTLK

PATVDNGPTITVPLFVEIGDRIKIDTRTRAYVERVK

>fig|272635.1.peg.513 [Mycoplasma pulmonis UAB CTIP] [Translation elongation factor P @ Translation initiation factor 5A]

MINVNEFKPGITFEDEGNIYVVLTAQHSKQGRGQANVKAKVKNLRTGSTT

LKSYTGGDKVQKAHIEKKPMDYLYNDGSNIILMDQESFEQIEIDVKKVEW

ELNFLTEGMKILVRQYQNEILDIEIPINIELKVINAPDAVKGNTTTNPQK

KVIVETGYELEVPMFIKEGETIIVSSETGKYGGKSSK

>fig|243273.1.peg.26 [Mycoplasma genitalium G-37] [Translation elongation factor P @ Translation initiation factor 5A]

MAEMIEAKNLRNGQTIFGPNKEILLVLENTFNKTAMRQGIVKTKVKNLRT

GAIVWLEFTGDKLEQVIIDKKKMNFLYKDGNNFVFMDQKDYSQIEINEKK

LEWEKNFITEEIEVTVITYQDEILGVNLPDLVPIEVEFAEDAIQGNTANM

ARKKARLVTGYELDVPQFINTGDKIVIATVDGNYRERFNK

>fig|402881.6.peg.2073 [Parvibaculum lavamentivorans DS-1] [Translation elongation factor P @ Translation initiation factor 5A]

MKINGNEIRPGNVIEHEGGLWAAVKVQHVKPGKGGAFAQVELKNLVDGRK

LNERFRSAETVERVRLEQKDYQYLYENDGMLTFMDNETYEQIELSREWVG

DRAAFLQDGMKVTVESHEGKALGISLPDQVVLEITETEPTVKGQTATSSY

KPALLENGVRVMVPPFITTGERIVVDTNEVTYLRRAD

>fig|406563.4.peg.1847 [Streptococcus pneumoniae SP23-BS72] [Translation elongation factor P @ Translation initiation factor 5A]

MIEASKLKAGMTFETADGKLIRVLEASHHKPGKGNTIMRMKLRDVRTGST

FDTSYRPEEKFEQAIIETVPAQYLYKMDDTAYFMNTETYDQYEIPVVNVE

NELLYILENSDVKIQFYGTEVIGVTVPTTVELTVAETQPSIKGATVTGSG

KPATMETGLVVNVPDFIEAGQKLVINTAEGTYVSRA

>fig|83332.1.peg.2536 [Mycobacterium tuberculosis H37Rv] [Translation elongation factor P @ Translation initiation factor 5A]

MATTADFKNGLVLVIDGQLWTITEFQHVKPGKGPAFVRTKLKNVLSGKVV

DKTFNAGVKVDTATVDRRDTTYLYRDGSDFVFMDSQDYEQHPLPEALVGD

AARFLLEGMPVQVAFHNGVPLYIELPVTVELEVTHTEPGLQGDRSSAGTK

PATLQTGAQINVPLFINTGDKLKVDSRDGSYLGRVNA

>fig|69042.3.peg.1136 [Synechococcus sp. WH 5701] [Translation elongation factor P @ Translation initiation factor 5A]

MAVPAGLRAFCRSSDFGTACRGPEPVSEGGSTASRTIPLTRSAGMISSND

FRTGTTIELDGQVWRVVEFLHVKPGKGSAFVRTKLKAVQSGSVVEKTFRA

GETLAQAQLEKSTLQHTYMEAEEYVFMDMATYEETRLTAKQIGDGRKYLK

EGMEVNVVSWNGSPLEVELPNSVVLEVTQTDPGVKGDTATGGTKPAIVET

GAQVMVPLFITIGEKIKIDTRNDSYLGRES

>fig|398578.3.peg.2862 [Delftia acidovorans SPH-1] [Translation elongation factor P @ Translation initiation factor 5A]

MKIAQEIRAGNVIMHGKDPMIVLKTEYARGGRGAATVRMKLKSLLGNMGT

EVVFKADDKIDNVILDKKECTYSYFADPMYVWMDADFNQYEVEATNMGDA

INYLEDGMEAEVVFYDGKAISCELPTSVEREITWTEPAVKGDTSGKVLKP

AKIATGFEVPVPLFVSQGDKIEIDTRTGEYRKRV

>fig|64471.10.peg.34 [Synechococcus sp. CC9311] [Translation elongation factor P @ Translation initiation factor 5A]

MNRAGSPQDPVRHCLEAGLPIPVWQARGQRRLARQEAERSDGGEPRRTPG

AVRTLAQPYDPLNRSAGMISSNDFRTGTTIELDGAVWRVVEFLHVKPGKG

SAFVRTKLKAVQSGSVVEKTFRAGEMLQQALLEKSTLQHTYMEGEDFVFM

DMSTYEETRLTAKQIGDSRKYLKEGMEVNVVTWNEKPLEVELPNSVVLEI

AQTDPGVKGDTATGGTKPAILETGAQVMVPLFLSIGEKIKVDTRNDTYLG

RENG

>fig|272631.1.peg.319 [Mycobacterium leprae TN] [Translation elongation factor P @ Translation initiation factor 5A]

MATTADFKNGLVLVIDGQLWTIIGFQHVKPGKGPAFVRTKLKNVLSGKVV

DKTYNAGVKVDTATVDRRDTTYLYRDGANFVFMDSQDYEQHPLPESLVGD

TARFLLEGMSVQVAFHNGVPLYVELPVTVELEVTHTEPGLQGDRSSAGTK

PATLETGAQINVPLFINTGDKLKVDSRDGSYLGRVNV

>fig|357809.4.peg.2499 [Clostridium phytofermentans ISDg] [Translation elongation factor P @ Translation initiation factor 5A]

MISAGDFRNGLTLEIDNAVYQVIEFQHVKPGKGAAFVRTKLRDIKNGGLT

ERTFRPQEKYPQAHIERSDMQYLYSDGELYNFMNVETFDQIALNDEAVGD

SLKFVKENDMVKMLSHQGNVFAIEPPLFVELVIVDTEPGFKGDTAQGATK

PAKVETGATVYVPLFVNQGDKISIDTRTGDYMKRV

>fig|323850.3.peg.1197 [Shewanella sp. PV-4] [Translation elongation factor P @ Translation initiation factor 5A]

MKTAHEIRPGNVIMLDGSPWVVQKTETTRSGRNAAIVKMKLKNLLQESST

ETTFKGEDKMEDIILDRLDCTYSYFADPMYVFMDAEYNQYDVEADNLGDA

AAYIVDGMEEQCQVTFYEGKAISVELPTTVVREVTYTEPSARGDTSGKVM

KPATIAGGATLSVADFVKTGDLIEIDTRTHEFKKRA

>fig|391009.4.peg.883 [Thermosipho melanesiensis BI429] [Translation elongation factor P @ Translation initiation factor 5A]

MIDVGSLSKGMYIKYDGDIYRVVDVNKHFRARGSGLIRTKLKNLSSGLVR

EVNFNSGEKVEEAEVTFRKASYIYNDGEMYYFMDAETFEQYGIPETEIED

EKNYLVENTEVDLIMHDGKPIGIQLPTSVVLEVVETEPGFKGDTVSGGGK

PAILETGLKITVPFFVEKGQKVRVDTRTGEYIERA

>fig|83331.1.peg.2696 [Mycobacterium tuberculosis CDC1551] [Translation elongation factor P @ Translation initiation factor 5A]

MATTADFKNGLVLVIDGQLWTITEFQHVKPGKGPAFVRTKLKNVLSGKVV

DKTFNAGVKVDTATVDRRDTTYLYRDGSDFVFMDSQDYEQHPLPEALVGD

AARFLLEGMPVQVAFHNGVPLYIELPVTVELEVTHTEPGLQGDRSSAGTK

PATLQTGAQINVPLFINTGDKLKVDSRDGSYLGRVNA

>fig|320373.3.peg.3355 [Burkholderia pseudomallei 668] [Translation elongation factor P @ Translation initiation factor 5A]

MKTAQELRVGNVVMIGNDAWVVSKTEYNKSGRNAAVVKMKLKNLLNGGGQ

ESVYKADDKFEVVVLDRKEVTYSYFADPMYVFMDADYNQYEVEAEMMGDA

LNYLEDGMACEVVFYNEKAISVELPTILVREITYTEPAVKGDTSSGKVLK

NAKLATGFELQVPLFCNTGDKIEIDTRTNEYRSRA

>fig|306263.1.peg.38 [Campylobacter lari RM2100] [Translation elongation factor P @ Translation initiation factor 5A]

MASYGMGDLKKGLKIEIDGIPFKIVEYQHVKPGKGPAFVRIKIKSFIDGK

VLEKTFHAGDKCESPNLEEKQMQYLYDDGENCQFMDTQTYEQVAISDEDV

GEAKKWMLDGTMVDVLFHNGKAIGVEVPQVMELKIIETAPNFKGDTQGSN

KKPATLETGAVVQIPFHVLEGEIIRVDTVRGEYIERANK

>fig|323848.3.peg.1386 [Nitrosospira multiformis ATCC 25196] [Translation elongation factor P @ Translation initiation factor 5A]

MAKVLATEIRVGNLIEWDKRIWRVLKCYHVHVGGRGGAFMQVEMKDIEAG

TKTNQRIRTEDKVERAFVEPREMTFLYQEGDNYIFMDKENYEQLSLSRDF

LEGQYEYLLPNTDVQVNFHNDRAIGVQLPASVVLTITDTEPNLKGATATS

SYKPATTETGLVVMVPPFVLQGERIKVNTDSGEYIERM

>fig|360107.5.peg.128 [Campylobacter hominis ATCC BAA-381] [Translation elongation factor P @ Translation initiation factor 5A]

MSYSMGDLKKGLKIEIDGIPYKIVEYQHVKPGKGPAFVRVKIKSFIDGKV

LEKTFHAGDKCETPNLEEKEMQYLYDDGEFCQFMDTETYEQIAISDEEVG

DNKKWMLDGTMVNVLFHNGRAIGIEIPQVVELKIIETAPDFRGDTQGSNK

KPATLETGAVVQIPFHILEGEVIRVDTVRGEYIEKANK

>fig|192222.1.peg.513 [Campylobacter jejuni subsp. jejuni NCTC 11168] [Translation elongation factor P @ Translation initiation factor 5A]

MASYSMGDLKKGLKIEIDGIPFKIVEYQHVKPGKGPAFVRIKIKSFIDGK

VLEKTFHAGDKCEAPNLEDKTMQYLYDDGENCQFMDTQTYEQVAISDDDV

GEAKKWMLDGMMVDVLFHNGKAIGVEVPQVVELKIIETAPNFKGDTQGSN

KKPATLETGAVVQIPFHVLEGEVIRVDTVRGEYIERANK

>fig|269801.1.peg.4885 [Bacillus cereus G9241] [Translation elongation factor P @ Translation initiation factor 5A]

MISVNDFRTGLTISVDNSLWQVLDFQHVKPGKGAAFVRSKLRNLRTGSVQ

EKTFRAGEKVEKAHIENRRMQYLYASGEAHVFMDNGTYEQIELGEKQIER

ELKFLKENMEVSIMTYQGEVLGVELPNTVELQVTETEPGIKGDTASNVTK

PATLETGLVVQVPIFINEGEMLIINTGEGKYVSRA

>fig|262543.4.peg.904 [Exiguobacterium sibiricum 255-15] [Translation elongation factor P @ Translation initiation factor 5A]

MVSVNDLKTGLTIKTSDGMIWQVLEFQHVKPGKGAAFVRTKMRNIRNGNI

QEMTFRGGERVERAHIERNKMQYLYPMGETYVFMDTESYEQLELTTAQVE

AALPFLLENMEVQIAIYNGEVLGIELPNTIVMTIVEAEPGVKGDTASNVK

KNATVETGHIIHVPLFIEAGEKVTVDTRTGDFTGRYNG

>fig|242231.4.peg.414 [Neisseria gonorrhoeae FA 1090] [Translation elongation factor P @ Translation initiation factor 5A]

MKTAQELRAGNVFMVGNDPMVVQKTEYIKGGRSSAKVSMKLKNLLTGAAS

ETIYKADDKFDVVILSRKNCTYSYFADPMYVFMDEEFNQYEIEADNIGDA

LKFIVDGMEDQCEVTFYEGNPISVELPTIIVREVEYTEPAVKGDTSGKVM

KTARLVGGTEIQVMSYIENGDKVEIDTRTGEFRKRA

>fig|265311.3.peg.454 [Mesoplasma florum L1] [Translation elongation factor P @ Translation initiation factor 5A]

MSVNDLRPGTTFIYEGNLFVVIEQSFSKTGRQQGKVSVKAKNLRTGSRVE

ITFTGGEKVEKAMIERKDMQYLYNDGTDAYLMDTDTYEQIQIPMTRLEWE

SKFLTDGLMIKMTEYDGEVLGISLPDKVELEVTEAEAAVKGDTTSGALKK

AVVETGLDIMVPLFVNVGTKVIISTTDGKYSGRAQ

>fig|320387.3.peg.3166 [Burkholderia mallei 10399] [Translation elongation factor P @ Translation initiation factor 5A]

MKTAQELRVGNVVMIGNDAWVVSKTEYNKSGRNAAVVKMKLKNLLNGGGQ

ESVYKADDKFEVVVLDRKEVTYSYFADPMYVFMDADYNQYEVEAEMMGDA

LNYLEDGMACEVVFYNEKAISVELPTILVREITYTEPAVKGDTSSGKVLK

NAKLATGFELQVPLFCNTGDKIEIDTRTNEYRSRA

>fig|224308.1.peg.2449 [Bacillus subtilis subsp. subtilis str. 168] [Translation elongation factor P @ Translation initiation factor 5A]

MISVNDFRTGLTIDVDGGIWRVVDFQHVKPGKGAAFVRSKLRNLRTGAIQ

EKTFRAGEKVAKAQIETKTMQYLYANGDQHVFMDTSSYEQLELSATQIEE

ELKYLLENMSVHIMMYQDETLGIELPNTVELKVVETEPGIKGDTASGGTK

PAKTETGLVVNVPFFVNEGDTLVVNTSDGSYVSRA

>fig|269483.3.peg.5473 [Burkholderia cepacia R18194] [Translation elongation factor P @ Translation initiation factor 5A]

MKTAQELRVGNVVQIGSDAWVIAKAEYNKSGRNSAVVKMKMKNLLTNAGQ

EAVYKADDKFDVVVLDRKEVTYSYFADPMYVFMDADYNQFEVEAEMMGEA

LNYLEDGMACEVVFYNEKAISVELPTVLVREITYTEPAVKGDTSSGKVLK

NAKLATGFELQVPLFCNTGDKIEIDTRTNEYRSRA

>fig|272558.1.peg.2799 [Bacillus halodurans C-125] [Translation elongation factor P @ Translation initiation factor 5A]

MISVNDFKTGLTIEVDNGIWQVMEFQHVKPGKGAAFVRSKLRNLRTGAVQ

EKTFRAGEKVSKAHIENRRMQYLYASGDVHTFMDNETFEQLELSTAQIEH

ELKFLKENMEVHVISYQGETLGVEVPNTVELTVTETEPGIKGDTASGGTK

PATLETGLTVQVPFFVNEGDVLVIDTRSGDYVSRA

>fig|170187.1.peg.404 [Streptococcus pneumoniae TIGR4] [Translation elongation factor P @ Translation initiation factor 5A]

MIEASKLKAGMTFETADGKLIRVLEASHHKPGKGNTIMRMKLRDVRTGST

FDTSYRPEEKFEQAIIETVPAQYLYKMDDTAYFMNTETYDQYEIPVVNVE

NELLYILENSDVKIQFYGTEVIGVTVPTTVELTVAETQPSIKGATVTGSG

KPATMETGLVVNVPDFIEAGQKLVINTAEGTYVSRA

>fig|516950.3.peg.448 [Streptococcus pneumoniae CGSP14] [Translation elongation factor P @ Translation initiation factor 5A]

MIEASKLKAGMTFETADGKLIRVLEASHHKPGKGNTIMRMKLRDVRTGST

FDTSYRPEEKFEQAIIETVPAQYLYKMDDTAYFMNTETYDQYEIPVVNVE

NELLYILENSDVKIQFYGTEVIGVTVPTTVELTVAETQPSIKGATVTGSG

KPATMETGLVVNVPDFIEAGQKLVINTAEGTYVSRA

>fig|398579.3.peg.1755 [Shewanella pealeana ATCC 700345] [Translation elongation factor P @ Translation initiation factor 5A]

MKTAHELRPGNVIMLDGSPWVVQKTETTRSGRNAAIVKLKLKHVLQDSST

ESTFKGEDKMEDIILERLDCTYSYFADPMYVFMDAEYNQYDVEAENLGDA

AAYIVDGMEENCQVTFYEGKAISVELPTSVVREVTYTEPSARGDTSGKVM

KPATITGGGTLSVADFVKTGDMIEIDTRTNEFKKRV

>fig|342451.4.peg.1473 [Staphylococcus saprophyticus subsp. saprophyticus ATCC 15305] [Translation elongation factor P @ Translation initiation factor 5A]

MISVNDFKTGLTISVDNGIWKVLDFQHVKPGKGSAFVRSKLRNLRTGAIQ

EKTFRGGEKVETALIENRRMQYLYADGDTHVFMDNQTFEQTELPADYLEY

ELNFLKANMEVQIQSYENETLGVELPKTVELTVTETEPGIKGDTANGATK

SATVETGYTLNVPLFVNEGDVLVINTGDGSYISRA

>fig|235909.3.peg.2460 [Geobacillus kaustophilus HTA426] [Translation elongation factor P @ Translation initiation factor 5A]

MISVNDFRTGLTIEVDGEIWRVLEFQHVKPGKGAAFVRSKLRNLRTGAIQ

ERTFRAGEKVNRAQIDTRKMQYLYANGDQHVFMDMETYEQIELPAKQIEH

ELKFLKENMEVFIMMYQGETIGVELPNTVELKVVETEPGIKGDTASGGSK

PAKLETGLVVQVPFFVNEGDTLIINTADGTYVSRA

>fig|406558.4.peg.1429 [Streptococcus pneumoniae SP9-BS68] [Translation elongation factor P @ Translation initiation factor 5A]

MIEASKLKAGMTFETADGKLIRVLEASHHKPGKGNTIMRMKLRDVRTGST

FDTSYRPEEKFEQAIIETVPAQYLYKMDDTAYFMNTETYDQYEIPVVNVE

NELLYILENSDVKIQFYGTEVIGVTVPTTVELTVAETQPSIKGATVTGSG

KPATMETGLVVNVPDFIEAGQKLVINTAEGTYVSRA

>fig|502025.5.peg.1863 [Haliangium ochraceum DSM 14365] [Translation elongation factor P @ Translation initiation factor 5A]

MEFQFVKPGKGSAFTRTKFKNLLTGGVVEKNIRSGEKLEPANVEERDMQF

LYHDGEGFVFMDQSNYEQVTVTADIVGDNHDMMLDNLQCQVLFFNDRAVD

VSLPNFIEVQVTETEPGARGDTSGNVTKPATVSTGAEVAVPLFIKEGDWL

KVDTRTKSYVERVNK

>fig|387093.4.peg.545 [Sulfurovum sp. NBC37-1] [Translation elongation factor P @ Translation initiation factor 5A]

MATIGMGDIKKGVRLELDGNPYKVTEFQHVKPGKGAAFVRVKIKNLKTGK

VIEKTVHAGDKFEVPELEQKTMQYLYDDGEMLQFMDTTTFDQIGLTHEQV

GKETFDFMIDGMEADILFHNGEAISVEIPQTVVLKIVETPPNFKGDSQGG

KKPATLESGAVVQVPFHVLEGEMIKVDTVEGKYLEKAK

>fig|293614.3.peg.328 [Rickettsia akari str. Hartford] [Translation elongation factor P @ Translation initiation factor 5A]

MKISANSIRTGNILVCNNDLWVVSKTPEHTQPGKGGAYVQVEMKNLKTGI

KRNDRFSSSDYLEKAELEQKDYQFLYFEGNDLILMDTKHFDQINVPKGIL

EEKLPFLTENMIVKVEFYNEKPLNIELPPTVILEISETDPVIKGATATAS

YKPAVLENGIKVKVPQYLAIGEKIVVKTDDITYVERAK

>fig|525904.4.peg.2617 [Thermobaculum terrenum ATCC BAA-798] [Translation elongation factor P @ Translation initiation factor 5A]

MISTGELRKGNTLVIDGDLVRVLDFQHVKLGRGSAFVRVQLKNLRTGAIT

ERTFQAGTKFETARLERRTVQYLYNDGENYTFMDTETFDQPTLSADLLGD

AVKYLKEGMNIDLLMYGDEPIGVELPTTVELQVVQTDPGVRGDTAAGGSK

PARLETGLVVQVPLFINEGDIIRVDTRTGEYIERVG

>fig|224914.1.peg.326 [Brucella melitensis 16M] [Translation elongation factor P @ Translation initiation factor 5A]

MKINGNEIRPGNVIEHEGGLWVAVKTNAVKPGKGGAYNQVELKNLINGTK

LNERFRAAESVERVRLEQKDFSFLYEQGEALIFMDTETYEQLELQKDFVG

DRAAFLQDGMMVTVELYEEKPIGIRLPDQVTLAITEADPVVKGQTAASSY

KPAVLENGIRIPVPPFIASGERVIVDTNELTYISRA

>fig|479433.4.peg.5692 [Catenulispora acidiphila DSM 44928] [Translation elongation factor P @ Translation initiation factor 5A]

MASTNELKNGMVLKIDNQLWSVVEFQHVKPGKGPAFVRTKLKAVLSGKVV

DRTFNAGIKVETANVDRRDMQYLYQEGTDFVFMDMDTYDQLHVSGDTVGT

AANYMLEGTMALVATNEGTPLYVELPAAVELVIEYTEPGLQGDRSTGGTK

PAKLETGHEIQVPLFLTTGEKVKVDTRDGSYLGRVSS

>fig|453364.7.peg.169 [Streptococcus pneumoniae CDC0288-04] [Translation elongation factor P @ Translation initiation factor 5A]

MIEASKLKAGMTFETADGKLIRVLEASHHKPGKGNTIMRMKLRDVRTGST

FDTSYRPEEKFEQAIIETVPAQYLYKMDDTAYFMNTETYDQYEIPVVNVE

NELLYILENSDVKIQFYGTEVIGVTVPTTVELTVAETQPSIKGATVTGSG

KPATMETGLVVNVPDFIEAGQKLVINTAEGTYVSRA

>fig|335283.5.peg.1567 [Nitrosomonas eutropha C91] [Translation elongation factor P @ Translation initiation factor 5A]

MKTAQELRVGNVFMLGRDPMVVLKTEFTKSGRNSSVVKMKYKNLLTESPG

EAVYKADDKFDIVVLEKKEVSYSYYASPMYVFMDAEFNQYEVEEETMSDV

LSFLEDGMPCEVVFYNDKPISVEMPNSVVREIIYTEPAVKGDTSGKVMKS

AKIVTGFELPVPLFCEIGDKIEIDTRTREYRSRVK

>fig|197221.1.peg.1293 [Thermosynechococcus elongatus BP-1] [Translation elongation factor P @ Translation initiation factor 5A]

MISSNDFRPGVSIELDGAVWRVVEFLHVKPGKGSAFVRTKLKNVQTGNVI

ERTFRAGETVPQATLEKRTMQHTYKDGEDYVFMDMESYEEARLTPAQVGD

RAKYLKEGMEVNIVKWGEQVLEVELPNSVVLEVVQTDPGVKGDTATGGSK

PAIVETGAQVMVPLFISVGERIRIDTRSDTYLGRE

>fig|360111.3.peg.538 [Campylobacter jejuni subsp. jejuni CF93-6] [Translation elongation factor P @ Translation initiation factor 5A]

MASYSMGDLKKGLKIEIDGIPFKIVEYQHVKPGKGPAFVRIKIKSFIDGK

VLEKTFHAGDKCEAPNLEDKTMQYLYDDGENCQFMDTQTYEQVAISDDDV

GEAKKWMLDGMMVDVLFHNGKAIGVEVPQVVELKIIETAPNFKGDTQGSN

KKPATLETGAVVQIPFHVLEGEVIRVDTVRGEYIERANK

>fig|338969.3.peg.1498 [Rhodoferax ferrireducens DSM 15236] [Translation elongation factor P @ Translation initiation factor 5A]

MKTAQEIRVGNVIMYGKDPMVVLRTEYSRGGRNSSTVRMKLKSLIANFNT

EVVYKADDKLEQVILDKKECTYSYFAEPMYICMDTEYNQYEVEAENMGDS

LNYLEDGMELEVVFYNEKAISVEMPTNVVREITWTEPAVKGDTSGKVLKP

AKISTGFDVGVPIFVAQGDKVEIDTRTGEYRKRV

>fig|257311.1.peg.2260 [Bordetella parapertussis 12822] [Translation elongation factor P @ Translation initiation factor 5A]

MKTAQELRVGNVIMVGKDPLVVQKTEYNKSGRNAAVVKLKFKNLLTGSGS

ESVYKADEKFDVVVLERKECTYSYFGDPMYVFMDEEYNQYEIEADSMGDA

LNYLEEAMPVEVVFYDGRAISVELPTILVREITYTEPAVRGDTSGKVLKP

AKINTGFELSVPLFCAIGDKIEIDTRTNEYRSRVN

>fig|351745.7.peg.1992 [Shewanella sp. W3-18-1] [Translation elongation factor P @ Translation initiation factor 5A]

MKTAHEIRPGNVIMLDGSPWVVQKTETTRSGRNAAIVKLKLKNLLLNSGT

ETTFKGEDKLEDIVLDRLDCTYSYFADPMYVFMDAEYNQYDVEAENLGDA

AAYIVDGMEETCQVTFYDGKAISVEMPTTIVREVIYTEPSARGDTSGKVM

KPATITGGGTVSVADFVKVGDKIEIDTRTGEFKKRV

>fig|315456.3.peg.1049 [Rickettsia felis URRWXCal2] [Translation elongation factor P @ Translation initiation factor 5A]

MKISANSIRTGNILVYNNDLWVVSKTPEHTQPGKGGAYVQVEMKNLKTGT

KRNERFSSSDYLEKAELEQKDYQFLYFEGDDLVLMDTKHFEQINVPKEIL

EEKLPFLTENMIVKVEFYNEKPLNIELPPTVILEINETDPVIKGATATAS

YKPAILENGIKVKVPQYLEVGEKIVVKTDDMTYVERAK

>fig|471854.4.peg.4682 [Dyadobacter fermentans DSM 18053] [Translation elongation factor P @ Translation initiation factor 5A]

MRNGLVIHFNHDLFQVIEFQHVKPGKGNAFVRTKLKSLTSGKVLDNTFSS

GAGITPVRVERHKFQFLYKDEVGYNFMNSETFDQVLIDEKLVTNADLMKE

GQEVEILINTETDAALICELPPFVELTVTYSEPGLKGDTANSPKKAIEVE

TGARIMVPLFIEEGQKIKVDTRTYDYVERVKS

>fig|526218.4.peg.2995 [Sebaldella termitidis ATCC 33386] [Translation elongation factor P @ Translation initiation factor 5A]

MKQAMELRQGSTYRKDNVPYLILKADRHQSTSGKKARAAEMKFKIKDLIS

GKVQEITVLSTEMIDDIILDRNQMQFLYAMDEEYFFMDQETFDQITLSKD

DLGDAVDFLIEEMVIQVLMYEGTPVGVELPNTVVREVTYTEPGLKGDTIG

KATKPATVSTGYILQVPLFIKIGDKIKIDTRTGDYMERAND

>fig|101031.3.peg.1513 [Bacillus B-14905] [Translation elongation factor P @ Translation initiation factor 5A]

MISVNDFRTGLTIVVDGQLYRVLDFQHVKPGKGAAFVRSKLRNLRNGSVN

EKTFRAGEKVEKAQIDNRKMQYLYAQGDDHVFMDLESYEQTELASAAIEY

ELKFLKENMEVHIQSYQGEMLGVELPNTVQLEVTETEPGIKGDTASGGTK

PATLQTGLIVQVPFFVNQGDVLIINTEEGSYVSRA

>fig|320386.3.peg.2570 [Burkholderia mallei 10229] [Translation elongation factor P @ Translation initiation factor 5A]

MKTAQELRVGNVVMIGNDAWVVSKTEYNKSGRNAAVVKMKLKNLLNGGGQ

ESVYKADDKFEVVVLDRKEVTYSYFADPMYVFMDADYNQYEVEAEMMGDA

LNYLEDGMACEVVFYNEKAISVELPTILVREITYTEPAVKGDTSSGKVLK

NAKLATGFELQVPLFCNTGDKIEIDTRTNEYRSRA

>fig|336407.4.peg.172 [Rickettsia bellii RML369-C] [Translation elongation factor P @ Translation initiation factor 5A]

MKISANSIRTGNILVYNNDLWVVSKQPEHTQPGKGGAYVQVEMKNLKTGT

KRNERFSSSDHLEKAELEQKDYQFLYFEDNNIVLMDNQTFEQISVNKEIL

DEKLPFLTENMIVKVEFYNEKPLSIELPATVILEIIETDPVIKGATATAS

YKPATLANGVKVKVPQYLEIGEKIVVKTEDLTYVERSK

>fig|393131.3.peg.1100 [Listeria monocytogenes J2818] [Translation elongation factor P @ Translation initiation factor 5A]

MISVNDFKTGLTIEVDNGIWRVLDFQHVKPGKGAAFVRSKLRNLRTGAIQ

EKTFRGGEKVAKAQIDNRKMAYLYADGTNHVFMDNESYEQIELPEDQIAH

ELKFLKENMEINIIMYQGETIGIDLPNTVELVVTATDPGIKGDTSSGGSK

PATLETGLVVQVPFFVNEGDKLVINTTEAAYVSRA

>fig|87626.3.peg.2116 [Pseudoalteromonas tunicata D2] [Translation elongation factor P @ Translation initiation factor 5A]

MKTAQEMKPNSVALIDGQPWLIQKAEFTKSGRSSAIVKMKLKNLLTGSST

ETVYKVDDKMEPVILDRIEVTYSYTADDLYVFMDADYNQYEINAEDLESV

LPYIVDGMEDICTAVFFEGKVITVDLPTTIIRQVTYTEGSARGDTTGKVM

KPAKLSNGTEIKVPDFCNIDDWIEIDTRTGEYKSRAKAPV

>fig|314262.3.peg.2237 [Roseobacter sp. MED193] [Translation elongation factor P @ Translation initiation factor 5A]

MPKINGNEIRPGNVLEHNDGLWAAVKVDHVKPGKGGAFAQVEMRNLRNGS

KLNERFRSADKVERVRLEQKDQQFLYESDGMLVFMDTETYEQIELPAELL

GDRRPFLQDGMTIVVEFYEEEALNATVPQKVICKIVETEPVVKGQTAANS

FKPAILDNGVKVMVPPFVGQDEMIVVNTDTMDYSERA

>fig|340099.4.peg.1068 [Thermoanaerobacter pseudethanolicus ATCC 33223] [Translation elongation factor P @ Translation initiation factor 5A]

MIAAGDFRKGVTIEVDGQVFTVVDFMHVKPGKGAAFVRTKLKNVMTGAVI

EKTFSPTEKFEEAVIERREMQYLYNDGELYYFMDTETYEQIPLNYDKVED

AIKYIKENMVVTVKFYKGEAFSVEPPTFVELEVVETEPGFRGDTATGGSK

PATVETGAVIQVPLFINVGDKIRIDTRTGEYLERV

>fig|293826.4.peg.2396 [Alkaliphilus metalliredigens QYMF] [Translation elongation factor P @ Translation initiation factor 5A]

MISAGDFRKGVTFVMDGHPYVVIDFQHVKPGKGAAFVRTKYKNLKTGSTR

EEAFNPSDKFPRAHIETKEMQYLYNDSDLYYFMDNETYEQVPLTLVEVED

AIKYLKENDSAIIKFYEGRPFQVEPPIFVELKVIETEPGVKGDTATNVTK

AATVETGAIVYVPVFVNEGNIIKVDTRTGEYMSRV

>fig|203123.1.peg.1258 [Oenococcus oeni PSU-1] [Translation elongation factor P @ Translation initiation factor 5A]

MAIDTSNFKNGLTIEYNKAIWRILSFQHVKPGKGGAFVRSKLKNLRTGAV

NEITFRAGEHMEAADITTRPMQYLYANGDSYVFMDVDTYDQVEIPGDKIK

DSLKFLLENMQVKVTSYKDEILDVELPATVELSVTDTQPSIKGATVNGGG

KPATMETGLVITVPDFVNAGDKLVVNTSDGGSYKERA

>fig|360105.6.peg.608 [Campylobacter curvus 525.92] [Translation elongation factor P @ Translation initiation factor 5A]

MAYSMGDLKKGLKIEIEGVPYKIVEYQHVKPGKGAAFVRAKIKSFVDGKV

LEKTFHAGDKCEQPHLEEKEMQYLYDDGEFCQFMDTTTYEQVAISDEDVG

DVKKWMIDGMMVEILFHNGKAIGVEVPQVVELKIVETPPNFKGDTQGGKK

PATLESGAVVQIPFHVLEGEVIRVDTVRGEYIERANK

>fig|300852.3.peg.1294 [Thermus thermophilus HB8] [Translation elongation factor P @ Translation initiation factor 5A]

MISVTDLRPGTKVKMDGGLWECVEYQHQKLGRGGAKVVAKFKNLETGATV

ERTFNSGEKLEDIYVETRELQYLYPEGEEMVFMDLETYEQFAVPRSRVVG

AEFFKEGMTALGDMYEGQPIKVTPPTVVELKVVDTPPGVRGDTVSGGSKP

ATLETGAVVQVPLFVEPGEVIKVDTRTGEYVGRA

>fig|35793.1.peg.336 [Rickettsia sibirica] [Translation elongation factor P @ Translation initiation factor 5A]

MKISANSIRTGNILVYNNDLWVVSKTPEHTQPGKGGAYVQVEMKNLKTGT

KRNERFSSANYLEKAELEQKDYQFLYFEGDDLVLMDTKHFDQINISKEML

EEKLSFLTENMIVKVEFYNDKPLNIELPPNVILEISETDPVIKGATATAS

YKPAILENGIKVKVPQYLEIGEKIVVKTDDMTYVERAK

>fig|163164.1.peg.1156 [Wolbachia sp. endosymbiont of Drosophila melanogaster] [Translation elongation factor P @ Translation initiation factor 5A]

MAERANDIRPGQVLEHNGGLFLVVGIMHTQPGKGGAYIQAEMKNIKTGAK

HYERFRSDATIRRAILDEEEYVYLFTEGNIVNLMHPSNYEQITINLDLLG

EKKIYLQDNMKIKVVAYQDKIISAHVPDYVTLAVKETESVIKGQTATASY

KPAILENGMRVNVPQFIKEEDKIVVYTPGDSYYERVKE

>fig|360104.4.peg.588 [Campylobacter concisus 13826] [Translation elongation factor P @ Translation initiation factor 5A]

MASYSMGDLKKGLKIEIDGVPYKIVEYQHVKPGKGAAFVRAKIKSFVDGK

VLEKTFHAGDKCEQPHLEEKEMQYLYDDGEFCQFMDTTTYEQVAISDEDV

GDVKKWMIDGMMVEILFHNGNAIGVEVPQVVELKIVETPPNFKGDTQGGK

KPATLESGAVVQIPFHVLEGEVIRVDTVRGEYIERANK

>fig|521096.4.peg.2702 [Tsukamurella paurometabola DSM 20162] [Translation elongation factor P @ Translation initiation factor 5A]

MASTADFKNGLVLNQEGQLWQIIEFQHVKPGKGPAFVRTKLKNVVSGKTV

DKTFNAGVKVDVATVDRRDFDYLYHDGSDYVFMDAETYDQINLPEEKVGE

GAKFLLESMRVQISMHEGEALFVELPISAEFVVQHTDPGLQGDRSTGGTK

PATLETGAEINVPLFINTGDKLKVDTRDGSYLGRVNS

>fig|326297.7.peg.1608 [Shewanella amazonensis SB2B] [Translation elongation factor P @ Translation initiation factor 5A]

MKTAHEIRPGNVIMFNDSPWVVQKTETTRSGRNAAIVKMKLKNLLIDSST

ETTFKGEDKMDVIVLERLDCTYSYFADPMYVFMDSEYNQYDVEAENVGDA

AKYIVDGMEETCQVTFYEGKAISVEMPTTIIREVGYTEPAARGDTSGKVM

KPAKLVGSDIELMVADFVKEGDKIEIDTRTGEFKKRVN

>fig|262698.3.peg.1603 [Brucella abortus biovar 1 str. 9-941] [Translation elongation factor P @ Translation initiation factor 5A]

MKINGNEIRPGNVIEHEGGLWVAVKTNAVKPGKGGAYNQVELKNLINGTK

LNERFRAAESVERVRLEQKDFSFLYEQGEALIFMDTETYEQLELQKDFVG

DRAAFLQDGMMVTVELYEEKPIGIRLPDQVTLAITEADPVVKGQTAASSY

KPAVLENGIRIPVPPFIASGERVIVDTNELTYISRA

>fig|194439.1.peg.159 [Chlorobium tepidum TLS] [Translation elongation factor P @ Translation initiation factor 5A]

MVSISNVSRGAIIRWNGAPHSIESLVHRTPGNLRAFYQASMKNLKTGRNV

EYRFSATEQVDVIVTERKKYQYLYRDGEDYVMMDTETFDQINVPEVAIGP

ASRFLKDSVMVDIVFADDGSILEVELPTFVELEVTETSPASKDDRATSGT

KPAIVETGAEVNVPMFIQTGSIIRIDTRSGEYMERVKK

>fig|483179.3.peg.1666 [Brucella canis ATCC 23365] [Translation elongation factor P @ Translation initiation factor 5A]

MKINGNEIRPGNVIEHEGGLWVAVKTNAVKPGKGGAYNQVELKNLINGTK

LNERFRAAETVERVRLEQKDFSFLYEQGEALIFMDTETYEQLELQKDFVG

DRAAFLQDGMMVTVELYEEKPIGIRLPDQVTLAITEADPVVKGQTAASSY

KPAVLENGIRILVPPFIASGERVIVDTNELTYISRA

>fig|479432.4.peg.2057 [Streptosporangium roseum DSM 43021] [Translation elongation factor P @ Translation initiation factor 5A]

MATTNDLKNGLVLKLDGGELWTVVEFQHVKPGKGGAFVRTKLKNVMSGKV

VDKTFNAGVKVEVANVDKREMQFSYMDGDDFVFMDTETYDMLHVSRTAVG

DAANYLLENMLATVAINEGNVLYIDLPAAVELIIAETEPGLQGDRSTGGT

KPAKLETGAEIKVPLFITTGEKVKVDTRTGDYLGRA

>fig|394221.5.peg.2530 [Maricaulis maris MCS10] [Translation elongation factor P @ Translation initiation factor 5A]

MKINGNEIRPGNVLMHQDTLWVAVKTDHVKPGKGGAFAQVELKNLLDGRK

LNERFRAADKVEKVRLEQKDYTFLFASDDMLTFMDAETYEQIELQTDFVG

EERASYLTDGMTVVVESHEERPIGISLPQHVTLEVADTEPVVKGQTAANS

YKPAILSNGVRSSVPPFVGVGEKIIVATEDGSYVKRAD

>fig|242619.1.peg.498 [Porphyromonas gingivalis W83] [Translation elongation factor P @ Translation initiation factor 5A]

MATTADFRNGMCLEIEGQYYFIVEFLHVKPGKGPAFVRTKLKNVATGRIL

DKTWNSGVKVEEVRIERRPYQYLYQDEMGYNFMHPETFEQITIPGASIDG

VQFLKDGDMVEAMVHATSETVLTCELPPHVKLRVTYTEPGLKGDTATNTL

KPATVETGAEVRVPLFIQEGELIEVDTRDGSYIGRVKE

>fig|242619.1.peg.1097 [Porphyromonas gingivalis W83] [Translation elongation factor P @ Translation initiation factor 5A]

MATTADFRNGMCLEIEGQYYFIVEFLHVKPGKGPAFVRTKLKNVTTGRIL

DKTWNSGVKVEEVRIERRPYQYLYQDEMGYNFMHPETFEQITIPGASIDG

VQFLKDGDMVEVMVHATSETVLTCELPPHVKLRVTYTEPGLKGDTATNTL

KPATVETGAEVRVPLFIQEGELIEVDTRDGSYIGRVKE

>fig|226185.1.peg.265 [Enterococcus faecalis V583] [Translation elongation factor P @ Translation initiation factor 5A]

MIAASDLKAGMTFEQDGKLIKVMEASHHKPGKGNTVMRMKLKDVRTGSTT

DTTMRPDEKVKKAHIDTKPVQYLYSQDDMAIFMDLETYEQYEVPTALIEE

ELKYLLENMEVKIQFYGEEVIGLTLPTTVILRVAETQPSIKGATVTGSGK

PATMETGLVVNVPDFVEADELLEINTAEGTYLKRAK

>fig|391735.5.peg.2996 [Verminephrobacter eiseniae EF01-2] [Translation elongation factor P @ Translation initiation factor 5A]

MSRAAPPQNMHGHGTPSSLGAAGRRIGRPQPKQLAMKIAQEIRAGNVIMH

GKDPMVVLKTEYARGGRGAATVRMKLKSLIANFGTEIVLRADDKIDNVIL

DKKECTYSYFADPMYVCMDAEYNQYEVEAGNMGDALNYLEDGMALEVVFY

DGKAISVELPTSVEREITWTEPAVKGDTSGKVMKPAKIATGFEVPVPLFV

AQGDRIEIDTRTGEYRRRV

>fig|399739.6.peg.2069 [Pseudomonas mendocina ymp] [Translation elongation factor P @ Translation initiation factor 5A]

MKTAQEMRVNSVALIDGQPWLIQKAEFTKSGRNSAIVKMKLKNLLNGSKT

ETVYKADDKMEPVILERKEVNLSYISGEDYVFMDPEYNSYELRAEDLESV

LPFIEEGMTDVCEAVFFEGKVISVDLPTTIVRQVVYTENAARGDTSGKVM

KPAKLRNGTEIKVAEFVDIDDWIEIDTRDGSYKGRTQAPA

>fig|273121.1.peg.1185 [Wolinella succinogenes DSM 1740] [Translation elongation factor P @ Translation initiation factor 5A]

MAYSMSDLKKGLKVEIEGVPYKIVEYQHVKPGKGAAFVRVKMKSFFDGRV

LEKTFHAGDKCEEPNLQEKNMQYLYHDGDHFQFMDVESYEQIALSDEQVG

DVAKWMTDGMTVSILFHNNKAISVDVPQVVELKITETPPNFKGDTSSGSK

KPATLETGAVIQIPYHVLEGDVVRVNTELGEYIEKVK

>fig|93061.3.peg.1531 [Staphylococcus aureus subsp. aureus NCTC 8325] [Translation elongation factor P @ Translation initiation factor 5A]

MISVNDFKTGLTISVDNAIWKVIDFQHVKPGKGSAFVRSKLRNLRTGAIQ

EKTFRAGEKVEPAMIENRRMQYLYADGDNHVFMDNESFEQTELSSDYLKE

ELNYLKEGMEVQIQTYEGETIGVELPKTVELTVTETEPGIKGDTATGATK

SATVETGYTLNVPLFVNEGDVLIINTGDGSYISRG

>fig|425104.3.peg.2073 [Shewanella sediminis HAW-EB3] [Translation elongation factor P @ Translation initiation factor 5A]

MKTAHEIRPGNVIMLNGSPWVVQKTETTRSGRNAAIVKMKLKNVLLDSTT

ETTFKGEDKMEDIILERLDCTYSYFADPMYVFMDAEYNQYDVESGNLGDA

AAYITDGMEEICQVTFYEGKAISVELPTTIVREVTYTEPSARGDTSGKVM

KPATIAGGATLSVADFIKTGEMIEIDPRTGEFKKRA

>fig|471852.4.peg.4493 [Thermomonospora curvata DSM 43183] [Translation elongation factor P @ Translation initiation factor 5A]

MTTTNDLKNGMTLNLDGQLWTVLEFQHVKPGKGGAFVRTKLKNVLSGKVV

DKTFNAGVKVEVASVDKREMQYLYREGEDFVFMDTETYDQPHIPAATVGD

AANYLLPEQTAVVAFHNETPLYVELPAAVVLEVTHTEPGLQGDRSTGGTK

PATLETGAEIQVPLFITTGEKVKVDTRTGEYLGRA

>fig|243365.1.peg.1378 [Chromobacterium violaceum ATCC 12472] [Translation elongation factor P @ Translation initiation factor 5A]

MKTAQELRAGNVFMVGSDPMVVQKAEFSKSGRNASVVKMKMKNLLTGAGS

EAVYRADDKFDVVVLDRKDCTYSYFADPMYVFMDTEFNQYEVEADNLGDT

INYIVDGMEDVCQVTFYDGKAISVELPTTVIREVEYTEPAVRGDTSGKVL

KPARLVGTTFEVQVPAFVNTGEKIEIDTRTNEFKKRA

>fig|393125.3.peg.1617 [Listeria monocytogenes FSL R2-503] [Translation elongation factor P @ Translation initiation factor 5A]

MISVNDFKTGLTIEVDNGIWRVLDFQHVKPGKGAAFVRSKLRNLRTGAIQ

EKTFRGGEKVAKAQIDNRKMAYLYADGTNHVFMDNESYEQIELPEDQIAH

ELKFLKENMEINIIMYQGETIGIDLPNTVELVVTATDPGIKGDTSSGGSK

PATLETGLVVQVPFFVNEGDKLVINTTEAAYVSRA

>fig|95662.4.peg.369 [Ureaplasma urealyticum serovar 8] [Translation elongation factor P @ Translation initiation factor 5A]

MATIIQAKDLRAGHTFLYKGSIYQVIENSFNKTAMREGIVKCKVKNLRTG

AITVEVLTGEKVEQAIIEKSKMTFSYDDGSGYVFMDNETYEQISIPYNQL

SWEKNFIEEGTEVSVMRYDGELMGVSLPDQLVVTIVEAEEAVQGNSVQNA

TKRAWLASKWEFQVPQFIKSGEKVIINPSNGQYVGRAK

>fig|216595.1.peg.5146 [Pseudomonas fluorescens SBW25] [Translation elongation factor P @ Translation initiation factor 5A]

MKTGKELKPGTVIRLENDPWLVQKAEFTKSGRNSAIMKTKLKNLLTGYKT

EIVYSADDKLDDVILDRKEATLSFISGDTYTFMDTTDYTMYELNAEDIEA

VLPFIEEGMEDVCEAIFFEERLVSVELPTTIVRKVAYTEGSARGDTSGKV

MKPAKLSNGTELQVADFIEIDDLIEIDTREGGSYKGRAKK

>fig|59931.3.peg.1825 [Synechococcus sp. WH 7805] [Translation elongation factor P @ Translation initiation factor 5A]

MAQPYDPLNRSAGMISSNDFRTGTTIELDGAVWRVVEFLHVKPGKGSAFV

RTKLKAVQSGNVVEKTFRAGEMLPQAILEKATLQHTYMEGEDYVFMDMGT

YEETRLSAKQIGESRKYLKEGMEVNVVSWNDKPLEVELPNSVVLEIKETD

PGVKGDTATGGTKPAILETGAQVMVPLFLSIGEKIKVDTRNDTYLGRENS

>fig|257363.1.peg.221 [Rickettsia typhi str. Wilmington] [Translation elongation factor P @ Translation initiation factor 5A]

MKISANSIRTGNILVYNNDLWVVSKTPEHTQPGKGGAYVQVEMKNLKTGT

KRNGRFSSSDYLEKAELEQKDCQFLYFEGNNLVLMDTKHFDQINVPKEIL

EAKLPFLTENMIVKVEFYNDKPLTIVLPPTVILAISETDPVIKGATVTSS

YKPAILENGIKVKVPQYLAIGEKIVVKTDDMTYVERAK

>fig|227882.1.peg.6861 [Streptomyces avermitilis MA-4680] [Translation elongation factor P @ Translation initiation factor 5A]

MASTNDLKNGLVLKLDGGQLWSVVEFQHVKPGKGPAFVRTKLKNVLSGKV

VDKTFNAGVKVETATIDKRDMQFSYMDGEYFVFMDMDTYDQLMVDRKSVG

DAANFLIEGFTATVAQHEGEVLFVELPAAVELVIQETEPGVQGDRSTGGT

KPATLETGHQIQVPLFITTGEKIKVDTRTSDYLGRVNS

>fig|95664.5.peg.92 [Ureaplasma urealyticum serovar 10] [Translation elongation factor P @ Translation initiation factor 5A]

MATIIQAKDLRAGHTFLYKGSIYQVIENSFNKTAMREGIVKCKVKNLRTG

AITVEVLTGEKVEQAIIEKSKMTFSYDDGSGYVFMDNETYEQISIPYNQL

SWEKNFIEEGTEVSVMRYDGELMGVSLPDQLVVTIVEAEEAVQGNSVQNA

TKRAWLASKWEFQVPQFIKSGEKVIINPSNGQYVGRAK

>fig|420246.5.peg.2247 [Geobacillus thermodenitrificans NG80-2] [Translation elongation factor P @ Translation initiation factor 5A]

MISVNDFRTGLTIEVDGEIWRVLEFQHVKPGKGAAFVRSKLRNLRTGAIQ

ERTFRAGEKVNRAQIDTRKMQYLYANGDLHVFMDMETYEQIELPAKQIEY

ELKFLKENMEVFIMMYQGETIGVELPNTVELKVVETEPGIKGDTASGGSK

PAKLETGLVVQVPFFVNEGDTLIINTADGTYVSRA

>fig|164756.6.peg.2235 [Mycobacterium sp. MCS] [Translation elongation factor P @ Translation initiation factor 5A]

MASTADFKNGLVLQIDGQLWQIVEFQHVKPGKGPAFVRTKLKNVVSGKVV

DKTYNAGVKVETATVDRRDATYLYRDGSDFVFMDSEDYEQHPLPESLVGR

AADFLLESMPVQIAFHDGVPLYLELPVTVELLVASTEPGLQGDRSSAGTK

PATMETGAEIQVPLFINTGDKLKVDSRDGSYLGRVNA

>fig|95665.5.peg.548 [Ureaplasma urealyticum serovar 11] [Translation elongation factor P @ Translation initiation factor 5A]

MATIIQAKDLRAGHTFLYKGSIYQVIENSFNKTAMREGIVKCKVKNLRTG

AITVEVLTGEKVEQAIIEKSKMTFSYDDGSGYVFMDNETYEQISIPYNQL

SWEKNFIEEGTEVSVMRYDGELMGVSLPDQLVVTIVEAEEAVQGNSVQNA

TKRAWLASKWEFQVPQFIKSGEKVIINPSNGQYVGRAK

>fig|290317.7.peg.250 [Chlorobium phaeobacteroides DSM 266] [Translation elongation factor P @ Translation initiation factor 5A]

MTSISNVSKGSIIRFKGEPHIIESLIHRTPGNLRAFYQANMKNLKTGRNV

EFRFSASESVDVIVTERKPYQYLYKDGTDFVMMDSGTFDQINVPEITLGT

SSRFLKDGITVVIVFSDDGSILDVEMPTFVEVEVTETSPTTKDDRATSGT

KPAIVETGAEVGVPMFIQTGSIIRVDTRTGEYIERVKK

>fig|395019.3.peg.2293 [Burkholderia multivorans ATCC 17616] [Translation elongation factor P @ Translation initiation factor 5A]

MKTAQELRVGNVVQIGSDAWVISKTEYNKSGRNAAVVKLKMKNLLTNAGQ

ESVYKADDKFDVVMLDRKEVTYSYFADPMYVFMDADYNQYEVEAEMMGDA

LNYLEDGMACEVVFYNEKAISVELPTILVREITYTEPAVKGDTSSGKVLK

NAKLATGFELQVPLFCNTGDKIEIDTRTHEYRSRA

>fig|262316.1.peg.1097 [Mycobacterium avium subsp. paratuberculosis str. k10] [Translation elongation factor P @ Translation initiation factor 5A]

MASTADFKNGLVLVIDGQLWQIVEFQHVKPGKGPAFVRTKLKNVLSGKVV

DKTYNAGVKVETATVDRRDTTYLYRDGSDFVFMDSQDYEQHPLPESLVGD

AARFLLEGMPVQVAFHNGSPLYIELPVSVEMEVTHTEPGLQGDRSSAGTK

PATVETGAEIQVPLFINTGDKLKVDTRDGSYLGRVNA

>fig|138119.3.peg.2377 [Desulfitobacterium sp. Y51] [Translation elongation factor P @ Translation initiation factor 5A]

MISSNEFKTGLTIEVDNDVYTIIEFQHVKPGKGAAFVRTKLKNVKTGGIT

ERKFNAGEKVPKAHVERREMQYLYKDGDHFVAMDNETYEQTSLTEAQIGD

GVKYLKENMNLGILFFNGTVIGVDLPNTVILEVAHTEPGVRGDTATGGSK

PATLETGAVVQVPFFVNEGEKLIIDTRTGNYVQRA

>fig|115713.1.peg.877 [Chlamydophila pneumoniae CWL029] [Translation elongation factor P @ Translation initiation factor 5A]

MVRVSTSEFRVGLRIEIDGQPYLILQNDFVKPGKGQAFNRIKVKNFLTGR

VIERTYKSGESVETADIVERSMRLLYTDQEGATFMDDETFEQEVVFWEKL

ENIRQWLLEDTIYTLVLYNGDVVAVEPPIFMELSIAETAPGVRGDTASGR

VLKPAVTNTGAKIMVPIFIDEGELVKVDTRTGSYESRVSK

>fig|106370.11.peg.3168 [Frankia sp. Ccl3] [Translation elongation factor P @ Translation initiation factor 5A]

MATTNDLKNGMTLDIDGVLWNVVGFQHVKPGKGGAFVRTTLKNVLTGKVV

DRTFNAGVKVDVATVDRREMTYLYRDGADFVFMDSESYDQIPIPPDVVGG

TADYMLENTVATVALHDGAPLYVELPASVELTISQTDPGVQGDRSTGGTK

PATLETGATINVPLFITSGEKVKVDTRDGRYLGRVTS

>fig|193567.1.peg.293 [Streptococcus pyogenes SSI-1] [Translation elongation factor P @ Translation initiation factor 5A]

MIEASKLKAGMTFEAEGKLIRVLEASHHKPGKGNTIMRMKLRDVRTGSTF

DTTYRPDEKFEQAIIETVPAQYLYKMDDTAYFMNTDTYDQYEIPVANVEQ

ELLYILENSDVKIQFYGSEVIGVTVPTTVELTVAETQPSIKGATVTGSGK

PATLETGLVVNVPDFIEAGQKLIINTAEGTYVSRA

>fig|393128.3.peg.1142 [Listeria monocytogenes F6900] [Translation elongation factor P @ Translation initiation factor 5A]

MISVNDFKTGLTIEVDNGIWRVLDFQHVKPGKGAAFVRSKLRNLRTGAIQ

EKTFRGGEKVAKAQIDNRKMAYLYADGTNHVFMDNESYEQIELPEDQIAH

ELKFLKENMEINIIMYQGETIGIDLPNTVELVVTATDPGIKGDTSSGGSK

PATLETGLVVQVPFFVNEGDKLVINTTEAAYVSRA

>fig|282459.1.peg.1453 [Staphylococcus aureus subsp. aureus MSSA476] [Translation elongation factor P @ Translation initiation factor 5A]

MISVNDFKTGLTISVDNAIWKVIDFQHVKPGKGSAFVRSKLRNLRTGAIQ

EKTFRAGEKVEPAMIENRRMQYLYADGDNHVFMDNESFEQTELSSDYLKE

ELNYLKEGMEVQIQTYEGETIGVELPKTVELTVTETEPGIKGDTATGATK

SATVETGYTLNVPLFVNEGDVLIINTGDGSYISRG

>fig|223283.1.peg.1728 [Pseudomonas syringae pv. tomato str. DC3000] [Translation elongation factor P @ Translation initiation factor 5A]

MKTGKELKPGTVIRLENDPWLVQKAEFTKSGRNSAIMKTKLKNLLTGYKT

EIVYSADDKLDDVILDRKEATLSFISGDTYTFMDTTDYTMYELNAEDIES

VLPFVEEGMTDVCEAVFFDERLVSVELPTTIVRQVDYTEGSARGDTSGKV

MKPAKLKNGTELSVADFIEIGDMIEIDTREGGSYKGRAK

>fig|388919.8.peg.400 [Streptococcus sanguinis SK36] [Translation elongation factor P @ Translation initiation factor 5A]

MIEASKLKAGMTFETADGKLIRVLEASHHKPGKGNTIMRMKLRDVRTGST

FDTSYRPEEKFEQAIIETVPAQYLYQMDDTAYFMNTETYDQYEIPVVNVE

EELKFILENSDVKIQFYGTEVIGVTVPTTVELVVTDTQPSIKGATVTGSG

KPATLETGLVVNVPDFIEVGQKLIINTAEGTYVSRA

>fig|205918.4.peg.4065 [Pseudomonas syringae pv. syringae B728a] [Translation elongation factor P @ Translation initiation factor 5A]

MKTGKELKPGTVIRLENDPWLVQKAEFTKSGRNSAIMKTKLKNLLTGYKT

EIVYSADDKLDDVILDRKEATLSFISGDTYTFMDTSDYTMYELNAEDIES

VLPFVEEGMTDVCEAVFFEDRLVSVELPTTIVRQVDYTEGSARGDTSGKV

MKPAKLKNGTELSVADFIEIGDMIEIDTREGGSYKGRAK

>fig|521.1.peg.214 [Bordetella avium] [Translation elongation factor P @ Translation initiation factor 5A]

MKTAQELRVGNVVMVGKDPLVVQKTEYNKSGRNAAVVKLKFKNLLTGSAS

ESVYKADEKFDIVMLERKECTYSYFGDPMYVFMDADYNQYEIEADSMGDA

LNYLEEAMPVEVVFYDGRAISVELPTILVREITYTEPAVRGDTSGKVLKP

AKINTGYELQVPLFCAIGDKIEIDTRTNEYRSRVN

>fig|335992.3.peg.1127 [Pelagibacter ubique HTCC1062] [Translation elongation factor P @ Translation initiation factor 5A]

MKLYASEIRVGMLIEYKNDLWQVLKTQHVKPGKGGAFAQVEMKSVNKNTK

LNERFRSSESVEKASLDETKFNYLYSDEIDYYFMDPKSYEQINIKKETIG

EKGKMLTENLEVSISFYNEKPLTVELPNQVTCTVDTTDVALKGQTVSSSY

KPATLDNGVNIQVPPFIESGDKIIVDTRTMEYVKKI

>fig|233413.1.peg.2539 [Mycobacterium bovis AF2122/97] [Translation elongation factor P @ Translation initiation factor 5A]

MATTADFKNGLVLVIDGQLWTITEFQHVKPGKGPAFVRTKLKNVLSGKVV

DKTFNAGVKVDTATVDRRDTTYLYRDGSDFVFMDSQDYEQHPLPEALVGD

AARFLLEGMPVQVAFHNGVPLYIELPVTVELEVTHTEPGLQGDRSSAGTK

PATLQTGAQINVPLFINTGDKLKVDSRDGSYLGRVNA

>fig|783.1.peg.295 [Rickettsia rickettsii] [Translation elongation factor P @ Translation initiation factor 5A]

MYNNDLWVVSKTPEHTQPGKGGAYVQVEMKNLKTGTKRNERFSSADYLEK

AELEQKDYQFLYFEGDDLVLMDTKHFDQINISKEMLEEKLSFLTENMIVK

IEFYNDKPLNIELPPTVILEISETDPVIKGATATASYKPAILENGIKVKV

PQYLEIGEKIVVKTDDMTYVERAK

>fig|206672.1.peg.64 [Bifidobacterium longum NCC2705] [Translation elongation factor P @ Translation initiation factor 5A]

MAQTTNDIKNGSVLNLDGQLWTVMKFQHVKPGKGPAFVRTTIKNVLSGKI

VDKTFNAGMKMEFETVDNRTLQYSYEDGDNFVFMDMTTYDQIMVPKTLLG

DKAKFLLEGTDCLVSFHDGTPLSVDLPGSVVLTITHTEPGLQGNRSNAGT

KPATVETGAEIQVPLFINEGDRVKINTEDGSYTGRENN

>fig|443906.9.peg.1814 [Clavibacter michiganensis subsp. michiganensis NCPPB 382] [Translation elongation factor P @ Translation initiation factor 5A]

MASTADIKNGVVLNMDGQLWTVIEFQHVKPGKGGAFVRTKVKNVMSGKVV

DRTFNAGAKIETETVDRRDFQYLYADGENFVFMDTSDYDQITLSAAQVGD

AKNFMLENQDVTVALHNGEGLYVELPASVVLTITYTEPGLQGDRSTGGTK

PATVETGHQIQVPLFLEQGTRVKVDTRTGDYLGRVTD

>fig|446465.4.peg.2417 [Brachybacterium faecium DSM 4810] [Translation elongation factor P @ Translation initiation factor 5A]

MATTNDLKNGMVLVLDQQLWSVVEFQHVKPGKGPAFVRTKLKNIMSGKGV

DKTFNAGVKVETATVDRRDMQFSYIDGDMYVFMDTSNWEQTSLTAEIVGE

AKDFMAENQDVVVAFHEGEALFVELPANVVLTIEETEPGLQGDRSSGGTK

PARLETGREIQVPLFINIGDRVRVSTAGGEYKDRA

>fig|382245.6.peg.1013 [Aeromonas salmonicida subsp. salmonicida A449] [Translation elongation factor P @ Translation initiation factor 5A]

MKTAQEIRAGNVVMIGTEPMVVQKAEFNKSGRNSAVVKMKLKGLLNGSAT

ETVFKADDKLDVVQLERKECTYSYFSDPLYVFMDTEYNQYDVEKDNLGDV

LNYLVDGMEDICEVTFYNEKAISVELPTTIVREVEYTEPAARGDTSGKVT

KPARLKGTAYELAVAAFVEIGDKIEIDSRTGEFKRRLS

>fig|469383.4.peg.5274 [Conexibacter woesei DSM 14684] [Translation elongation factor P @ Translation initiation factor 5A]

MISTNQLKNGNHIEVDGTIFKVVEFQHVKPGKGPAFVRTKLRRTTDGNVI

DKTFRAGEKFRSVRTEVRKMQFLYSDDADAHFMDVESFEQTAIPTGLLRE

ALQWTRASDEVDLLSIDGVPADIQLPSAVDLEVTETEPGLRGDTASGGGT

KPATLETGAKIQVPLFVNIGDKVRVDTRSGDYVSRA

>fig|373153.25.peg.426 [Streptococcus pneumonia pneumoniae D39] [Translation elongation factor P @ Translation initiation factor 5A]

MIEASKLKAGMTFETADGKLIRVLEASHHKPGKGNTIMRMKLRDVRTGST

FDTSYRPEEKFEQAIIETVPAQYLYKMDDTAYFMNTETYDQYEIPVVNVE

NELLYILENSDVKIQFYGTEVIGVTVPTTVELTVAETQPSIKGATVTGSG

KPATMETGLVVNVPDFIEAGQKLVINTAEGTYVSRA

>fig|485917.5.peg.3034 [Pedobacter heparinus DSM 2366] [Translation elongation factor P @ Translation initiation factor 5A]

MAKASDIKNGNILRFNGELVQVEEFLHRTPGNLRAFYQARMRNVKTGKLV

EYRFRVDEEVEICRVETSDYQYLYEDGDALVVMDNNTYEQFNIPKLLFGK

SVRFLKEGMNVIIAFESDEPIMAQTPSHVELEITYSEPAVKGDTSTNALK

YATVETGVEIKVPMFINQGDKVKIDTRTGDYIERVK

>fig|360110.3.peg.1630 [Campylobacter jejuni subsp. jejuni 84-25] [Translation elongation factor P @ Translation initiation factor 5A]

MASYSMGDLKKGLKIEIDGIPFKIVEYQHVKPGKGPAFVRIKIKSFIDGK

VLEKTFHAGDKCEAPNLEDKTMQYLYDDGENCQFMDTQTYEQVAISDDDV

GEAKKWMLDGMMVDVLFHNGKAIGVEVPQVVELKIIETAPNFKGDTQGSN

KKPATLETGAVVQIPFHVLEGEVIRVDTVRGEYIERANK

>fig|367830.3.peg.2330 [Staphylococcus aureus subsp. aureus USA300] [Translation elongation factor P @ Translation initiation factor 5A]

MISVNDFKTGLTISVDNAIWKVIDFQHVKPGKGSAFVRSKLRNLRTGAIQ

EKTFRAGEKVEPAMIENRRMQYLYADGDNHVFMDNESFEQTELSSDYLKE

ELNYLKEGMEVQIQTYEGETIGVELPKTVELTVTETEPGIKGDTATGATK

SATVETGYTLNVPLFVNEGDVLIINTGDGSYISRG

>fig|204669.6.peg.1069 [Acidobacteria bacterium Ellin345] [Translation elongation factor P @ Translation initiation factor 5A]

MAGLIDAISVKRKMFFEHEGVPYHCLDAEVNTPTARGGQTLVRLKMRNLL

TRAVFDKTFKASDKFKEPDLEMVPASFLYSDADGFYFMDQETFETHTLRE

DMIGDAADLLTEGLIVQLSKYNGNPIGLEMPTFVELTVTQTEPGMRDSGA

GSVTKAATLETGVEIRVPLFIKEGEKVKVSTETRGFAGRA

>fig|204669.6.peg.3612 [Acidobacteria bacterium Ellin345] [Translation elongation factor P @ Translation initiation factor 5A]

MPCAPFPNTGPDGRIECLAFLSAARQSFSRRPAQTQNLSLGVRVFMAIPA

TQMRPGMVIKHNNDLHSVFSVEHRTPGNLRAFIQAKLRNLRTGAMFEHRF

RSGDPIDKITVDEEKMEYLYQEGDAYVFMNTENYEQLYLNHDVLGDAADY

LTPNIQISVEFFDGKPVGVGLPQTVELTVVETEPGLKSATASSVAKPAKT

ETGLVVYVPPFINEGDKIRVDTSEGAYLGRA

>fig|272947.1.peg.227 [Rickettsia prowazekii str. Madrid E] [Translation elongation factor P @ Translation initiation factor 5A]

MKISANSIRTGNILVYNNDLWVVSKTPEHTQPGKGGAYVQVEMKNLKTGT

KRNDRFSSSDYLEKAELEQKDCQFLYFEGNNLVLMDTKHFDQINVPKEIL

EAKLPFLTENMIVKVEFYNDKPLTIVLPPTVILAISETDPVIKGATVTSS

YKPAILENGIKVKVPQYLAIGEKIVVKTDDMTYVERAK

>fig|216600.3.peg.342 [Streptococcus pneumoniae 23F] [Translation elongation factor P @ Translation initiation factor 5A]

MIEASKLKAGMTFETADGKLIRVLEASHHKPGKGNTIMRMKLRDVRTGST

FDTSYRPEEKFEQAIIETVPAQYLYKMDDTAYFMNTETYDQYEIPVVNVE

NELLYILENSDVKIQFYGTEVIGVTVPTTVELTVAETQPSIKGATVTGSG

KPATMETGLVVNVPDFIEAGQKLVINTAEGTYVSRA

>fig|354.1.peg.3006 [Azotobacter vinelandii] [Translation elongation factor P @ Translation initiation factor 5A]

MGGQAVALAYLSAGGRRPPGQAGSLPFPLSRGFVAGRRGRPAGALARLER

RGGDGPGLVGAGGSVAGAARACRALVRPTGRPAGSGRGTGAVLRGLAMIR

GLFFVIHPNGYPYENRSRIPCRPGSHDQWRAPGSSQKAEFNKSGRNAAVV

KMKLKNLLNGQATETVYKADDKFEPVILERKEVTYSYFADPMYVFMDNEF

NQYEIEKDDLGDAYNFIEDGMQDVCEAVFYNDRVISIELPTTIVRQISYT

EPAVRGDTSGKVMKVARLNSGYELRVAEFCDIGDYIEIDTRTFEYKSRAK

A

>fig|478801.4.peg.705 [Kytococcus sedentarius DSM 20547] [Translation elongation factor P @ Translation initiation factor 5A]

MPVHRGHTNSPTTQKDFPVATTNDLKNGMVLKLEGQLWQVQEFQHVKPGK

GPAFVRTKIKNVLSGKTVDKTFNAGTKVETATVDRRDMQYLYNDGDSFIF

MDTKDYEQLPIPAEIVGDASRFMLENQEVQVSTHEGVPLFIELPPSVELR

ITHTDPGLQGDRSTGGTKPATLETGAEIQVPLFLESDTLVKVDTRDGSYI

SRVS

>fig|350701.3.peg.2307 [Burkholderia dolosa AUO158] [Translation elongation factor P @ Translation initiation factor 5A]

MKTAQELRVGNVVQIGSDAWVVSKTEYNKSGRNAAVVKLKMKNLLTNAGQ

ESVYKADDKFDVVVLDRKEVTYSYFADPMYVFMDADYNQYEVEAEMMGDA

LNYLEDGMACEVVFYNEKAISVELPTILVREITYTEPAVKGDTSSGKVLK

NAKLATGFELQVPLFCNTGDKIEIDTRTHEYRSRA

>fig|95660.5.peg.448 [Ureaplasma parvum serovar 6] [Translation elongation factor P @ Translation initiation factor 5A]

MATIIQAKDLRAGHTFLYKGNIYQVIENSFNKTAMREGIVKCKVKNLRTG

AITIEVLTGEKLEQAVIEKSKMTFSYDDGSGYVFMDNDTYEQISIPYSQL

SWEKNFIEEGTEVSVMRYDGELMGVSLPDQLVVTIIEAEEAVQGNSVQNA

TKRAWLESKWEFQVPQFIKSGEKVIINPSNGQYVGRAK

>fig|504472.5.peg.283 [Spirosoma linguale DSM 74] [Translation elongation factor P @ Translation initiation factor 5A]

MATTADIRNGLVLNYNNDLFQITEFQHVKPGKGAAFVRTKLKSLTTGRVI

DNTFNSGATIYPVRVERRKFQYLYKDEAGFNFMDQESFDQINLDEKLVDG

ADLMKEGQEVEILINADSDTPLSCELPPFVELEVTYAEPGIKGDTANSPK

KRVEVESGAKIMVPLFIESGQKIRVDTRTRDYVERVK

>fig|526227.4.peg.3137 [Meiothermus silvanus DSM 9946] [Translation elongation factor P @ Translation initiation factor 5A]

MISVTDLRSGTKVQMDGGLWQCVEYQHQKIGRGGAKVVAKFRNLETGVTV

EKSFNSGEKLQDIYVETKDLQYLYSEGDELVLMDLETYEQFHVPRAITDA

AKFLKEGMTVVGEMYQGRPLSITPPFTVELKIIDTPPGVRGDTVSGGTKP

ATLETGAVVQVPLFVNPGETIKVDTRTGEYISRA

>fig|315277.3.peg.691 [Chlamydia trachomatis A/HAR-13] [Translation elongation factor P @ Translation initiation factor 5A]

MVRVSTSEFRVGLRVKIDGQPYVILQNDFVKPGKGQAFNRIKVKNFLTGR

VIEKTFKSGESIETADVREQQMRLLYTDQEGATFMDDETFEQELIFWDKL

ENVRQWLLEDTIYTLVLYNGDVISVEPPIFMELTIAETAPGVRGDTASGR

VLKPATTNTGAKIMVPIFIEEGEVVKVDTRTGSYESRVSK

>fig|453361.3.peg.70 [Streptococcus pneumoniae CDC1087-00] [Translation elongation factor P @ Translation initiation factor 5A]

MIEASKLKAGMTFETADGKLIRVLEASHHKPGKGNTIMRMKLRDVRTGST

FDTSYRPEEKFEQAIIETVPAQYLYKMDDTAYFMNTETYDQYEIPVVNVE

NELLYILENSDVKIQFYGTEVIGVTVPTTVELTVAETQPSIKGATVTGSG

KPATMETGLVVNVPDFIEAGQKLVINTAEGTYVSRA

>fig|383372.4.peg.1471 [Roseiflexus castenholzi DSM 13941] [Translation elongation factor P @ Translation initiation factor 5A]

MATTSDLRTNMIIRHNGQLHRVMEFYHHAPGNWRAMVIMKLKNIETGKTI

EERVRAGSEIEIVRVEKRPMQFLYREGDIYHFMDTETFEQIEVAEDLIGE

PAKFLKENEMADILFYDDNKILGVEPPLFVTLQVTEASVAVRGDTATNVN

KQVTLETGAVISVPAFVNQGDYVRVDTRTGEYIERIK

>fig|426430.6.peg.1393 [Staphylococcus aureus subsp. aureus str. Newman] [Translation elongation factor P @ Translation initiation factor 5A]

MISVNDFKTGLTISVDNAIWKVIDFQHVKPGKGSAFVRSKLRNLRTGAIQ

EKTFRAGEKVEPAMIENRRMQYLYADGDNHVFMDNESFEQTELSSDYLKE

ELNYLKEGMEVQIQTYEGETIGVELPKTVELTVTETEPGIKGDTATGATK

SATVETGYTLNVPLFVNEGDVLIINTGDGSYISRG

>fig|359787.3.peg.1339 [Staphylococcus aureus subsp. aureus JH1] [Translation elongation factor P @ Translation initiation factor 5A]

MISVNDFKTGLTISVDNAIWKVIDFQHVKPGKGSAFVRSKLRNLRTGAIQ

EKTFRAGEKVEPAMIENRRMQYLYADGDNHVFMDNESFEQTELSSDYLKE

ELNYLKEGMEVQIQTYEGETIGVELPKTVELTVTETEPGIKGDTATGATK

SATVETGYTLNVPLFVNEGDVLIINTGDGSYISRG

>fig|315730.5.peg.4608 [Bacillus weihenstephanensis KBAB4] [Translation elongation factor P @ Translation initiation factor 5A]

MISVNDFRTGLTISVDNALWQVMDFQHVKPGKGAAFVRSKLRNLRTGSVQ

EKTFRAGEKVEKAHIENRRMQYLYASGESHVFMDNETYEQIELGENQIER

ELKFLKENMGVSIMTYQDEVLGVELPNTVELTVSETEPGIKGDTASNVTK

PAKLETGLVVQVPIFINEGEMLIINTGEGKYVSRA

>fig|95668.5.peg.510 [Ureaplasma parvum serovar 14] [Translation elongation factor P @ Translation initiation factor 5A]

MATIIQAKDLRAGHTFLYKGNIYQVIENSFNKTAMREGIVKCKVKNLRTG

AITIEVLTGEKLEQAVIEKSKMTFSYDDGSGYVFMDNDTYEQISIPYSQL

SWEKNFIEEGTEVSVMRYDGELMGVSLPDQLVVTIIEAEEAVQGNSVQNA

TKRAWLESKWEFQVPQFIKSGEKVIINPSNGQYVGRAK

>fig|321956.5.peg.1149 [Lactobacillus delbrueckii subsp. bulgaricus ATCC BAA-365] [Translation elongation factor P @ Translation initiation factor 5A]

MTMISVNEFKNGLTIEYNNDLWRIVEFQHVKPGKGGAFVRSKLKSLRTGA

VQEYTFRSTAKVETADIQTRQMQYLYNDGSSYVFMDTATYEQLEVPNAQI

DQEAKYLKENMIVNIISHNGETLGLDLPNTVDLEVVETEPGIRGDTSSGG

GKPATMETGLVVTVPFFINVGDVLTINTSDGSYVSRSK

>fig|479437.4.peg.458 [Eggerthella lenta DSM 2243] [Translation elongation factor P @ Translation initiation factor 5A]

MAISTADFKNGMCIEFNGKLCTIVEFQHVKPGKGSAFVRTKLRDIKTGRI

IDNTFNAGVKVESVRLETKKLQYLYNDGADFNFMDNDTFEQMAISTETVG

DAAKWLKENDEASLLYAGDELISIEPQMFVELEVTHTEPGFKGDTATNTT

KPATLETGVEVQVPTFVEIGDVLQIDTRDGRFIKRV

>fig|292415.3.peg.285 [Thiobacillus denitrificans ATCC 25259] [Translation elongation factor P @ Translation initiation factor 5A]

MKIAQELRAGNVVMIGKDPMVVQKAEFSKSGRNASVVKMKLKNLLTGAGM

ESVYRADDKFDTVTLDRKECTYSYFADPLYVFMDSDYNQYEVEGDNLGDA

LNYLDDGMPVEVVFYEGKAISVEMPTTVIREVEYTEPAVRGDTSGKVMKP

ARIKPTGFELPVAAFVEIGDMIEIDTRTNEFKRRAN

>fig|457570.7.peg.1734 [Natranaerobius thermophilus JW/NM-WN-LF] [Translation elongation factor P @ Translation initiation factor 5A]

MITSNDFKNGMTIEVDGEVYSIVEFQHVKPGKGAAFVRTKLRHMKSGNVS

EKTFRAGEKVKRAHLEEREMQFLYAAGDMYNFMDTESFEQYTLTKDQLED

KTQFIKENMIITVLFHNGEEISIELPVFVELAVSETEPGVKGDTASGGSK

PATLETGATVNVPFFINEGDIIKVDTRTSEYIERVKGE

>fig|95667.5.peg.5 [Ureaplasma urealyticum serovar 13] [Translation elongation factor P @ Translation initiation factor 5A]

MATIIQAKDLRAGHTFLYKGSIYQVIENSFNKTAMREGIVKCKVKNLRTG

AITVEVLTGEKVEQAIIEKSKMTFSYDDGSGYVFMDNETYEQISIPYNQL

SWEKNFIEEGTEVSVMRYDGELMGVSLPDQLVVTIVEAEEAVQGNSVQNA

TKRAWLASKWEFQVPQFIKSGEKVIINPSNGQYVGRAK

>fig|313594.3.peg.2179 [Polaribacter irgensii 23-P] [Translation elongation factor P @ Translation initiation factor 5A]

MATTSDIRNGLCIRYNNDIYKVIEFLHVKPGKGPAFVRTKLKSVTNGKVV

DNTFPAGRKIEDIRVETHKFQYLYNEGETFHFMNEKDYSQIQLQKNVLDA

PELMKEGEVVTIIINAEDEMPLSVDMPASVVLEVTHTEPGVKGNTATNAT

KPATVESGATVNVPLFINEGDKIKVETTKGTYQERIKE

>fig|134537.1.peg.6487 [Burkholderia fungorum] [Translation elongation factor P @ Translation initiation factor 5A]

MKTAQELRTGNVVMIGADAMVVQKAEYNKSGRNSAVVKMKFKNLLTGAGM

ESVYKADDKFDVVVLERKEVTYSYFADPMYVFMDADYNQFEVESEMMGDA

LHYLEDGMACEVVFYNDKAISVELPTTLVREIIYTEPAVKGDTSSGKVLK

NAKLNTGFELQVPLFCNIGDKIEIDTRTHEYRSRA

>fig|218497.4.peg.795 [Chlamydophila abortus S26/3] [Translation elongation factor P @ Translation initiation factor 5A]

MVRVSTSEFRVGLRIEIDGQPYLILQNDFVKPGKGQAFNRIKVKNFLTGR

VIEKTFKSGESVETADVREQQMRFLYSDQEGATFMDDKTFEQEVIFWDKI

ENIRQWLLEDTIYTLVLYNGNVIAVEPPIFMELTIAETAPGVRGDTASGR

VLKPAVTNTAAKIMVPIFIEEGEVVKIDTRTGSYESRVSK

>fig|296591.1.peg.2050 [Polaromonas sp. JS666] [Translation elongation factor P @ Translation initiation factor 5A]

MANNHTGSPVPPAGAAGPEYPPARICGKKPLKSKALRISLLGEPHNPPQN

RTQVQAFGGLPSSRTAMKIAQEIRAGNVIMHGKDPMVVLKTEYSRGGRNS

ATVRMKLKSLIANFNTEVVFKADDKMDQVILDKKECTYSYFADPMYICMD

SEYNQYEVEAENMGDSLNYLQDGMELEVVFYDGKAISVEVPTSVQREITW

TEPAVKGDTSGKVLKPAKIATGFEIGVPIFVAQGDVVEIDTRTGEYRKRV

>fig|331109.3.peg.4610 [Burkholderia pseudomallei 1655] [Translation elongation factor P @ Translation initiation factor 5A]

MKTAQELRVGNVVMIGNDAWVVSKTEYNKSGRNAAVVKMKLKNLLNGGGQ

ESVYKADDKFEVVVLDRKEVTYSYFADPMYVFMDADYNQYEVEAEMMGDA

LNYLEDGMACEVVFYNEKAISVELPTILVREITYTEPAVKGDTSSGKVLK

NAKLATGFELQVPLFCNTGDKIEIDTRTNEYRSRA

>fig|85962.1.peg.174 [Helicobacter pylori 26695] [Translation elongation factor P @ Translation initiation factor 5A]

MAIGMSELKKGLKIELGGVPYRIVEYQHVKPGKGAAFVRAKIKSFLDGKV

IEKTFHAGDKCEEPNLVEKTMQYLYHDGDTYQFMDIESYEQIALNDSQVG

EASKWMLDGMQVQVLLHNDKAISVDVPQVVALKIVETAPNFKGDTSSASK

KPATLETGAVVQVPFHVLEGEIIKVNTETEEYLEKVK

>fig|243274.1.peg.1744 [Thermotoga maritima MSB8] [Translation elongation factor P @ Translation initiation factor 5A]

MIEVGDLKKGMFIIYDGEIYRVLEASKHFMGRGSGLIRTKLKNVKTGFVR

EVNFPSGEKVQEAELSFRKAQYLYRDGDHYYFMTLDDYEQYALSEEEIGD

AKYYLVENMEVDLVFHEGTPIGIELPTTVELTVVETEPSFKGDTVSGGGK

PAVLETGLKITVPYFIEVGDKIKVDTRTGEYVGRA

>fig|260799.1.peg.4060 [Bacillus anthracis str. Sterne] [Translation elongation factor P @ Translation initiation factor 5A]

MISVNDFRTGLTIAVDNGLWQVLDFQHVKPGKGAAFVRSKLRNLRTGSVQ

EKTFRAGEKVEKAHIENRRMQYLYASGEAHVFMDNGTYEQIELGEKQIER

ELKFLKENMEVSIMTYQGEVLGVELPNTVELQVTETEPGIKGDTASNVTK

PATLETGLVVQVPIFINEGEMLIINTGEGKYVSRA

>fig|272634.1.peg.29 [Mycoplasma pneumoniae M129] [Translation elongation factor P @ Translation initiation factor 5A]

MADMIEAKSLRSGQTIFGPNKEILLVLENTFNKTAMRQGIVKTKVKNLRT

GAIVWIEFTGDKLEQVIIDKKKMTFLYKDGANYVFMDQQDYSQIEIPEKQ

LEWEKNFITEDSEVTIISYQSEILGVNLPELVPIEVEFAEEAVQGNTANM

ARKRARLVSGYELDVPQFIRTGDKIVISTIDGSYRERYNK

>fig|235279.1.peg.108 [Helicobacter hepaticus ATCC 51449] [Translation elongation factor P @ Translation initiation factor 5A]

MAIGMSELKKGLKIEIDGIPYRITEYQHVKPGKGAAFVRAKIKSFLDGKV

IEKTFHAGDKCEEPNLQEKTMQFLYHDGGAFQFMDTTTYEQIALSDDQVG

DVAKWIIDGLNVQILFHNEKAISVDVPLVVELTITETAPNFKGDTSSGGK

KPATLETGAVVQVPFHVLEGEKIKVNTETGEYLEKVK

>fig|247156.1.peg.3545 [Nocardia farcinica IFM 10152] [Translation elongation factor P @ Translation initiation factor 5A]

MADTSDFKNGLVLKIDGQLQQIVEFQHVKPGKGPAFVRTKLKNVVSGKIV

DKTFNAGVKVETATVDRRDMTYLYHDGSDYVFMDGETFDQISISEATIGS

SARFLLENMAVQVAMHEGAPLYVELPVSVELEVTHTDIGLQGDRSTGGTK

PATLETGAEVQVPLFINTGDKLRIDSRDGSYLGRVNA

>fig|321967.8.peg.1610 [Lactobacillus casei ATCC 334] [Translation elongation factor P @ Translation initiation factor 5A]

MRSTLKNLRTGAVQEKTFRSTEKVEKAQIDSKNMQYLYADGDNYVFMDTD

TYDQLTLPGDEIRDQLNYLKENMNVKIIMHGNETLGIELPKTVDLVVKET

EPGIRGNTSSGGSKPATMETGLVVQVPFFINVDDVLTINTDDGTYISRAN

N

>fig|1140.3.peg.2647 [Synechococcus elongatus PCC 7942] [Translation elongation factor P @ Translation initiation factor 5A]

MISSNDFRTGTTIEIDGAVWRVVEFLHVKPGKGSAFVRTKLKNAKTGNVV

EKTFRAGETVPQAVLEKSTLQYTYKDGDDFVFMDMETYEEGRLTAATIGD

RVKYLKEGMEANVITWNGQVIEVELPNSVVLEVIETDPGVKGDTATGGTK

PAKVETGAQVMVPLFISVGERIKIDTRNDSYLGRE

>fig|205913.1.peg.1127 [Bifidobacterium longum DJO10A] [Translation elongation factor P @ Translation initiation factor 5A]

MAQTTNDIKNGSVLNLDGQLWTVMKFQHVKPGKGPAFVRTTIKNVLSGKI

VDKTFNAGMKMEFETVDNRTLQYSYEDGDNFVFMDMTTYDQIMVPKTLLG

DKAKFLLEGTDCLVSFHDGTPLSVDLPGSVVLTITHTEPGLQGNRSNAGT

KPATVETGAEIQVPLFINEGDRVKINTEDGSYTGRENN

>fig|525903.4.peg.613 [Thermanaerovibrio acidaminovorans DSM 6589] [Translation elongation factor P @ Translation initiation factor 5A]

MAQVVDTSDLRPGMKIKWEGGMWTILECSHHKMGRGGAIVRGKLRNLETG

AAIEQSFKSGERFERIVFDEKPAQYQYQEGDNYVFMDLESYDQVYIHKDI

LGDVTKFLVDNLEVQLEMYEGRIMGIELPNSVVMKVVDTPPGFKGDTASG

GGKPATTETGLVVTVPFFVENGEEIVVDTRSGEYLERAKK

>fig|334803.3.peg.1259 [Burkholderia mallei JHU] [Translation elongation factor P @ Translation initiation factor 5A]

MKTAQELRVGNVVMIGNDAWVVSKTEYNKSGRNAAVVKMKLKNLLNGGGQ

ESVYKADDKFEVVVLDRKEVTYSYFADPMYVFMDADYNQYEVEAEMMGDA

LNYLEDGMACEVVFYNEKAISVELPTILVREITYTEPAVKGDTSSGKVLK

NAKLATGFELQVPLFCNTGDKIEIDTRTNEYRSRA

>fig|1314.1.peg.225 [Streptococcus pyogenes M5] [Translation elongation factor P @ Translation initiation factor 5A]

MIEASKLKAGMTFEAEGKLIRVLEASHHKPGKGNTIMRMKLRDVRTGSTF

DTTYRPDEKFEQAIIETVPAQYLYKMDDTAYFMNTDTYDQYEIPVANVEQ

ELLYILENSDVKIQFYGSEVIGVTVPTTVELTVAETQPSIKGATVTGSGK

PATLETGLVVNVPDFIEAGQKLIINTAEGTYVSRA

>fig|295405.3.peg.262 [Bacteroides fragilis YCH46] [Translation elongation factor P @ Translation initiation factor 5A]

MINAQDIKNGTCIRMDGKLYFCIEFLHVKPGKGNTFMRTKLKDVVSGYVL

ERRFNIGEKLEDVRVERRPYQYLYKEGEDYIFMNQETFDQHPIAHDLING

VDFLLEGAVVEVVSDASTETVLYADMPIKVQMKVTYTEPGLKGDTATNTL

KPATVESGATVRVPLFISEGETIEIDTRDGSYVGRVKA

>fig|351746.4.peg.3803 [Pseudomonas putida F1] [Translation elongation factor P @ Translation initiation factor 5A]

MKTGKELKPGTVLRIDNDPWLVQKAEFTKSGRNSAIMKTKLKNLLTGYKT

ETVYGADDKLDDVILDRKEATLSFINGDEYTFMDTTDYTMYELNAEDIEA

VLPYIEEGMEDVCEAVFFEGRLVSVELPTTISRKVVYTENAARGDTSGKV

MKPAKLANGTEISVADFIQIDEWIDIDTRDNSFKGRSKK

>fig|186103.1.peg.1555 [Streptococcus pyogenes MGAS8232] [Translation elongation factor P @ Translation initiation factor 5A]

MIEASKLKAGMTFEAEGKLIRVLEASHHKPGKGNTIMRMKLRDVRTGSTF

DTTYRPDEKFEQAIIETVPAQYLYKMDDTAYFMNTDTYDQYEIPVANVEQ

ELLYILENSDVKIQFYGSEVIGVTVPTTVELTVAETQPSIKGATVTGSGK

PATLETGLVVNVPDFIEAGQKLIINTAEGTYVSRA

>fig|264203.3.peg.824 [Zymomonas mobilis subsp. mobilis ZM4] [Translation elongation factor P @ Translation initiation factor 5A]

MKISGVDIRPGNILEYEGGLWRAAKIQHTQPGKGGAYMQVEMKNLIDGRK

TNVRFRSAETVERVRLDTKDFQYLFADGDMLTFMDKETYEQISLPKDLLG

DAVAFLQDGMDVVMELYEEKPISVQLPEQVEAEIVEADAVVKGQTASSSY

KPAILDNGVRVMVPPHITAGTRIIVDVNTQEYVKRAD

>fig|95661.4.peg.379 [Ureaplasma urealyticum serovar 7] [Translation elongation factor P @ Translation initiation factor 5A]

MATIIQAKDLRAGHTFLYKGSIYQVIENSFNKTAMREGIVKCKVKNLRTG

AITVEVLTGEKVEQAIIEKSKMTFSYDDGSGYVFMDNETYEQISIPYNQL

SWEKNFIEEGTEVSVMRYDGELMGVSLPDQLVVTIVEAEEAVQGNSVQNA

TKRAWLASKWEFQVPQFIKSGEKVIINPSNGQYVGRAK

>fig|390874.10.peg.1056 [Thermotoga petrophila RKU-1] [Translation elongation factor P @ Translation initiation factor 5A]

MIEVGDLKKGMFIIYDGEIYRVLEASKHFMGRGSGLIRTKLKNVKTGLVR

EVNFPSGDKVPEAELSFRKAQYLYRDGDHYYFMTLDDYEQYALSEEEIGD

AKYYLVENMEVDLVFHEGTPIGIELPTTVELTVVETEPSFKGDTVSGGGK

PAVLETGLKITVPYFIEVGDKIKVDTRTGEYVGRA

>fig|246194.3.peg.2438 [Carboxydothermus hydrogenoformans Z-2901] [Translation elongation factor P @ Translation initiation factor 5A]

MISTNDFRTGLTIELDGEVYQVIEFQHVKPGKGSPFVRSKLRNLMTGAVI

EKTFNAGEKVPKAHVDRREVQYLYNDGDNFYCMDMETYDQFPLTKEQFGD

AINYVKENTNLWVLFFKDKVIGVELPNFVELKVIDTPPGIKGDTASGGSK

PATLETGYVVQVPFFVEVGDVLQIDTRTGQYIKRV

>fig|312284.3.peg.2333 [marine actinobacterium PHSC20C1] [Translation elongation factor P @ Translation initiation factor 5A]

MASTADIRNGVVLNMDGGLWTVIEFQHVKPGKGGAFVRTKVKNVRTGKTV

DRTFNAGAKIETETVDRSEFQYLYKDLENYVFMNTSDYDQITLSAAAVGD

ASNFMLENQNVVIALHNGEPLYVELPASVVLEITYTEPGLQGDRSTGGSK

PATVETGYQIQVPLFLEQGTKVKVDTRDGSYLGRVND

>fig|266779.1.peg.101 [Mesorhizobium sp. BNC1] [Translation elongation factor P @ Translation initiation factor 5A]

MKINGNEIRPGNVIEHNGGLWVAVKTNAVKPGKGGAYNQVELKNLIDGTK

LNERFRSAETVEKVRLEQKDFTFLYEQGDALVFMDSETYEQLELQKDFVG

DRAAFLQDGMTVTVELYQEKPIGISLPPQVTLQVAEADPVVKGQTAASSY

KPAVLENGVRILVPPFVSAGERIVVDTDEITYLRRAD

>fig|357808.3.peg.3297 [Roseiflexus sp. RS-1] [Translation elongation factor P @ Translation initiation factor 5A]

MATTSDLRTNMIIRYNGQLHRVVEFYHHAPGNWRAMVIMKLKNLETGKTI

EERVRAGSEIEIVRVEKRPMQFLYRDGDVYHFMDTETFEQIEVPEEMIGE

PAKFLKENEMADILFYDDNKILGVEPPLFVTLQVTEASVAVRGDTATNVN

KQVTLETGAVISVPAFVNQGDYVRVDTRTGEYIERIK

>fig|402882.10.peg.2289 [Shewanella baltica OS185] [Translation elongation factor P @ Translation initiation factor 5A]

MKTAHEIRPGNVIMLDGSPWVVQKTETTRSGRNAAIVKLKLKNLLLNSGT

ETTFKGEDKLEDIILDRLDCTYSYFADPMFVFMDAEYNQYDVEAENLGDA

AAYIVDGMEETCQVTFYDGKAISVEMPTTIVREVIYTEPSARGDTSGKVM

KPATITGGGTVTVADFVKVGDKIEIDTRTGEFKKRV

>fig|411154.5.peg.1676 [Gramella forsetii KT0803] [Translation elongation factor P @ Translation initiation factor 5A]

MASTSDIRNGLCIRYNHDIYKITEFLHVKPGKGPAFVRTKLKSVTTGKVL

DNTFSAGHKIEEIRVETHKFQFLYEDGEFWHFMNVEDYTQIRLTENALDM

PKLIKEGEVVTILINTEDNMPLSVDMPASVVLEVTHTEPGVKGNTATNAT

KPATVETGFEVNVPLFINEGDKIKIETDKGTYKERIKE

>fig|266264.4.peg.2808 [Cupriavidus metallidurans CH34] [Translation elongation factor P @ Translation initiation factor 5A]

MKIAQELRVGNVFMIGSDPMVVQKAEYNKSGRNAAVVKMKYKNLLTEAPG

ESVFKADDKFEVVVLERRECTYSYFADPMYVFMDTEYNQYEVEKDSMGDS

LNYLEDGMVVEVVFYNDKAISVEMPTTLVREIIYTEPAVKGDTSSGKVLK

GAKINTGFELQVPLFCNIGDKIEIDTRTGEYRSRAN

>fig|324831.10.peg.693 [Lactobacillus gasseri ATCC 33323] [Translation elongation factor P @ Translation initiation factor 5A]

MTMISVNEFKNGLTIEYNNDLWRIVEFQHVKPGKGSAFVRSKLKSLRTGA

VQEYTFRSTAKVNTADIQTKAMQYLYNDGTSFVFMDTTTYEQLEIPEAQV

ERESKFLKENMVVNVIMHDGETLGVDLPNTVDLEVAETEPNIKGDTSSGG

GKPATMETGLVVNVPFFINQGDVLTINTADGTYVSRANK

>fig|176280.1.peg.1213 [Staphylococcus epidermidis ATCC 12228] [Translation elongation factor P @ Translation initiation factor 5A]

MISVNDFKTGLTISVDNGIWKVIDFQHVKPGKGSAFVRSKLRNLRTGAIQ

EKTFRAGEKVEQAMIENRRMQYLYADGDNHVFMDNETFNQIELPGDYLKD

ELNYLKANMEVQVQSYESEVIGVELPKTVELEVTETEPGIKGDTATGATK

SATVETGYTLNVPLFVNEGDTLVINTSDGSYISRG

>fig|479435.4.peg.6776 [Kribbella flavida DSM 17836] [Translation elongation factor P @ Translation initiation factor 5A]

MATTTNDLKNGMVLNLDGQLWSVVWFQHHKPGKGGAVVRTKLKNVLSGKV

VDKTFNADVKVEVASVDKRDMTYLYNDGAAFVFMDKSTYDQLQIQPDVVG

DVSNFLLENQDAIVAVHNDIPLYVELPASVELTVEYTEPGLQGDRSTGGT

KPAKLETGYTIQVPLFLTTGEKVKVDTRTGDYLGRVTS

>fig|453362.3.peg.90 [Streptococcus pneumoniae CDC1873-00] [Translation elongation factor P @ Translation initiation factor 5A]

MIEASKLKAGMTFETADGKLIRVLEASHHKPGKGNTIMRMKLRDVRTGST

FDTSYRPEEKFEQAIIETVPAQYLYKMDDTAYFMNTETYDQYEIPVVNVE

NELLYILENSDVKIQFYGTEVIGVTVPTTVELTVAETQPSIKGATVTGSG

KPATMETGLVVNVPDFIEAGQKLVINTAEGTYVSRA

>fig|176279.3.peg.1204 [Staphylococcus epidermidis RP62A] [Translation elongation factor P @ Translation initiation factor 5A]

MISVNDFKTGLTISVDNGIWKVIDFQHVKPGKGSAFVRSKLRNLRTGAIQ

EKTFRAGEKVEQAMIENRRMQYLYADGDNHVFMDNETFDQIELPGDYLKD

ELNYLKANMEVQVQSYESEVIGVELPKTVELEVTETEPGIKGDTATGATK

SATVETGYTLNVPLFVNEGDTLVINTSDGSYISRG

>fig|160490.1.peg.1401 [Streptococcus pyogenes M1 GAS] [Translation elongation factor P @ Translation initiation factor 5A]

MIEASKLKAGMTFEAEGKLIRVLEASHHKPGKGNTIMRMKLRDVRTGSTF

DTTYRPDEKFEQAIIETVPAQYLYKMDDTAYFMNTDTYDQYEIPVANVEQ

ELLYILENSDVKIQFYGSEVIGVTVPTTVELTVAETQPSIKGATVTGSGK

PATLETGLVVNVPDFIEAGQKLIINTAEGTYVSRA

>fig|320371.3.peg.3744 [Burkholderia pseudomallei 1710a] [Translation elongation factor P @ Translation initiation factor 5A]

MKTAQELRVGNVVMIGNDAWVVSKTEYNKSGRNAAVVKMKLKNLLNGGGQ

ESVYKADDKFEVVVLDRKEVTYSYFADPMYVFMDADYNQYEVEAEMMGDA

LNYLEDGMACEVVFYNEKAISVELPTILVREITYTEPAVKGDTSSGKVLK

NAKLATGFELQVPLFCNTGDKIEIDTRTNEYRSRA

>fig|370553.3.peg.1572 [Streptococcus pyogenes MGAS2096] [Translation elongation factor P @ Translation initiation factor 5A]

MIEASKLKAGMTFEAEGKLIRVLEASHHKPGKGNTIMRMKLRDVRTGSTF

DTTYRPDEKFEQAIIETVPAQYLYKMDDTAYFMNTDTYDQYEIPVANVEQ

ELLYILENSDVKIQFYGSEVIGVTVPTTVELTVAETQPSIKGATVTGSGK

PATLETGLVVNVPDFIEAGQKLIINTAEGTYVSRA

>fig|313603.3.peg.3093 [Flavobacteriales bacterium HTCC2170] [Translation elongation factor P @ Translation initiation factor 5A]

MASTSDIRKGLCIKYNHDIFKIIEFLHVKPGKGPAFVRTKLKSVTTGKVI

DNTFSAGHKIEDVRVETRSYQFLYAEGDTYHFMNTSDYNQISLEKSSLDA

PGLLKEGEVVTIMFNTEDSMPLSVDMPASVILEVTHTEPGVKGNTATNAT

KPATVETGAKVNVPLFINEGDKIKVETEKGSYMERVKD

>fig|392499.4.peg.3134 [Sphingomonas wittichii RW1] [Translation elongation factor P @ Translation initiation factor 5A]

MKISGVDIRPGNIIEYEGGIWRAVKIQHTQPGKGGAYMQVEMKNLIDGRK

NNVRFRSAETVERVRLDTKDFQFLFAEGEDLTFMDKDTYEQITLPRDLLG

DAAAFLQDGMDVVMELYEERPISVQLPDQVEATIVEADAVVKGQTASSSY

KPAMLDNGVRVMVPPHISSGTRIVVDVYEQTYVRRAD

>fig|232721.5.peg.2907 [Acidovorax sp. JS42] [Translation elongation factor P @ Translation initiation factor 5A]

MKIAQEIRAGNVIMQGKDPMIVLKTEYARGGRGAATVRMKLKALLSNMGT

EVVFKADDKIDNVILDKKECTYSYFADPMYVWMDAEYNQYEVEASNMGDA

ISYLEDGMAAEVVFYDGKAISVELPTSVEREITWTEPAVKGDTSGKVLKP

AKIATGFEVAVPLFVDQGDKIEIDTRTGEYRKRV

>fig|292805.3.peg.59 [Wolbachia endosymbiont strain TRS of Brugia malayi] [Translation elongation factor P @ Translation initiation factor 5A]

MAERANDIRPGQVLEHNGGLFLVVSIMHTQPGKGGAYIQAEMKNIKTGAK

LYERFRSDATIRRAILDEEEYIYLFTEGNIVNLMHPSNYEQITINLDLLE

EKKIYLQDNMRIKVVTYQDKIIFAHVPDYVRLTVKETESFIKGQTITSSY

KPAVLENGMRINVPQFIKEEDKIVVYTPDDSYYERVKE

>fig|1496.1.peg.140 [Clostridium difficile 630] [Translation elongation factor P @ Translation initiation factor 5A]

MEEQKMVSAGDFRKGVTFEKDGQPCLVVDFQHVKPGKGAAFVRTKYKNLK

TGAIREESFNPSEKFPKAVIDTRQMQYLYNDGELYYFMDQENFEQIPLNY

EQVEDAIKFLKENEVATIRFYQGQPFQVEAPNFAELEVTDTEPGIKGDTA

SNVTKAATVETGAVVQVPLFINTGDKVKIDTRTGEYLSRV

>fig|375451.6.peg.1633 [Roseobacter denitrificans OCh 114] [Translation elongation factor P @ Translation initiation factor 5A]

MPKINGNEIRPGNVLEHNDGLWAAVKVDHVKPGKGGAFAQVEMRNLRNGS

KLNERFRSADKVERVRLEQKDQQFLYEDAGMLVVMDTETYEQVQLPAELL

GERRPFLQDGMTIVVEFYEEEALNATLPQKVVCKIVETEAVVKGQTAAKS

FKPALLENGVKVMVPPFVGQDENIVVNTETMEYSERA

>fig|60480.16.peg.1938 [Shewanella sp. MR-4] [Translation elongation factor P @ Translation initiation factor 5A]

MKTAHEVRPGNVIMFEGSPWVVQKTETTRSGRNAAIVKLKLKNLLLNSGT

ETTFKGEDKIDDIILDRLDCTYSYFADPMYVFMDAEYNQYDVEAENLGDA

AAYIVDGMEETCQVTFYEGKAISVEMPTTIVREVIYTEPSARGDTSGKVM

KPATITGGGTISVADFVKVGDKIEIDTRTGEFKKRV

>fig|298653.4.peg.1671 [Frankia sp. EAN1pec] [Translation elongation factor P @ Translation initiation factor 5A]

MATTNDLKNGMTLDIDGVLWNVVGFQHVKPGKGGAFVRTTLKNVLTGKVV

DRTFNAGIKVDVATVDRREMTYLYRDGADFVFMDSESYDQIPVPPGVVGG

VADYMLENTIATVALHDDAPLYVELPASVELTISATDPGVQGDRSTGGTK

PATLETGANIQVPLFITTGEKVKVDTRDGRYLGRVTS

>fig|95663.4.peg.660 [Ureaplasma urealyticum serovar 9] [Translation elongation factor P @ Translation initiation factor 5A]

MATIIQAKDLRAGHTFLYKGSIYQVIENSFNKTAMREGIVKCKVKNLRTG

AITVEVLTGEKVEQAIIEKSKMTFSYDDGSGYVFMDNETYEQISIPYNQL

SWEKNFIEEGTEVSVMRYDGELMGVSLPDQLVVTIVEAEEAVQGNSVQNA

TKRAWLASKWEFQVPQFIKSGEKVIINPSNGQYVGRAK

>fig|485916.4.peg.2219 [Desulfotomaculum acetoxidans DSM 771] [Translation elongation factor P @ Translation initiation factor 5A]

MISTNDFRTGLTIEIDNDIYQVVDFQHVKPGKGAAFVRSKLRNMRTGAVV

DKTFNAGEKLHKGRVERKEVQYLYNDGTSYNFMDMETFDQMSMNREELGD

AVKYLKENMLITIQTFQEKPIGFDLPNFVELEVTDTAPGIKGDTASGGSK

PATLETGYVVQVPFFINVGDVLQIDTRTGNYIKRA

>fig|522772.4.peg.706 [Denitrovibrio acetiphilus DSM 12809] [Translation elongation factor P @ Translation initiation factor 5A]

MITPNQFKRGAKIEIDGEPYGIVEYLHIKCGRGGATVRTKMKSLRTGAVI

ERTFKSDEKIKEPDFEDKEMQYLYNDGTVFFFMDTESYEQIEIPAEFVGD

DSDFMPDNIMVNVQVFNGQPIGITLPNFVELEITYTEPGLKGDTVTGGNK

PATVNTGGTVNVPLFINIGDVIKIDTREQTYMERVSSAK

>fig|159087.4.peg.2046 [Dechloromonas aromatica RCB] [Translation elongation factor P @ Translation initiation factor 5A]

MKTAMELRSGNVIMVGADPLVVQKSEYNKSGRNAAVVKMKLKNLLTGAAS

EAVYKADDKFEVVVLDKKEVTYSYFADPMYVFMDADYEQFEVEAENMTDA

LKYLEDGLACEVVFYNGKAISVELPNSVVREVVYTEPAVKGDTSGKVMKP

AKLATGFELPVPAFVSIGDKIEIDTRTDEYKNRVK

>fig|471853.4.peg.2425 [Beutenbergia caverna cavernae DSM 12333] [Translation elongation factor P @ Translation initiation factor 5A]

MASTNDLKNGLVLDLDGQLWTVVEFQHVKPGKGPAFVRTKLKHVLSGKTV

DKTFNAGIKVDTATVDKRDMQYLYKDGDDFVFMDSSDYEQFHVGPQIVGD

AANFMLENTEVIVALHEGNPLYVELPTSVVLEITYTEPGLQGDRSSAGTK

AATVETGYQIQVPLFLEIGTKVKVDTRSGDYLGRVN

>fig|306537.3.peg.723 [Corynebacterium jeikeium K411] [Translation elongation factor P @ Translation initiation factor 5A]

MATTADFKNGLVLKVDGKLQQIVEFQHVKPGKGPAFVRTKLKDVVSGKTI

DKTWNAGVKVETATVDRRDMTYLYNDGTSFVLMDEKTYDQIELAPHLMGD

GAKFLLENTSVQVSFHEGEPLFAELPVSVELKIEHTDPGLQGDRSTGGSK

PATLETGAEIQVPLFLETGNVVKVDTRTGEYLSRVNN

>fig|399726.4.peg.1486 [Thermoanaerobacter sp. X514] [Translation elongation factor P @ Translation initiation factor 5A]

MIAAGDFRKGVTIEVDGQVFTVVDFMHVKPGKGAAFVRTKLKNVMTGAVI

EKTFSPTEKFEEAVIERREMQYLYNDGELYYFMDTETYEQIPLNYDKVED

AIKYIKENMVVTVKFYKGEAFSVEPPTFVELEVVETEPGFRGDTATGGSK

PATVETGAVIQVPLFINVGDKIRIDTRTGEYLERV

>fig|196627.4.peg.2166 [Corynebacterium glutamicum ATCC 13032] [Translation elongation factor P @ Translation initiation factor 5A]

MATTADFKNGLVLKNEGKLQQIIEFQHVKPGKGPAFVRTKLKDVVTGKTI

DKTWNAGVKVETATVDRRDVTYLYNDGTSFIVMDDKTFEQYELSPDAFGD

AGRFLLENMRVQVSFHEGEALFGELPVSVDLRVEHTDPGLQGDRSTGGTK

PATLETGAEIQVPLFIETGNVLKVDTRDGSYLSRVNN

>fig|295358.3.peg.527 [Mycoplasma hyopneumoniae 232] [Translation elongation factor P @ Translation initiation factor 5A]

MINVNEFRPGITFEFENEIYVVISAQHSKQGRGQANVKAKVKNLRTGAIT

IKTFSGGERVEKAHIEKISMSFLYNDGVSIVLMDDSTYEQVAIENTKITW

ELNFLTEGIKVKLRKFNNEILDIELPAKIELKITSTFDAVRGNTTTNPTK

RATLETGYEIDVPLFIKEGESVIVSTEDGKYVSRA

>fig|272562.1.peg.2242 [Clostridium acetobutylicum ATCC 824] [Translation elongation factor P @ Translation initiation factor 5A]

MISAGDLRKGTTFELDGQVYNVVDFLHVKPGKGAAFVRTKLKNVITGAVT

ETTFNPTAKMQEAVIERKEMQYLYSDESFYYFMDQETYEQIPLSFDQVEN

AIKYLKENMFATIKFYKGEAFSVEAPNFVELKIVSCEPGVKGNTTSNVMK

PATLETNAVVQVPLFVNEGETIRVDTRTGEYMERVQ

>fig|281309.3.peg.4177 [Bacillus thuringiensis serovar konkukian str. 97-27] [Translation elongation factor P @ Translation initiation factor 5A]

MISVNDFRTGLTIAVDNGLWQVLDFQHVKPGKGAAFVRSKLRNLRTGSVQ

EKTFRAGEKVEKAHIENRRMQYLYASGEAHVFMDNGTYEQIELGEKQIER

ELKFLKENMEVSIMTYQGEVLGVELPNTVELQVTETEPGIKGDTASNVTK

PATLETGLVVQVPIFINEGEMLIINTGEGKYVSRA

>fig|203124.1.peg.4852 [Trichodesmium erythraeum IMS101] [Translation elongation factor P @ Translation initiation factor 5A]

MISSNDFRPGVTIELDGSVWRVVEFLHVKPGKGSAFVRTKLKNVQNGNVV

ERTFRAGETVPQATLDKRAMQHTYKDSDQFVFMDMETYEETNLSSEQIGE

RVKYLNEGMEVNVIMWGEQVIEVELPNSVVLEVIETDPGVKGDTATGGSK

PATVETGAQIMVPLFISKGERIKIDTRNDSYLGRE

>fig|211586.1.peg.2108 [Shewanella oneidensis MR-1] [Translation elongation factor P @ Translation initiation factor 5A]

MKTAHEVRPGNVIMFEGSPWVVQKTETTRSGRNAAIVKLKLKNLLLNSGT

ETTFKGEDKIDDIILDRLDCTYSYFADPMYVFMDAEYNQYDVEAENLGDA

AAYIVDGMEETCQVTFYDGKAISVEMPTTIVREVIYTEPSARGDTSGKVM

KPATITGGGTISVADFVKVGDKIEIDTRTGEFKKRV

>fig|160488.1.peg.1843 [Pseudomonas putida KT2440] [Translation elongation factor P @ Translation initiation factor 5A]

MKTGKELKPGTVLRIDNDPWLVQKAEFTKSGRNSAIMKTKLKNLLTGYKT

ETVYGADDKLDDVILDRKEATLSFINGDEYTFMDTTDYTMYELNAEDIEA

VLPYIEEGMEDVCEAVFFEGRLVSVELPTTISRKVVYTENAARGDTSGKV

MKPAKLANGTEISVADFIQIDEWIDIDTRDNSFKGRSKK

>fig|36873.1.peg.2903 [Burkholderia xenovorans LB400] [Translation elongation factor P @ Translation initiation factor 5A]

MKTAQELRTGNVVMIGADAMVVQKAEYNKSGRNSAVVKMKFKNLLTGAGM

ESVYKADDKFDVVVLERKEVTYSYFADPMYVFMDADYNQFEVESEMMGDA

LHYLEDGMACEVVFYNDKAISVELPTTLVREIIYTEPAVKGDTSSGKVLK

NAKLNTGFELQVPLFCNIGDKIEIDTRTHEYRSRA

>fig|234267.9.peg.3048 [Solibacter usitatus Ellin6076] [Translation elongation factor P @ Translation initiation factor 5A]

MAVLIDAIDVKKRTLFELDNVPFSCLEAEVNTPTARGGQTLVRLKVRNLL

TNAVFEKTFKAGEKFKEPDLQLVSASYLYSDGSGSHFLDQESFETHTLSG

EIMGNALDFLIDGTLVELLKFNGNPIGLNLPASVELTVTFTEPGVRGDSS

SGSVTKPAKLETGLEIRVPLFIKEGEKVKVSTENRSFSGRA

>fig|234267.9.peg.3494 [Solibacter usitatus Ellin6076] [Translation elongation factor P @ Translation initiation factor 5A]

MISATQLRPGMVIKYNNELYTVFSVNHRTPGNLRGFVQARMRSLRTGSMT

ENRFSSEDKVEKAIMEQHEMEYLYDDGEYYYFMNIETYEQMHLMKDLLGD

ATNYLIPNLKLSVEFYEGKPISVELPPTVDMTVVETEPGLKGATVSNVTK

PAKMETGLVVQVPPFITEGEKIRVNTTEGTYQERA

>fig|272633.1.peg.159 [Mycoplasma penetrans HF-2] [Translation elongation factor P @ Translation initiation factor 5A]

MSQIIHAKDLRPGNTFIYKNNLYLVIENSFNKTAMREGIVKCKVKNLRTS

SITVEVLTGEKLERAIIDKVKAMYSYADKNTLVFMDGESFEQIEIDANKL

KWEKNFIVDGSEVSILKYGEEVLNVSLPDQVSLLVDFAEEAVQGNTVQTA

MKKARLETGLEIEVPQFIKTGEKIIVNTVDGKYVGRDK

>fig|266117.6.peg.1342 [Rubrobacter xylanophilus DSM 9941] [Translation elongation factor P @ Translation initiation factor 5A]

MISTNQFRNGSAIRVDGKRFTILSFQHVKPGKGHAFVRTRLRNMDTGAVI

ERTFRAGEKVESIRTDSRPMSFLYRDGDLYYFMDQETYEQVAIPEDVVGE

AKDFITPNGEVSVLYADGEVVSVQPPAHVELEVVETDPGVRGDTATGGSK

PATLETGLVVQVPLFVSVGDRVRVDTRTREYQTRV

>fig|410289.13.peg.2534 [Mycobacterium bovis BCG str. Pasteur 1173P2] [Translation elongation factor P @ Translation initiation factor 5A]

MATTADFKNGLVLVIDGQLWTITEFQHVKPGKGPAFVRTKLKNVLSGKVV

DKTFNAGVKVDTATVDRRDTTYLYRDGSDFVFMDSQDYEQHPLPEALVGD

AARFLLEGMPVQVAFHNGVPLYIELPVTVELEVTHTEPGLQGDRSSAGTK

PATLQTGAQINVPLFINTGDKLKVDSRDGSYLGRVNA

>fig|360112.3.peg.468 [Campylobacter jejuni subsp. jejuni HB93-13] [Translation elongation factor P @ Translation initiation factor 5A]

MASYSMGDLKKGLKIEIDGIPFKIVEYQHVKPGKGPAFVRIKIKSFIDGK

VLEKTFHAGDKCEAPNLEDKTMQYLYDDGENCQFMDTQTYEQVAISDDDV

GEAKKWMLDGMMVDVLFHNGKAIGVEVPQVVELKIIETAPNFKGDTQGSN

KKPATLETGAVVQIPFHVLEGEVIRVDTVRGEYIERANK

>fig|320374.3.peg.3947 [Burkholderia pseudomallei S13] [Translation elongation factor P @ Translation initiation factor 5A]

MKTAQELRVGNVVMIGNDAWVVSKTEYNKSGRNAAVVKMKLKNLLNGGGQ

ESVYKADDKFEVVVLDRKEVTYSYFADPMYVFMDADYNQYEVEAEMMGDA

LNYLEDGMACEVVFYNEKAISVELPTILVREITYTEPAVKGDTSSGKVLK

NAKLATGFELQVPLFCNTGDKIEIDTRTNEYRSRA

>fig|1313.5.peg.1574 [Streptococcus pneumoniae OXC141] [Translation elongation factor P @ Translation initiation factor 5A]

MIEASKLKAGMTFETADGKLIRVLEASHHKPGKGNTIMRMKLRDVRTGST

FDTSYRPEEKFEQAIIETVPAQYLYKMDDTAYFMNTETYDQYEIPVVNVE

NELLYILENSDVKIQFYGTEVIGVTVPTTVELTVAETQPSIKGATVTGSG

KPATMETGLVVNVPDFIEAGQKLVINTAEGTYVSRA

>fig|233150.3.peg.506 [Mycoplasma gallisepticum R] [Translation elongation factor P @ Translation initiation factor 5A]

MASIIHAKDLRSGHTFTLDGKIYLVIENSFNKTAMREGIVKCKVKNLRTG

SITTEVLTGMKLEQANIEKVKMSFVYQDQNSFVFMNNETYEQIEVPAKLL

EYEKNFITENTEALIMRYEDEILGVNLPDQVVIEIDYAEDAVQGNSVNNA

LKKATLVTGYVIEVPQFIKSKEKVIVSTVDGKYVSRAN

>fig|196162.6.peg.2563 [Nocardioides sp. JS614] [Translation elongation factor P @ Translation initiation factor 5A]

MATTNDLKNGMVLNIDGQLWAVVEFQHVKPGKGPAFVRTKLKNVESNKTV

DKTFNAGTKVETATVDRRTMQYLYNDGSSYVFMDVQSYDQLEIAPEIVGN

AKNFLLENQEAIVATNEGRVLFIELPASVELEITFTEPGLAGDSATGRTK

PATLETGHEIQVPLFINQGEKVKVDTRDSSYLGRVKG

>fig|350702.3.peg.665 [Burkholderia cenocepacia PC184] [Translation elongation factor P @ Translation initiation factor 5A]

MKTAQELRVGNVVQIGSEAWVIAKAEYNKSGRNSAVVKMKMKNLLSNAGQ

ESVYKADDKFEVVVLDRKEVTYSYFADPMYVFMDADYNQYEVEAEMMGEA

LNYLEDGMACEVVFYNEKAISVELPTVLVREITYTEPAVKGDTSSGKVLK

NAKLATGFELQVPLFCNTGDKIEIDTRTNEYRSRA

>fig|445932.3.peg.764 [Elusimicrobium minutum Pei191] [Translation elongation factor P @ Translation initiation factor 5A]

MISTTDFKEGLIFENENGEIVEIVDYQHHRKSQARAVVRVKLRKLGSGSY

VETSYRPEDKFKEVSVEKRPFMYLYSEGDMAHFMNNESYDQVAVPLDKLE

NQRKYLIENMECTGLYINDQLFDIVLPIKVVLTIKSTVPGVKGDTVSNLT

KEAELETGVTIKVPLFINEGDKVIMDTRYCTYVERA

>fig|331272.3.peg.2770 [Burkholderia cenocepacia HI2424] [Translation elongation factor P @ Translation initiation factor 5A]

MKTAQELRVGNVVQIGSEAWVIAKAEYNKSGRNSAVVKMKMKNLLSNAGQ

ESVYKADDKFEVVVLDRKEVTYSYFADPMYVFMDADYNQYEVEAEMMGEA

LNYLEDGMACEVVFYNEKAISVELPTVLVREITYTEPAVKGDTSSGKVLK

NAKLATGFELQVPLFCNTGDKIEIDTRTNEYRSRA

>fig|269482.4.peg.4452 [Burkholderia vietnamiensis strain G4] [Translation elongation factor P @ Translation initiation factor 5A]

MKTAQELRVGNVVQIGSDAWVIAKTEYNKSGRNAAVVKMKMKNLLTNAGQ

EAVYKADDKFDVVVLDRKEVTYSYFADPMYVFMDADYNQYEVEAEMMGEA

LNYLEDGMACEVVFYNEKAISVELPTILVREITYTEPAVKGDTSSGKVLK

NAKLATGFELQVPLFCNTGDKIEIDTRTNEYRSRA

>fig|392500.3.peg.2244 [Shewanella woodyi ATCC 51908] [Translation elongation factor P @ Translation initiation factor 5A]

MKTAHEIRPGNVIMLDGSPWVVQKTETTRSGRNAAIVKMKLKNVLLDSST

ETTFKGEDKMDDIILERLDCTYSYFADPMYVFMDAEYNQYDVEAGNLGDA

SAYIVDGMEEVCQVTFYEGKAISVELPTTIVREVTYTEPSARGDTSGKVM

KPATVSGGATLSVADFVKTGDMIEIDTRTGEFKKRV

>fig|526224.4.peg.1144 [Brachyspira murdochi murdochii DSM 12563] [Translation elongation factor P @ Translation initiation factor 5A]

MLPVNDLRKGDAIILDGETYLVVDAHFHRAQQRKANVKTKLKNMLKGNMI

EKTFSSTESVEEADLSYKKAQYLYEEGDSYVFMILDNYEQVHVSADILGE

SKYYLLDNSEVDLQYINDEVTAVRFPIHVVLEVTYTEPGFKGDTTGTTLK

PAKLETGIEINVPLFINIGDKIKVDTRDDSYVERVNK

>fig|412694.5.peg.3659 [Bacillus thuringiensis str. Al Hakam] [Translation elongation factor P @ Translation initiation factor 5A]

MISVNDFRTGLTIAVDNGLWQVLDFQHVKPGKGAAFVRSKLRNLRTGSVQ

EKTFRAGEKVEKAHIENRRMQYLYASGEAHVFMDNGTYEQIELGEKQIER

ELKFLKENMEVSIMTYQGEVLGVELPNTVELQVTETEPGIKGDTASNVTK

PATLETGLVVQVPIFINEGEMLIINTGEGKYVSRA

>fig|216594.1.peg.580 [Mycobacterium marinum M] [Translation elongation factor P @ Translation initiation factor 5A]

MASTADFKNGLVLQIDGQLWSIVEFQHVKPGKGPAFVRTKLKNVLSGKVV

DKTYNAGVKVETATVDRRDTTYLYRDGSDFVFMDSQDYEQHPLPESLVGD

AARFLLEGMPVQVAFHDGAPLYIELPVTVEIVVTHTEPGLQGDRSSAGTK

PATLETGAQINVPLFINTGDKLKVDSRDGGYLGRVNA

>fig|257314.1.peg.1341 [Lactobacillus johnsonii NCC 533] [Translation elongation factor P @ Translation initiation factor 5A]

MTMISVNEFKNGLTIEYNNDLWRIVEFQHVKPGKGSAFVRSKLKSLRTGA

VQEYTFRSTAKVNTADIQTKAMQYLYNDGTSFVFMDTNTYEQLEIPEAQV

ERESKFLKENMVVNVITHEGETLGVDLPNTVDLEVAETEPNIKGDTSSGG

GKPATMETGLVVNVPFFINQGDVLTINTADGTYVSRANK

>fig|267748.1.peg.551 [Mycoplasma mobile 163K] [Translation elongation factor P @ Translation initiation factor 5A]

MVNVNNFKNGITFQEEDEIFSVIEAQHSKQGRGQASVKAKVKNLRTGAIT

IKSYTGGDKVKKAHIEKIEMNFLYDEGENIVLMDNATYEQISIPKTRVEW

EMNFLVEGAKVHIRKFADEILDIEIPVNIELKVIDAPEAVKGNTSSNPQK

KVKVETGFELETPLFIKEGEIIIVSSETGKYMGKGNNK

>fig|59920.3.peg.1518 [Prochlorococcus marinus str. NATL2A] [Translation elongation factor P @ Translation initiation factor 5A]

MISSNDFRTGTTIELDGAVWRVIEFLHVKPGKGSAFVRTKLKAVVSGNVV

EKTFRAGEMVPQALLEKSKLQHTYMDGDDFVFMDMTSYEETRLTAKQIGE

SRKYLKEGMEVNVVSWNEKPLEVELPNSVVLEIKETDPGVKGDTASGGTK

PAILETGAQVMVPLFISIGEKIRVDTRNDSYLGRETQ

>fig|391774.5.peg.1447 [Desulfovibrio vulgaris subsp. vulgaris DP4] [Translation elongation factor P @ Translation initiation factor 5A]

MYSTTDFRKGLKIELDGTPFEIVDFQHFKPGKGGAMVRTKLRNILNGRVV

DNTFRSGEKVGRPDLESRDMQYLYHEGDDLVLMDLTTYEQLYMHEDLTDG

KAGFLKDGQQVRVLLYNGKPLDLELPVSLVLEVVETEPGAKGDTVSNVTK

PAKLETGIVVQVPIFVNQGDRIKVDTRSREYLGRE

>fig|269084.3.peg.1205 [Synechococcus elongatus PCC 6301] [Translation elongation factor P @ Translation initiation factor 5A]

MISSNDFRTGTTIEIDGAVWRVVEFLHVKPGKGSAFVRTKLKNAKTGNVV

EKTFRAGETVPQAVLEKSTLQYTYKDGDDFVFMDMETYEEGRLTAATIGD

RVKYLKEGMEANVITWNGQVIEVELPNSVVLEVIETDPGVKGDTATGGTK

PAKVETGAQVMVPLFISVGERIKIDTRNDSYLGRE

>fig|243230.1.peg.304 [Deinococcus radiodurans R1] [Translation elongation factor P @ Translation initiation factor 5A]

MISVTELRNGTKVQMDGGLWECLDYSHLKMGRGGAKVVTKFRNMESGSIV

DRTFNSTEKLQDIYVEGKKMQYLYPDGDDYVFMDMETFDQVHLGKNIVSD

AAKFMKENTEVEVAMYGDKALSISLPNQVILKITQTDPGVRGDTVSGGTK

PATLETGAVVQVPLFVEQGTDVKVDTRTGQYLSRA

>fig|205921.3.peg.1898 [Streptococcus agalactiae A909] [Translation elongation factor P @ Translation initiation factor 5A]

MIEASKLKAGMTFETADGKLIRVLEASHHKPGKGNTIMRMKLRDVRTGST

FDTSYRPEEKFEQAIIETVPAQYLYKMDDTAYFMNNETYDQYEIPTVNIE

NELLYILENSEVKIQFYGTEVIGVQIPTTVELTVAETQPSIKGATVTGSG

KPATMETGLVVNVPDFIEAGQKLVINTAEGTYVSRA

>fig|212717.1.peg.1421 [Clostridium tetani E88] [Translation elongation factor P @ Translation initiation factor 5A]

MEGYIKMISAGDLRKGNTFELDGQVYTVVDFLHVKPGKGAAFVRTKLRNV

LNGGLKETTFNPTEKFQEAVIERKEMQYLYTDGELYYFMDQQTFEQIPLN

FEQVEGAIKFLKENMFAIIKFYKGEAFSVEAPNFVELQITHTDPGAKGNT

ATNVMKPATVETGAVVHVPLFINEGDSIRIDTRTGEYMERV

>fig|955.1.peg.161 [Wolbachia pipientis quinquefasciatus] [Translation elongation factor P @ Translation initiation factor 5A]

MAERANDIRPGQVLEHNGGLFSVISIMHTQPGKGGAYIQAEMKNIQTGAK

YYERFRSDATIRRAILDEEEYVYLFTEGNIVNLMHPSSYEQIVINLDLLG

EKKAYLQDNMKIKVVTYQDKIISAHVPDHVTLTVKETESVIKGQTVTASY

KPAILENGIRVNVPQFIKEGDKIVLHTPDNSYYERVKE

>fig|93062.4.peg.1181 [Staphylococcus aureus subsp. aureus COL] [Translation elongation factor P @ Translation initiation factor 5A]

MISVNDFKTGLTISVDNAIWKVIDFQHVKPGKGSAFVRSKLRNLRTGAIQ

EKTFRAGEKVEPAMIENRRMQYLYADGDNHVFMDNESFEQTELSSDYLKE

ELNYLKEGMEVQIQTYEGETIGVELPKTVELTVTETEPGIKGDTATGATK

SATVETGYTLNVPLFVNEGDVLIINTGDGSYISRG

>fig|370552.3.peg.1614 [Streptococcus pyogenes MGAS10270] [Translation elongation factor P @ Translation initiation factor 5A]

MIEASKLKAGMTFEAEGKLIRVLEASHHKPGKGNTIMRMKLRDVRTGSTF

DTTYRPDEKFEQAIIETVPAQYLYKMDDTAYFMNTDTYDQYEIPVANVEQ

ELLYILENSDVKIQFYGSEVIGVTVPTTVELTVAETQPSIKGATVTGSGK

PATLETGLVVNVPDFIEAGQKLIINTAEGTYVSRA

>fig|446469.4.peg.2605 [Sanguibacter keddiei keddieii DSM 10542] [Translation elongation factor P @ Translation initiation factor 5A]

MATTNDIKNGTVLKLDGQLWTVIDFQHVKPGKGGAFVRTKMKNVTSGKTV

DKTFNAGLKIETATVDKSDMQYLYKDGTDYVFMDQTTYDQINIPETVVGD

AANFLLESQVVNVATNEGTVLYIELPPSVILEITYSEPGLQGDRSTGGTK

PATLETGHVIQVPLFLEQGTKVKVDTRDSSYLGRVND

>fig|406557.4.peg.411 [Streptococcus pneumoniae SP6-BS73] [Translation elongation factor P @ Translation initiation factor 5A]

MIEASKLKAGMTFETADGKLIRVLEASHHKPGKGNTIMRMKLRDVRTGST

FDTSYRPEEKFEQAIIETVPAQYLYKMDDTAYFMNTETYDQYEIPVVNVE

NELLYILENSDVKIQFYGTEVIGVTVPTTVELTVAETQPSIKGATVTGSG

KPATMETGLVVNVPDFIEAGQKLVINTAEGTYVSRA

>fig|369723.3.peg.1826 [Salinispora tropica CNB-440] [Translation elongation factor P @ Translation initiation factor 5A]

MATTNDLKNGMVLNLDGELWAVVEFQHVKPGKGGAFVRTTLKNVLSGKVV

DKTFNAGTKVETATVDKRTMQYLYADGEDFVFMDLETFDQINVLGDTVGE

AANYLLPEAEATVATHEGVPLYVELPTSVVLEITYTEPGLQGDRSTGGSK

PATVETGATVQVPLFITTGEKIKVDTRDGRYLGRA

>fig|393133.3.peg.1281 [Listeria monocytogenes 10403S] [Translation elongation factor P @ Translation initiation factor 5A]

MISVNDFKTGLTIEVDNGIWRVLDFQHVKPGKGAAFVRSKLRNLRTGAIQ

EKTFRGGEKVAKAQIDNRKMAYLYADGTNHVFMDNESYEQIELPEDQIAH

ELKFLKENMEINIIMYQGETIGIDLPNTVELVVTATDPGIKGDTSSGGSK

PATLETGLVVQVPFFVNEGDKLVINTTEAAYVSRA

>fig|290318.4.peg.1582 [Prosthecochloris vibrioformis DSM 265] [Translation elongation factor P @ Translation initiation factor 5A]

MTSISNVSKGAIIRFKGEPHIVESLVHRTPGNLRAFYQANMRNLKSGRNV

EYRFSATESVDVIITERKEYQYLYMDGADYVMMDSTTFDQINVSEAAIGS

GSRFIKDGITLTIVFSDDGAILSVELPTFVEVEVTETSPASKDDRATSGT

KPAIVETGAEVNVPMFVQNGSVIRVDTRSGDYIERVKK

>fig|167539.1.peg.26 [Prochlorococcus marinus subsp. marinus str. CCMP1375] [Translation elongation factor P @ Translation initiation factor 5A]

MISSNDFRTGTTIELDGAVWRVVEFLHVKPGKGSAFVRTKLKAVQAGNVV

EKTFRAGEMVPQALLEKATLQHTYMESGDYVFMDMSTYEETRLTANQIGD

SRKYLKEGMEVNVVSWNEKPLEVELPNSVVLEVKETDPGVKGDTATGGTK

PAILETGAQIMVPLFISVGEKIKVDTRNDSYLGREN

>fig|278197.10.peg.719 [Pediococcus pentosaceus ATCC 25745] [Translation elongation factor P @ Translation initiation factor 5A]

MSTISTSDFKNGLTIEFDDNIWRIVEFQHVKPGKGSAFVRTKLKNLRNGA

VQEKTFRAGEKMEQANIETKRMQYLYNDGANYVFMDNNTYEQVEIPNERL

EEEKNYLVENMEVSIAFFGTETLGVEVPKNITLKVTATEPGIKGNTASGG

SKPATMETGLVVQVPFFVNEGDALNINTQDGTYISRA

>fig|48935.1.peg.2666 [Novosphingobium aromaticivorans] [Translation elongation factor P @ Translation initiation factor 5A]

MKISGVDIRPGNILEYEKGIWKVAKTQHTQPGKGGAFMQVEMKNLIDGRK

TNVRFRSADTVERVRLDTKDFQFLYAEGDDLVFMDVETYDQITLPSDLLG

DAAAFLQDGMTVLLEMYDERPISVQLPEQVEATIVEADAVVKGQTASSSY

KPAILDNGVRVMVPPHIESGTRIVVDVYERSYVGKAN

>fig|205919.3.peg.1944 [Bacillus anthracis str. Kruger B] [Translation elongation factor P @ Translation initiation factor 5A]

MISVNDFRTGLTIAVDNGLWQVLDFQHVKPGKGAAFVRSKLRNLRTGSVQ

EKTFRAGEKVEKAHIENRRMQYLYASGEAHVFMDNGTYEQIELGEKQIER

ELKFLKENMGVSIMTYQGEVLGVELPNTVELQVTETEPGIKGDTASNVTK

PATLETGLVVQVPIFINEGEMLIINTGEGKYVSRA

>fig|203267.1.peg.368 [Tropheryma whipplei str. Twist] [Translation elongation factor P @ Translation initiation factor 5A]

MASTSDIRNGVVLNINGQLNTVIEFQHVKPGKGGAFVRTKLKNILTGKVV

DKTFNAGASVVLENVDRRDCTYLYRDADSFVFMDLADYDQIRLTASQVAS

AANYLSDNQKVVIATHNNAPLYVDLPPSVVLAVTHTEPGVQADRSTGGTK

PATLETGYQIQVPLFITVGTRIRVDTRTGAYIGKA

>fig|279808.3.peg.1790 [Staphylococcus haemolyticus JCSC1435] [Translation elongation factor P @ Translation initiation factor 5A]

MISVNDFKTGLTISVDNGIWKVIDFQHVKPGKGSAFVRSKLRNLRTGAIQ

EKTFRAGEKVEQAMIENRRMQYLYADGDNHVFMDNETFDQTELSADYLKD

ELKYLKANMEVQIQSYEGETIGVELPKTVELEVTETEPGIKGDTATGATK

SATVETGYTLNVPLFVNEGDVLVINTSDGSYISRA

>fig|431943.4.peg.1264 [Clostridium kluyveri DSM 555] [Translation elongation factor P @ Translation initiation factor 5A]

MISAGDLRKGTTFEQDGQVYTVVDFLHVKPGKGAAFVRTKLRNVITGSVT

DTTFNPSAKLQEAVIERKEMQYLYSDGELYYFMDQETFEQIPLEYAKVQE

AIKFLKENMFAIIKFYKGEAFSVEAPNFVELQVTHTEPGVKGNTATNVLK

PATLETGAVVSVPIFVNQGETIRVDTRSGEYMERV

>fig|110662.3.peg.526 [Synechococcus sp. CC9605] [Translation elongation factor P @ Translation initiation factor 5A]

MISSNDFRTGTTIEIDGAVWRVVEFLHVKPGKGSAFVRTKLKAVKSGNVV

EKTFRAGEMLPQAMLEKSSLQHTYMEGEDYVFMDMATYEETRLSADQIGE

SRKYLKEGMEVNVVSWNDTPLEVELPNSVVLEIKETDPGVKGDTATGGTK

PAILETGAQVMVPLFLSVGEKIKVDTRNDSYLGRENG

>fig|148942.4.peg.367 [Streptococcus equi subsp. equi] [Translation elongation factor P @ Translation initiation factor 5A]

MIEASKLRAGMTFEAEGKLIRVLEASHHKPGKGNTIMRMKLRDVRTGSTF

DTTYRPDEKFEQAIIETVPAQYLYKMDETAYFMNTETYDQYEIPVANVEQ

ELLYILENSDVKIQFYGTEVIGVQVPTTVELTVTETQPSIKGATVTGSGK

PATLETGLVVNVPDFIEVGQKLIINTAEGTYVSRA

>fig|446468.4.peg.1102 [Nocardiopsis dassonvillei subsp. dassonvillei DSM 43111] [Translation elongation factor P @ Translation initiation factor 5A]

MATTNDIKNGTTLRLDGGVLWNVLEFQHVKPGKGGAFVRTKLKNVLTGKI

VDKTFNAGSKVEFANVDRREMEYLYHDGEAFIFMDTSTYDQIPVPEAVVG

DGKDFLLENTKVTVATNEGNPLYIELPAAVELEITQTDPGVQGDRSTGGT

KPATVQTGATIQVPLFISEGERVKVDTRTGDYLGRVN

>fig|282458.1.peg.1520 [Staphylococcus aureus subsp. aureus MRSA252] [Translation elongation factor P @ Translation initiation factor 5A]

MISVNDFKTGLTISVDNAIWKVIDFQHVKPGKGSAFVRSKLRNLRTGAIQ

EKTFRAGEKVEPAMIENRRMQYLYADGDNHVFMDNESFEQTELSSDYLKE

ELNYLKEGMEVQIQTYEGETIGVELPKTVELTVTETEPGIKGDTATGATK

SATVETGYTLNVPLFVNEGDVLIINTGDGSYISRG

>fig|504728.4.peg.2517 [Meiothermus ruber DSM 1279] [Translation elongation factor P @ Translation initiation factor 5A]

MISVTDLRNGTKVKMDGALWQCIEYQHQKIGRGGAKVVAKFRNLETGATV

ERSFNSGEKLEDIYVETKDLQYLYPEGDELVFMDLETYEQFHVPRGISEA

TKFLKEGMTVQGAMYNGRPLDITLPASVELKIVDTPPGVRGDTVSGGTKP

ATLETGAVVQVPLFVEAGEVIRVDTRSGEYLGRA

>fig|122586.1.peg.909 [Neisseria meningitidis MC58] [Translation elongation factor P @ Translation initiation factor 5A]

MKTAQELRAGNVFMVGNDPMVVQKTEYIKGGRSSAKVSMKLKNLLTGAAS

ETIYKADDKFDVVILSRKNCTYSYFADPMYVFMDEEFNQYEIEADNIGDA

LKFIVDGMEDQCEVTFYEGNPISVELPTIIVREVEYTEPAVKGDTSGKVM

KTARLVGGTEIQVMSYIENGDKVEIDTRTGEFRKRA

>fig|469381.4.peg.1408 [Dethiosulfovibrio peptidovorans DSM 11002] [Translation elongation factor P @ Translation initiation factor 5A]

MAQVVDTSKFYPGIKIVWQEGMWEVVEFQHHKMGRGGAVVKTKLRNLDTG

SIIENAFRSGEKFDRIVFEEKPAQFLYQDGDSYVFMDMVSYDQIYLSTEV

LGKAIKYLTDNIEVTLEMYGERIMGIELPNSVTLKIIETSPNFKGDTASG

GGKPATTETGLTVTVPMFVEVGEDIVVDTRTGAYLERAKK

>fig|331271.3.peg.2971 [Burkholderia cenocepacia AU 1054] [Translation elongation factor P @ Translation initiation factor 5A]

MKTAQELRVGNVVQIGSEAWVIAKAEYNKSGRNSAVVKMKMKNLLSNAGQ

ESVYKADDKFEVVVLDRKEVTYSYFADPMYVFMDADYNQYEVEAEMMGEA

LNYLEDGMACEVVFYNEKAISVELPTVLVREITYTEPAVKGDTSSGKVLK

NAKLATGFELQVPLFCNTGDKIEIDTRTNEYRSRA

>fig|265072.7.peg.1780 [Methylobacillus flagellatus KT] [Translation elongation factor P @ Translation initiation factor 5A]

MKTAQELRVGNVVMIGNDPLVVQKTEYNKSGRNAAVVKMKFKNLLTEAAS

EAVYKADDKFDVVILEKKEVTYSYFADPMYVFMDAEYNQYEVEADNMTDA

LNFLEDGMTCEVVFYNGKAISVDLPNSVVREITYTEPAVKGDTSGKVMKP

AKIATGFELAVPLFCDIGDKIEIDTRTLEYKNRVK

>fig|224326.1.peg.598 [Borrelia burgdorferi B31] [Translation elongation factor P @ Translation initiation factor 5A]

MAVVKSSEIEKGSFLLIKGAPHIVLEREFSKTGRGGAIVRLKLKNLKNKF

VIRETLKGADTAEAIEIYEVSAQYLYKDKDVLVFMDLETYDQVSLDLKES

ANLQDKVPFLQESEIYSLVTFDNVVIDIKLAPKIAFEVVEVEAAVKGDTV

TNAMKNITLNTGLVVKAPLFINVGDKVLINSETKEYAERIKN

>fig|340177.8.peg.370 [Chlorobium chlorochromatii CaD3] [Translation elongation factor P @ Translation initiation factor 5A]

MPSISTVSKGSIIRFKGEPHSIESLIHRTPGNLRAFYQANMKNLKTGRNV

EYRFSSSETVDVIVTERKQYQYLYRDGSDFVMMDNNTFEQINVPEVALGE

GANFMKDNINVTIVFSDDGSILQVELPTFVEVEVTDTNPASKDDRATSGT

KPAIVETGAEVNVPMFIQIGSVIRVDTRTGEYIERVKK

>fig|452471.3.peg.749 [Amoebophilus asiaticus 5a2] [Translation elongation factor P @ Translation initiation factor 5A]

MATTSDFRNGLCIEFNNDLYTIVEFQHVKPGKGGAFVRTKLKSLTNGKVI

DNTFNSGVKITTARIERKTYQFLYKDGAGYHLMDNNTFEQLDLDEKAIQN

PLLIKEGQELDVLFHQETGNFIGCELPPFVELKVTYTEPGLKGDTATRAL

KPATLETGLQIQVPLFIDNDEIIKIDTRTIAYVERVKTDI

>fig|521674.4.peg.3500 [Planctomyces limnophilus DSM 3776] [Translation elongation factor P @ Translation initiation factor 5A]

MSVKLAGDLQSGNVVIVEGNPLVVLKAQYNKSGRNAAVVKLRMKNLLTGR

VSETVVKADEKMEQIILDKKECTYSYSAPPVHVFVDGDYNQYEIDAETMA

DVEKYLMPEMTDVVEVTFYESKPISVVFPKVIIREVEYTEPVTRGDTSGK

ITKAAVLKHSKYELQVSAFVEIGDKIEIDTETGEFRRRC

>fig|382638.8.peg.346 [Helicobacter acinonychis str. Sheeba] [Translation elongation factor P @ Translation initiation factor 5A]

MAIGMSELKKGLKIELGGVPYRIVEYQHVKPGKGAAFVRAKIKSFLDGKV

IEKTFHAGDKCEEPNLVEKTMQYLYHDGDAYQFMDIESYEQIALSDSQVG

EASKWMLDGMQVQVLLYNDKAISVDVPQVVALKIVETAPNFKGDTSSASK

KPATLETGAVVQVPFHVLEGEIIKVNTETEEYLEKVK

>fig|322098.19.peg.459 [Aster yellows witches'-broom phytoplasma AYWB] [Translation elongation factor P @ Translation initiation factor 5A]

MINTNDFKTGKTIKFNNQIYQILEFLHVKPGKGSAFVRTKLRNLRTGSVI

DYTFNAGIKVQPALITKIKMQLIYVLEDNYIFMNTQNYEQLEINKYQLKD

FLKYLYEGLLVDIIFYENDEIVGISLPEKISIKVAYTEPGAKGDTKTNSL

KDATLETGLVIKVPLFINIGEKIIINTETGLYLSRDNNK

>fig|817.1.peg.3709 [Bacteroides fragilis 638R] [Translation elongation factor P @ Translation initiation factor 5A]

MINAQDIKNGTCIRMDGKLYFCIEFLHVKPGKGNTFMRTKLKDVVSGYVL

ERRFNIGEKLEDVRVERRPYQYLYKEGEDYIFMNQETFDQHPIAHDLING

VDFLLEGAVVEVVSDASTETVLYADMPIKVQMKVTYTEPGLKGDTATNTL

KPATVESGATVRVPLFISEGETIEIDTRDGSYVGRVKA

>fig|269482.1.peg.4673 [Burkholderia cepacia R1808] [Translation elongation factor P @ Translation initiation factor 5A]

MKTAQELRVGNVVQIGSDAWVIAKTEYNKSGRNAAVVKMKMKNLLTNAGQ

EAVYKADDKFDVVVLDRKEVTYSYFADPMYVFMDADYNQYEVEAEMMGEA

LNYLEDGMACEVVFYNEKAISVELPTILVREITYTEPAVKGDTSSGKVLK

NAKLATGFELQVPLFCNTGDKIEIDTRTNEYRSRA

>fig|115711.7.peg.933 [Chlamydophila pneumoniae AR39] [Translation elongation factor P @ Translation initiation factor 5A]

MVRVSTSEFRVGLRIEIDGQPYLILQNDFVKPGKGQAFNRIKVKNFLTGR

VIERTYKSGESVETADIVERSMRLLYTDQEGATFMDDETFEQEVVFWEKL

ENIRQWLLEDTIYTLVLYNGDVVAVEPPIFMELSIAETAPGVRGDTASGR

VLKPAVTNTGAKIMVPIFIDEGELVKVDTRTGSYESRVSK

>fig|354242.8.peg.580 [Campylobacter jejuni subsp. jejuni 81-176] [Translation elongation factor P @ Translation initiation factor 5A]

MASYSMGDLKKGLKIEIDGIPFKIVEYQHVKPGKGPAFVRIKIKSFIDGK

VLEKTFHAGDKCEAPNLEDKTMQYLYDDGENCQFMDTQTYEQVAISDDDV

GEAKKWMLDGMMVDVLFHNGKAIGVEVPQVVELKIIETAPNFKGDTQGSN

KKPATLETGAVVQIPFHVLEGEVIRVDTVRGEYIERANK

>fig|264198.3.peg.2849 [Ralstonia eutropha JMP134] [Translation elongation factor P @ Translation initiation factor 5A]

MKIAQELRVGNVFMMNGAPMVVQKAEYNKSGRNAAVVKMKYKNLLTEAPG

ESVFKADDKFEVVVLERRECTYSYFADPMYVFMDADYNQFEVEQDSMGDA

LNYLEDGMAVEVVFYNEKAISVEMPTTLVREIVYTEPAVKGDTSSGKVLK

GAKINTGFELQVPLFCNIGDKIEIDTRTGEYRSRAN

>fig|272559.3.peg.286 [Bacteroides fragilis ATCC 25285] [Translation elongation factor P @ Translation initiation factor 5A]

MINAQDIKNGTCIRMDGKLYFCIEFLHVKPGKGNTFMRTKLKDVVSGYVL

ERRFNIGEKLEDVRVERRPYQYLYKEGEDYIFMNQETFDQHPIAHDLING

VDFLLEGAVVEVVSDASTETVLYADMPIKVQMKVTYTEPGLKGDTATNTL

KPATVESGATVRVPLFISEGETIEIDTRDGSYVGRVKA

>fig|207559.3.peg.1286 [Desulfovibrio desulfuricans G20] [Translation elongation factor P @ Translation initiation factor 5A]

MYSTTDFRRGLRIEIDGTPFEIVDFQHFKPGKGGAIVRTKMRNLLTGRIM

DNNFRSGEKVGRPDMENRDMQFLYREDANLVFMDMTTYEQIYMPEETTEG

KAGFLKEGQTIRVLLFNGTPLAIELPAALVLEVTETEPGAKGDTVSNVTK

PATLETGIVIQVPIFVNQGDKVKVNTDTREYMGRE

>fig|246196.1.peg.3049 [Mycobacterium smegmatis str. MC2 155] [Translation elongation factor P @ Translation initiation factor 5A]

MASTADFKNGLVLQIDGQLWQIVEFQHVKPGKGPAFVRTKLKNVVSGKVV

DKTYNAGVKVETATVDRRDATYLYRDGSDFVFMDSEDFEQHPLPESLVGR

LADFLLESMPVQIAFHDGTPLYLELPVSVELEVTHTEPGLQGDRSSAGTK

PATVETGAEIQVPLFINTGDRLKVDTRDGSYLGRVNA

>fig|485918.5.peg.1916 [Chitinophaga pinensis DSM 2588] [Translation elongation factor P @ Translation initiation factor 5A]

MATTADIRTGLIIKLDNSLYSVVEFGQNKTARAAAKVWAKLKGVDNSRSI

EHTWNSGDTIYPVRVEKKAFQFLYQDDSGYNFMDNETFEQIALPETMIDA

PQFLKEGQEVGVSINTETEQPMSVELPDKIVVKVTYSEPGLKGDTATRTL

KPATVETGATVNVPLFVNEGELIRVNTKNGEYIERVKE

>fig|370551.3.peg.1550 [Streptococcus pyogenes MGAS9429] [Translation elongation factor P @ Translation initiation factor 5A]

MIEASKLKAGMTFEAEGKLIRVLEASHHKPGKGNTIMRMKLRDVRTGSTF

DTTYRPDEKFEQAIIETVPAQYLYKMDDTAYFMNTDTYDQYEIPVANVEQ

ELLYILENSDVKIQFYGSEVIGVTVPTTVELTVAETQPSIKGATVTGSGK

PATLETGLVVNVPDFIEAGQKLIINTAEGTYVSRA

>fig|286636.1.peg.1536 [Streptococcus pyogenes MGAS10394] [Translation elongation factor P @ Translation initiation factor 5A]

MIEASKLKAGMTFEAEGKLIRVLEASHHKPGKGNTIMRMKLRDVRTGSTF

DTTYRPDEKFEQAIIETVPAQYLYKMDDTAYFMNTDTYDQYEIPVANVEQ

ELLYILENSDVKIQFYGSEVIGVTVPTTVELTVAETQPSIKGATVTGSGK

PATLETGLVVNVPDFIEAGQKLIINTAEGTYVSRA

>fig|446471.4.peg.2260 [Xylanimonas cellulosilytica DSM 15894] [Translation elongation factor P @ Translation initiation factor 5A]

MATSNDIKNGTVLRIDGQLWTIIEFQHVKPGKGGAFVRTKMKNVVSGKTV

DKTFNAGIKVETANVDRRDYQFSYMDGDDFVFMDTDTWEQFHIPATVVGD

AKDYMLEGMSVMIAMNEGTPLYVELPTSVVLEITHAEPGLQGDRSTGGTK

PATLETGKEIQVPLFLEAGVKVKVDTRDGSYLGRVNE

>fig|220668.1.peg.1323 [Lactobacillus plantarum WCFS1] [Translation elongation factor P @ Translation initiation factor 5A]

MISTADFKNGLTIEVDNAIWRIVEFQHVKPGKGGAFVRSKLKNLRTGAVQ

DKTFRAGARMEQAPIEKSTMQYLYADGDSYVFMNTETYEQIEIPGDHIQD

ELKFLKENMEVQVTLYKGEVLGIDLPNTVTLEVAETEPGIKGDTASGGSK

PATLETGAIIQVPFFVKAGDKLIVNTVDSTYVSRA

>fig|164757.7.peg.2370 [Mycobacterium sp. JLS] [Translation elongation factor P @ Translation initiation factor 5A]

MASTADFKNGLVLQIDGQLWQIVEFQHVKPGKGPAFVRTKLKNVVSGKVV

DKTYNAGVKVETATVDRRDATYLYRDGSDFVFMDSEDYEQHPLPESLVGR

AADFLLESMPVQIAFHDGVPLYLELPVTVELLVASTEPGLQGDRSSAGTK

PATMETGAEIQVPLFINTGDKLKVDSRDGSYLGRVNA

>fig|257310.1.peg.1832 [Bordetella bronchiseptica RB50] [Translation elongation factor P @ Translation initiation factor 5A]

MKTAQELRVGNVIMVGKDPLVVQKTEYNKSGRNAAVVKLKFKNLLTGSGS

ESVYKADEKFDVVVLERKECTYSYFGDPMYVFMDEEYNQYEIEADSMGDA

LNYLEEAMPVEVVFYDGRAISVELPTILVREITYTEPAVRGDTSGKVLKP

AKINTGFELSVPLFCAIGDKIEIDTRTNEYRSRVN

>fig|126740.4.peg.1072 [Thermotoga sp. RQ2] [Translation elongation factor P @ Translation initiation factor 5A]

MIEVGDLKKGMFIIYDGEIYRVLEASKHFMGRGSGLIRTKLKNVKTGLVR

EVNFPSGDKVPEAELSFRKAQYLYRDGDHYYFMTLDDYEQYALSEEEIGD

AKYYLVENMEVDLVFHEGTPIGIELPTTVELTVVETEPSFKGDTVSGGGK

PAVLETGLKITVPYFIEVGDKIKVDTRTGEYVGRA

>fig|370554.3.peg.1606 [Streptococcus pyogenes MGAS10750] [Translation elongation factor P @ Translation initiation factor 5A]

MIEASKLKAGMTFEAEGKLIRVLEASHHKPGKGNTIMRMKLRDVRTGSTF

DTTYRPDETFEQAIIETVPAQYLYKMDDTAYFMNTDTYDQYEIPVANVEQ

ELLYILENSDVKIQFYGSEVIGVTVPTTVELTVAETQPSIKGATVTGSGK

PATLETGLVVNVPDFIEAGQKLIINTAEGTYVSRA

>fig|272944.1.peg.322 [Rickettsia conorii str. Malish 7] [Translation elongation factor P @ Translation initiation factor 5A]

MKISANSIRTGNILVYNNDLWVVSKTPEHTQPGKGGAYVQVEMKNLKTGT

KRNERFSSANYLEKAELEQKDYQFLYFEGDDLVLMDTKHFDQINISKEML

EEKLSFLTENMIVKVEFYNDKPLNIELPPTVILEISETDPVIKGATATAS

YKSAILENGIKVKVPQYLEIGEKIVVKTDDMTYVERAK

>fig|228410.1.peg.870 [Nitrosomonas europaea ATCC 19718] [Translation elongation factor P @ Translation initiation factor 5A]

MKTAQELRVGNVFMLGKDPMVVLKTEFTKSGRNSSVVKMKYKNLLTESPG

EAVYKADDKFDIVVLDKKEVNYSYFASPMYVFMDAEFNQYEVEEETMSDA

LSFLEDGMPCEVVFYNDKPISVELPNTVVREIIYTEPAIKGDTTGKVLKP

AKIPTGFELAVPLFCEIGDKIEIDTRTREYRSRVK

>fig|525898.4.peg.291 [Sulfurospirillum deleyianum DSM 6946] [Translation elongation factor P @ Translation initiation factor 5A]

MASISMGDLKKGLKIEINGTPYKIVEYQHVKPGKGAAFVRCKIKSFMDGK

VIEKTFHAGDKCETPNLEDKIMQFLYDDGEFLQFMDSATYEQIALTHDQV

GEAADWIIDGMNVDMLYHNGKPISVEAPQFVELKIVETPPNFKGDTQGGK

KPATLESGAVVQVPFHVVEGDVIKVDTVRGEYLEKVK

>fig|325240.9.peg.38 [Shewanella baltica OS155] [Translation elongation factor P @ Translation initiation factor 5A]

MKTAHEIRPGNVIMLDGSPWVVQKTETTRSGRNAAIVKLKLKNLLLNSGT

ETTFKGEDKLEDIILDRLDCTYSYFADPMFVFMDAEYNQYDVEAENLGDA

AAYIVDGMEETCQVTFYDGKAISVEMPTTIVREVIYTEPSARGDTSGKVM

KPATITGGGTVTVADFVKVGDKIEIDTRTGEFKKRV

>fig|325240.9.peg.2529 [Shewanella baltica OS155] [Translation elongation factor P @ Translation initiation factor 5A]

MKTAHEIRPGNVIMLDGSPWVVQKTETTRSGRNAAIVKLKLKNLLLNSGT

ETTFKGEDKLEDIILDRLDCTYSYFADPMFVFMDAEYNQYDVEAENLGDA

AAYIVDGMEETCQVTFYDGKAISVEMPTTIVREVIYTEPSARGDTSGKVM

KPATITGGGTVTVADFVKVGDKIEIDTRTGEFKKRV

>fig|222891.5.peg.149 [Neorickettsia sennetsu str. Miyayama] [Translation elongation factor P @ Translation initiation factor 5A]

MRCELQCQPVFLYLFAVSQKILGNDIRVGNVLEYKNSLYQVLKREHVKPG

KGGAFVNVEMKSIDGASKVNHRFRSDEVVFKAFLEEDECHYLFKEGDAIV

LMSLSTYEQFSVDADLFVDVDKYLKEEVVVKLLRHGDKIVGVKVQDNLAY

TVEETEPYIKGQTVTSSYKPAILNGGLLKVMVPPFIKVGDQIVVKTEDGT

YVGRTEK

>fig|315749.4.peg.2954 [Bacillus cereus subsp. cytotoxis NVH 391-98] [Translation elongation factor P @ Translation initiation factor 5A]

MISVNDFRTGLTITVDNDLWQVIEFQHVKPGKGAAFVRSKLRNLRTGAIQ

EKTFRAGEKVEKAHIENRRMQYLYASGEAHVFMDNGTYEQIELSEAQIER

ELKFLKENMEVSIMTYQGEVLGVELPNTVELKVVETEPGIKGDTASNVTK

PATLETGLVVQVPIFINEGEMLVINTAEGKYVSRA

>fig|406556.4.peg.296 [Streptococcus pneumoniae SP3-BS71] [Translation elongation factor P @ Translation initiation factor 5A]

MIEASKLKAGMTFETADGKLIRVLEASHHKPGKGNTIMRMKLRDVRTGST

FDTSYRPEEKFEQAIIETVPAQYLYKMDDTAYFMNTETYDQYEIPVVNVE

NELLYILENSDVKIQFYGTEVIGVTVPTTVELTVAETQPSIKGATVTGSG

KPATMETGLVVNVPDFIEAGQKLVINTAEGTYVSRA

>fig|262722.3.peg.501 [Mycoplasma hyopneumoniae 7448] [Translation elongation factor P @ Translation initiation factor 5A]

MINVNEFRPGITFEFENEIYVVISAQHSKQGRGQANVKTKVKNLRTGAIT

IKTFSGGERVEKAHIEKISMSFLYNDGESIVLMDDSTYEQVAIENTKITW

ELNFLTEGIKVKLRKFNNEILDIELPAKIELKITSTFDAVRGNTTTNPTK

RATLETGYEIDVPLFIKEGESVIVSTEDGKYVSRG

>fig|196164.1.peg.1737 [Corynebacterium efficiens YS-314] [Translation elongation factor P @ Translation initiation factor 5A]

MGHPLTPNTREIITVASTADFKNGLVLKVDGKLQQIVEFQHVKPGKGPAF

VRTKLKDVVTGKTVDKTWNAGVKVETATVDRRDMTYLYNDGSSYILMDDK

TFEQFELPLDAFGDAGRFLLENMRVQVSFHDGEALFGELPVSVDLRVEHT

DPGLQGDRSTGGTKPATLETGAEIQVPLFIETGNVLKVDTRDGSYLSRVN

N

>fig|376686.6.peg.2884 [Flavobacterium johnsonia johnsoniae UW101] [Translation elongation factor P @ Translation initiation factor 5A]

MASTSDIRNGLCIKFNHDIYKIIEFLHVKPGKGPAFVRTKLKSLTSGKVL

DNTFSAGHKIDVIRVETHTFQFLYPEGDEFHFMNAETFEQISLNKNILDA

PDLLKEGTNVMVQINTETDLPLSVDMPASVILEVTYAEPGVKGNTATNAT

KNATVETGANVNVPLFINEGDKIKIDTASGSYMERVKE

>fig|208435.1.peg.1650 [Streptococcus agalactiae 2603V/R] [Translation elongation factor P @ Translation initiation factor 5A]

MIEASKLKAGMTFETADGKLIRVLEASHHKPGKGNTIMRMKLRDVRTGST

FDTSYRPEEKFEQAIIETVPAQYLYKMDDTAYFMNNETYDQYEIPTVNIE

NELLYILENSEVKIQFYGTEVIGVQIPTTVELTVAETQPSIKGATVTGSG

KPATMETGLVVNVPDFIEAGQKLVINTAEGTYVSRA

>fig|264202.3.peg.144 [Chlamydophila felis Fe/C-56] [Translation elongation factor P @ Translation initiation factor 5A]

MVRVSTSEFRVGLRVEIDGQPYLILQNDFVKPGKGQAFNRIKIKNFLTGR

VIEKTFKSGESVETADVREQQMRFLYSDQEGATFMDDETFEQEVIFWDKI

ENIRQWLLEDTIYTLVLYNGNVIGVEPPIFMELTIAETAPGVRGDTASGR

VLKPAVTNTGAKIMVPIFIEEGEVVKVDTRTGSYESRVSK

>fig|68909.1.peg.769 [Deinococcus geothermalis DSM11300] [Translation elongation factor P @ Translation initiation factor 5A]

MISVTELRNGTKVEMDGGLWECLEYSHLKMGRGGAKVVTKFRNMETGSIV

DRTFNSGEKLQDIYVEGKKMQYLYRDGDDYVFMDMETFDQVHLPPALVGD

TAKFMKENTEVEVAMYGDKALSITLPNQVILKIVQTDPGVRGDTVSGGTK

PATLETGAVVQVPLFVEQGTDVKVDTRTGQYLSRA

>fig|314254.3.peg.2579 [Oceanicaulis alexandrii HTCC2633] [Translation elongation factor P @ Translation initiation factor 5A]

MKINGNEIKPGNVIKHQDTLWVAVKADHVKPGKGGAFAQVELKNLLDGRK

LNERFRSADKVERVRLEQKDHQFLYPEGEMLVFMDTETYEQTNLPIDFVG

EDRAAYLTDGMMVVLEIYEEKPIGIELPKHVELEVIETEPVVKGQTAANS

FKPAILTGNVRTAVPPFVGVGERIVVATEDGSYVRRAE

>fig|122587.1.peg.1045 [Neisseria meningitidis Z2491] [Translation elongation factor P @ Translation initiation factor 5A]

MKTAQELRAGNVFMVGNDPMVVQKTEYIKGGRSSAKVSMKLKNLLTGAAS

ETIYKADDKFDVVILSRKNCTYSYFADPMYVFMDEEFNQYEIEADNIGDA

LKFIVDGMEDQCEVTFYEGNPISVELPTIIVREVEYTEPAVKGDTSGKVM

KTARLVGGTEIQVMSYIENGDKIEIDTRTGEFRKRA

>fig|406561.4.peg.2271 [Streptococcus pneumoniae SP18-BS74] [Translation elongation factor P @ Translation initiation factor 5A]

MIEASKLKAGMTFETADGKLIRVLEASHHKPGKGNTIMRMKLRDVRTGST

FDTSYRPEEKFEQAIIETVPAQYLYKMDDTAYFMNTETYDQYEIPVVNVE

NELLYILENSDVKIQFYGTEVIGVTVPTTVELTVAETQPSIKGATVTGSG

KPATMETGLVVNVPDFIEAGQKLVINTAEGTYVSRA

>fig|314269.3.peg.1408 [Aurantimonas sp. SI85-9A1] [Translation elongation factor P @ Translation initiation factor 5A]

MGWEPRRTGCRQRNGRCWPSGWKTPTRPSTTSSAGSIRRPRSRRWRQPTG

SVRAERHSRNPPRGLNGGVNYGLEPRPAEIVRRRHGARHRPAAPFRRASL

PINNAAAAVRPDRRRRENPSRASMKINGNEIRPGNVIEHNGGLWAAVKTA

HVKPGKGGAFAQVELKNLIDGTKLNERFRASETVERVRLEQKDYQFLYAE

GEMLVFMDTETYEQLELQKDFVGERAAFLQDGMMVTVESHEDKPIGIKLP

DQVVLAIVEADPVVKGQTAASSYKPAVMENGMRVMVPPFIETGERILVDT

NEITYLRRAD

>fig|471855.4.peg.2555 [Slackia heliotrinireducens DSM 20476] [Translation elongation factor P @ Translation initiation factor 5A]

MATISTTDFKTGMCILYKNKKCTVIEYQHVKPGKGPAFVRMKVRDLSTGR

VLTDTVRPETKFETVMLQQQKMQYLYNDGTDFYFMDPDTYEQVSLEADHV

GETAQWLKENDEVTLSYADGELMGVEPQMFVELEVTMTEPGFKGDTVQGS

TKPATLETGAEVKVPMYIEIGERVQIDTRDGRFVKRV

>fig|288681.3.peg.473 [Bacillus cereus ZK] [Translation elongation factor P @ Translation initiation factor 5A]

MISVNDFRTGLTIAVDNGLWQVLDFQHVKPGKGAAFVRSKLRNLRTGSVQ

EKTFRAGEKVEKAHIENRRMQYLYASGEAHVFMDNGTYEQIELGEKQIER

ELKFLKENMEVSIMTYQGEVLGVELPNTVELQVTETEPGIKGDTASNVTK

PATLETGLVVQVPIFINEGEMLIINTGEGKYVSRA

>fig|317655.9.peg.1248 [Sphingopyxis alaskensis RB2256] [Translation elongation factor P @ Translation initiation factor 5A]

MKITGVEIRPGNIIEFEGGIWKVTKIQHTQPGKGGAYMQVEAKNLIDGRK

LNNRFRSADTVEKVRLDTKDFQYLYAEGDDLVFMDKDTYEQITIGKDVVG

EAHEFLQDGMDVVLELWEERPISVELPEQIEATIVEADAVVKGQTASSSY

KPAILDNGVRVMVPPHITSGTRIVVNVYDREYVRRAD

>fig|320388.3.peg.658 [Burkholderia mallei SAVP1] [Translation elongation factor P @ Translation initiation factor 5A]

MKTAQELRVGNVVMIGNDAWVVSKTEYNKSGRNAAVVKMKLKNLLNGGGQ

ESVYKADDKFEVVVLDRKEVTYSYFADPMYVFMDADYNQYEVEAEMMGDA

LNYLEDGMACEVVFYNEKAISVELPTILVREITYTEPAVKGDTSSGKVLK

NAKLATGFELQVPLFCNTGDKIEIDTRTNEYRSRA

>fig|312153.3.peg.427 [Polynucleobacter sp. QLW-P1DMWA-1] [Translation elongation factor P @ Translation initiation factor 5A]

MKTAQELRVGNVVMIGTDAMVVLKAEYSRSGRNSSVVKMKFKNLLTGAPN

EGVFKADDKFDVVILDKKECTYSYFADPMYVFMDTEYNQYEVEAEFMGDA

LNYLEESMPCEVVFYEGKALSVAMPNSLVREIIYTEPAVKGDTSSGKVLK

NAKLATGYELQVPLFCNTGDKIEIDTRTGEYRSRAN

>fig|280354.3.peg.1741 [Bacillus anthracis str. CNEVA-9066] [Translation elongation factor P @ Translation initiation factor 5A]

MISVNDFRTGLTIAVDNGLWQVLDFQHVKPGKGAAFVRSKLRNLRTGSVQ

EKTFRAGEKVEKAHIENRRMQYLYASGEAHVFMDNGTYEQIELGEKQIER

ELKFLKENMEVSIMTYQGEVLGVELPNTVELQVTETEPGIKGDTASNVTK

PATLETGLVVQVPIFINEGEMLIINTGEGKYVSRA

>fig|471856.4.peg.750 [Jonesia denitrificans DSM 20603] [Translation elongation factor P @ Translation initiation factor 5A]

MATTNDIKNGSVLRLDGQLWAVLEFQHVKPGKGGAFVRTKIRNITSGKLV

DKTFNAGMKIEMAIVDKSDMQYLYQDGEDYVFMDLTTYDQITIPAATVGD

AKNFLLENGTATVATNDGTVLYIELPASITTVITYTEPGLQGDRSSAGTK

SATIETGYEIQVPLFLEQGTKVKVDTRDGSYLGRVND

>fig|89187.3.peg.1213 [Roseovarius nubinhibens ISM] [Translation elongation factor P @ Translation initiation factor 5A]

MPKINGNEIRPGNVLEHNGGLWSAVKVDHVKPGKGGAFAQVEMRNLRNGS

KLNERFRSADKVERVRLEQKDQQFLYETDGILTFMDSETYEQIELPADLL

GDRRPFLQDGMMITVEFYENEALNATLPQKVVCKIVETEPVVKGQTAANS

FKPATLENGVKVMVPPFVGQDEEIVVNTETMEYAERA

>fig|269484.4.peg.69 [Ehrlichia canis str. Jake] [Translation elongation factor P @ Translation initiation factor 5A]

MAERGSDIRPGHILEHNNALYLVVKVMHTQPGKGGAYIQAEMKNLKTGAK

QYERFRTDGDIKRAIVDESDYQYIYGDGSMITVMHLKTYEQITISKDILG

DKSIYLQDNIVITLVFYNGEIISAKVPDYVTLRVIETEAVIKGQTVSSSS

YKVAMLENNQRISVPTFIKPGDKIVVYTPDDSYYERAKG

>fig|243161.4.peg.455 [Chlamydia muridarum Nigg] [Translation elongation factor P @ Translation initiation factor 5A]

MVRVSTSEFRVGLRVEIDGQPYVILQNDFVKPGKGQAFNRIKVKNFLTGR

VIEKTFKSGESIETADVREQQMRLLYTDQEGATFMDDETFEQELIFWDKL

ENIRQWLLEDTVYTLVRYNGDVISVEPPIFMELSIAETAPGVRGDTASGR

VLKPATTNTGAKIMVPIFIEEGEVVKVDTRTGSYESRVSK

>fig|316274.3.peg.4837 [Herpetosiphon aurantiacus ATCC 23779] [Translation elongation factor P @ Translation initiation factor 5A]

MVSTGEVRKGLTLIIDGELFRVMEANHVKQGRGTAFLRLTLRNVRTSATT

VKTFMAGERFEVARLSTRNVQYLYREDNYIYVMNTETYDQFPVSVDLLED

ALLYIRENETFDVLTYEDEVLDISLPPSVEMVVVETEPNYKGDTASGGGK

PATTDTGLVVQVPSFVAVGERIRVDTTNGKYITRI

>fig|281309.1.peg.3898 [Bacillus thuringiensis serovar konkukian str. 97-27] [Translation elongation factor P @ Translation initiation factor 5A]

MISVNDFRTGLTIAVDNGLWQVLDFQHVKPGKGAAFVRSKLRNLRTGSVQ

EKTFRAGEKVEKAHIENRRMQYLYASGEAHVFMDNGTYEQIELGEKQIER

ELKFLKENMEVSIMTYQGEVLGVELPNTVELQVTETEPGIKGDTASNVTK

PATLETGLVVQVPIFINEGEMLIINTGEGKYVSRA

>fig|419947.3.peg.2564 [Mycobacterium tuberculosis H37Ra] [Translation elongation factor P @ Translation initiation factor 5A]

MATTADFKNGLVLVIDGQLWTITEFQHVKPGKGPAFVRTKLKNVLSGKVV

DKTFNAGVKVDTATVDRRDTTYLYRDGSDFVFMDSQDYEQHPLPEALVGD

AARFLLEGMPVQVAFHNGVPLYIELPVTVELEVTHTEPGLQGDRSSAGTK

PATLQTGAQINVPLFINTGDKLKVDSRDGSYLGRVNA

>fig|314232.3.peg.1002 [Loktanella vestfoldensis SKA53] [Translation elongation factor P @ Translation initiation factor 5A]

MPKINGNEIRPGNVLEHEGSLWGAVKVDHVKPGKGGAFAQVELKNLRDGR

KLNERFRSADKVERVRLDQKDQQFLFETDGMLTFMDSETYEQIALPVDIL

GDRRPFLQDGMVVHIEYYGEEALNVTLPQKVVCKVAETEPVVKGQTAANS

FKPAILDNGVRVMIPPFVGTDEDIIVNTELFEYVERA

>fig|350688.3.peg.1597 [Alkaliphilus oremlandi oremlandii OhILAs] [Translation elongation factor P @ Translation initiation factor 5A]

MISASDFRKGVTFEMNGEPYVVLDFQHVKPGKGAAFVRTKYKNLKNGGTR

EEAFNPSDKFPKAHIETKEMQYLYSDGELYYFMDNETFEQTPLTYEEVED

AIKFLKENDNATIKFYHGKPFQVDPPNFVELQITETEPGVKGDTASNVTK

TATVETGAVIHVPLFVNEGDTVRIDTRTGEYMSRV

>fig|398580.3.peg.2082 [Dinoroseobacter shibae DFL 12] [Translation elongation factor P @ Translation initiation factor 5A]

MRLASDTSARYRSGHSSFNLLPWFHRMPKINGNEIRPGNVLEHNGGLWAA

VKVDHVKPGKGGAFAQVELRNLRNGSKLNERFRSADKVERVRLEQKDQQF

LYESDGMLVFMDNETYEQIELPAEILGDRRPFLQDGMTIQIEYYESEALN

ASLPQKVTCKVIETEPVVKGQTAANSFKPAILDNGVKVMVPPFVGQDEAI

VVNTETMEYSERA

>fig|2433.3.peg.3223 [Roseobacter Sp. GAI101] [Translation elongation factor P @ Translation initiation factor 5A]

MPKINGNEIRPGNVLEHNGGLWAAVKVDHVKPGKGGAFAQVEMRNLRNGS

KLNERFRSADKVEKVRLEQKDQQFLYEDAGMLVLMDTQTYDQVQLSAELL

GDRRPFLQDGMMVVVEYHEDEALNASLPQKVTCKIVETEPVVKGQTAANS

FKPAILDNGVKVMVPPFVGPDEDIIVNTDTMEYSERA

>fig|261591.3.peg.3124 [Bacillus anthracis str. Vollum] [Translation elongation factor P @ Translation initiation factor 5A]

MISVNDFRTGLTIAVDNGLWQVLDFQHVKPGKGAAFVRSKLRNLRTGSVQ

EKTFRAGEKVEKAHIENRRMQYLYASGEAHVFMDNGTYEQIELGEKQIER

ELKFLKENMEVSIMTYQGEVLGVELPNTVELQVTETEPGIKGDTASNVTK

PATLETGLVVQVPIFINEGEMLIINTGEGKYVSRA

>fig|386043.6.peg.1326 [Listeria welshimeri serovar 6b str. SLCC5334] [Translation elongation factor P @ Translation initiation factor 5A]

MISVNDFKTGLTIEVDNGIWRVLDFQHVKPGKGAAFVRSKLRNLRTGAIQ

EKTFRGGEKVAKAQIDNRKMAYLYADGSNHVFMDNESYEQIELPEDQIAH

ELKFLKENMEINIIMYQGETIGIDLPNTVELVVTATDPGIKGDTSSGGSK

PATLETGLVVQVPFFVNEGDKLVINTTEAAYVSRA

>fig|198466.1.peg.1574 [Streptococcus pyogenes MGAS315] [Translation elongation factor P @ Translation initiation factor 5A]

MIEASKLKAGMTFEAEGKLIRVLEASHHKPGKGNTIMRMKLRDVRTGSTF

DTTYRPDEKFEQAIIETVPAQYLYKMDDTAYFMNTDTYDQYEIPVANVEQ

ELLYILENSDVKIQFYGSEVIGVTVPTTVELTVAETQPSIKGATVTGSGK

PATLETGLVVNVPDFIEAGQKLIINTAEGTYVSRA

>fig|320372.3.peg.4552 [Burkholderia pseudomallei 1710b] [Translation elongation factor P @ Translation initiation factor 5A]

MKTAQELRVGNVVMIGNDAWVVSKTEYNKSGRNAAVVKMKLKNLLNGGGQ

ESVYKADDKFEVVVLDRKEVTYSYFADPMYVFMDADYNQYEVEAEMMGDA

LNYLEDGMACEVVFYNEKAISVELPTILVREITYTEPAVKGDTSSGKVLK

NAKLATGFELQVPLFCNTGDKIEIDTRTNEYRSRA

>fig|261594.1.peg.4383 [Bacillus anthracis str. 'Ames Ancestor'] [Translation elongation factor P @ Translation initiation factor 5A]

MISVNDFRTGLTIAVDNGLWQVLDFQHVKPGKGAAFVRSKLRNLRTGSVQ

EKTFRAGEKVEKAHIENRRMQYLYASGEAHVFMDNGTYEQIELGEKQIER

ELKFLKENMEVSIMTYQGEVLGVELPNTVELQVTETEPGIKGDTASNVTK

PATLETGLVVQVPIFINEGEMLIINTGEGKYVSRA

>fig|217.1.peg.235 [Helicobacter mustelae 43772] [Translation elongation factor P @ Translation initiation factor 5A]

MAIGMSELKKGLKIEIDGVPYRIVEYQHVKPGKGAAFVRAKIKSFFDGRV

IEKTFHAGDKCEEPNLVERTMQYLYHDGEAFQFMDTETYEQLALSDDQVG

DVAKWMLDGMNVQVLFHNGKAISVDVPQIVQLKIIETAPNFKGDTSSGGK

KPATLETGVVVQIPFHVLEGEVIRVNTETHEYVERVK

>fig|204773.3.peg.928 [Herminiimonas arsenicoxydans] [Translation elongation factor P @ Translation initiation factor 5A]

MKPAKEIRVGNIIMVDSKPMIVLRSDVNGSSRTGFTYKWKMKNLLTNTPM

ENVFRGDDKFDVIVLDKKPVTYSYFADPLFVFMDEEYNQYEIEEENLGDA

LHYLKEGMECEAVFYDGKAISVELPITIARQVVYSEPAVKGNTSGNVLKE

AIIENAVEAHRHTVQVPLFVSTDDVIEIDSRTNEYKRVVRN

>fig|399599.3.peg.2234 [Shewanella baltica OS195] [Translation elongation factor P @ Translation initiation factor 5A]

MKTAHEIRPGNVIMLDGSPWVVQKTETTRSGRNAAIVKLKLKNLLLNSGT

ETTFKGEDKLEDIILDRLDCTYSYFADPMFVFMDAEYNQYDVEAENLGDA

AAYIVDGMEETCQVTFYDGKAISVEMPTTIVREVIYTEPSARGDTSGKVM

KPATITGGGTVTVADFVKVGDKIEIDTRTGEFKKRV

>fig|326298.3.peg.1249 [Thiomicrospira denitrificans ATCC 33889] [Translation elongation factor P @ Translation initiation factor 5A]

MATIGMGDIKKNIRLIIGEVPCKVIEFQHVKPGKGAAFVRMKAKSFLNGR

VFEKTVHAGDKFEVPEITFKTMQYLYDDGEQYQFMDNDSYEQIGLSYEQC

DDASKWFKDGIQVDMIFYKGNAISVSAPEVMELLITDTPPNFKGDTSSGS

KKPATLETGAVVQVPYHVLEGDTIRVNTVDCEYLEKVK

>fig|403833.5.peg.11 [Petrotoga mobilis SJ95] [Translation elongation factor P @ Translation initiation factor 5A]

MIDVGDLRKGDMIVYQNEMYRVIEANKHFMGRGSGLIRTRLKSVITGLIK

EVSFSSGEKVEEADISFRKAQYLYNDGDHYYFMLLDTYEQYSLPAQELED

EKFYLTENLEVDLIFFNGNPVSIQLPTVVVLTVIDTEPNFKGNTVSGGGK

PATLETGLKTTVPFFVERGQKIKVDTRTGDYLERA

>fig|273036.3.peg.1216 [Staphylococcus aureus RF122] [Translation elongation factor P @ Translation initiation factor 5A]

MISVNDFKTGLTISVDNAIWKVIDFQHVKPGKGSAFVRSKLRNLRTGAIQ

EKTFRAGEKVEPAMIENRRMQYLYADGDNHVFMDNESFEQTELSSDYLKE

ELNYLKEGMEVQIQTYEGETIGVELPKTVELTVTETEPGIKGDTATGATK

SATVETGYTLNVPLFVNEGDVLIINTGDGSYISRG

>fig|318167.10.peg.1846 [Shewanella frigidimarina NCIMB 400] [Translation elongation factor P @ Translation initiation factor 5A]

MKTAHEIRPGNVIMLDGSPWVVQKTETTRSGRNAAIVKLKLKNVLLDSGT

EQTFKGEDKLDDIILERLDCTYSYFADPMYVFMDEEYNQYDVEADNLGDA

ADYIIDGMEDVCQVTFYEGKAISVELPVHIVREVIYTEPSARGDTSGKVM

KPATITGGGTVTVADFVKVGDKIEIDTRTGEFKKRV

>fig|272622.8.peg.857 [Lactococcus lactis subsp. cremoris SK11] [Translation elongation factor P @ Translation initiation factor 5A]

MVLAKDLKSGMTFLNGEKLLRVMEASHHKPGKGNTIMRMKLKDVRSGSTF

DDTYRPEDKFEQAVIETVTAQYLYSMDDIANFMNNETYEQYEIPVEQVKD

ELLYVLENTDVKIQFYGTEVIGIQLPTTVVLEVTETQPSIKGATVTGSGK

PATMETGLVVNVPDFVEVGTKLEINTQTGEYLKRA

>fig|314264.3.peg.4029 [Roseovarius sp. 217] [Translation elongation factor P @ Translation initiation factor 5A]

MPKINGNEIRPGNVLEHNGGLWAAVKVDHVKPGKGGAFAQVEMKNLRNGT

KLNERFRSADKVERVRLEQKDQQFLYEENGKLVFMDTETYEQIELPADLL

GERRPFLQDGMTITVEFHNSEALHASLPQKVTCKIVETEPVVKGQTAANS

FKPAILDNGVKVMVPPFVGPDEDIIVNTETMEYSERA

>fig|525919.4.peg.975 [Anaerococcus prevoti prevotii DSM 20548] [Translation elongation factor P @ Translation initiation factor 5A]

MISANDLRKGVTFVYDNDVYQIVDFQHVKPGKGAAFVRAKIRSVMNGGAK

DVTFNPNEKFEQAVISTKEMSYLYNDGQLYYFMDPETFEQIGIEYEAVKD

AIIYVKENEPVQIKFYEGKPFQIEAPNFVELKVEETEPAIKGDTATNVTK

PATVETGAVIQVPVFVNEGDVIKIDTRTGDYLSRV

>fig|95659.5.peg.535 [Ureaplasma urealyticum serovar 5] [Translation elongation factor P @ Translation initiation factor 5A]

MATIIQAKDLRAGHTFLYKGSIYQVIENSFNKTAMREGIVKCKVKNLRTG

AITVEVLTGEKVEQAIIEKSKMTFSYDDGSGYVFMDNETYEQISIPYNQL

SWEKNFIEEGTEVSVMRYDGELMGVSLPDQLVVTIVEAEEAVQGNSVQNA

TKRAWLASKWEFQVPQFIKSGEKVIINPSNGQYVGRAK

>fig|470137.3.peg.1152 [Brucella suis ATCC 23445] [Translation elongation factor P @ Translation initiation factor 5A]

MKINGNEIRPGNVIEHEGGLWVAVKTNAVKPGKGGAYNQVELKNLINGTK

LNERFRAAETVERVRLEQKDFSFLYEQGEALIFMDTETYEQLELQKDFVG

DRAAFLQDGMMVTVELYEEKPIGIRLPDQVTLAITEADPVVKGQTAASSY

KPAVLENGIRILVPPFIASGERVIVDTNELTYISRA

>fig|402612.4.peg.949 [Flavobacterium psychrophilum JIP02/86] [Translation elongation factor P @ Translation initiation factor 5A]

MASTSDIRNGLCIKFNHDIYKIIEFLHVKPGKGPAFVRTKLRSLSNGKVL

DNTFSAGHKIDEVRVETHTYQYLYAEGDQFHFMNIESFEQITLDKKILDN

PGLLKEGTNVMVQVNTETDLPLSVDMPASIILEVTYAEPGVKGNTATNAT

KSATVETGASINVPLFINEGDKIKIDTASGSYMERVK

>fig|523794.4.peg.500 [Leptotrichia buccalis DSM 1135] [Translation elongation factor P @ Translation initiation factor 5A]

MKPAAELRQGSTYRKNNIPYLILKAERHQSTSGKRQRAAEVKFKTKELIS

GKIQEITVLATELMDDIILDRNQMQFLYEIDGEYNFMDQETFEQIALSTE

DLGDAVNFLEEEMIIQVLMYEGTPVGVELPNTVIREVTYTEPGLKGDTIG

RATKPATVSTGYTLQVPLFVAIGDKIKIDTRTGEYIERAN

>fig|390235.3.peg.1467 [Pseudomonas putida W619] [Translation elongation factor P @ Translation initiation factor 5A]

MKTGKELKPGTVLRIDNDPWLVQKAEFTKSGRNSAIMKTKLKNLLTGYKT

ETVYGADDKLDDVILDRKEATLSFISGDSYTFMDTTDYTMYELNAEDIDA

VLPYIEEGMEDICEAVFFEGRLVSVELPTTISRQVVYTENAARGDTSGKV

MKPAKLKNGTEIQVADFIQIDEWIDIDTRDNSFKGRSKK
